# Suggestion for item allocation to 8 nursing activity categories of the Korean Nursing Licensing Examination: a survey-based descriptive study

**DOI:** 10.3352/jeehp.2023.20.18

**Published:** 2023-06-12

**Authors:** Kyunghee Kim, So Young Kang, Younhee Kang, Youngran Kweon, Hyunjung Kim, Youngshin Song, Juyeon Cho, Mi-Young Choi, Hyun Su Lee

**Affiliations:** 1 College of Nursing, Chung-Ang University, Seoul, Korea; 2 College of Nursing, Catholic University of Pusan, Busan, Korea; 3 College of Nursing, Ewha Womans University, Seoul, Korea; 4 College of Nursing, Chonnam National University, Gwangju, Korea; 5 School of Nursing, Hallym University, Chuncheon, Korea; 6 College of Nursing, Chungnam National University, Daejeon, Korea; 7 Department of Nursing, Suwon Science College, Suwon, Korea; 8 Department of Nursing Science, Chungbuk-National University, Cheongju, Korea; 9 Department of Nursing, Kyungmin University, Uijeongbu, Korea; Hallym University, Korea

**Keywords:** Licensure, Nursing education, Nursing license, Republic of Korea

## Abstract

**Purpose:**

This study aims to suggest the number of test items in each of 8 nursing activity categories of the Korean Nursing Licensing Examination, which comprises 134 activity statements including 275 items. The examination will be able to evaluate the minimum ability that nursing graduates must have to perform their duties.

**Methods:**

Two opinion surveys involving the members of 7 academic societies were conducted from March 19 to May 14, 2021. The survey results were reviewed by members of 4 expert associations from May 21 to June 4, 2021. The results for revised numbers of items in each category were compared with those reported by Tak and his colleagues and the National Council License Examination for Registered Nurses of the United States.

**Results:**

Based on 2 opinion surveys and previous studies, the suggestions for item allocation to 8 nursing activity categories of the Korean Nursing Licensing Examination in this study are as follows: 50 items for management of care and improvement of professionalism, 33 items for safety and infection control, 40 items for management of potential risk, 28 items for basic care, 47 items for physiological integrity and maintenance, 33 items for pharmacological and parenteral therapies, 24 items for psychosocial integrity and maintenance, and 20 items for health promotion and maintenance. Twenty other items related to health and medical laws were not included due to their mandatory status.

**Conclusion:**

These suggestions for the number of test items for each activity category will be helpful in developing new items for the Korean Nursing Licensing Examination.

## Introduction

### Background/rationale

The Korean Nursing Licensing Examination (KNLE) is a representative criterion-referenced assessment, based on the latest guidelines for nursing practice. It evaluates the minimum knowledge and skills that nursing graduates must possess to efficiently perform their duties [1]. However, the subjects that this licensing examination should cover are under constant debate. It has been suggested that the learning objectives of the 8 major nursing subjects overlap and that the KNLE may not be adequately equipped to appropriately evaluate the job competency of new nursing graduates [2,3]. Hence, continuing efforts are being made to verify the job-based knowledge of nursing graduates and to resolve the issue of overlap among the objectives of nursing subjects [4-7]. As part of this effort, Kim et al. [1] reviewed the feasibility of linking nurse jobs with the licensing examination. Song et al. [3] proposed subjects for an integrated nursing examination by extracting 1,303 statements from 481 knowledge statements. Consequently, 134 activity statements based on graduate nurse job activities in the field of nursing practice and 481 knowledge statements have been developed for the KNLE. These knowledge statements can be grouped into the following 8 activity categories: management of care and improvement of professionalism, safety and infection control, management of potential risk, basic care, physiological integrity and maintenance, pharmacological and parenteral therapies, psychosocial integrity and maintenance, and health promotion and maintenance. However, despite ongoing efforts to design a more appropriate licensing examination, the process of sharing information, collecting opinions, and securing agreement among academic societies and related organizations, such as those involved in nursing education, and nursing practice experts is still insufficient.

### Objectives

This study aims to suggest the number of test items in each of 8 nursing activity categories of the KNLE. The specific goals of the study were as follows: first, to elicit opinions on the distribution of the number of test items from 7 major academic societies affiliated with the Korean Society of Nursing Science; and second, to obtain perspectives from 4 professional associations on those opinions from 7 academic societies. It is hoped that the results will help resolve redundancy among the objectives of the 8 nursing subjects by proposing job-based item development for the KNLE.

## Methods

### Ethics statement

Institutional Review Board approval was not required for this process, because the survey questionnaire items did not contain information about the individual characteristics of the participants. Instead, it had information about the policies of societies and associations. No sensitive or individually identifiable information was obtained from the participants.

### Study design

This was a survey-based descriptive study and content analysis, described according to the STROBE (Strengthening the Reporting of Observational studies in Epidemiology) statement and 6 steps for conducting thematic analysis [8].

### Setting

**Fig. 1.**
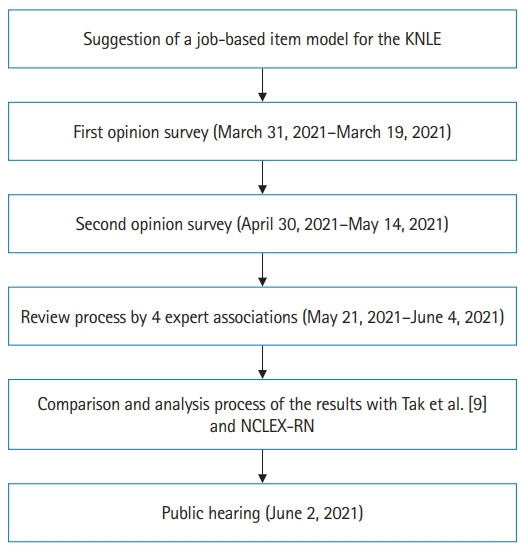
Flow of the study. KNLE, Korean Nursing Licensing Examination; NCLEX-RN, National Council License Examination for Registered Nurses.

A job-based item model for the KNLE to resolve redundancy among the objectives of the 8 nursing subjects was provided by the authors (Supplement 1). Two opinion surveys were conducted from members of 7 academic societies to assess the suitability of this model. The first opinion survey was conducted from March 19 to 31, 2021. The second opinion survey was conducted from April 30 to May 14, 2021, after sharing the results of the first survey. Approximately 12–27 members from all academic societies and associations participated in the opinion-collection process. Next, 4 experts from 4 nursing associations reviewed the opinion-survey results from May 21 to June 4, 2021. The review results were summarized by content analysis. Official e-mails were sent to each academic society and association, and the executives of each academic society and association gave their opinions. After summarizing the survey and analysis results, these results were compared with those reported by Tak et al. [9] and the National Council License Examination for Registered Nurses (NCLEX-RN) of the United States [10]. The overall workflow of the study is depicted in Fig. 1. Although a public hearing was held, the content was not reflected in this article. For more information on the public hearing, refer to Supplement 2.

### Participants

Seven academic societies participated in this survey, including the following: the Korean Academic Society of Community Health Nursing, the Korean Academic Society of Nursing Administration, the Korean Academic Society of Fundamentals of Nursing, the Korean Society of Adult Nursing, the Korean Academic Society of Child Health Nursing, the Korean Academic Society of Psychiatric and Mental Health Nursing, and the Korean Society of Women Health Nursing. The 4 expert associations participating in the review were the Korean Society of Nursing Science, the Korean Association of College of Nursing, the Korean Deans Association of Nursing College, and the Korean Hospital Nurses Association. The executives from the 7 academic societies included nursing education experts, while the reviewers from the associations included both nursing educators and practice experts.

### Variables

The primary outcome was the number of items for each category.

### Data sources/measurement

The results of the opinion survey of the KNLE among the 7 academic societies and 4 associations were used as data sources. The survey questionnaire for 7 academic societies consisted of the items and the responses by the participants (Supplement 3). A more precise source report for the article is available in Supplement 2.

### Bias

There was no bias in selecting participants because this was a descriptive study in which no comparison was conducted.

### Study size

We did not perform any sample size estimation because this was a descriptive study without any further analysis.

### Statistical methods

The collected quantitative data were organized through content analysis by focusing on key concepts and analyzed with descriptive statistics such as frequencies and percentages.

## Results

### Participants

Table 1 presents the participating societies and associations.

### Number of items suggested for the 8 categories by 7 academic societies

The KNLE items comprising 8 activity categories based on the results of the second opinion survey of the 7 member societies are listed in Table 2 (Dataset 1). The exam items included the following: management of care and improvement of professionalism, 41 items; health promotion and maintenance, 33 items; basic care, 37 items; physiological integrity and maintenance, 63 items; psychosocial integrity and maintenance, 41 items; safety and infection control, 16 items; pharmacological and parenteral therapies, 11 items; and management of potential risk, 31 items. In total, 273 items were confirmed. Two items on nursing history were exempted. Another 20 test items related to medical laws were also exempted.

### Opinions of experts from 4 professional associations

The 4 professional associations emphasized that the safety and infection control activity category and pharmacological and parenteral therapies activity category play major roles in clinical and community nursing practice in real-life situations. Therefore, in the guideline on item-making, the proportion of items in the pharmacological and parenteral therapy activity category increased from 10% (in the guideline on item-making presented by Tak et al. [9] in 2019) to 12%, and that of the safety and infection control activity category also increased from 10% to 12%. The results of the opinion survey of the 4 expert associations on the KNLE standards for each activity category presented by the 7 academic societies mentioned above are summarized in Table 3.

### Comparison with research on other examination items

We compared the results obtained with those of the NCLEX-RN evaluation conducted in 2019 [9] and those reported by Tak et al. [9] in a study that was also conducted in 2019 (Table 4). Among the items presented by Tak et al. [9], the percentage of items related to the management of care and improvement of professionalism, basic care, psychosocial integrity and maintenance, and health promotion and maintenance activity categories were the same as that determined by the opinion surveys conducted for this study. In the examination subjects developed by Tak et al. [9], the percentage of items in the management of potential risk activity category was 16% and that of items in the physiological integrity and maintenance activity category was 20%, which were higher than those in the NCLEX-RN Test Plan (2019) [10]. Referring to the opinion-survey results, we noted that the maximum percentage of the management of risk potential activity category in the distribution of the NCLEX-RN Test Plan (2019) was 15% (9%–15%) [10]. The percentage of the physiological integrity and maintenance activity category in the NCLEX-RN Test Plan (2019) was adjusted to 17%, corresponding to the maximum range of the distribution (11%–17%) [10]. In the opinion survey of the academies, the percentages of items in the safety and infection control and pharmacological and parenteral therapies activity categories were 4%–6%p lower than those in the item model suggested by Tak et al. [9] and 6%–10%p lower than those in the NCLEX-RN Test Plan (2019) [10].

## Discussion

### Interpretation

This study proposes a guideline for item-making based on the item model of Tak et al. [9] in 2019 and the NCLEX-RN Test Plan (2019 NCLEX-RN), opinion surveys of 7 academic societies and 4 associations, interviews with experts on clinical and community nursing practice, and the results of a content validity survey. The distribution of items in 4 activity categories—management of care and improvement of professionalism, basic care, physiological integrity and maintenance, and health promotion and maintenance—were maintained. However, the number of items in the management of potential risk and psychosocial integrity and maintenance areas were adjusted downward. The number of items in the areas of pharmacological and parenteral therapies and safety and infection control was increased.

In the expert opinion survey of the associations, some differences were noted in the understanding of the following 4 activity categories: management of potential risk, basic care, physiological integrity and maintenance, and pharmacological and parenteral therapies. To gain a clear understanding of the 8 activity categories, the NCLEX-RN Test Plan should be referred to [10] and the definition and goals for each activity category should be presented more clearly. An opinion was expressed that the 8 activity categories should be arranged systematically in a sequence. Furthermore, a specialist society needs to be established to collect opinions and reach a consensus regarding clinical and community nursing practice. Additionally, the 481 knowledge statements have different levels of specificity for each group. Some of the knowledge statements are not linked to any major subject, while others are linked to 2 or more major subjects. Therefore, the process of collecting opinions and reaching a consensus regarding knowledge statements should be modified and take precedence over other procedures. Because the competency of new nursing graduates is directly related to the quality of nursing and patient safety [11,12], it is necessary to prepare a guideline on item-making that can redirect the aim of the examination to a knowledge-verification model to evaluate examinees’ competency.

### Limitations

Given the basic research nature of this study, which investigated the opinions of experts on the job-based integrated national examination model and a guideline on item-making for new nursing graduates, there is a possibility that it lacks objective justification.

### Generalizability

The problems and directions for improvement proposed in this study relate to the KNLE. The study results are based on the cultural and situational background of the Republic of Korea. Hence, the results of this study have limited applicability to nursing licensing examinations of other countries or licensing examinations for other job groups.

### Suggestions for further studies

Future studies should increase the level of objective legitimacy achieved in this study. For this, a task force team composed of nursing education and practice experts should be formed, and a consensus should be reached through sufficient discussion and careful collection of experts’ opinions.

### Conclusion

The study results suggest that the distribution of items in the following 4 activity categories should be maintained: management of care and improvement of professionalism, basic care, physiological integrity and maintenance, and health promotion and maintenance. However, the number of items of the management of potential risk and the psychosocial integrity and maintenance area should be reduced. The number of items in pharmacological and parenteral therapies, and safety and infection control should be increased.

## Figures and Tables

**Table 1.** Participating societies and associations

Study subjects	Opinion-survey content	No. of executives (no. of audit committee members)
Academic society		
Korean Academic Society of Nursing Administration	Nursing management	11 (2)
Korean Academic Society of Fundamentals of Nursing	Fundamentals of nursing	10 (2)
Korean Academic Society of Child Health Nursing	Children’s health nursing	12 (2)
Korean Society of Women Health Nursing	Women’s health nursing	12 (2)
Korean Society of Adult Nursing	Adult nursing	19 (2)
Korean Academic Society of Psychiatric and Mental Health Nursing	Psychiatric and mental health nursing	19 (8)
Korean Academic Society of Community Health Nursing	Community health nursing	14 (2)
Association		
Korean Society of Nursing Science	1st and 2nd opinion surveys	13 (2)
Korean Association of College of Nursing	1st and 2nd opinion surveys	15
Korean Deans Association of Nursing College	1st and 2nd opinion surveys	17 (2)
Hospital Nurses Association	1st and 2nd opinion surveys	14

**Table 2.** Survey results from 7 academic societies on the number of items in each nursing activity category

Activity category	Activity statements	Knowledge statements	Survey	NM	FN	WH	AN	CH	PMH	CHN	Total after 2nd survey
Management of care and improvement of professionalism	22	79	1st	29					2		41
			2nd	30				1	2	8	
Safety and infection control	3	25	1st	4	6		2		2		16
			2nd	3	6		2	1	2	2	
Management of potential risk	28	93	1st		2		10		2		31
			2nd		2	13	10	2	2	2	
Basic care	19	60	1st		12		15				37
			2nd		12		15	8	1	1	
Physiological integrity and maintenance	36	130	1st		6		39		2		63
			2nd		6	8	39	7	2	1	
Pharmacological and parenteral therapies	7	14	1st		4		2		2		11
			2nd		4		2	2	2	1	
Psychosocial integrity and maintenance	9	43	1st				2		22		41
			2nd			1	2	14	22	2	
Health promotion and maintenance	10	37	1st						2		33
			2nd			13			2	18	
Total	134	481		33 (2)^a)^	30	35	70	35	35	35	273 (2)^b)^

NM, nursing management; FN, fundamental nursing; WH, women’s health nursing; AN, adult nursing; CH, children’s health nursing; PMH, psychiatric and mental health nursing; CHN, community health nursing.

a) Of the 35 test items in nursing management, 2 were about nursing history (world nursing history and Korean nursing history) and were not included here.

b) Twenty items on the medical law were excluded.

**Table 3.** Opinions of experts from the 4 expert associations on the item model for the KNLE (units: number of comments)

Activity category	Activity statements (n=134)									Knowledge statements (n=481)								
	Activity statements	NM	FN	WH	AN	CH	PMH	CHN	Total	Knowledge statements	NM	FN	WH	AN	CH	PMH	CHN	Total
I. Management of care and improvement of professionalism	22	20	-	3	-	3	8	17	51	79	58	-	3	-	4	9	53	127
II. Safety and infection control	3	3	2	-	1	1	3	2	12	25	17	16	-	2	2	12	13	62
III. Management of potential risk	28	-	2	17	10	13	8	5	55	93	-	3	48	20	48	13	6	138
VI. Basic care	19	-	11	-	14	12	3	-	40	60	-	42	-	32	42	8	-	124
V. Physiological integrity and maintenance	36	-	4	4	32	25	2	2	69	130	-	16	15	79	85	4	9	208
VI. Pharmacological and parenteral therapies	7	-	5	1	1	2	3	2	14	14	-	10	1	1	5	3	8	28
VII. Psychosocial integrity and maintenance	9	-	-	2	1	4	9	2	18	43	-	-	2	4	9	40	2	57
VIII. Health promotion and maintenance	10	-	-	2	-	4	2	9	17	37	-	-	3	-	10	4	36	53
Total	134	23	24	29	59	64	38	39	276	481	75	87	72	138	205	93	127	797

NM, nursing management; FN, fundamental nursing; WH, women’s health nursing; AN, adult nursing; CH, children’s health nursing; PMH, psychiatric and mental health nursing; CHN, community health nursing.

**Table 4.** A revised guideline on item-making based on the model for the Korean Nursing Licensing Examination to expresses the item distribution (item model) based on each activity category

Activity category	NCLEX-RN [9] (2019)	Knowledge statements from item model	Tak et al. [9] (2019)	A guideline on item-making based on the opinion survey of the academic societies	Revised guideline on item-making
Management of care and improvement of professionalism	(17–23, 20)	79 (16.4)	50 (18.2)	41 (15.0)	50 (18.2)
Safety and infection control	(9–15, 12)	25 (5.2)	27 (9.8)	16 (5.9)	33 (12.0)
Management of potential risk	(9–15, 12)	93 (19.3)	44 (16.0)	31 (11.4)	40 (14.5)
Basic care	(6–12, 9)	60 (12.5)	27 (9.8)	37 (13.6)	28 (10.2)
Physiological integrity and maintenance	(11–17, 14)	130 (27.0)	55 (20.0)	63 (23.1)	47 (17.1)
Pharmacological and parenteral therapies	(12–18, 15)	14 (2.9)	27 (9.8)	11 (4.0)	33 (12.0)
Psychosocial integrity and maintenance	(6–12, 9)	43 (8.9)	25 (9.1)	41 (15.0)	24 (8.7)
Health promotion and maintenance	(6–12, 9)	37 (7.7)	20 (7.3)	33 (12.1)	20 (7.3)
Total	-100	481 (100.0)	275 (100.0)	273^a)^ (100.0)	275^b)^ (100.0)

Values are presented as (%) or number (%). The NCLEX-RN exam is managed according to the candidate’s competency level, so the distribution of the number of items for each section may vary by up to ±3% depending on the test taker’s time. Further information is available at: https://www.ncsbn.org/2019_RN_TestPlan-English.pdf NCLEX-RN, National Council License Examination for Registered Nurses.

a) Of the 275 test items, 2 were on nursing history (world and Korean nursing history) and were not included here.

b) Of the 275 test items, 20 test items related to medical law were excluded.

## Supplementary materials

Supplementary files are available from Harvard Dataverse: https://doi.org/10.7910/DVN/S4CZRO

Supplement 1. Job-based integrated item model for the Korean Nursing Licensing Examination (in Korean).

Supplement 2. Source report for this article.

Supplement 3. Survey questionnaires sent to 7 academic societies on the number of items in each nursing activity category of the Korean Nursing Licensing Examination.

Supplement 4. Audio recording of the abstract.
