# Supplementary material for: Suggestion for item allocation to 8 nursing activity categories of the Korean Nursing Licensing Examination: a survey-based descriptive study
Source: J Educ Eval Health Prof. 2023 Jun 12;20:18. doi: 10.3352/jeehp.2023.20.18 (PMC10352010; doi:10.3352/jeehp.2023.20.18)
Supplement: Supplementary file 2 — Supplement 1. Job-based integrated item model for the Korean Nursing Licensing Examination (in Korean). [file jeehp-20-18-suppl1.pdf]

간호사 국가시험 출제모형 제시

간호사 국가시험 출제기준(standard of test construction)은 시험문제를 출제하는 기준으로, 시험과목 및 대항목, 분야(또는 영역), 시험문제수, 배점 등으로 구성된다(한국보건의료인 국가시험원 홈페이지, 2020). 출제기준의 근거가 되는 표준으로 간호사 국가시험 출제모형(model of test construction)을 제시하기 위하여 국내외 국가시험 관련 문헌을 고찰하였다. 문헌고찰 결과, <표 Ⅳ-1>와 같이 신규간호사 직무에 기반한 출제기준 개발을 위해 단계별로 순차적으로 2012년에 개발된 8개 신규간호사 직무영역, 2018년 8개 직무영역과 연계하여 개발된 134개 직무항목, 7개 국가시험 교과목 학습목표를 통합한 표준학습목표, 2019년 직무영 역, 직무항목 및 통합형 표준학습목표와 연계하여 개발된 481개 범주의 지식항목 등의 주개념 으로 구성된 출제모형을 확인하였다. 문헌고찰을 통해서 확인된 최신의 신규간호사 직무에 기 반한 통합형 간호사 국가시험 출제모형을 제시하기 위해서는 출제모형을 구성하는 주개념으로 2018년에 개발된 134개의 신규간호사 직무항목에 대한 타당성 및 적절성 검증이 우선되어야 할 필요성이 제기되어 신규간호사 직무항목에 대한 타당성과 적절성 평가를 실시하였다.

가. 출제모형의 간호사 직무항목에 대한 타당성과 적절성 평가

최신의 직무에 기반한 통합형 간호사 국가시험 출제모형을 제시하기 위해, 우선적으로 2018년에 개발된 134개 신규간호사 직무항목(Activity Statement)에 대한 타당성과 적절성 평가를 하였다. 임상 및 지역사회 간호 실무현장의 간호사 264명을 대상으로 설문조사를 실시하였고, 이 중 260부를 자료분석에 활용하였다.

1) 인구사회학적 특성

대상자의 인구사회학적 특성은 다음과 같다 <표 Ⅳ-2>. 직위별로는 일반간호사가 75.38%, 주임 및 책임간호사 8.08%, 수간호사 5.00%, 보건교사 4.23%, 간호직 공무원 3.46%, 기타 3.08% 순으로 나타났다. 기관 소재지별로는 수도권 34.61%, 충청권 30.77%, 강원권 11.54%, 전라권 11.54%, 경상권 11.54% 순이었다. 기관별로는 상급종합병원이 49.62%로 가장 많았고, 종합병원 36.92%, 보건소 6.92%, 학교(보건교사) 3.46% 순으로 나타났다. 간호사로 근무한 기간의 경우 7년 이상이 38.85%로 가장 높게 나타났고, 3~5년 미만이 21.92%, 1~3년 미만이 19.23%, 5~7년 미만이 13.46% 순으로 나타났으며, 현 직장 근무 기간의 경우 7년 이상이 33.46%, 1~3년 미만이 24.62%, 3~5년 미만이 19.62%, 5~7년 미만이 11.54% 순이었으며, 1년 미만이
10.76%로 가장 낮게 나타났다. 근무부서별로는 중환자실이 13.85%로 가장 높았고, 소아청소년과 병동 11.15%, 외과계 병동 11.15%, 수술실/마취회복실 10.77%, 응급

실 10.00%, 정신과 병동 9.23%, 내과계 병동 9.23%, 분만실, 산부인과 병동 8.08%, 보건소 6.92% 순으로 나타났다.

<표 Ⅳ-2> 대상자의 인구사회학적 특성

(N=260)

구분	변수	빈도 (n)	백분율 (%)
직위	일반간호사	196	75.38
	주임, 책임간호사	21	8.08
	수간호사	13	5.00
	간호관리자	2	0.77
	보건교사	11	4.23
	간호직 공무원	9	3.46
	기타	8	3.08
기관 소재지	수도권	90	34.61
	강원권	30	11.54
	충청권	80	30.77
	전라권	30	11.54
	경상권	30	11.54
기관	상급종합병원	129	49.62
	종합병원	96	36.92
	병·의원	7	2.69
	학교(보건교사)	9	3.46
	보건소	18	6.92
	기타	1	0.39
간호사 근무기간	1년 미만	17	6.54
	1 ~ 3년 미만	50	19.23
	3 ~ 5년 미만	57	21.92
	5 ~ 7년 미만	35	13.46
	7년 이상	101	38.85
현 직장 근무기간	1년 미만	28	10.76
	1 ~ 3년 미만	64	24.62
	3 ~ 5년 미만	51	19.62
	5 ~ 7년 미만	30	11.54
	7년 이상	87	33.46
근무부서	내과계 병동	24	9.23
	외과계 병동	29	11.15
	중환자실	36	13.85
	소아청소년과 병동	29	11.15
	분만실, 산부인과병동	21	8.08
	신생아실	12	4.62
	응급실	26	10.00
	정신과병동	24	9.23
	수술실/마취회복실	28	10.77
	학교	10	3.85
	보건소	18	6.92
	기타	3	1.15

2) 8개 직무영역별 신규간호사 직무항목의 중요도와 수행도

가) 간호관리와 전문성 향상

간호관리와 전문성 향상 영역의 직무에 대한 신규간호사 직무 중요도와 수행도는 다음 과 같다<표 Ⅳ-3>.

신규간호사 직무 중요도 순으로 살펴본 결과,「6. 장비를 적절하고 안전하게 사용」이 4.67±0.56,「 법적 직무 범위 내에서 간호 수행 」이 4.66±0.57, 「 11. 대상자의 개인정보 및 사생활 보호 」가 4.66±0.57,「 12. 대상자에게 치료 및 절차에 따라 적절한 설명을 하 고 동의를 획득하였는지 확인 」이 4.58±0.60, 「 4. 지침에 따라 간호기록 」이 4.51±0.66, 「 1. 인수인계 시행 」이 4.39±0.80, 「 13. 환자의 권리와 책임에 관하여 대상자에게 교육 제공 」이 4.38±0.74,「 14. 억제대 사용 시 법적 및 윤리적 간호 」가 4.35±0.75, 「 5. 입 원, 전동, 퇴원 」이 4.34±0.81,「 10. 간호전문직 윤리 준수와 역할 」이 4.34±0.78. 「 3. 기록 시 표준화된 약어사용 」이 4.28±0.77 순이었다.

신규 간호사 직무 수행도 순으로 살펴본 결과, 「 11. 대상자의 개인정보 및 사생활 보호 」가 4.19±0.84, 「 13. 환자의 권리와 책임에 관하여 대상자에게 교육 제공 」이 3.88±0.93, 「 12. 대상자에게 치료 및 절차에 따라 적절한 설명을 하고 동의를 획득하였는 지 확인 」이 3.88±0.91,「 14. 억제대 사용 시 법적 및 윤리적 간호 」가 3.82±0.88,「 10. 간호전문직 윤리 준수와 역할 」 3.81±0.92, 「 6. 장비를 적절하고 안전하게 사용 」 이 3.80±3.34,「 4. 지침에 따라 간호기록 」이 3.76±0.92, 「 2. 기록 시 표준화된 약어사용 」이 3.76±0.92, 「 2. 법적 직무 범위 내에서 간호수행 」이 3.68±0.90, 「 5. 입원, 전동, 퇴원 」이 3.55±0.95,「 7. 간호단위 물품교환체계에 따른 물품관리 」가 3.39±1.01,「 15. 일 차의료기반의 보건간호 」가 3.38±1.01, 「 1. 인수인계시행 」이 3.25±0.99, 「 22. 전문직간 협업 」이 3.07±0.99,「 16. 지역사회간호사업의 법적 기준 및 지침에 따른 활동 참여 」가 3.05±1.09 순이었다.

<표 Ⅳ-3> 간호관리와 전문성 향상

번호	항목	중요도 (Mean±SD)	수행도 (Mean±SD)
1	인수인계시행	4.39±0.80	3.25±0.99
2	법적 직무범위 내에서 간호 수행	4.66±0.57	3.68±0.90
3	기록시 표준화된 약어사용	4.28±0.77	3.70±0.89

4	지침에 따라 간호기록	4.51±0.66	3.76±0.92
5	입원, 전동, 퇴원	4.34±0.81	3.55±0.95
6	장비를 적절하고 안전하게 사용	4.67±0.56	3.80±3.34

번호	항목	중요도 (Mean±SD)	수행도 (Mean±SD)
7	간호단위 물품교환체계에 따른 물품관리	3.91±0.95	3.39±1.01
8	질 향상(QI) 활동에 참여	3.16±1.08	2.64±0.96
9	간호사업 평가 관련 업무수행(도구개발, 자료조사, 분석, 비교 및 사업개선)	2.81±1.15	2.42±0.98
10	간호전문직 윤리 준수와 역할	4.34±0.78	3.81±0.92
11	대상자의 개인정보 및 사생활 보호	4.66±0.56	4.19±0.84
12	대상자에게 치료 및 절차에 따라 적절한 설명을 하고 동의를 획득하였는지 확인 4.58±0.60		3.88±0.91
13	환자의 권리와 책임에 관하여 대상자에게 교육 제공	4.38±0.74	3.88±0.93
14	억제대 사용 시 법적 및 윤리적 간호	4.35±0.75	3.82±0.88
15	일차의료기반의 보건간호	3.78±1.08	3.38±1.01
16	지역사회 간호사업의 법적 기준 및 지침에 따른 활동 참여 3.05±1.09		3.35±1.18
17	사례관리활동 참여	3.12±1.05	2.78±0.95
18	업무를 조직화하여 일을 효율적으로 관리	3.70±1.07	2.88±0.96
19	환자분류체계 관련 정보수집 및 활용	3.54±1.02	3.00±0.94
20	간호대상에 적합한 다양한 자원 및 매체선택	3.40±1.00	2.86±0.91
21	취약가족에게 필요한 지역사회 자원활용	2.97±1.08	2.58±0.98
22	전문직간 협업	3.90±0.90	3.07±0.99

※문항별 결측치 제외

간호관리와 전문성 향상의 직무 항목에 대해 신규 간호사의 직무 중요도 및 수행도 를 y축은 중요도, x축을 수행도로 설정하고 3점을 기준선으로 IPA 분석한 결과는 [그 림 Ⅳ-2]과 같다. 우선, 1사분위에 위치하고 있는「 8. 질 향상(QI) 활동에 참여 」,「 17. 사례관리활동 참여 」,「 18. 업무를 조직화하여 일을 효율적으로 관리 」,「 20. 간호대상 에 적합한 다양한 자원 및 매체선택 」항목은 중요도는 3점 이상이나 수행도가 3점 이 하인 직무로 나타났다. 3사분위에 위치하고 있는「 9. 간호사업 평가 관련 업무수행(도 구개발, 자료조사, 분석, 비교 및 사업개선)」,「21. 취약가족에게 필요한 지역사회 자 원활용 」, 은 중요도와 수행도 모두 3점 이하로 나타났으며 그 이외의 직무들은 모두 2사분위에 위치하고 있어 신규 간호사의 직무 중요도와 수행도 모두 3점 이상인 것으 로 나타났다.

나) 안전과 감염관리

안전과 감염관리 영역의 직무에 대한 신규간호사 직무 중요도와 수행도는 다음과 같다<표 Ⅳ-4>.

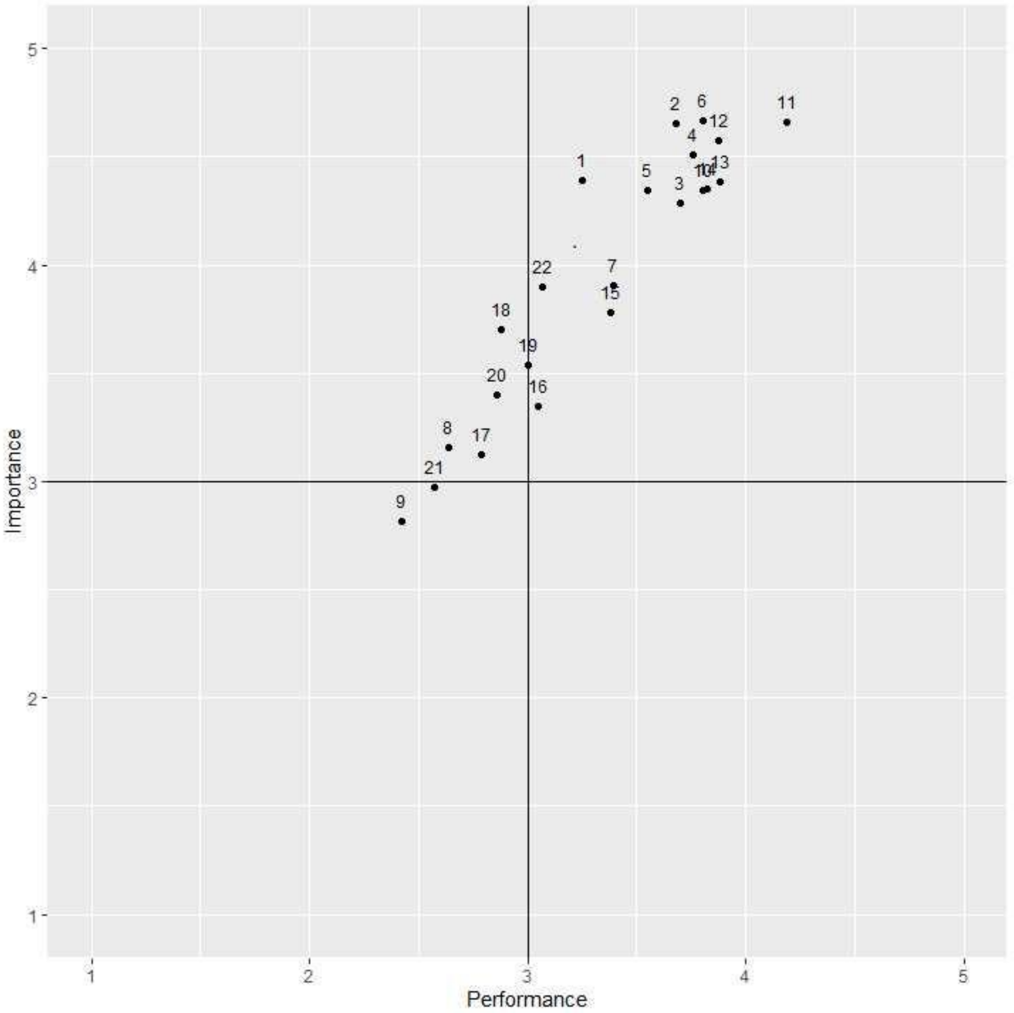

[그림 Ⅳ-2] 간호관리와 전문성 향상 직무의 IPA

신규 간호사 직무 중요도 순으로 살펴본 결과,「 24. 감염관리 」가 4.76±0.51「 23. 안 전한 환경 제공 」이 4.67±0.58,「 25. 위험물질과 유해물질 관리 」가 4.59±0.69 순이 었다.

신규 간호사 직무 수행도 순으로 살펴본 결과,「 23. 안전한 환경 제공 」이 4.02±0.81, 「 24. 감염관리 」가 4.00±0.88,「 25. 위험물질과 유해물질 관리 」가 3.71±0.96 순으로 나타났다.

<표 Ⅳ-4> 안전과 감염관리

번호	항목	중요도 (Mean±SD)	수행도 (Mean±SD)
23	안전한 환경 제공	4.67±0.58	4.02±0.81
24	감염관리	4.76±0.51	4.00±0.88
25	위험물질과 유해물질 관리	4.59±0.69	3.71±0.96

※문항별 결측치 제외

안전과 감염관리 직무 항목에 대해 신규 간호사의 직무 중요도 및 수행도를 y축은 중요도, x축을 수행도로 설정하고 3점을 기준선으로 IPA 분석한 결과는 [그림 Ⅳ-3]과 같다. 「23. 안전한 환경 제공」, 「24. 감염관리」, 「25. 위험물질과 유해물질 관리」 모 두 2사분위에 위치하고 있어 신규 간호사의 직무 중요도와 수행도 모두 3점 이상인 것 으로 나타났다.

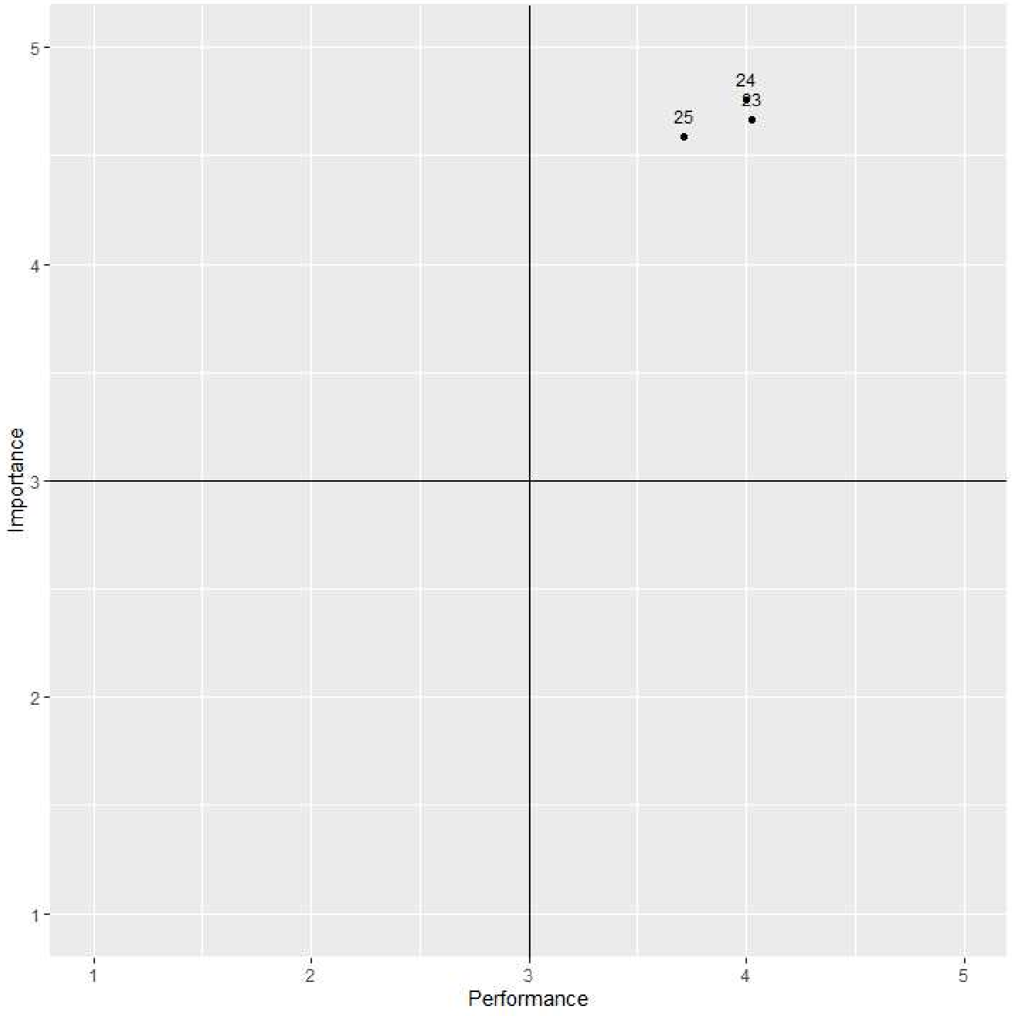

[그림 Ⅳ-3] 안전과 감염관리 직무의 IPA

다) 위험요인 사정

위험요인 사정 영역의 직무에 대한 신규간호사 직무 중요도와 수행도는 다음과 같다<표 Ⅳ-5>.

신규 간호사 직무 중요도 순으로 살펴본 결과, 「 28. 활력징후 사정 」이 4.83±0.43,

「44. 심폐소생술 간호」 가 4.81±0.47, 「45. 응급간호」 가 4.76±0.50, 「38. 건강력
사정 」 이 4.53±0.68,「 47. 수술간호 」 가 4.51±0.72,「 39. 신체검진 수행 및 결과 해
석」 이 4.47±0.70, 「27. 대상자의 건강문제에 대해 우선순위 결정」 이 4.46±0.74,
「46. 진단검사 간호」 가 4.42±0.75,「26. 간호계획, 진료지침 수행」 이 4.35±0.76,
「 36. 노인간호 」가 4.32±0.81 순이었다.

신규 간호사 직무 수행도 순으로 살펴본 결과,「28. 활력징후 사정」이 4.47±0.75,
「38. 건강력 사정」 이 3.81±0.86, 「26. 간호계획, 진료지침 수행」 이 3.69±0.90,
「39. 신체검진 수행 및 결과 해석」 이 3.64±0.89, 「36. 노인간호」 가 3.63±0.91,
「 34. 청소년 간호 」가 3.54±0.93,「 35. 폐경기 여성 간호 」가 3.52±0.91,
「 33. 학령 기 간호 」가 3.51±0.95,「 47. 수술간호 」가 3.50±0.94 순이었다.

<표 Ⅳ-5> 위험요인 사정

번호	항목	중요도 (Mean±SD)	수행도 (Mean±SD)
26	간호계획, 진료지침 수행	4.35±0.76	3.69±0.90
27	대상자의 건강문제에 대해 우선순위 결정	4.46±0.74	3.47±0.95
28	활력징후 사정	4.83±0.43	4.47±0.75
29	신생아 간호	4.24±0.92	3.40±1.01
30	영아 간호	4.19±0.94	3.39±1.02
31	유아 간호	4.20±0.94	3.42±1.01
32	학령전기 간호	4.17±0.95	3.49±0.95
33	학령기 간호	4.17±0.95	3.51±0.95
34	청소년 간호	4.17±0.94	3.54±0.93
35	폐경기 여성 간호	4.17±0.92	3.52±0.91
36	노인간호	4.32±0.81	3.63±0.91
37	성 건강 간호	4.10±0.93	3.48±0.94

38	건강력 사정	4.53±0.68	3.81±0.86
39	신체검진 수행 및 결과 해석	4.47±0.70	3.64±0.89
40	생식기 건강사정	4.13±0.88	3.44±0.96
41	태아건강사정 및 간호	4.10±1.02	3.25±1.03
42	신생아 건강사정 및 간호	4.12±1.00	3.29±1.03
43	고위험신생아 건강사정 및 간호	4.18±1.01	3.1±1.07
44	심폐소생술 간호	4.81±0.47	3.34±1.09
45	응급간호	4.76±0.50	3.24±1.02
46	진단검사 간호	4.42±0.75	3.33±0.95
47	수술간호	4.51±0.72	3.50±0.94
48	산전간호와 교육	4.09±0.99	3.34±0.99
49	분만 중 간호와 교육	4.11±0.98	3.27±1.01

번호	항목	중요도 (Mean±SD)	수행도 (Mean±SD)
50	산후관리와 교육	4.11±0.98	3.29±0.99
51	고위험 임부간호	4.15±1.00	3.16±1.02
52	고위험 산부간호	4.14±1.00	3.15±1.01
53	고위험 산모간호	4.14±1.00	3.15±1.01

※문항별 결측치 제외

위험요인 사정 직무 항목에 대해 신규 간호사의 직무 중요도 및 수행도를 y축은 중요도, x축을 수행도로 설정하고 3점을 기준선으로 IPA 분석한 결과는 [그림 Ⅳ-4]과 같다. 위험요인 사정 영역의 28개 직무 모두 2사분위에 위치하고 있어 신규 간호사의 직무 중요도와 수행도 모두 3점 이상인 것으로 나타났다.

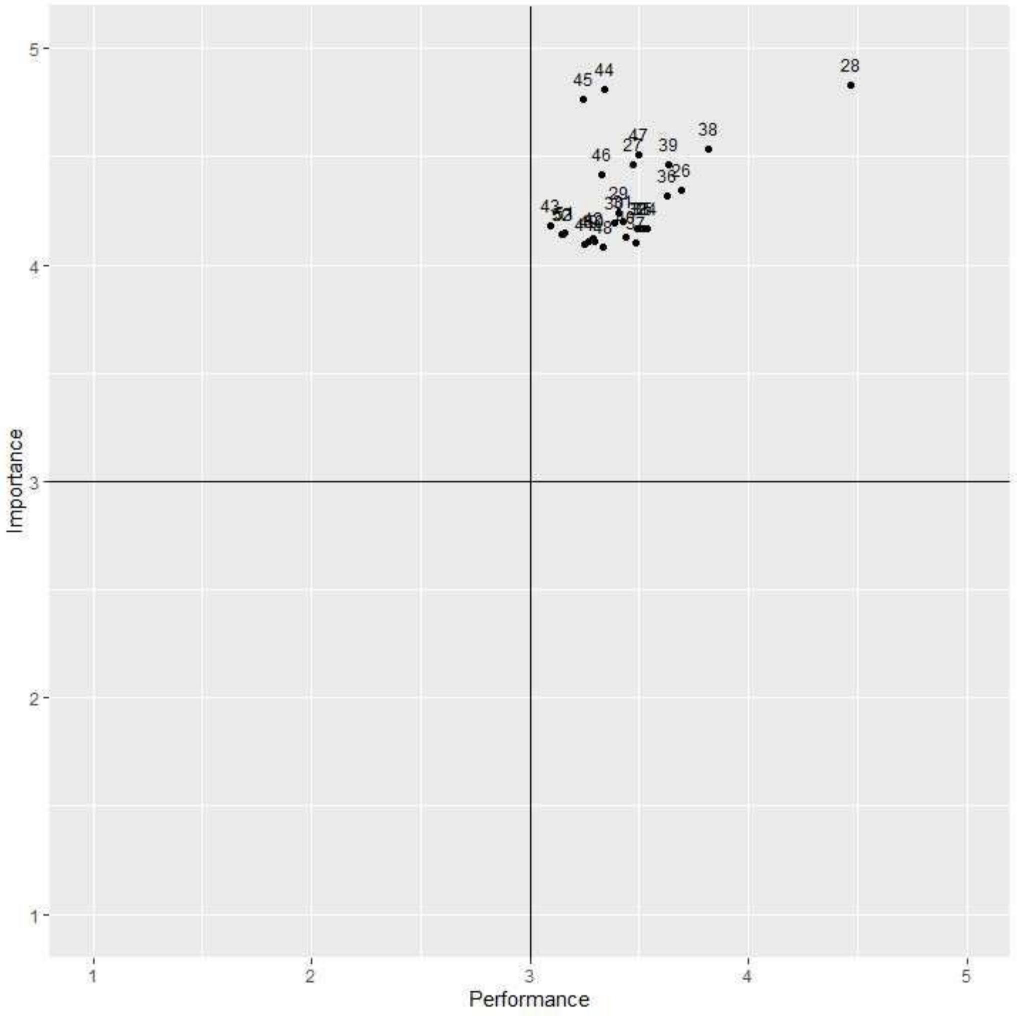

[그림 Ⅳ-4] 위험요인 사정 직무의 IPA

라) 기본간호

기본간호 영역의 직무에 대한 신규간호사 직무 중요도와 수행도는 다음과 같다<표 Ⅳ-6>.

신규 간호사 직무 중요도 순으로 살펴본 결과, 「57. 섭취량 및 배설량 사정과 간호」가 4.60±0.60, 「54. 체온유지간호」가 4.49±0.66, 「64. 기관절개부 관리」가 4.38±0.81, 「61. 배뇨장애 대상자 사정 및 간호」가 4.38±0.74, 「67. 피부통합성 사정 및 간호」가 4.35±0.77, 「62. 투석대상자 관리」가 4.34±0.81, 「56. 개인위생간호」가4.34±0.76, 「60. 섭취장애 대상자 사정 및 간호」가 4.32±0.77, 「65. 배변관리」가 4.32±0.76, 「59. 질환별 영양문제의 사정 및 관리」가 4.31±0.80 순이었다. 신규 간호사 직무 수행도 순으로 살펴본 결과, 「54. 체온유지간호」가 4.19±0.81, 「56. 개인위생간호」가 4.17±0.78, 「57. 섭취량 및 배설량 사정과 간호」가 4.16±0.76, 「66. 수면과 휴식 간호」가 4.02±0.81, 「58. 영양사정 및 관리」가 3.95±0.85, 「68. 이동간호」가 3.91±0.91, 「65. 배변관리」가 3.90±0.87, 「67. 피부 통합성 사정 및 간호」가 3.85±0.91, 「55. 세척(irrigation)(방광, 귀, 눈) 수행」이 3.81±0.95, 「61. 배뇨장애 대상자 사정 및 간호」가 3.80±0.86 순이었다.

<표 Ⅳ-6> 기본간호

번호	항목	중요도 (Mean±SD)	수행도 (Mean±SD)
54	체온유지간호	4.49±0.66	4.19±0.81
55	세척(irrigation)(방광, 귀, 눈) 수행	4.19±0.86	3.81±0.95
56	개인위생간호	4.34±0.76	4.17±0.78
57	섭취량 및 배설량 사정과 간호	4.60±0.60	4.16±0.76
58	영양사정 및 관리	4.29±0.80	3.95±0.85
59	질환별 영양문제의 사정 및 관리	4.31±0.80	3.66±0.88
60	섭취장애 대상자 사정 및 간호	4.32±0.77	3.76±0.83
61	배뇨장애 대상자 사정 및 간호	4.38±0.74	3.80±0.86
62	투석대상자 관리	4.34±0.81	3.34±0.97
63	요루 및 장루관리	4.28±0.84	3.27±1.00
64	기관절개부 관리	4.38±0.81	3.34±1.05

65	배변관리	4.32±0.76	3.90±0.87
66	수면과 휴식 간호	4.19±0.89	4.02±0.81
67	피부통합성 사정 및 간호	4.35±0.77	3.85±0.91

번호	항목	중요도 (Mean±SD)	수행도 (Mean±SD)
68	이동간호	4.08±0.96	3.91±0.91
69	활동과 자기돌봄 장애 사정 및 간호	4.02±0.96	3.76±0.91
70	외과적 장치 관리	4.25±0.85	3.45±0.97
71	척추손상 및 신경질환 대상자의 간호	4.25±0.85	3.39±0.99
72	관절대치술 환자 간호	4.16±0.93	3.44±0.97

※문항별 결측치 제외

기본간호 직무 항목에 대해 신규 간호사의 직무 중요도 및 수행도를 y축은 중요도, x축을 수행도로 설정하고 3점을 기준선으로 IPA 분석한 결과는 [그림 Ⅳ-5]와 같다. 기본간호 영역의 19개 직무 모두 2사분위에 위치하고 있어 신규 간호사의 직무 중요도와 수행도 모두 3점 이상인 것으로 나타났다.

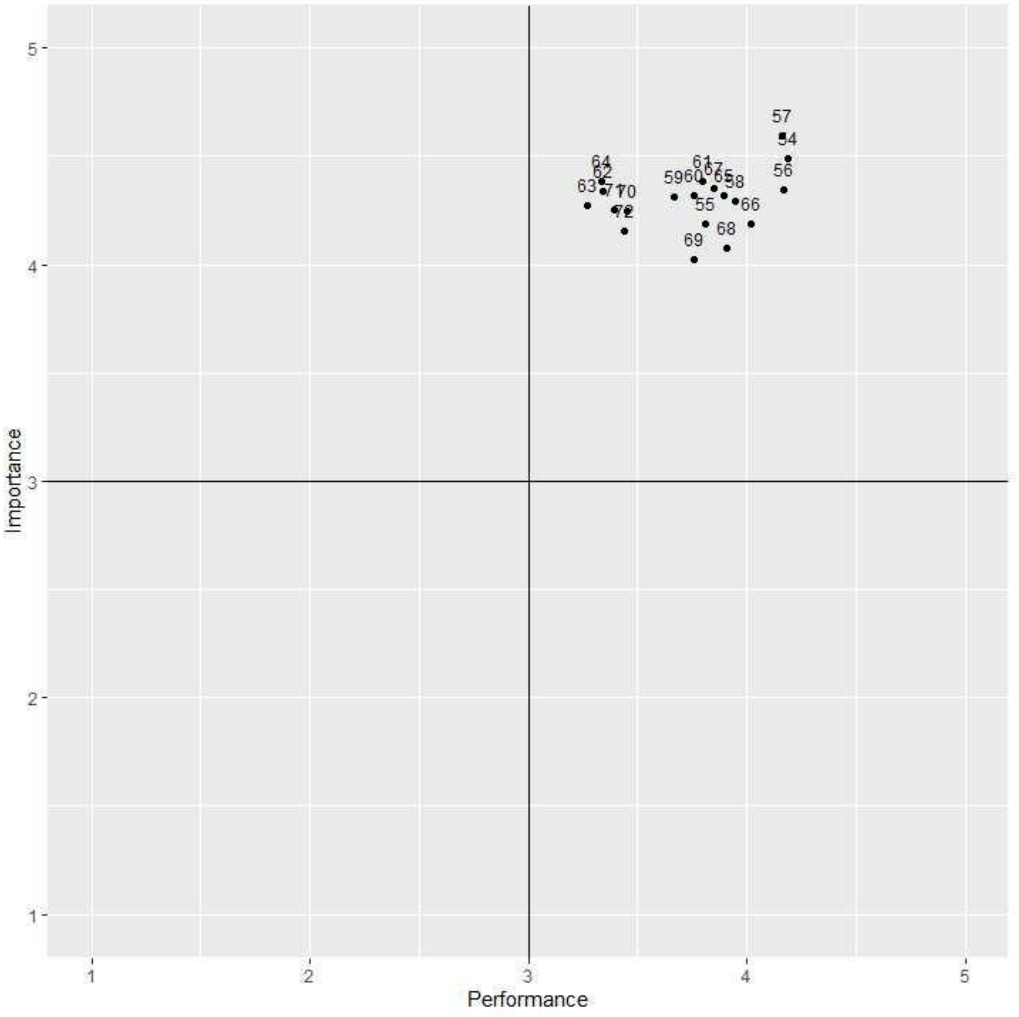

[그림 Ⅳ-5] 기본간호 직무의 IPA

마) 생리적 통합유지

생리적 통합유지 영역의 직무에 대한 신규간호사 직무 중요도와 수행도는 다음과 같다<표 Ⅳ-7>.

신규 간호사 직무 중요도 순으로 살펴본 결과,「73. 호흡기능장애 대상자 간호」가 4.60±0.62,「 76. 중심정맥관 관리 」가 4.57±0.64,「 74. 호흡증진 중재 」가 4.57±0.61,「 75. 호흡보조장치 관리 」가 4.53±0.67,「 106. 통증간호 」가 4.50±0.68,「104. 배액장치 관리」가 4.48±0.67,「84. 체액 전해질 불균형 사정 및 간호」가 4.47±0.72,「 108. (법정) 감염질환자의 전파예방 및 간호 」가 4.43±0.78,「 98. 두개 내압 상승 환자의 간호 」가 4.40±0.83 순이었다.

신규 간호사 직무 수행도 순으로 살펴본 결과,「 106. 통증간호 」가 3.87±0.85,「 101. 상처간호 수행 및 드레싱교환 」이 3.65±0.96,「 93. 당뇨병 합병증 예방을 위 한 간호」가 3.64±0.90,「104. 배액장치 관리」가 3.63±0.95,「74. 호흡증진 중재」가 3.57±0.90, 「91. 배뇨장애를 가진 대상자 간호」가 3.56±0.91,「103. 피부질환 대상자의 간호중재」가 3.55±0.89,「94. 내분비계 장애 대상자 간호」가 3.53±0.90,「73. 호흡기능장애 대상자 간호」가 3.52±0.86,「92. 당질 대사장애 대상자 간호」가 3.50±0.95 순이었다.

<표 Ⅳ-7> 생리적 통합유지

번호	항목	중요도 (Mean±SD)	수행도 (Mean±SD)
73	호흡기능장애 대상자 간호	4.60±0.62	3.52±0.86
74	호흡증진 중재	4.57±0.61	3.57±0.90
75	호흡보조장치 관리	4.53±0.67	3.32±0.98
76	중심정맥관 관리	4.57±0.64	3.45±0.99
77	태아질식 증상과 징후 사정 및 간호	4.25±0.94	3.11±1.02
78	고위험 신생아 보육기 적용간호	4.05±1.04	3.02±1.08
79	호흡재활관리	4.12±0.89	3.24±0.98
80	심전도 관리	4.38±0.78	3.29±1.02
81	순환 보조장치 관리	4.25±0.86	3.05±1.06
82	동맥관 관리	4.30±0.85	3.07±1.06
83	조직 관류 장애 대상자 사정 및 간호	4.32±0.82	3.26±0.99

84	체액 전해질 불균형 사정 및 간호	4.47±0.72	3.42±0.94

번호	항목	중요도 (Mean±SD)	수행도 (Mean±SD)
85	활동 지속성 장애 대상자 사정 및 간호	4.18±0.89	3.42±0.94
86	정맥 순환증진 장치 간호	4.30±0.78	3.38±1.00
87	혈액 기능장애 대상자 간호	4.36±0.77	3.32±0.95
88	순환 기능장애 대상자 간호	4.38±0.75	3.33±0.96
89	심장 수술 후 간호	4.34±0.84	3.12±1.03
90	소화 기능장애 대상자 간호	4.35±0.76	3.46±0.92
91	배뇨장애를 가진 대상자 간호	4.35±0.76	3.56±0.91
92	당질 대사장애 대상자 간호	4.34±0.76	3.50±0.95
93	당뇨병 합병증 예방을 위한 간호	4.35±0.77	3.64±0.90
94	내분비계 장애 대상자 간호	4.37±0.74	3.53±0.90
95	생식기 질환/생식기 건강문제를 가진 대상자 간호		4.17±0.86
3.49±0.89 96	면역손상 대상자 사정 및 간호	4.32±0.78	3.46±0.91
97	감각기능 장애를 가진 대상자 사정 및 간호	4.22±0.83	3.45±0.93
98	두개내압 상승 환자의 간호	4.4±0.83	3.25±1.00
99	신경계 질환별 간호중재	4.37±0.79	3.32±0.97
100	운동기능 장애 간호중재	4.17±0.89	3.47±0.92
101	상처간호 수행 및 드레싱교환	4.39±0.76	3.65±0.96
102	화상환자 간호중재	4.17±0.93	3.30±0.96
103	피부질환 대상자의 간호중재	4.20±0.83	3.55±0.89
104	배액장치 관리	4.48±0.67	3.63±0.95
105	계통별 신생물 질환 대상자 간호	4.07±0.96	3.31±0.94
106	통증간호	4.50±0.68	3.87±0.85
107	재활간호서비스	3.90±1.04	3.34±0.97
108	(법정) 감염질환자의 전파예방 및 간호	4.43±0.78	3.47±0.97

※문항별 결측치 제외

생리적 통합유지 직무 항목에 대해 신규 간호사의 직무 중요도 및 수행도를 y축은 중요도, x축을 수행도로 설정하고 3점을 기준선으로 IPA 분석한 결과는 [그림 Ⅳ-6]와 같다. 기본간호 영역의 36개 직무 모두 2사분위에 위치하고 있어 신규 간호사의 직무 중요도와 수행도 모두 3점 이상인 것으로 나타났다.

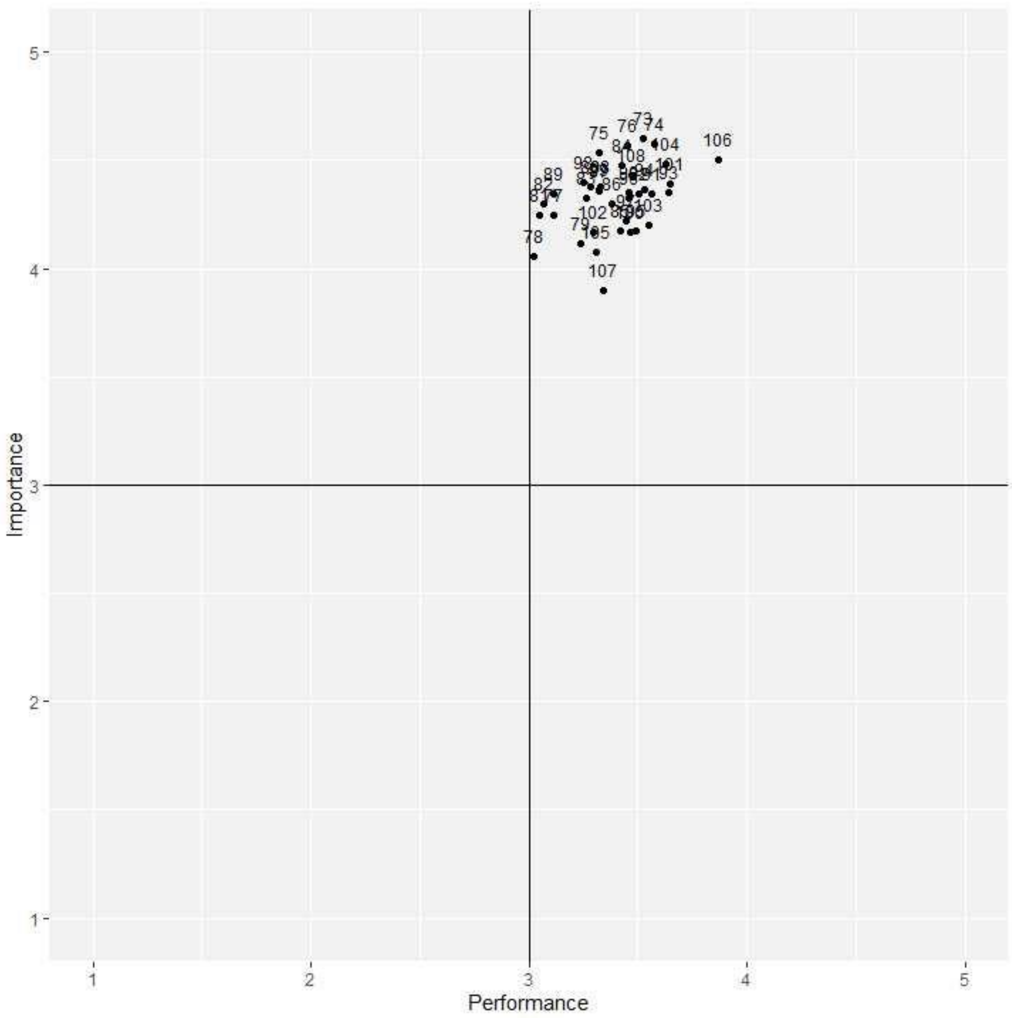

[그림 Ⅳ-6] 생리적 통합유지 직무의 IPA

바) 약물 및 비경구요법

약물 및 비경구요법 영역의 직무에 대한 신규간호사 직무 중요도와 수행도는 다음 과 같다<표 Ⅳ-8>.

신규 간호사 직무 중요도 순으로 살펴본 결과, 「109. 투약의 적절성과 정확성」 이 4.85±0.44, 「 111. 정맥주입 장치 관리 」가 4.85±0.44, 「 110. 약물투여에 필요한 계 산 시행 」가 4.83±0.47, 「 114. 대상자에게 약물에 대해 교육 」이 4.78±0.47, 「 113. 말초정맥관 삽입, 유지, 제거」 가 4.77±0.55, 「115. 고위험 약품관리(마약관리)」 가 4.75±0.54, 「 112. 약품관리 」가 4.73±0.57 순으로 모든 항목에서 4점을 상회하였다. 신규 간호사 직무 수행도 순으로 살펴본 결과, 「109. 투약의 적절성과 정확성」 이 4.11±0.89, 「111. 정맥주입 장치 관리」 가 4.04±0.93, 「110. 약물투여에 필요한 계

산 시행 」이 3.97±0.97,「 114. 대상자에게 약물에 대해 교육 」이 3.99±0.99,「 113. 말초정맥관 삽입, 유지, 제거」가 3.93±1.01,「115. 고위험 약품관리(마약관리)」가 3.76±1.09,「 112. 약품관리 」가 3.97±0.98 순이었다.

<표 Ⅳ-8> 약물 및 비경구요법

번호	항목	중요도 (Mean±SD)	수행도 (Mean±SD)
109	투약의 적절성과 정확성	4.85±0.44	4.11±0.89
110	약물투여에 필요한 계산 시행	4.83±0.47	3.97±0.97
111	정맥주입 장치 관리	4.85±0.44	4.04±0.93
112	약품관리	4.73±0.57	3.97±0.98
113	말초정맥관 삽입, 유지, 제거	4.77±0.55	3.93±1.01
114	대상자에게 약물에 대해 교육	4.78±0.47	3.99±0.99
115	고위험 약품관리(마약관리)	4.75±0.54	3.76±1.09

※문항별 결측치 제외

약물 및 비경구요법 직무 항목에 대해 신규 간호사의 직무 중요도 및 수행도를 y축은 중요도, x축을 수행도로 설정하고 3점을 기준선으로 IPA 분석한 결과는 [그림 Ⅳ
-7]과 같다. 약물 및 비경구요법 영역의 7개 직무 모두 2사분위에 위치하고 있어 신 규 간호사의 직무 중요도와 수행도 모두 3점 이상인 것으로 나타났다.

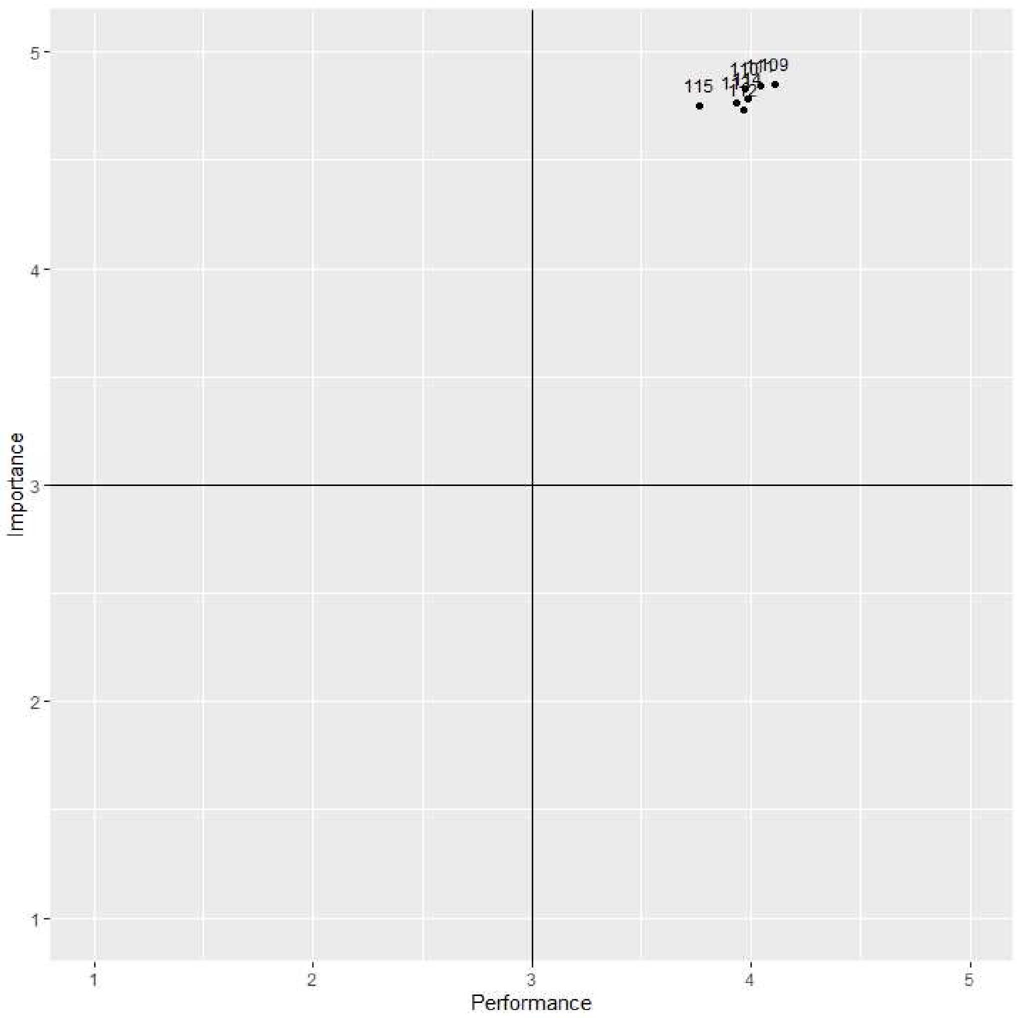

[그림 Ⅳ-7] 약물 및 비경구요법 직무의 IPA

사) 심리사회적 통합 유지

심리사회적 통합유지 영역의 직무에 대한 신규간호사 직무 중요도와 수행도는 다음 과 같다<표 Ⅳ-9>.

신규 간호사 직무 중요도 순으로 살펴본 결과, 「 124. 치료적 의사소통 기법 」 이 4.26±0.87, 「123. 임종간호와 교육」 이 4.13±0.90, 「116. 이상행동 사정 및 간호」가 4.12±0.84, 「118. 학대 또는 방임 대상자를 확인하고 적절하게 중재」 가 4.06±0.91,「117. 폭력의 잠재성 평가 및 예방」이 4.02±0.93,「121. 정신질환자 간호」 가 3.98±0.99,「119. 중독 사정 및 간호」가 3.97±0.96,「122. 아동 정신질환자 간호」 가 3.95±1.01,「120. 정신사회건강문제 간호 및 교육」 이 3.86±1.01 순이었 다.

신규 간호사 직무 수행도 순으로 살펴본 결과,「 124. 치료적 의사소통 기법 」이 3.37±1.00, 「116. 이상행동 사정 및 간호」 가 3.28±0.96, 「123. 임종간호와 교육」이 3.20±1.00, 「 117. 폭력의 잠재성 평가 및 예방 」이 3.16±0.94, 「 119. 중독 사정 및 간호」가 3.14±1.01, 「118. 학대 또는 방임 대상자를 확인하고 적절하게 중재」가 3.12±0.98,「 121. 정신질환자 간호 」가 3.11±0.97,「 120. 정신사회건강문제 간호 및 교육」 이 3.10±0.98, 「122. 아동 정신질환자 간호」가 3.05±1.00 순이었다.

<표 Ⅳ-9> 심리사회적 통합 유지

번호	항목	중요도 (Mean±SD)	수행도 (Mean±SD)
116	이상행동 사정 및 간호	4.12±0.84	3.28±0.96
117	폭력의 잠재성 평가 및 예방	4.02±0.93	3.16±0.94
118	학대 또는 방임 대상자를 확인하고 적절하게 중재	4.06±0.91	3.12±0.98
119	중독 사정 및 간호	3.97±0.96	3.14±1.01
120	정신사회건강문제 간호 및 교육	3.86±1.01	3.10±0.98
121	정신질환자 간호	3.98±0.99	3.11±0.97
122	아동 정신질환자 간호	3.95±1.01	3.05±1.00
123	임종간호와 교육	4.13±0.90	3.20±1.00
124	치료적 의사소통 기법	4.26±0.87	3.37±1.00

※문항별 결측치 제외

심리사회적 통합 유지 직무 항목에 대해 신규 간호사의 직무 중요도 및 수행도를 y축은 중요도, x축을 수행도로 설정하고 3점을 기준선으로 IPA 분석한 결과는 [그림 Ⅳ-8]과 같다. 심리사회적 통합 유지 영역의 9개 직무 모두 2사분위에 위치하고 있어 신규 간호사의 직무 중요도와 수행도 모두 3점 이상인 것으로 나타났다.

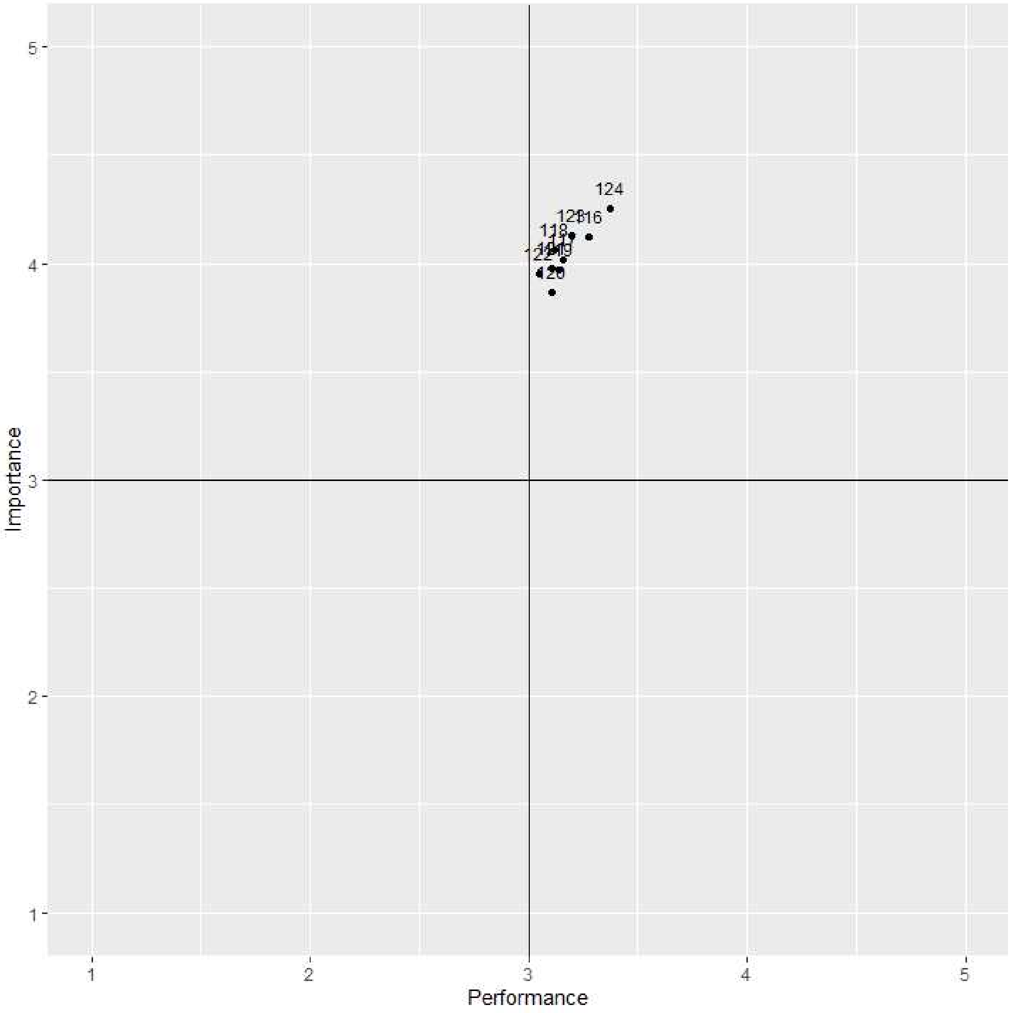

[그림 Ⅳ-8] 심리사회적 통합 유지 직무의 IPA

아) 건강유지 및 증진

건강유지 및 증진 영역의 직무에 대한 신규간호사 직무 중요도와 수행도는 다음과 같다<표 Ⅳ-10>.

신규 간호사 직무 중요도 순으로 살펴본 결과, 「 127. 고위험 건강행위의 예방 및 치료에 대한 정보(금연, 안전한 성 행위, 바늘교환)제공 」 이 3.70±0.97, 「 126. 건강증 진 및 유지관리에 대한 정보(예방접종 등)제공 」 이 3.52±1.01, 「 132. 치료계획을 결 정하기 위해 가족 역동성(family dynamics) (가족구조, 결속, 의사소통, 경계, 대처기 전) 사정 」 이 3.39±1.07, 「 131. 재난간호 」 가 3.35±1.14, 「 125. (지역사회)건강교육
계획 」 이 3.33±1.10, 「 128. 성 건강증진 간호 」 가 3.33±1.04, 「 129. 문화간호 」 가

3.14±1.13,「 130. 산업과 환경 간호 」가 3.14±1.12 순이었다.

신규 간호사 직무 수행도 순으로 살펴본 결과,「 127. 고위험 건강행위의 예방 및 치료에 대한 정보(금연, 안전한 성 행위, 바늘교환)제공 」이 3.28±1.01,「 126. 건강증 진 및 유지관리에 대한 정보(예방접종 등)제공 」이 3.17±1.00,「 134. 건강위험요인 사정과 교육」이 3.11±1.01,「128. 성 건강증진 간호」 가 3.03±0.99로 4항목에서 평균 3점을 상회하셨으나,「 129. 문화간호 」가 2.96±1.05,「 125. (지역사회)건강교육 계획 」이 2.96±1.02,「 132. 치료계획을 결정하기 위해 가족 역동성(family dynamics) (가족구조, 결속, 의사소통, 경계, 대처기전) 사정」이 2.94±1.01,「130. 산업과 환경 간호 」가 2.91±1.06,「 131. 재난간호 」가 2.90±1.10,「 133. 가정환경에 서 대상자를 관리할 수 있는 역량(장비, 지역사회 자원) 평가 2.90±1.02 」순으로 6개 항목에서 평균 3점 미만인 것으로 나타났다.

<표 Ⅳ-10> 건강유지 및 증진

번호	항목	중요도 (Mean±SD)	수행도 (Mean±SD)
125	(지역사회)건강교육 계획	3.33±1.10	2.96±1.02
126	건강증진 및 유지관리에 대한 정보(예방접종 등)제공	3.52±1.01	3.17±1.00
127	고위험 건강행위의 예방 및 치료에 대한 정보 (금연, 안전한 성 행위, 바늘교환)제공	3.70±0.97	3.28±1.01
128	성 건강증진 간호	3.33±1.04	3.03±0.99
129	문화간호	3.14±1.13	2.96±1.05
130	산업과 환경 간호	3.14±1.12	2.91±1.06
131	재난간호	3.35±1.14	2.90±1.10
132	치료계획을 결정하기 위해 가족 역동성(family dynamics) (가족구조, 결속, 의사소통, 경계, 대처기전) 사정	3.39±1.07	2.94±1.01
133	가정환경에서 대상자를 관리할 수 있는 역량 (장비, 지역사회 자원) 평가	3.33±1.07	2.90±1.02
134	건강위험요인 사정과 교육	3.64±1.03	3.11±1.01

※문항별 결측치 제외

건강유지 및 증진 직무 항목에 대해 신규 간호사의 직무 중요도 및 수행도를 y축은 중요도, x축을 수행도로 설정하고 3점을 기준선으로 IPA 분석한 결과는 [그림 Ⅳ-9]과 같다. 우선, 1사분위에 위치하고 있는 「125. (지역사회)건강교육 계획」, 「129. 문

화간호」, 「130. 산업과 환경 간호」, 「131. 재난간호」, 「132. 치료계획을 결정하기 위 해 가족 역동성(family dynamics) (가족구조, 결속, 의사소통, 경계, 대처기전) 사

정 」, 「 133. 가정환경에서 대상자를 관리할 수 있는 역량(장비, 지역사회 자원) 」 항목 의 중요도는 3점 이상이나 수행도가 3점 이하인 직무로 나타났다. 그 이외의 「 126. 건강증진 및 유지관리에 대한 정보(예방접종 등)제공 」, 「 127. 고위험 건강행위의 예방 및 치료에 대한 정보(금연, 안전한 성 행위, 바늘교환)제공」, 「128. 성 건강증진 간

호」, 「134. 건강위험요인 사정과 교육」 항목은 모두 2사분위에 위치하고 있어 신규 간호사의 직무 중요도와 수행도 모두 3점 이상인 것으로 나타났다.

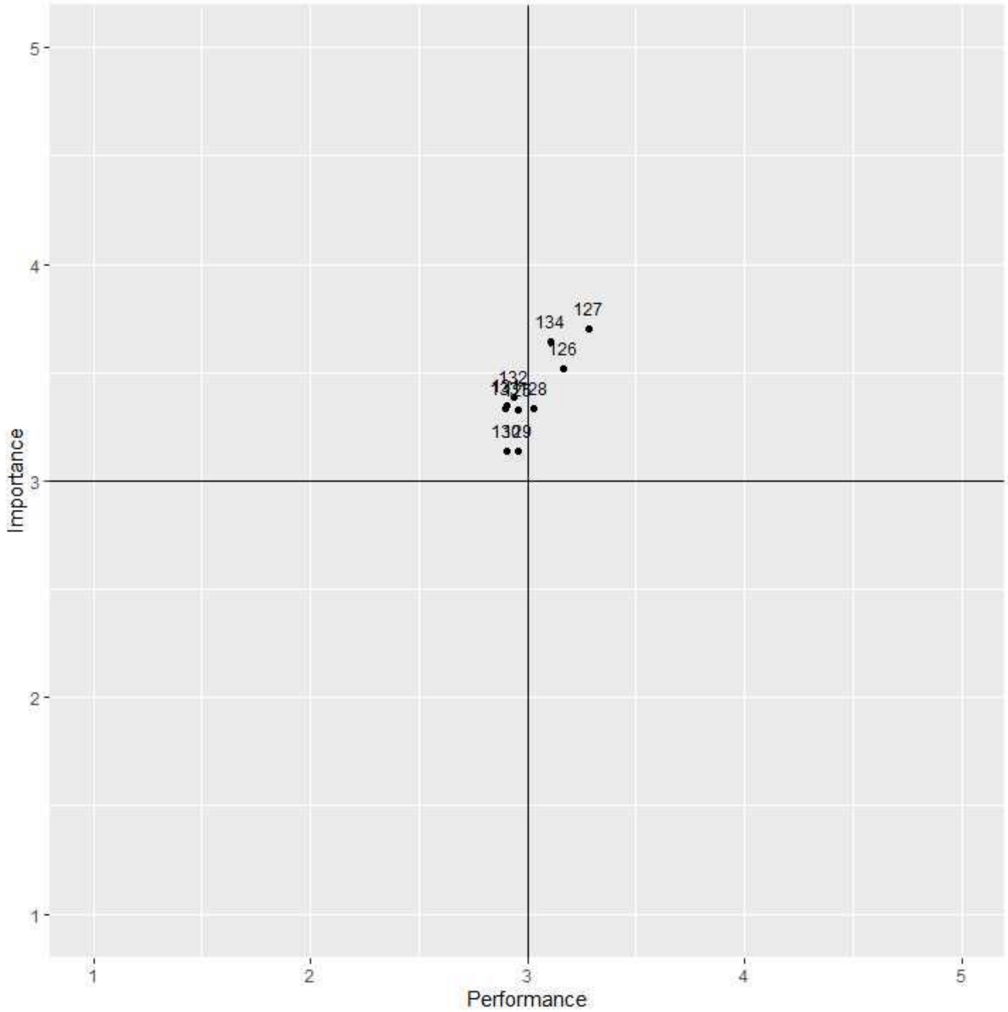

[그림 Ⅳ-9] 건강유지 및 증진 직무의 IPA

8개 직무영역과 연계된 134개 신규간호사 직무항목에 대한 IPA 분석한 결과, 간호관리와 전문성 향상 영역은 22개의 직무항목 중 16개 항목은 2사분위, 4개 항목은 1사분위, 2개 항목은 3사분위에 위치하였다. 건강증진 및 유지 영역의 10개 직무항목 중 4개 항목은 2사분위, 6개 항목은 1사분위에 위치하였다. 안전과 감염관리 영역의 3개 직무항목, 위험요인 사정 영역의 28개 직무항목, 기본간호 영역의 19개의 직무항 목, 생리적 통합유지 영역의 36개 직무항목, 약물 및 비경구 요법 영역의 7개 직무항 목, 심리적 통합유지 영역의 9개 직무항목은 2사분위에 위치하였다.

2사분위에 위치한 직무항목 중에서 안전과 감염관리 영역과 약물 및 비경구 요법 영역의 직무항목은 중요도와 수행도가 다른 영역에 비해서 상대적으로 높은 위치에 분포하였다. 반면에 생리적 통합유지 영역, 심리사회적 통합유지 영역, 건강유지 및 증진 영역의 직무항목의 중요도와 수행도는 안전과 감염관리 영역과 약물 및 비경구 요법 영역의 직무항목에 비해서 상대적으로 낮은 위치에 분포하였다[그림 Ⅳ-10].

1사분위에 위치하고 있는「 8. 질 향상(QI) 활동에 참여 」,「 17. 사례관리활동 참여 」,
「 18. 업무를 조직화하여 일을 효율적으로 관리 」,「 20. 간호대상에 적합한 다양한 자 원 및 매체선택」,「125. (지역사회)건강교육 계획」,「129. 문화간호」,「130. 산업과 환 경 간호」,「131. 재난간호」,「132. 치료계획을 결정하기 위해 가족 역동성(family dynamics) (가족구조, 결속, 의사소통, 경계, 대처기전) 사정」,「134. 건강위험요인 사정과 교육 」등 10개 직무항목은 중요도는 3점 이상이나 수행도가 3점 미만이었다. 1사분위에 위치하고 있는 10개 직무항목 중「 8. 질 향상(QI) 활동에 참여 」,「 17. 사례 관리활동 참여 」,「 18. 업무를 조직화하여 일을 효율적으로 관리 」,「 20. 간호대상에 적 합한 다양한 자원 및 매체선택」 등 4개 직무항목은 간호관리와 전문성 향상 영역이었
고, 「125. (지역사회)건강교육 계획」, 「129. 문화간호」, 「130. 산업과 환경 간호」,
「131. 재난간호」,「132. 치료계획을 결정하기 위해 가족 역동성(family dynamics) (가족구조, 결속, 의사소통, 경계, 대처기전) 사정」,「134. 건강위험요인 사정과 교육」등 6개 직무항목은 건강유지 및 증진 영역이었다. 3사분위에 위치하고 있는「 9. 간호 사업 평가 관련 업무수행(도구개발, 자료조사, 분석, 비교 및 사업개선) 」,「 21. 취약가 족에게 필요한 지역사회 자원활용 」등 2개 직무항목은 중요도와 수행도 모두 3점 미 만으로 나타났다. 3사분위에 위치하고 있는 2개 직무항목은 건강유지 및 증진 영역이 었다. 1사분위, 3사분위에 위치한 12개 직무항목 이외의 122개의 직무항목은 2사분위 에 위치하고 있어서 직무 중요도와 수행도 모두 3점 이상인 것으로 나타났다.

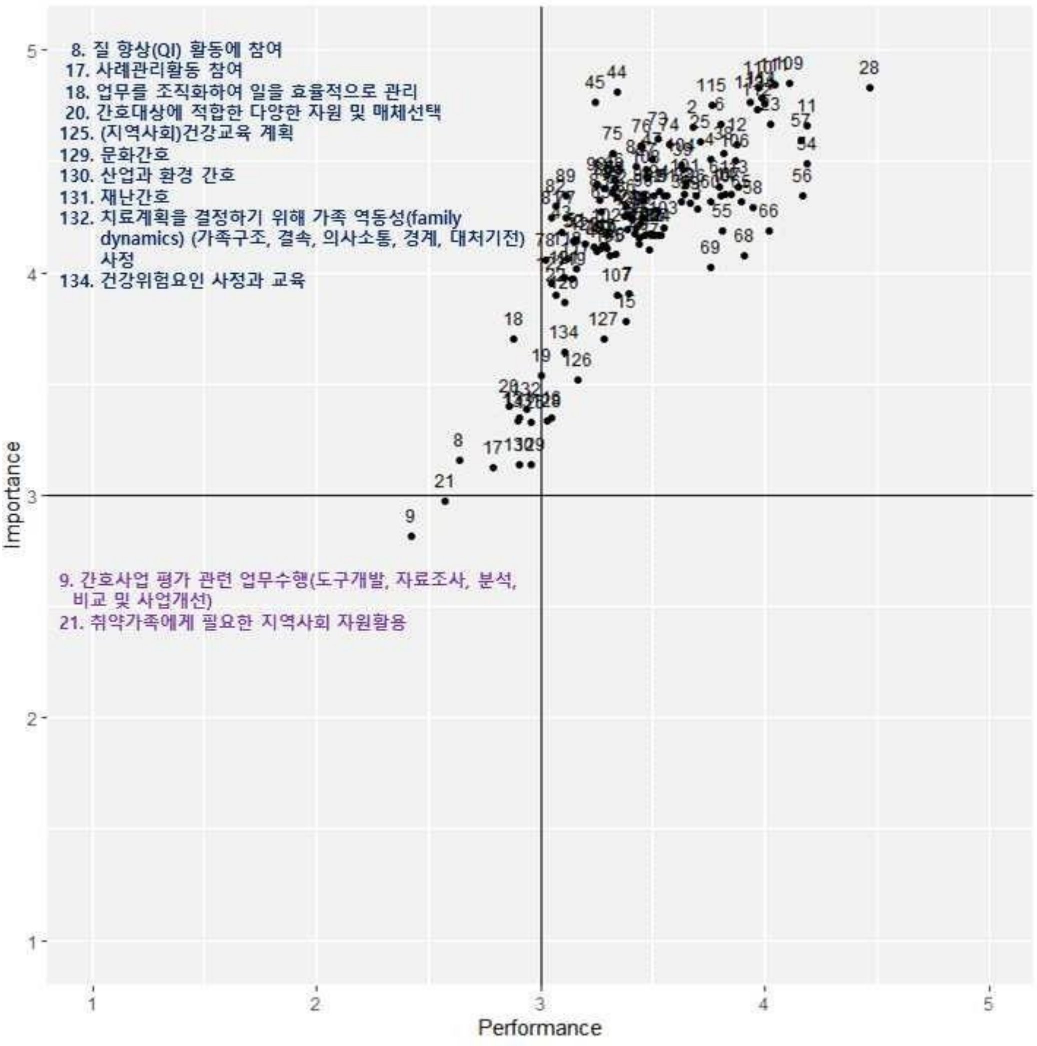

[그림 Ⅳ-10] 간호사 직무의 IPA

3) 8개 직무영역별 신규간호사 직무항목의 개선 요구도

가) 간호관리와 전문성 향상

간호관리와 전문성 향상 영역에 대한 신규간호사 직무 개선 요구도는 <표 Ⅳ-11>과 같다. 대부분의 직무에서 유지에 대한 요구도가 높았으며, 분리에 대한 요구도는「 21. 취약가족에게 필요한 지역사회 자원활용」이 35.00%, 「17. 사례관리활동 참여」 가
34.62%,「 8. 질 향상(QI) 활동에 참여 」가 34.62%,「 9. 간호사업 평가 관련 업무수행 (도구개발, 자료조사, 분석, 비교 및 사업개선) 」이 33.08%,「 16. 지역사회 간호사업 의 법적 기준 및 지침에 따른 활동 참여 」 가 25.38%,「 20. 간호대상에 적합한 다양 한 자원 및 매체선택」 이 25.38%, 「18. 업무를 조직화하여 일을 효율적으로 관리」

가 19.62%,「 19. 환자분류체계 관련 정보수집 및 활용 」이 18.46%,「 7. 간호단위 물 품교환체계에 따른 물품관리」가 18.08%,「22. 전문직간 협업」 이 17.31% 순으로 높게 나타났다.

통합에 대한 요구도는「20. 간호대상에 적합한 다양한 자원 및 매체선택」이 21.92%,「 19. 환자분류체계 관련 정보수집 및 활용 」이 18.46%,「 18. 업무를 조직화 하여 일을 효율적으로 관리 」가 17.31%,「 22. 전문직간 협업 」이 17.31%,「 21. 취약 가족에게 필요한 지역사회 자원활용 」이 13.85%,「 14. 억제대 사용 시 법적 및 윤리 적 간호 」가 13.85%,「 15. 일차의료기반의 보건간호 」가 13.46%,「 16. 지역사회 간 호사업의 법적 기준 및 지침에 따른 활동 참여 」가 13.08%,「 7. 간호단위 물품교환 체계에 따른 물품관리 」가 13.08%,「 10. 간호전문직 윤리 준수와 역할 」이 13.08% 순으로 높게 나타났다.

삭제에 대한 요구도는「 9. 간호사업 평가 관련 업무수행(도구개발, 자료조사, 분석, 비교 및 사업개선) 」이 23.08%,「 8. 질 향상(QI) 활동에 참여 」가 16.54%,「 16. 지역 사회 간호사업의 법적 기준 및 지침에 따른 활동 참여 」가 13.46%,「 21. 취약가족에 게 필요한 지역사회 자원활용 」 이 12.31%, 「 17. 사례관리활동 참여 」 가 10.77%,
「15. 일차의료기반의 보건간호」 가 9.23%,「20. 간호대상에 적합한 다양한 자원 및
매체선택 」이 8.08%,「 19. 환자분류체계 관련 정보수집 및 활용 」이 7.69%,「 7. 간 호단위 물품교환체계에 따른 물품관리」 가 5.38%, 「18. 업무를 조직화하여 일을 효 율적으로 관리 」가 4.23% 순으로 높게 나타났다.

<표 Ⅳ-11> 간호관리와 전문성 향상

(N=260)

번호	항목	유지 n (%)	분리 n (%)	통합 n (%)	삭제 n (%)	결측 n (%)
1	인수인계시	209 (80.38)	25 (9.62)	21 (8.08)	4 (1.54)	1 (0.38)
2	법적 실무범위 내에서 간호 수행	219 (84.23)	16 (6.15)	24 (9.23)	0 (0.00)	1 (0.38)
3	기록시 표준화된 약어사용	216 (83.08)	13 (5.00)	29 (11.15)	2 (0.77)	0 (0.00)
4	지침에 따라 간호기록	215 (82.69)	19 (7.31)	25 (9.62)	1 (0.38)	0 (0.00)
5	입원, 전동, 퇴원	200 (76.92)	35 (13.46)	25 (9.62)	0 (0.00)	0 (0.00)
6	장비를 적절하고 안전하게 사용	207 (79.62)	23 (8.85)	28 (10.77)	0 (0.00)	2 (0.77)
7	간호단위 물품교환체계에 따른 물품관리	165 (63.46)	47 (18.08)	34 (13.08)	14 (5.38)	0 (0.00)
8	질 향상(QI) 활동에 참여	94 (36.15)	90 (34.62)	32 (12.31)	43 (16.54)	1 (0.38)

번호	항목	유지	분리	통합	삭제	결측
9	간호사업 평가 관련 업무수행(도구개발, 자료조사, 분석, 비교 및 사업개선)	86 (33.08)	86 (33.08)	26 (10.00)	60 (23.08)	2 (0.77)
10	간호전문직 윤리 준수와 역할	200 (76.92)	23 (8.85)	34 (13.08)	2 (0.77)	1 (0.38)
11	대상자의 개인정보 및 사생활 보호	223 (85.77)	11 (4.23)	25 (9.62)	1 (0.38)	0 (0.00)
12	대상자에게 치료 및 절차에 따라 적절한 설명을 하고 동의를 획득하였는지 확인	218 (83.85)	12 (4.62)	27 (10.38)	2 (0.77)	1 (0.38)
13	환자의 권리와 책임에 관하여 대상자에게 교육 제공	213 (81.92)	13 (5.00)	30 (11.54)	3 (1.15)	1 (0.38)
14	억제대 사용 시 법적 및 윤리적 간호	209 (80.38)	10 (3.85)	36 (13.85)	1 (0.38)	4 (1.54)
15	일차의료기반의 보건간호	157 (60.38)	43 (16.54)	35 (13.46)	24 (9.23)	1 (0.38)
16	지역사회 간호사업의 법적 기준 및 지침 에 따른 활동 참여	124 (47.69)	66 (25.38)	34 (13.08)	35 (13.46)	1 (0.38)
17	사례관리활동 참여	111 (42.69)	90 (34.62)	29 (11.15)	28 (10.77)	2 (0.77)
18	업무를 조직화하여 일을 효율적으로 관리	152 (58.46)	51 (19.62)	45 (17.31)	11 (4.23)	1 (0.38)
19	환자분류체계 관련 정보수집 및 활용	143 (55.00)	48 (18.46)	48 (18.46)	20 (7.69)	1 (0.38)
20	간호대상에 적합한 다양한 자원 및 매체 선택	115 (44.23)	66 (25.38)	57 (21.92)	21 (8.08)	1 (0.38)
21	취약가족에게 필요한 지역사회 자원활용	100 (38.46)	91 (35.00)	36 (13.85)	32 (12.31)	1 (0.38)
22	전문직간 협업	165 (63.46)	45 (17.31)	45 (17.31)	5 (1.92)	0 (0.00)

나) 안전과 감염관리

안전과 감염관리 영역에 대한 신규간호사 직무 개선 요구도는 다음과 같이 나타났다<표 Ⅳ-12>. 유지에 대한 요구도는「23. 안전한 환경 제공」이 83.85%,「24. 감염관리」가 83.46%,「25. 위험물질과 유해물질 관리」가 75.38% 순으로 높게 나타났으며, 분리에 대한 요구도는「25. 위험물질과 유해물질 관리」가 11.54%,「23. 안전한 환경 제공」이 5.77%,「24. 감염관리」가 5.77% 순으로 나타났다. 통합에 대한 요구도는「25. 위험물질과 유해물질 관리」가 11.92%,「24. 감염관리」가 10.77%,「23. 안전한 환경 제공」이 10.00% 순이었고, 삭제에 대한 요구도는「25. 위험물질과 유해물질 관리」가 1.15%,「23. 안전한 환경 제공」이 0.38%,「24. 감염관리」가 0.00% 순으로 나타났다.

<표 Ⅳ-12> 안전과 감염관리

번호	항목	유지 n (%)	분리 n (%)	통합 n (%)	삭제 n (%)	결측 n (%)
23	안전한 환경 제공	218 (83.85)	15 (5.77)	26 (10.00)	1 (0.38)	0 (0.00)
24	감염관	217 (83.46)	15 (5.77)	28 (10.77)	0 (0.00)	0 (0.00)
25	위험물질과 유해물질 관리	196 (75.38)	30 (11.54)	31 (11.92)	3 (1.15)	0 (0.00)

다) 위험요인 사정

위험요인 사정 영역에 대한 신규간호사 직무 개선 요구도는 다음과 같이 나타났다<표 Ⅳ-13>.

대부분의 직무에서 유지에 대한 요구도가 높았으며, 분리에 대한 요구도는「50. 산후관리와 교육」이 16.54%,「51. 고위험 임부간호」가 16.54%,「53. 고위험 산모간호」가 16.54%,「52. 고위험 산부간호」가 16.54%,「49. 분만 중 간호와 교육」이 15.77%,「48. 산전간호와 교육」이 14.62%,「41. 태아건강사정 및 간호」가 13.85%,「37. 성 건강 간호」가 13.46%,「42. 신생아 건강사정 및 간호」가 13.08%,「43. 고위험신생아 건강사정 및 간호」가 13.08% 순으로 높게 나타났다.

통합에 대한 요구도는「52. 고위험 산부간호」가 18.46%,「51. 고위험 임부간호」

가 18.08%, 「 53. 고위험 산모간호 」 가 18.08%,「 43. 고위험신생아 건강사정 및 간
호」 가 18.08%, 「33. 학령기 간호」 가 17.31%, 「32. 학령전기 간호」 가 17.31%,
「43. 신생아 건강사정 및 간호」 가 16.92%, 「34. 청소년 간호」 가 16.92% 순으로

나타났으며, 삭제에 대한 요구도는「 43. 고위험신생아 건강사정 및 간호 」 가 2.69%,
「42. 신생아 건강사정 및 간호」 가 2.69%,「41. 태아건강사정 및 간호」 가 2.69%,
「 49. 분만 중 간호와 교육 」이 1.92%,「 40. 생식기 건강사정 」이 1.92%,「 37. 성 건 강 간호」 가 1.54%, 「30. 영아 간호」 가 1.54%, 「46. 진단검사 간호」 가 1.54%,
「 52. 고위험 산부간호 」 가 1.15%,「 51. 고위험 임부간호 」 가 1.15%,「 53. 고위험 산
모간호 」가 1.15%,「 31. 유아 간호 」가 1.15%,「 29. 신생아 간호 」가 1.15%,
「 50. 산후관리와 교육 」가 1.15% 순으로 높게 나타났다.

<표 Ⅳ-13> 위험요인 사정

번호	항목	유지 n (%)	분리 n (%)	통합 n (%)	삭제 n (%)	결측 n (%)
26	간호계획, 진료지침 수행	199 (76.54)	14 (5.38)	40 (15.38)	2 (0.77)	5 (1.92)
27	대상자의 건강문제에 대해 우선순위 결정	205 (78.85)	16 (6.15)	35 (13.46)	0 (0.00)	4 (1.54)
28	활력징후 사정	226 (86.92)	5 (1.92)	24 (9.23)	2 (0.77)	3 (1.15)
29	신생아 간	183 (70.38)	31 (11.92)	42 (16.15)	3 (1.15)	1 (0.38)
30	영아 간	182 (70.00)	31 (11.92)	42 (16.15)	4 (1.54)	1 (0.38)
31	유아 간	182 (70.00)	32 (12.31)	42 (16.15)	3 (1.15)	1 (0.38)
32	학령전기 간호	183 (70.38)	31 (11.92)	45 (17.31)	0 (0.00)	1 (0.38)
33	학령기 간	181 (69.62)	32 (12.31)	45 (17.31)	1 (0.38)	1 (0.38)
34	청소년 간	181 (69.62)	33 (12.69)	44 (16.92)	1 (0.38)	1 (0.38)
35	폐경기 여성 간호	184 (70.77)	31 (11.92)	42 (16.15)	2 (0.77)	1 (0.38)
36	노인간	193 (74.23)	29 (11.15)	36 (13.85)	1 (0.38)	1 (0.38)
37	성 건강 간	177 (68.08)	35 (13.46)	42 (16.15)	4 (1.54)	2 (0.77)
38	건강력 사	201 (77.31)	22 (8.46)	35 (13.46)	1 (0.38)	1 (0.38)
39	신체검진 수행 및 결과 해석	199 (76.54)	18 (6.92)	40 (15.38)	2 (0.77)	1 (0.38)
40	생식기 건강사정	183 (70.38)	33 (12.69)	37 (14.23)	5 (1.92)	2 (0.77)
41	태아건강사정 및 간호	175 (67.31)	36 (13.85)	41 (15.77)	7 (2.69)	1 (0.38)
42	신생아 건강사정 및 간호	174 (66.92)	34 (13.08)	44 (16.92)	7 (2.69)	1 (0.38)
43	고위험신생아 건강사정 및 간호	171 (65.77)	34 (13.08)	47 (18.08)	7 (2.69)	1 (0.38)
44	심폐소생술 간호	193 (74.23)	28 (10.77)	36 (13.85)	1 (0.38)	2 (0.77)
45	응급간호	196 (75.38)	27 (10.38)	35 (13.46)	1 (0.38)	1 (0.38)
46	진단검사 간호	190 (73.08)	32 (12.31)	33 (12.69)	4 (1.54)	1 (0.38)

번호	항목	유지 n (%)	분리 n (%)	통합 n (%)	삭제 n (%)	결측 n (%)
47	수술간	196 (75.38)	26 (10.00)	35 (13.46)	2 (0.77)	1 (0.38)
48	산전간호와 교육	179 (68.85)	38 (14.62)	41 (15.77)	1 (0.38)	1 40.38)
49	분만 중 간호와 교육	175 (67.31)	41 (15.77)	38 (14.62)	5 (1.92)	1 (0.38)
50	산후관리와 교육	174 (66.92)	43 (16.54)	39 (15.00)	3 (1.15)	1 (0.38)
51	고위험 임부간호	166 (63.85)	43 (16.54)	47 (18.08)	3 (1.15)	1 (0.38)
52	고위험 산부간호	165 (63.46)	43 (16.54)	48 (18.46)	3 (1.15)	1 (0.38)
53	고위험 산모간호	166 (63.85)	43 (16.54)	47 (18.08)	3 (1.15)	1 (0.38)

라) 기본간호

기본간호 영역에 대한 신규간호사 직무 개선 요구도는 다음과 같다<표 Ⅳ-14>.

대부분의 질문에서 유지에 대한 요구도가 높았으며, 분리에 대한 요구도는 「63. 요루 및 장루관리」가 18.08%,「62. 투석대상자 관리」가 17.31%,「64. 기관절개부 관리 」가 15.77%,「 72. 관절대치술 환자 간호 」가 13.46%,「 71. 척추손상 및 신경질환 대상자의 간호 」가 13.46%,「 70. 외과적 장치 관리 」가 13.08%,「 59. 질환별 영양문 제의 사정 및 관리 」 가 10.38%, 「 61. 배뇨장애 대상자 사정 및 간호 」 가 9.62%, 「 58. 영양사정 및 관리 」가 9.23%,「 68. 이동간호 」가 9.23% 순으로 높게 나타났다. 통합에 대한 요구도는「 63. 요루 및 장루관리 」가 10.77%,「 62. 투석대상자 관리」가 11.15%, 「 64. 기관절개부 관리 」 가 11.54%, 「 72. 관절대치술 환자 간호 」 가 13.46%,「71. 척추손상 및 신경질환 대상자의 간호」가 13.85%,「70. 외과적 장치 관리 」가 13.08%,「 59. 질환별 영양문제의 사정 및 관리 」가 15.77%,「 61. 배뇨장애 대상자 사정 및 간호 」 가 14.23%,「 58. 영양사정 및 관리 」가 16.15%,「 68. 이동간호 」가 13.46% 순으로 높게 나타났다.

삭제에 대한 요구도는 「 68. 이동간호 」 가 3.85%, 「 70. 외과적 장치 관리 」 가 2.69%,「 69. 활동과 자기돌봄 장애 사정 및 간호 」 가 2.69%,「 62. 투석대상자 관리 」가 1.92%, 「71. 척추손상 및 신경질환 대상자의 간호」 가 1.92%, 「64. 기관절개부 관리 」 가 1.54%, 「 72. 관절대치술 환자 간호 」 가 1.54%, 「 59. 질환별

영양문제의 사 정 및 관리 」가 1.54%,「 65. 배변관리 」가 1.54%,「 66. 수면과 휴식 간호 」 가 1.54% 순으로 나타났다.

<표 Ⅳ-14> 기본간호

번호	항목	유지	분리	통합	삭제	결측
54	체온유지간호	212 (81.54)	5 (1.92)	38 (14.62)	1 (0.38)	4 (1.54)
55	세척(irrigation)(방광, 귀, 눈) 수행	202 (77.69)	15 (5.77)	37 (14.23)	3 (1.15)	3 (1.15)
56	개인위생간호	208 (80)	5 (1.92)	42 (16.15)	2 (0.77)	3 (1.15)
57	섭취량 및 배설량 사정과 간호	210 (80.77)	11 (4.23)	36 (13.85)	0 (0.00)	3 (1.15)
58	영양사정 및 관리	188 (72.31)	24 (9.23)	42 (16.15)	1 (0.38)	5 (1.92)
59	질환별 영양문제의 사정 및 관리	184 (70.77)	27 (10.38)	41 (15.77)	4 (1.54)	4 (1.54)
60	섭취장애 대상자 사정 및 간호	191 (73.46)	21 (8.08)	42 (16.15)	1 (0.38)	5 (1.92)
61	배뇨장애 대상자 사정 및 간호	193 (74.23)	25 (9.62)	37 (14.23)	1 (0.38)	4 (1.54)
62	투석대상자 관리	177 (68.08)	45 (17.31)	29 (11.15)	5 (1.92)	4 (1.54)
63	요루 및 장루관리	179 (68.85)	47 (18.08)	28 (10.77)	1 (0.38)	5 (1.92)
64	기관절개부 관리	181 (69.62)	41 (15.77)	30 (11.54)	4 (1.54)	4 (1.54)
65	배변관	190 (73.08)	19 (7.31)	41 (15.77)	4 (1.54)	6 (2.31)
66	수면과 휴식 간호	194 (74.62)	11 (4.23)	46 (17.69)	4 (1.54)	5 (1.92)
67	피부통합성 사정 및 간호	200 (76.92)	16 (6.15)	37 (14.23)	1 (0.38)	6 (2.31)
68	이동간	186 (71.54)	24 (9.23)	35 (13.46)	10 (3.85)	5 (1.92)
69	활동과 자기돌봄 장애 사정 및 간호	183 (70.38)	20 (7.69)	45 (17.31)	7 (2.69)	5 (1.92)
70	외과적 장치 관리	180 (69.23)	34 (13.08)	34 (13.08)	7 (2.69)	5 (1.92)
71	척추손상 및 신경질환 대상자의 간호	179 (68.85)	35 (13.46)	36 (13.85)	5 (1.92)	5 (1.92)
72	관절대치술 환자 간호	181 (69.62)	35 (13.46)	35 (13.46)	4 (1.54)	5 (1.92)

마) 생리적 통합유지

생리적 통합유지 영역에 대한 신규간호사 직무 개선 요구도는 다음과 같다<표 Ⅳ-15>.

대부분의 질문에서 유지에 대한 요구도가 높았으며, 분리에 대한 요구도는「 78. 고위험 신생아 보육기 적용간호」가 17.69%, 「77. 태아질식 증상과 징후 사정 및 간호 」가 16.54%,「 107. 재활간호서비스 」가 15.00%,「 79. 호흡재활관리 」가 14.62%, 「81. 순환 보조장치 관리」가 14.62%,「108.(법정) 감염질환자의 전파예방 및 간호」가 14.23%,「 98. 두개내압 상승 환자의 간호 」가 13.46%,「 89. 심장수술 후 간호 」가 13.08%, 「102. 화상환자 간호중재」 가 13.08%, 「103. 계통별 신생물 질환 대상자

간호 」가 13.08% 순으로 높게 나타났다.

통합에 대한 요구도는「 74. 호흡증진 중재 」가 16.15%,「 108. (법정) 감염질환자의 전파예방 및 간호」 가 15.00%, 「83. 조직 관류 장애 대상자 사정 및 간호」 가
14.23%, 「 73. 호흡기능장애 대상자 간호 」 가 14.23%, 「 107.
재활간호서비스 」가
13.85%,「 75. 호흡보조장치 관리 」가 13.85%,「 95. 생식기 질환/생식기
건강문제를 가진 대상자 간호 」 가 13.85%,「 84. 체액 전해질 불균형 사정 및 간호 」 가 13.46%,
「103. 피부질환 대상자의 간호중재」가 13.46%,「105. 배액장치 관리」가 13.46% 순으로 높게 나타났다.

삭제에 대한 요구도는「 105. 계통별 신생물 질환 대상자 간호 」가 4.23%,「 78. 고 위험 신생아 보육기 적용간호 」 가 3.85%,「 107. 재활간호서비스 」 가 3.08%,「 7. 호
흡재활관리」 가 3.08%, 「9. 순환 보조장치 관리」 가 2.31%, 「30. 화상환자 간호중
재 」가 2.31%,「 89. 심장 수술 후 간호 」가 2.31%,「 77. 태아질식 증상과 징후 사정 및 간호 」 가 1.92%, 「 75. 호흡보조장치 관리 」 가 1.54%, 「 80. 심전도 관리 」가
1.54%,「82. 동맥관 관리」가 1.15%,「97. 감각기능 장애를 가진 대상자 사정 및 간
호 」가 1.15% 순으로 높게 나타났다.

<표 Ⅳ-15> 생리적 통합유지

번호	항목	유지	분리	통합	삭제	결측
73	호흡기능장애 대상자 간호	199 (76.54) (77.69)	84	체	간	및간호 21 (8.08)
74	호흡증진 중재	193 (74.23)		액	호	2 22 (8.46)
75	호흡보조장치 관리	187 (71.92)		전	2	0 5 30 (11.54)
76	중심정맥관 관리	198 (76.15)		해	0	27 (10.38)
77	태아질식 증상과 징후 사정 및 간호	177 (68.08)		질	2	43 (16.54)
78	고위험 신생아 보육기 적용간호	168 (64.62)		불 85	활동	46 (17.69)
79	호흡재활관리	180 (69.23)		균	지속	38 (14.62)
80	심전도 관	193 (74.23)		형	성장	26 (10.00)
81	순환 보조장치 관리	181 (69.62)		사	애대	38 (14.62)
82	동맥관 관	189 (72.69)		정	상자	33 (12.69)
83	조직 관류 장애 대상자 사정 및 간호	196 (75.38)		및	사정	23 (8.85)
						20

(7.69)	37	0	3	
23	(14.23)	(0.00)	(1.15)	
	42	0	3	
	(16.15)	(0.00)	(1.15)	
	36	4	3	
	(13.85)	(1.54)	(1.15)	
	32	0	3	
	(12.31)	(0.00)	(1.15)	
	33	5	2	
	(12.69)	(1.92)	(0.77)	
	32	10	4	
	(12.31)	(3.85)	(1.54)	
	31	8	3	
	(11.92)	(3.08)	(1.15)	
	34	4	3	
	(13.08)	(1.54)	(1.15)	
	33	6	2	
	(12.69)	(2.31)	(0.77)	
	33	3	2	
	(12.69)	(1.15)	(0.77)	
	37	2	2	
	(14.23)	(0.77)	(0.77)	
	35	1	2	
	(13.46)	(0.38)	(0.77)	
	29	1	2	
(78.85)	(8.85)	(11.15)	(0.38)	(0.77)

번호	항목	유지<br>n (%)	분리<br>n (%)	통합<br>n (%)	삭제<br>n (%)	결측<br>n (%)
86	정맥 순환증진 장치 간호	205<br>(78.85)	19<br>(7.31)	33<br>(12.69)	1<br>(0.38)	2<br>(0.77)
87	혈액 기능장애 대상자 간호	205<br>(78.85)	19<br>(7.31)	34<br>(13.08)	0<br>(0.00)	2<br>(0.77)
88	순환 기능장애 대상자 간호	206<br>(79.23)	18<br>(6.92)	33<br>(12.69)	1<br>(0.38)	2<br>(0.77)
89	심장 수술 후 간호	194<br>(74.62)	34<br>(13.08)	24<br>(9.23)	6<br>(2.31)	2<br>(0.77)
90	소화 기능장애 대상자 간호	[illegible]<br>(82.31)	[illegible]<br>(6.15)	[illegible]<br>(10.00)	[illegible]<br>(0.00)	[illegible]<br>(1.54)
91	배뇨장애를 가진 대상자 간호	213<br>(81.92)	16<br>(6.15)	28<br>(10.77)	0<br>(0.00)	3<br>(1.15)
92	당질 대사장애 대상자 간호	210<br>(80.77)	18<br>(6.92)	30<br>(11.54)	0<br>(0.00)	2<br>(0.77)
93	당뇨병 합병증 예방을 위한 간호	213<br>(81.92)	12<br>(4.62)	33<br>(12.69)	1<br>(0.38)	1<br>(0.38)
94	내분비계 장애 대상자 간호	212<br>(81.54)	15<br>(5.77)	32<br>(12.31)	0<br>(0.00)	1<br>(0.38)
95	생식기 질환/생식기 건강문제를 가진 대상자 간호	201<br>(77.31)	20<br>(7.69)	36<br>(13.85)	1<br>(0.38)	2<br>(0.77)
96	면역손상 대상자 사정 및 간호	203<br>(78.08)	24<br>(9.23)	32<br>(12.31)	0<br>(0.00)	1<br>(0.38)
97	감각기능 장애를 가진 대상자 사정 및 간호	199<br>(76.54)	25<br>(9.62)	32<br>(12.31)	3<br>(1.15)	1<br>(0.38)
98	두개내압 상승 환자의 간호	195<br>(75)	35<br>(13.46)	28<br>(10.77)	1<br>(0.38)	1<br>(0.38)
99	신경계 질환별 간호중재	206<br>(79.23)	25<br>(9.62)	28<br>(10.77)	0<br>(0.00)	1<br>(0.38)
100	운동기능 장애 간호중재	201<br>(77.31)	28<br>(10.77)	28<br>(10.77)	2<br>(0.77)	1<br>(0.38)
101	상처간호 수행 및 드레싱교환	197<br>(75.77)	30<br>(11.54)	29<br>(11.15)	2<br>(0.77)	2<br>(0.77)
102	화상환자 간호중재	190<br>(73.08)	34<br>(13.08)	29<br>(11.15)	6<br>(2.31)	1<br>(0.38)
103	피부질환 대상자의 간호중재	202<br>(77.69)	19<br>(7.31)	35<br>(13.46)	2<br>(0.77)	2<br>(0.77)
104	배액장치 관리	204<br>(78.46)	17<br>(6.54)	35<br>(13.46)	1<br>(0.38)	3<br>(1.15)
105	계통별 신생물 질환 대상자 간호	184<br>(70.77)	34<br>(13.08)	29<br>(11.15)	11<br>(4.23)	2<br>(0.77)
106	통증간	212<br>(81.54)	12<br>(4.62)	34<br>(13.08)	0<br>(0.00)	2<br>(0.77)
107	재활간호서비스	175<br>(67.31)	39<br>(15.00)	36<br>(13.85)	8<br>(3.08)	2<br>(0.77)
108	(법정) 감염질환자의 전파예방 및 간호	181<br>(69.62)	37<br>(14.23)	39<br>(15.00)	2<br>(0.77)	1<br>(0.38)

바) 약물 및 비경구요법

약물 및 비경구요법 항목에 대한 신규간호사 직무 개선 요구도는 다음과 같다<표 Ⅳ-16>.

대부분의 질문에서 유지에 대한 요구도가 높았으며, 분리에 대한 요구도는 「 115. 고위험 약품관리(마약관리)」가 9.62%, 「114. 대상자에게 약물에 대해 교육」이

6.92%,「110. 약물투여에 필요한 계산 시행」이 5.38%,「112. 약품관리」가 5.00%,
「 113. 말초정맥관 삽입, 유지, 제거 」 가 4.62%,「 111. 정맥주입 장치 관리 」 가
3.85%,「109. 투약의 적절성과 정확성」이 3.46% 순으로 나타났다.

통합에 대한 요구도는 「112. 약품관리」가 12.31%, 「115. 고위험 약품관리(마약관
리) 」가 11.92%,「 114. 대상자에게 약물에 대해 교육 」이 10.38%,「 110. 약물투여에 필요한 계산 시행 」 이 10.38%, 「 113. 말초정맥관 삽입, 유지, 제거 」 가 10.00%,
「 111. 정맥주입 장치 관리 」가 10.00%,「 109. 투약의 적절성과 정확성 」이 10.00% 순으로 나타났다.

삭제에 대한 요구도는「 115. 고위험 약품관리(마약관리) 」가 0.77%,「 113. 말초정 맥관 삽입, 유지, 제거」가 0.38%,「109. 투약의 적절성과 정확성」이 0.38% 순으로
나타났으며,「 111. 정맥주입 장치 관리 」,「 112. 약품관리 」,「 114. 대상자에게 약물에 대해 교육 」,「 110. 약물투여에 필요한 계산 시행 」에서 0.00%로 나타났다.

<표 Ⅳ-16> 약물 및 비경구요법

번호	항목	유지 n (%)	분리 n (%)	통합 n (%)	삭제 n (%)	결측 n (%)
109	투약의 적절성과 정확성	222 (85.38)	9 (3.46)	26 (10.00	1 (0.38)	2 (0.77)
110	약물투여에 필요한 계산 시행	218 (83.85)	14 (5.38)	27 (10.38)	0 (0.00)	1 (0.38)
111	정맥주입 장치 관리	222 (85.38)	10 (3.85)	26 (10.00	0 (0.00)	2 (0.77)
112	약품관	213 (81.92)	13 (5)	32 (12.31)	0 (0.00)	2 (0.77)
113	말초정맥관 삽입, 유지, 제거	220 (84.62)	12 (4.62)	26 (10.00	1 (0.38)	1 (0.38)
114	대상자에게 약물에 대해 교육	214 (82.31)	18 (6.92)	27 (10.38)	0 (0.00)	1 (0.38)
115	고위험 약품관리(마약관리)	201 (77.31)	25 (9.62)	31 (11.92)	2 (0.77)	1 (0.38)

사) 심리사회적 통합 유지

심리사회적 통합 유지 항목에 대한 신규간호사 직무 개선 요구도는 다음과 같이 나타났다<표 Ⅳ-17>.

대부분의 질문에서 유지에 대한 요구도가 높았으며, 분리에 대한 요구도는
「 122. 아동 정신질환자 간호」 가 23.08%, 「120. 정신사회건강문제 간호 및 교육」 이

21.92%,「 121. 정신질환자 간호 」가 21.54%,「 118. 학대 또는 방임 대상자를 확인하 고 적절하게 중재」가 21.15%,「119. 중독 사정 및 간호」가 20.77%,「117. 폭력의

잠재성 평가 및 예방 」이 19.23%,「 123. 임종간호와 교육 」이 18.46%,「 116. 이상 행동 사정 및 간호 」 가 16.92%,「 124. 치료적 의사소통 기법 」 이 14.62% 순으로 높 게 나타났다.

통합에 대한 요구도는「118. 학대 또는 방임 대상자를 확인하고 적절하게 중재」가
16.15%, 「117. 폭력의 잠재성 평가 및 예방」 이 16.15%, 「116. 이상행동 사정 및
간호 」가 16.15%,「 120. 정신사회건강문제 간호 및 교육 」이 15.77%,「 124. 치료적 의사소통 기법 」이 15.00%,「 119. 중독 사정 및 간호 」가 14.23%,「 122. 아동 정신 질환자 간호」 가 13.85%, 「121. 정신질환자 간호」 가 13.85%, 「123. 임종간호와 교
육 」이 11.92% 순으로 나타났다.

삭제에 대한 요구도는「118. 학대 또는 방임 대상자를 확인하고 적절하게 중재」가
2.31%,「 117. 폭력의 잠재성 평가 및 예방 」이 2.31%,「 120. 정신사회건강문제 간호 및 교육」 이 1.92%,「122. 아동 정신질환자 간호」 가 1.92%,「123. 임종간호와 교
육」이 1.92%,「119. 중독 사정 및 간호」가 1.54%, 「121. 정신질환자 간호」 가 1.15% 순으로 나타났으며,「116. 이상행동 사정 및 간호」,「124. 치료적 의사소통 기
법 」이 0.00%로 나타났다.

<표 Ⅳ-17> 심리사회적 통합 유지

번호	항목	유지 n (%)	분리 n (%)	통합 n (%)	삭제 n (%)	결측 n (%)
116	이상행동 사정 및 간호	171 (65.77)	44 (16.92)	42 (16.15)	0 (0.00)	3 (1.15)
117	폭력의 잠재성 평가 및 예방	161 (61.92)	50 (19.23)	42 (16.15)	6 (2.31)	1 (0.38)
118	학대 또는 방임 대상자를 확인하고 적절하게 중재	156 (60.00)	55 (21.15)	42 (16.15)	6 (2.31)	1 (0.38)
119	중독 사정 및 간호	164 (63.08)	54 (20.77)	37 (14.23)	4 (1.54)	1 (0.38)
120	정신사회건강문제 간호 및 교육	156 (60.00	57 (21.92)	41 (15.77)	5 (1.92)	1 (0.38)
121	정신질환자 간호	164 (63.08)	56 (21.54)	36 (13.85)	3 (1.15)	1 (0.38)
122	아동 정신질환자 간호	158 (60.77)	60 (23.08)	36 (13.85)	5 (1.92)	1 (0.38)
123	임종간호와 교육	174 (66.92)	48 (18.46)	31 (11.92)	5 (1.92)	2 (0.77)
124	치료적 의사소통 기법	181 (69.62)	38 (14.62)	39 (15.00)	0 (0.00)	2 (0.77)

아) 건강유지 및 증진

건강유지 및 증진 항목에 대한 신규간호사 직무 개선 요구도는 다음과 같다<표 Ⅳ-18>.

유지에 대한 요구도는「 127. 고위험 건강행위의 예방 및 치료에 대한 정보(금연, 안 전한 성 행위, 바늘교환)제공 」이 52.69%,「 126. 건강증진 및 유지관리에 대한 정보 (예방접종 등)제공 」이 52.31%, 「 134. 건강위험요인 사정과 교육 」이 52.31%,「 125. (지역사회)건강교육 계획 」이 44.62%,「 132. 치료계획을 결정하기 위해 가족 역동성 (family dynamics) (가족구조, 결속, 의사소통, 경계, 대처기전) 사정 」 이 44.62%, 「128. 성 건강증진 간호 」 가 43.85%,「130. 산업과 환경 간호」 가 42.69%,「131. 재난간호 」가 41.92%,「 133. 가정환경에서 대상자를 관리할 수 있는 역량(장비, 지역 사회 자원) 평가」가 41.92%,「129. 문화간호」가 38.08% 순으로 나타났다.

분리에 대한 요구도는「 133. 가정환경에서 대상자를 관리할 수 있는 역량(장비, 지역사회 자원) 평가」 가 31.92%,「129. 문화간호」 가 31.92%,「130. 산업과 환경 간호」 가 30.38%,「131. 재난간호」 가 30.38%,「128. 성 건강증진 간호」 가 29.62%, 「 132. 치료계획을 결정하기 위해 가족 역동성(family dynamics) (가족구조, 결속, 의사소통, 경계, 대처기전) 사정 」이 28.08%, 「 125. (지역사회)건강교육 계획 」 이 25.77%,「127. 고위험 건강행위의 예방 및 치료에 대한 정보(금연, 안전한 성 행위, 바늘교환)제공 」이 23.08%,「 134. 건강위험요인 사정과 교육 」이 21.92%,「 126. 건강증진 및 유지관리에 대한 정보(예방접종 등)제공」이 21.15% 순으로 나타났다.

통합에 대한 요구도는「 134. 건강위험요인 사정과 교육 」이 19.62%,「 126. 건강증 진 및 유지관리에 대한 정보(예방접종 등)제공 」 이 19.62%,「 132. 치료계획을 결정하 기 위해 가족 역동성(family dynamics) (가족구조, 결속, 의사소통, 경계, 대처기전) 사정 」이 18.85%,「 127. 고위험 건강행위의 예방 및 치료에 대한 정보(금연, 안전한 성 행위, 바늘교환)제공」이 18.46%,「133. 가정환경에서 대상자를 관리할 수 있는 역량(장비, 지역사회 자원) 평가 」 가 17.69%, 「 125. (지역사회)건강교육 계획 」 이 17.31%,「131. 재난간호」 가 16.92%,「128. 성 건강증진 간호」 가 16.54%,「130. 산업과 환경 간호 」가 14.62%,「 129. 문화간호 」가 14.23% 순으로 높게 나타났다.

삭제에 대한 요구도는 「129. 문화간호」 가 14.62%, 「125.

(지역사회)건강교육 계
획」 이 11.15%,「130. 산업과 환경 간호」가 11.15%,「131. 재난간호」가 9.62%,
「128. 성 건강증진 간호」가 8.46%,「132. 치료계획을 결정하기 위해 가족 역동성 (family dynamics) (가족구조, 결속, 의사소통, 경계, 대처기전) 사정 」이 7.31%,
「 133. 가정환경에서 대상자를 관리할 수 있는 역량(장비, 지역사회 자원) 평가 」가

7.31%, 「 126. 건강증진 및 유지관리에 대한 정보(예방접종 등)제공 」이 6.15%, 「 134. 건강위험요인 사정과 교육 」이 4.62%, 「 127. 고위험 건강행위의 예방 및 치료에 대 한 정보(금연, 안전한 성 행위, 바늘교환)제공」 이 4.62% 순으로 나타났다.

<표 Ⅳ-18> 건강유지 및 증진

번호	항목	유지	분리	통합	삭제	결측
125	(지역사회)건강교육 계획	116 (44.62)	67 (25.77)	45 (17.31)	29 (11.15)	3 (1.15)
126	건강증진 및 유지관리에 대한 정보(예방접종 등)제공	136 (52.31)	55 (21.15)	51 (19.62)	16 (6.15)	2 (0.77)
127	고위험 건강행위의 예방 및 치료에 대한 정보(금연, 안전한 성 행위, 바늘교환)제공	137 (52.69)	60 (23.08)	48 (18.46)	12 (4.62)	3 (1.15)
128	성 건강증진 간호	114 (43.85)	77 (29.62)	43 (16.54)	22 (8.46)	4 (1.54)
129	문화간	99 (38.08)	83 (31.92)	37 (14.23)	38 (14.62)	3 (1.15)
130	산업과 환경 간호	111 (42.69)	79 (30.38)	38 (14.62)	29 (11.15)	3 (1.15)
131	재난간	109 (41.92)	79 (30.38)	44 (16.92)	25 (9.62)	3 (1.15)
132	치료계획을 결정하기 위해 가족 역동성(family dynamics) (가족구조, 결속, 의사소통, 경계, 대처기전) 사정	116 (44.62)	73 (28.08)	49 (18.85)	19 (7.31)	3 (1.15)
133	가정환경에서 대상자를 관리할 수 있는 역량(장비, 지역사회 자원) 평가	109 (41.92)	83 (31.92)	46 (17.69)	19 (7.31)	3 (1.15)
134	건강위험요인 사정과 교육	136 (52.31)	57 (21.92)	51 (19.62)	12 (4.62)	4 (1.54)

8개 직무영역별 134개 신규간호사 직무항목의 개선 요구도에 대한 자료분석 결과, 간호관리와 전문성 향상 영역의 22개 직무항목에서 항목별로 삭제에 응답한 응답율은
「 9. 간호사업 평가 관련 업무수행(도구개발, 자료조사, 분석, 비교 및 사업개선) 」
23.08%, 「 8. 질 향상(QI) 활동에 참여 」 16.54%, 「 16. 지역사회 간호사업의 법적 기준 및 지침에 따른 활동 참여」13.46%, 「21. 취약가족에게 필요한 지역사회 자원활용」
12.31%, 「 17. 사례관리활동 참여 」 10.77% 순이었다. 건강증진 및 유지 영역의 10개 직무항목에서 항목별로 삭제에 응답한 응답율은 「 129. 문화간호 」 14.62%,

「 130. 산 업과 환경 간호 」11.15%,「 125. (지역사회)건강교육 계획 」11.15%,
「 131. 재난간호 」
9.62%,「 128. 성 건강 증진 간호 」8.46% 순이었다. 안전과 감염관리 영역의 3개 직 무항목에서 항목별로 삭제에 응답한 응답률은 1.15% 이하, 위험요인 사정 영역의 28 개 직무항목에서 항목별로 삭제에 응답한 응답률은 2.69% 이하, 기본간호 영역의 19

개 직무항목에서 항목별로 삭제에 응답한 응답률은 3.85% 이하, 생리적 통합유지 영역의 19개 직무항목에서 항목별로 삭제에 응답한 응답률은 4.23%이하, 약물 및 비경 구 요법 영역의 7개 직무항목에서 항목별로 삭제에 응답한 응답률은 0.77% 이하, 심 리사회적 통합유지 영역의 9개 직무항목에서 항목별로 삭제에 응답한 응답률은 2.31% 이하이었다.

이상에서 살펴본 바와 같이 출제모형의 간호사 직무항목에 대한 타당성과 적절성 평가를 위해 신규간호사 직무항목의 중요도와 수행도 IPA 분석에서는 간호관리와 전문성 향상 영역의「 8. 질 향상(QI) 활동에 참여 」,「 17. 사례관리활동 참여 」,「 18. 업 무를 조직화하여 일을 효율적으로 관리」,「20. 간호대상에 적합한 다양한 자원 및 매
체선택」 등 4개 직무항목, 건강증진 및 유지 영역의「125. (지역사회)건강교육 계획」,
「129. 문화간호」, 「130. 산업과 환경 간호」, 「131. 재난간호」, 「132. 치료계획을 결 정하기 위해 가족 역동성(family dynamics) (가족구조, 결속, 의사소통, 경계, 대처기 전) 사정 」,「 134. 건강위험요인 사정과 교육 」등 6개 직무항목의 중요도는 3점 이상 이었으나 수행도는 3점 미만으로 1사분위에 위치하였다. 그리고 간호관리와 전문성 향상 영역의「9. 간호사업 평가 관련 업무수행(도구개발, 자료조사, 분석, 비교 및 사
업개선)」,「21. 취약가족에게 필요한 지역사회 자원활용」 등 2개 직무항목은 중요도 와 수행도 모두 3점 미만으로 3사분위에 위치하였다.

신규간호사 직무항목의 중요도 및 수행도 IPA 분석결과 와 개선 요구도 분석 결과를 비교해보면,「 8. 질 향상(QI) 활동에 참여 」,「 9. 간호사업 평가 관련 업무수행(도구 개발, 자료조사, 분석, 비교 및 사업개선) 」,「 17. 사례관리활동 참여 」,「 21. 취약가족 에게 필요한 지역사회 자원활용 」 등 간호관리와 전문성 향상 영역의 4개 직무항목,
「125. (지역사회)건강교육 계획」, 「129. 문화간호」,「130. 산업과 환경 간호」, 「131.
재난간호 」등 건강유지 및 증진 영역의 4개 직무항목은 중요도와 수행도 IPA 분석결 과 1사분위, 3사분위에 위치하는 직무항목이면서 개선요구도 분석에서 삭제에 응답한 응답률 상위 5위 범주에도 포함되는 직무항목 이었다. 따라서 IPA 분석결과 1사분위, 3사분위에 위치하는 12개 직무항목은 개선 요구도에서 삭제에 응답한 응답율로 분석 한 상위 5위 범주의 직무항목과 8개 항목이 중복되었다.

이와 같은 설문조사 분석 결과에 기반하여 임상 및 지역사회 간호실무 현장에 근무하는 전문가를 대상으로 12개 직무항목(8,9,17,18,20,21,125,129,130,131,132,134)의 타당성과 적절성을 확인하기 위해 인터뷰를 하였고, 134개 직무항목에 대한 내용타당도 조사를 실시하였다.

나. 출제모형의 간호사 직무항목에 대한 전문가 인터뷰 및 타당도 조사

1) 전문가 인터뷰

134개 신규간호사 직무항목의 시의성과 타당성, 적절성 평가를 위해 임상 및 지역사회 간호실무 현장에서 근무하는 간호사 260명을 대상으로 134개 신규간호사 직무항목에 대한 설문조사를 실시하여 직무 중요도 및 수행도 3점을 기준으로 IPA 분석결과, 134개의 직무항목 중에서 122개 항목은 중요도와 수행도 모두 3점 이상이었고,
「8. 질 향상(QI) 활동에 참여」,「17. 사례관리활동 참여」,「18. 업무를 조직화하여 일을 효율적으로 관리」,「20. 간호대상에 적합한 다양한 자원 및 매체선택」,「125. (지역사 회)건강교육 계획」, 129. 문화간호」,「130. 산업과 환경 간호」,「131. 재난간호」,「132. 치료계획을 결정하기 위해 가족 역동성(family dynamics) (가족구조, 결속, 의사소 통, 경계, 대처기전) 사정 」, 「 134. 건강위험요인 사정과 교육 」 등 10개 직무항목은 중 요도는 3점 이상이었으나 수행도가 3점 미만이었지만, 거의 3점에 근접하였다. 「 9. 간호사업 평가 관련 업무수행(도구개발, 자료조사, 분석, 비교 및 사업개선) 」, 「 21. 취 약가족에게 필요한 지역사회 자원활용 」은 등 2개 직무항목은 중요도와 수행도 모두 3 점 미만이었으나 거의 3점에 근접하였다.

134개 신규간호사 직무항목에 대한 개선 요구도 분석결과, 간호관리와 전문성 향상 영역에서 삭제에 응답한 직무항목별 응답율은「 9. 간호사업 평가 관련 업무수행(도구개발, 자료조사, 분석, 비교 및 사업개선) 」, 「 8. 질 향상(QI) 활동에 참여 」, 「 16. 지역 사회 간호사업의 법적 기준 및 지침에 따른 활동 참여」, 「21. 취약가족에게 필요한 지역사회 자원활용 」, 「 17. 사례관리활동 참여 」순이었다. 건강증진 및 유지 영역에서 삭제에 응답한 직무항목별 응답율은 「 129. 문화간호 」, 「 130. 산업과 환경 간호 」,
「125. (지역사회)건강교육 계획」, 「131. 재난간호」, 「128. 성 건강 증진 간호」순이었다.

이와 같은 분석 결과에 대해 임상 및 지역사회 간호실무현장 전문가를 대상으로 전 화 인터뷰를 하였다. 총 7인의 전문가가 참여하였으며, 소속 기관은 학교 보건교사 1 인(14.3%), 상급종합병원 간호사 6인(85.7%) 이었다. 근무기간은 7년 이상 6인 (85.7%), 보건교사 16년, 현재 근무하고 있는 부서는 내·외과계 4인(57.1%), 소아청소 년과, 정신과, 학교가 각 1인(14.3%)이었다.

신규간호사 12개 직무항목(8,9,17,18,20,21,125,129,130,131,132,134)에 대해 임상
및 지역사회 간호 실무현장 전문가 집단을 대상으로 전화 인터뷰를 실시한 결과, 대부분 '모든 간호사의 책임과 의무(8.17.125,134)', '확대되고 있는 간호사의 역할에서

중요한 역할이 될 것(20,129,130,132)’, ‘임상 및 지역사회 간호사에게 필요한 내용(21,132)’, ‘간호과정 적용과 유사한 과정(9)’, ‘이직을 감소시킬 수 있는 역량(18) 등의 긍정적 답변이었다[부록 2]. 따라서 본 연구진은 전문가 인터뷰 결과와 더불어 변화하는 보건의료환경과 간호사의 역할 확대 측면에서 고려해 볼 때 12개 직무항목은 타당하면서 적절하다고 평가하였다.

2) 내용타당도 조사

134개 직무항목의 타당도는 4점 척도로 평가하고 수정의견이 있을 경우에는 기술하도록 하였다. 평가 결과 3, 4점(타당함, 매우 타당함)인 항목은 1점, 1, 2점(매우 타당하지 않음, 타당하지 않음)인 항목은 0점으로 계산하여 산출한 문항 내용타당도 지수(Item-level Content Validity Index, I-CVI)에서 0.8 미만인 항목은 없었다.

가) 간호관리와 전문성 향상

간호관리와 전문성 향상 영역의 직무항목 내용타당도 지수는 다음과 같다<표 Ⅳ-19>. ‘7. 간호단위 물품교환체계에 따른 물품관리’ 항목에 대해 신규간호사의 경우 관리자처럼 포괄적인 물품관리가 명확하지 않으므로 ‘환자처치관련 물품 확인’, ‘처치 등 관리영역 일지’로 수정의견을 제시하였다.

<표 Ⅳ-19> 간호관리와 전문성 향상

번호	항목	Mean±SD	I-CVI
1	인수인계시행	4.00±0.000	1.00
2	법적 실무범위 내에서 간호 수행	4.00±0.000	1.00
3	기록시 표준화된 약어사용	4.00±0.000	1.00
4	지침에 따라 간호기록	4.00±0.000	1.00
5	입원, 전동, 퇴원	3.86±0.378	1.00
6	장비를 적절하고 안전하게 사용	3.71±0.488	1.00
7	간호단위 물품교환체계에 따른 물품관리	3.43±0.787	0.86
8	질 향상(QI) 활동에 참여	3.29±0.488	1.00
9	간호사업 평가 관련 업무수행(도구개발, 자료조사, 분석, 비교 및 사업개선) 3.29±0.488		1.00

번호	항목	Mean±SD	I-CVI
10	간호전문직 윤리 준수와 역할	4.00±0.000	1.00
11	대상자의 개인정보 및 사생활 보호	4.00±0.000	1.00
12	대상자에게 치료 및 절차에 따라 적절한 설명을 하고 동의를 획득하였는지 확인	4.00±0.000	1.00
13	환자의 권리와 책임에 관하여 대상자에게 교육 제공	3.86±0.378	1.00
14	억제대 사용 시 법적 및 윤리적 간호	3.86±0.378	1.00
15	일차의료기반의 보건간호	3.57±0.535	1.00
16	지역사회 간호사업의 법적 기준 및 지침에 따른 활동 참여	3.57±0.535	1.00
17	사례관리활동 참여	3.43±0.535	1.00
18	업무를 조직화하여 일을 효율적으로 관리	3.43±0.535	1.00
19	환자분류체계 관련 정보수집 및 활용	3.71±0.488	1.00
20	간호대상에 적합한 다양한 자원 및 매체선택	3.57±0.535	1.00
21	취약가족에게 필요한 지역사회 자원활용	3.29±0.488	1.00
22	전문직간 협업	3.71±0.488	1.00

나) 안전과 감염관리

안전과 감염관리 영역의 직무항목 내용타당도 지수는 다음과 같다<표 Ⅳ-20>.

<표Ⅳ-20> 안전과 감염관리

번호	항목	Mean±SD	I-CVI
23	안전한 환경 제공	4.00±0.000	1.00
24	감염관리	4.00±0.000	1.00
25	위험물질과 유해물질 관리	3.86±0.378	1.00

다) 위험요인 사정

위험요인 사정 영역의 직무항목 내용타당도 지수는 다음과 같다<표 Ⅳ-21>.

<표 Ⅳ-21> 위험요인 사정

번호	항목	Mean±SD	I-CVI
26	간호계획, 진료지침 수행	4.00±0.000	1.00
27	대상자의 건강문제에 대해 결정 우선순위	4.00±0.000	1.00
28	활력징후 사정	4.00±0.000	1.00
29	신생아 간호	3.86±0.378	1.00
30	영아 간호	3.86±0.378	1.00
31	유아 간호	3.86±0.378	1.00
32	학령전기 간호	3.86±0.378	1.00
33	학령기 간호	3.86±0.378	1.00
34	청소년 간호	3.86±0.378	1.00
35	폐경기 여성 간호	3.71±0.488	1.00
36	노인간호	3.86±0.378	1.00
37	성 건강 간호	3.71±0.488	1.00
38	건강력 사정	4.00±0.000	1.00
39	신체검진 수행 및 결과 해석	4.00±0.000	1.00
40	생식기 건강사정	3.71±0.488	1.00
41	태아건강사정 및 간호	3.71±0.488	1.00
42	신생아 건강사정 및 간호	3.86±0.378	1.00
43	고위험신생아 건강사정 및 간호	3.71±0.488	1.00
44	심폐소생술 간호	4.00±0.000	1.00
45	응급간호	4.00±0.000	1.00
46	진단검사 간호	3.86±0.378	1.00
47	수술간호	4.00±0.000	1.00
48	산전간호와 교육	3.86±0.378	1.00
49	분만 중 간호와 교육	3.86±0.378	1.00
50	산후관리와 교육	3.86±0.378	1.00
51	고위험 임부간호	3.71±0.488	1.00
52	고위험 산부간호	3.71±0.488	1.00
53	고위험 산모간호	3.71±0.488	1.00

라) 기본간호

기본간호 영역의 직무항목 내용타당도 지수는 다음과 같다<표 Ⅳ-22>.

<표 Ⅳ-22> 기본간호

번호	항목	Mean±SD	I-CVI
54	체온유지간호	4.00±0.000	1.00
55	세척(irrigation)(방광, 귀, 눈) 수행	3.71±0.488	1.00
56	개인위생간호	4.00±0.000	1.00
57	섭취량 및 배설량 사정과 간호	4.00±0.000	1.00
58	영양사정 및 관리	4.00±0.000	1.00
59	질환별 영양문제의 사정 및 관리	4.00±0.000	1.00
60	섭취장애 대상자 사정 및 간호	3.86±0.378	1.00
61	배뇨장애 대상자 사정 및 간호	3.86±0.378	1.00
62	투석대상자 관리	3.71±0.488	1.00
63	요루 및 장루관리	3.71±0.488	1.00
64	기관절개부 관리	3.86±0.378	1.00
65	배변관리	3.71±0.488	1.00
66	수면과 휴식 간호	3.86±0.378	1.00
67	피부통합성 사정 및 간호	3.86±0.378	1.00
68	이동간호	3.86±0.378	1.00
69	활동과 자기돌봄 장애 사정 및 간호	3.71±0.488	1.00
70	외과적 장치 관리	3.57±0.535	1.00
71	척추손상 및 신경질환 대상자의 간호	3.86±0.378	1.00
72	관절대치술 환자 간호	3.86±0.378	1.00

마) 생리적 통합유지

생리적 통합유지 영역의 직무항목 내용타당도 지수는 다음과 같다<표 Ⅳ-23>.

<표 Ⅳ-23> 생리적 통합유지

번호	항목	Mean±SD	I-CVI
73	호흡기능장애 대상자 간호	3.86±0.378	1.00
74	호흡증진 중재	3.86±0.378	1.00
75	호흡보조장치 관리	3.71±0.488	1.00
76	중심정맥관 관리	3.86±0.378	1.00
77	태아질식 증상과 징후 사정 및 간호	3.71±0.488	1.00
78	고위험 신생아 보육기 적용간호	3.71±0.488	1.00
79	호흡재활관리	3.71±0.488	1.00

번호	항목	Mean±SD	I-CVI
80	심전도 관리	3.86±0.378	1.00
81	순환 보조장치 관리	3.71±0.488	1.00
82	동맥관 관리	3.71±0.488	1.00
83	조직 관류 장애 대상자 사정 및 간호	3.86±0.378	1.00
84	체액 전해질 불균형 사정 및 간호	4.00±0.000	1.00
85	활동 지속성 장애 대상자 사정 및 간호	4.00±0.000	1.00
86	정맥 순환증진 장치 간호	3.86±0.378	1.00
87	혈액 기능장애 대상자 간호	3.71±0.488	1.00
88	순환 기능장애 대상자 간호	3.71±0.488	1.00
89	심장 수술 후 간호	3.86±0.378	1.00
90	소화 기능장애 대상자 간호	3.86±0.378	1.00
91	배뇨장애를 가진 대상자 간호	3.86±0.378	1.00
92	당질 대사장애 대상자 간호	3.86±0.378	1.00
93	당뇨병 합병증 예방을 위한 간호	3.86±0.378	1.00
94	내분비계 장애 대상자 간호	3.86±0.378	1.00
95	생식기 질환/생식기 건강문제를 가진 대상자 간호	3.71±0.488	1.00
96	면역손상 대상자 사정 및 간호	3.71±0.488	1.00
97	감각기능 장애를 가진 대상자 사정 및 간호	3.71±0.488	1.00
98	두개내압 상승 환자의 간호	3.86±0.378	1.00
99	신경계 질환별 간호중재	3.86±0.378	1.00
100	운동기능 장애 간호중재	3.86±0.378	1.00
101	상처간호 수행 및 드레싱교환	3.71±0.488	1.00
102	화상환자 간호중재	3.71±0.488	1.00
103	피부질환 대상자의 간호중재	3.71±0.488	1.00
104	배액장치 관리	3.86±0.378	1.00
105	계통별 신생물 질환 대상자 간호	3.57±0.535	1.00
106	통증간호	4.00±0.000	1.00
107	재활간호서비스	3.71±0.488	1.00
108	(법정) 감염질환자의 전파예방 및 간호	3.86±0.378	1.00

바) 약물 및 비경구요법

약물 및 비경구요법 영역의 직무항목 내용타당도 지수는 다음과 같다<표 Ⅳ-24>.

<표 Ⅳ-24> 약물 및 비경구요법

번호	항목	Mean±SD	I-CVI
109	투약의 적절성과 정확성	4.00±0.000	1.00
110	약물투여에 필요한 계산 시행	4.00±0.000	1.00
111	정맥주입 장치 관리	4.00±0.000	1.00
112	약품관리	4.00±0.000	1.00
113	말초정맥관 삽입, 유지, 제거	4.00±0.000	1.00
114	대상자에게 약물에 대해 교육	3.86±0.378	1.00
115	고위험 약품관리(마약관리)	4.00±0.000	1.00

사) 심리사회적 통합 유지

심리사회적 통합 유지 영역의 직무항목 내용타당도 지수는 다음과 같다<표 Ⅳ-25>.

<표 Ⅳ-25> 심리사회적 통합 유지

번호	항목	Mean±SD	I-CVI
116	이상행동 사정 및 간호	3.86±0.378	1.00
117	폭력의 잠재성 평가 및 예방	3.86±0.378	1.00
118	학대 또는 방임 대상자를 확인하고 적절하게 중재	3.86±0.378	1.00
119	중독 사정 및 간호	3.86±0.378	1.00
120	정신사회건강문제 간호 및 교육	3.86±0.378	1.00
121	정신질환자 간호	3.86±0.378	1.00
122	아동 정신질환자 간호	3.86±0.378	1.00
123	임종간호와 교육	3.86±0.378	1.00
124	치료적 의사소통 기법	4.00±0.000	1.00

아) 건강유지 및 증진

건강유지 및 증진 영역의 직무항목 내용타당도 지수는 다음과 같다<표 Ⅳ-26>.

<표 Ⅳ-26> 건강유지 및 증진

번호	항목	Mean±SD	I-CVI
125	(지역사회)건강교육 계획	3.86±0.378	1.00
126	건강증진 및 유지관리에 대한 정보(예방접종 등)제공	3.86±0.378	1.00
127	고위험 건강행위의 예방 및 치료에 대한 정보(금연, 안전한 성 행위, 바늘교환)제공	3.86±0.378	1.00
128	성 간호 건강증진	3.71±0.488	1.00
129	문화간호	3.71±0.488	1.00
130	산업과 간호 환경	3.71±0.488	1.00
131	재난간호	3.71±0.488	1.00
132	치료계획을 결정하기 위해 가족 역동성(family dynamics) (가족구조, 결속, 의사소통, 경계, 대처기전) 사정	3.71±0.488	1.00
133	가정환경에서 대상자를 관리할 수 있는 역량(장비, 지역사회 자원) 평가	3.57±0.535	1.00

134	건강위험요인 사정과 교육	4.00±0.000	1.00

2012년부터 국가시험 출제기준 개발을 위해 단계별로 순차적으로 진행된 연구를 통하여 도출된 신규간호사 직무기반 통합형 국가시험 출제모형의 주개념은 직무영역(대분류), 직무항목(중분류), 표준 학습목표, 지식항목(소분류)이다. 신규간호사 직무항목은 직무영역, 표준학습목표, 지식항목과 연계되어 있으므로, 우선적으로 출제모형의 주개념인 직무항목의 시의성을 확인하기 위하여 타당성과 적절성 평가를 실시하였다. 설문조사, 전문가 인터뷰 및 내용타당도 조사에서 직무항목의 타당성과 적절성이 확인되어, 연구진 합의하에 직무항목의 용어만 일관성을 유지할 수 있도록 간결하게 정리하고 134개 직무항목은 출제모형에 반영하기로 하였다.
