# Supplementary material for: Suggestion for item allocation to 8 nursing activity categories of the Korean Nursing Licensing Examination: a survey-based descriptive study
Source: J Educ Eval Health Prof. 2023 Jun 12;20:18. doi: 10.3352/jeehp.2023.20.18 (PMC10352010; doi:10.3352/jeehp.2023.20.18)
Supplement: Supplementary file 3 — Supplement 2. Source report for this article. [file jeehp-20-18-suppl2.pdf]

RE02-2111-05

간호사 국가시험의 통합모형에 근거한 출제기준개발 기초연구

2021

책임연구자
김경희 (중앙대학교)

한국보건의료인국가시험원

RE02-2111-05

간호사 국가시험의 통합모형에 근거한 출제기준개발 기초연구

2020. 09.01 ~ 2021. 06. 25

**책임 연구자**	김 경 희 (중앙대학교)
**공동 연구자**	강 소 영 (부산가톨릭대학교)
	강 윤 희 (이화여자대학교)
	권 영 란 (전남대학교)
	김 현 정 (한림대학교)
	송 영 신 (충남대학교)
	조 주 연 (수원과학대학교)
	최 미 영 (충북대학교)
**연구 보조원**	이 현 수 (한국성서대학교)

본 연구는 한국보건의료인국가시험원(이하 국시원)의 연구비로 수행되었으나, 여기서 제시된 내용은 국시원의 공식적인 의견이 아니며, 연구진들의 연구결과물임을 알려드리는 바입니다.

간호사 국가시험의 통합모형에 근거한 출제기준개발 기초연구 2021

김경희

차 례

I. 서론 ······ 1
1. 연구배경 및 필요성 ······ 1
2. 연구목적 ······ 3

II. 연구내용 및 방법 ······ 4
1. 연구내용 ······ 4
가. 간호사 국가시험 관련 국내외 문헌고찰 ······ 4
나. 간호사 국가시험 출제모형 제시 ······ 4
다. 간호사 국가시험 출제모형에 근거한 출제기준(안) 제시 ······ 5
라. 간호사 국가시험 출제기준 최종(안) 도출 ······ 5
2. 연구방법 ······ 6
가. 국내외 문헌고찰 분석 및 정리 ······ 6
나. 설문조사, 전문가 인터뷰 및 내용타당도 조사, 유관 단체 의견조사 ······ 6
다. 유관 단체 전문가 의견조사 ······ 8
라. 공청회를 통한 전문가 의견수렴 ······ 8

III. 문헌고찰 ······ 9
1. 국내 간호사 국가시험 관련 문헌고찰 ······ 9
가. 간호사 2차 직무분석 연구 ······ 9
나. 간호사 직무분석, 학습목표, 국가시험 연계성 분석연구 ······ 11
다. 간호대학 졸업자의 최소직무능력규정 및 국가시험 적용방안 연구 ······ 11
라. 직무기반 간호사 국가시험을 위한 문항개발기준 및 문항개발절차 개선 연구 ·· 12
마. 간호사 국가시험 통합출제를 위한 모형구축 ······ 13
바. 보건의료환경 변화에 따른 간호사 국가시험 및 면허관리체계 개선방안 ······ 35

2. 국외 간호사 국가시험 관련 문헌고찰 ··· 39
가. 미국과 캐나다 ··· 39
나. 일본 ··· 48

Ⅳ. 연구결과 ··· 50
1. 간호사 국가시험 국내외 문헌고찰 ··· 50
가. 국내 간호사 국가시험 관련 문헌고찰 ··· 50
나. 국외 간호사 국가시험 관련 문헌고찰 ··· 50
2. 간호사 국가시험 출제모형 제시 ··· 54
가. 출제모형의 간호사 직무항목에 대한 타당성과 적절성 평가 ··· 54
나. 출제모형의 간호사 직무항목에 대한 전문가 인터뷰 및 타당도 조사 ··· 88
다. 간호사 국가시험 출제모형에 대한 유관 단체 전문가 의견 조사 ··· 95
라. 직무기반 통합형 간호사 국가시험 출제모형 제시 ··· 103
3. 간호사 국가시험 출제모형에 근거한 출제기준(안) 제시 ··· 105
가. 직무영역별 국가시험 출제비율에 대한 유관 단체 전문가 의견조사 ··· 105
나. 직무영역별 국가시험 출제비율에 대한 전문가 타당도 조사 ··· 108
다. 간호사 국가시험 출제모형에 근거한 출제기준(안) 제시 ··· 108
4. 간호사 국가시험 출제기준 최종안 도출 및 전문가 의견수렴 ··· 111
가. 공청회 개최 ··· 111

Ⅴ. 결론 및 제언 ··· 116

참고문헌 ··· 119

【부록1】 간호사 직무분석 관련 설문조사지 ···································· 121
【부록2】 전문가 타당도 평가 설문지 ·· 132
【부록3】 간호사 국가시험 출제모형에 대한 7개 회원학회 1,2차 의견······································144
【부록4】 간호사 국가시험 출제모형에 대한 간호행정학회 의견 ······································177
【부록5】 간호사 국가시험 출제모형에 대한 여성건강간호학회 의견 ··································184
【부록6】 간호사 국가시험 출제모형에 대한 아동간호학회 의견 ······································190
【부록7】 간호사 국가시험 출제모형에 대한 지역사회간호학회 의견 ··································192
【부록8】 간호사 국가시험 출제모형에 대한 7개 회원학회 의견 ··································201
【부록9】 공청회에서 제기된 전문가 의견 ··202
【부록10】 한국간호과학회 7개 회원학회 1차 의견조사 ····································209
【부록11】 한국간호과학회 7개 회원학회 2차 의견조사 ···································· 211
【부록12】 한국간호과학회, 한국간호대학(과)장협의회, 한국전문간호대학(부)장 협의회, 병원간호사회 의견조사 ·· 213

표 차 례

<표 III-1> 간호사 2차 직무분석에서 도출된 간호사 직무 (박인숙 외, 2012) ··············· 10
<표 III-2> 간호사 국가시험 통합모형에 따른 실무항목과 지식항목 ······························ 14
<표 III-3> 실무항목별 행위지식 수의 교과목별 분포 (송라윤 외, 2018) ························ 31
<표 III-4> 각 과목별 누락된 학습목표 개수 (송라윤 외, 2018) ······································ 32
<표 III-5> 각 과목별 누락된 학습목표 (송라윤 외, 2018) ·· 32
<표 III-6> 선행연구의 직무영역별 비중 차이 비교 ·· 35
<표 III-7> 간호사 직무 영역별 지식항목의 배정표 (탁영란 외, 2019) ··························· 37
<표 III-8> 직무영역별 배정 교육목표에 근거한 영역별 총 가중치 (탁영란 외, 2019) ·· 37
<표 III-9> 직무영역에 따른 실무항목과 행위지식 분포에 기반한 최종 가중치. 간호사 국가시험 출제기준표 (탁영란 외, 2019) ··· 39
<표 III-10> NCLEX-RN 문항출제의 직무분석 분류와 출제 비율 ···································· 40
<표 III-11> NCLEX-RN Knowledge statements (NCSBN, 2018) ································· 41
<표 IV-1> 간호사 국가시험 출제모형 개발을 위한 선행연구 ··· 52
<표 IV-2> 대상자의 인구사회학적 특성 ·· 55
<표 IV-3> 간호관리와 전문성 향상 ·· 56
<표 IV-4> 안전과 감염관리 ·· 58
<표 IV-5> 위험요인 사정 ·· 60
<표 IV-6> 기본간호 ·· 62
<표 IV-7> 생리적 통합유지 ·· 64
<표 IV-8> 약물 및 비경구요법 ··· 67
<표 IV-9> 심리사회적 통합 유지 ·· 69
<표 IV-10> 건강유지 및 증진 ··· 71
<표 IV-11> 간호관리와 전문성 향상 ·· 75
<표 IV-12> 안전과 감염관리 ·· 77
<표 IV-13> 위험요인 사정 ·· 78
<표 IV-14> 기본간호 ·· 80
<표 IV-15> 생리적 통합유지 ·· 81

<표 Ⅳ-16> 약물 및 비경구요법 ··· 83
<표 Ⅳ-17> 심리사회적 통합 유지 ··· 84
<표 Ⅳ-18> 건강유지 및 증진 ··· 86
<표 Ⅳ-19> 간호관리와 전문성 향상 ··· 89
<표 Ⅳ-20> 안전과 감염관리 ··· 90
<표 Ⅳ-21> 위험요인 사정 ··· 91
<표 Ⅳ-22> 기본간호 ··· 92
<표 Ⅳ-23> 생리적 통합유지 ··· 92
<표 Ⅳ-24> 약물 및 비경구요법 ··· 94
<표 Ⅳ-25> 심리사회적 통합 유지 ··· 94
<표 Ⅳ-26> 건강유지 및 증진 ··· 95
<표 Ⅳ-27> 지식항목의 교과목 배정에 대한 선행연구와 7개 학회 의견 비교 ··· 99
<표 Ⅳ-28> 134개 신규간호사 직무항목 용어정리 ··· 100
<표 Ⅳ-29> 직무영역별 국가시험 출제비율에 대한 7개 회원학회 의견조사 ··· 107
<표 Ⅳ-30> 간호사 국가시험 출제모형에 근거한 출제기준(안) ··· 111
<표 Ⅳ-31> 공청회 프로그램 ··· 112

그 림 차 례

[그림 II-1] 신규간호사 직무역량 평가를 위한 국가시험 통합모형 ······ 7
[그림 II-2] 국가시험 교과목과 신규간호사 실무항목의 연관성 ······ 7
[그림 Ⅳ-1] 직무기반 통합형 간호사 국가시험 출제모형 개발과정 ······ 51
[그림 Ⅳ-2] 간호관리와 전문성 향상 직무의 IPA ······ 58
[그림 Ⅳ-3] 안전과 감염관리 직무의 IPA ······ 59
[그림 IV-4] 위험요인 사정 직무의 IPA ······ 61
[그림 IV-5] 기본간호 직무의 IPA ······ 63
[그림 IV-6] 생리적 통합유지 직무의 IPA ······ 66
[그림 IV-7] 약물 및 비경구요법 직무의 IPA ······ 68
[그림 IV-8] 심리사회적 통합 유지 직무의 IPA ······ 70
[그림 IV-9] 건강유지 및 증진 직무의 IPA ······ 72
[그림 IV-10] 간호사 직무의 IPA ······ 74
[그림 Ⅳ-11] 간호사 국가시험 출제모형 ······ 103
[그림 Ⅳ-12] 출제모형에 근거한 출제기준(안) 개발과정 ······ 109

I. 서론

1. 연구 배경 및 필요성

간호사 국가시험은 간호대학 졸업자가 간호 실무현장에서 최신의 신규간호사 직무에 기반한 직무를 수행하는데 필요한 최소한의 능력을 평가하는 대표적인 준거참조평가(Criterion referenced assessment)이다(김영경 외, 2018).

현재 우리나라 간호사 면허 취득을 위한 간호사 국가시험은 의료법 시행규칙 2조(별표1의3)에 따라 기본간호학, 성인간호학, 모성간호학, 아동간호학, 지역사회간호학, 정신간호학, 간호관리학, 보건의약관계 법규 등 8개 간호학 전공교과목 필기시험을 실시하여 전 과목 60% 이상, 매 과목 40% 이상 득점한 자를 합격자로 한다.

간호사 국가시험 교과목의 교육내용을 비교 분석한 박호란 외(2011)는 7개 간호학 전공교과목의 학습목표와 평가문항에서 심각한 수준의 중복이 있음을 확인하였다. 이러한 교과목 간의 중복성은 간호사 국가시험 응시자, 문항개발자, 시험출제자 등 모든 이해관계자들에게 부담이 되며, 간호사 국가시험의 효율성을 저하시키는 요인이 되고 있다.

이와 같이 교과목 간에 중복성이 확인된 간호학 전공교과목의 학습목표를 출제기준으로 하는 간호사 국가시험이 간호대학에서 졸업예정자를 대상으로 시행하는 졸업시험과 유사하여, 준거참조평가로서 기대되는 간호사 국가시험이 간호사의 직무능력을 적절하게 평가하고 있는지에 대한 비판이 꾸준히 제기되어 왔다(송라윤 외, 2018).

이에 간호사 국가시험의 패러다임이 간호학 전공교과목의 지식검증에서 최신의 신규간호사 직무에 기반한 직무기반 지식검증으로 전환되어야 할 필요성이 제기되면서, 간호사 국가시험이 교과목 간 중복성을 해결하면서 직무기반 지식검증을 할 수 있도록 통합된 방안을 모색하기 위한 연구가 끊임없이 진행되어왔다.

먼저, 박인숙 외(2012)는 다변화 되고 있는 간호 실무현장의 간호사 직무에 대한 명확한 개념과 직무의 범위를 규명하기 위해 ‘간호사 2차 직무분석’을 실시하였다. 이어서 간호사 국가시험 교과목의 통합 가능성을 확인하기 위하여 ‘국가시험 과목 제도 개선에 관한 연구(김금순 외, 2013)’가 시행되었고, 이후 ‘간호사 직무분석, 학습목표, 국가시험 연계성 분석 연구(박인숙 외, 2014)’를 통해 직무분석 결과와 학습목표를 국가시험에 반영하기 위한 노력이 있었으며, 후속 연구로 ’간호대학 졸업자의 최소직무능력 규정 및 국가시험 적용방안 연구(강소영 외, 2015)‘는 국가시험을 통해 평가해야 하는 최소직무능력의 근거를 제공함으로써 직무기반 준거참조평가의 틀을 마련하였다

(김영경 외, 2018).

간호사 국가시험 관련 선행연구 결과를 기반으로 한 김영경 외(2018)의 ‘직무기반 간호사 국가시험을 위한 문항개발 기준 및 문항개발 절차 개선연구’에서는 간호사 직무와 국가시험 연계의 타당성을 검토하였고, 송라윤 외(2018)의 ‘간호사 국가시험 통합출제를 위한 모형구축’ 연구에서는 선행연구에서 개발된 간호관리와 전문성 향상, 안전과 감염관리, 잠재적 위험요인 관리, 기본간호, 생리적 통합유지, 약물 및 비경구요법, 심리사회적 통합유지, 건강증진 및 유지 등 8개 신규간호사 직무영역에 신규간호사 직무항목(Activity Statement: AS) 134개와 전공교과목의 학습목표에서 추출한 표준 학습목표를 연계하여 국가시험 문항개발의 기준이 되는 481개 범주의 1,303개 지식항목(knowledge statement: KS)을 도출하여 간호사 국가시험 통합출제를 위한 통합모형을 제안하였다.

송라윤 외(2018)의 연구를 기반으로, 탁영란 외(2019)는 간호사 국가시험의 출제기준(안)을 제시하기 위해서 8개 직무영역에 134개 직무항목과 481개 범주의 1,303개 지식항목을 연계하여 교과목별로 가중치를 산출하여, 현행 간호사 국가시험 295개 문제 수에 연구에서 산출한 가중치를 적용하여 8개 직무영역별 출제 문제 수와 출제기준표 초안을 제시하였다.

이상에서 살펴본 바와 같이 지금까지 진행되어 온 선행연구를 기반으로 간호사 국가시험 출제모형에 근거한 출제기준을 개발하기 위해서는 우선적으로, 선행연구에서 제시하고 있는 신규간호사 134개의 직무항목이 지속적으로 변화하는 간호 실무현장에서 실제적으로 신규간호사가 수행하는 최신의 신규간호사 직무를 반영하고 있는지가 규명되어야 한다.

이에 본 연구는 간호 실무현장의 간호사를 대상으로, 기존의 연구에서 개발된 134개의 간호사 직무항목이 최신의 신규 간호사 직무를 반영하고 있는지 확인하여, 신규간호사 직무가 반영된 출제기준안을 출제모형에 근거하여 8개 직무영역별로 개발함으로써 간호사 국가시험의 패러다임이 직무기반 지식검증으로 전환될 수 있는 기초자료를 제공하고자 한다.

2. 연구목적

본 연구의 목적은 간호 실무현장의 간호사를 대상으로, 기존의 연구에서 개발된 134개의 간호사 직무항목이 최신의 신규간호사 직무를 반영하고 있는지 확인하여, 신규간호사 직무가 반영된 출제기준(안)을 국가시험 출제모형에 근거하여 8개 직무영역별로 개발함으로써 간호사 국가시험의 패러다임이 직무기반 지식검증으로 전환될 수 있는 기초자료를 제공하기 위함이다. 이를 위한 구체적인 목적은 다음과 같다.

1) 국내외 간호사 국가시험 관련 문헌을 고찰한다.
2) 문헌고찰에 기반한 간호사 국가시험 출제모형을 제시한다.
3) 간호사 국가시험 출제모형에 근거한 출제기준(안)을 제시한다.
4) 전문가 의견수렴 및 간호사 국가시험 출제기준 최종(안)을 도출한다.

II. 연구내용 및 방법

1. 연구내용

신규간호사 직무가 반영된 출제기준(안)을 간호사 국가시험 출제모형에 근거하여 8개 직무영역별로 개발하기 위하여 다음의 세부 목적에 따른 연구내용은 다음과 같다.

가. 간호사 국가시험 관련 국내외 문헌고찰

1) **목표** : 국내외 간호사 국가시험 관련 문헌을 고찰한다.

2) **내용**

- 국내 간호사 국가시험 관련 문헌을 고찰한다.
- 국외 간호사 국가시험 관련 문헌을 고찰한다.

나. 간호사 국가시험 출제모형 제시

1) **목표** : 신규간호사의 역량을 평가할 수 있는 간호사 국가시험 출제기준의 근거가 되는 출제모형을 제시한다.

2) **내용**

- 간호사 국가시험 출제모형의 8개 직무영역과 직무영역에 연계된 134개 간호사 직무항목의 시의성, 타당성, 적절성 평가를 위해 임상 및 지역사회 간호 실무 현장에서 근무하는 간호사를 대상으로 설문조사를 실시하여 결과를 분석한다.
- 간호사 국가시험 출제모형의 8개 직무영역에 연계된 134개 간호사 직무항목에 대해 임상 및 지역사회 간호 실무현장 전문가를 대상으로 설문조사 분석 결과에 대한 인터뷰를 실시하고 134개 간호사 직무항목에 대한 내용타당도를 조사한다.
- 출제모형의 시험문제로의 개발가능성과 평가실행 가능성에 대한 타당성과 적절성을 전문가 집단을 대상으로 의견조사를 실시한다.
- 문헌고찰, 설문조사 결과, 전문가 대상의 인터뷰 및 내용타당도 조사 결과, 전문가 집단을 대상으로 시험문제로의 개발가능성과 평가실행 가능성에 대한 타당성과 적절성 조사 결과를 근거로 간호사 국가시험 출제모형을 제시한다.

다. 간호사국가시험 출제모형에 근거한 출제기준(안) 제시

1) **목표** : 간호사 국가시험 출제모형에 근거하여 신규간호사의 직무역량을 평가하는 출제기준(안)을 제시한다.

2) **내용**

- 간호사 국가시험 출제모형의 8개 직무영역(대분류), 134개 직무항목(중분류), 481개 범주의 지식항목(소분류)에 대한 검토와 더불어 국가시험 교과목의 8개 직무영역별 출제 비율에 대해 교육전문가와 임상 및 지역사회 간호 실무현장 전문가 집단을 대상으로 의견조사를 실시한다.
- 교육전문가와 임상 및 지역사회 간호 실무현장 전문가 집단의 의견조사 결과를 기반으로 간호사 국가시험 출제모형에 근거한 출제기준(안)을 제시한다.

라. 전문가 의견수렴 및 간호사 국가시험 출제기준 최종(안) 도출

1) **목표** : 교육전문가와 임상 및 지역사회 간호 실무현장 전문가 집단을 대상으로 의견수렴 과정과 합의를 거쳐서 간호사 국가시험 출제기준 최종(안)을 도출한다.

2) **내용**

- 전국 간호대학 교수와 임상 및 지역사회 간호 실무현장 전문가를 대상으로 공청회를 개최하여 교육전문가와 임상 및 지역사회 간호 실무현장 전문가 집단의 의견조사 결과를 기반으로 연구진 합의 하에 도출한 간호사 국가시험 출제모형에 근거한 출제기준(안)에 대해 설명하고 이에 대한 의견을 수렴하여 합의를 도출한다.
- 전국 간호대학 교수와 임상 및 지역사회 간호 실무현장 전문가를 대상으로 설명회를 개최하여 간호사 국가시험 출제모형에 근거한 간호사 국가시험 출제기준 최종(안)을 설명한다.

2. 연구방법

가. 국내외 문헌고찰 분석 및 정리

간호사 국가시험 출제기준(안)의 근거가 되는 출제모형을 확인하기 위해 국내외 간호사 국가시험 관련 문헌을 고찰하였다. 현행 국내 간호사 국가시험 출제기준, 미국 간호사 국가시험, 국내외 의료인 국가시험 출제기준 개발과 관련된 문헌을 고찰하고 분석한 내용을 정리하였다.

나. 설문조사, 전문가 인터뷰 및 내용타당도 조사, 유관 단체 의견조사

선행연구에서 개발된 간호사 국가시험 통합모형 [그림 Ⅱ-1], [그림 Ⅱ-2]의 8개 직무영역에 연계된 134개 신규간호사 직무항목의 시의성, 타당성, 적절성 평가를 위해 임상 및 지역사회 간호 실무현장에서 근무하는 간호사 260명을 대상으로 134개 신규간호사 직무항목에 대한 수행도, 중요도 및 개선 요구도에 관한 설문조사를 실시하였다. 임상 및 지역사회 간호 실무현장 전문가 7명과의 인터뷰를 통해 설문조사의 IPA(Importance Performance Analysis, IPA)자료 분석 결과에 대한 의견을 듣고, 134개 신규간호사 직무항목에 대해 '매우 타당하지 않다' 1점에서 '매우 타당하다' 4점 척도로 내용타당도 I-CVI(Item -level Content Validity Index)를 확인하였다. 문헌고찰, 설문조사 결과, 임상 및 지역사회 간호 실무현장 전문가 인터뷰 및 내용타당도 조사 결과에 근거하여 연구진의 합의에 의해 국내 신규간호사 직무기반 통합형 간호사 국가시험 출제모형을 확인하였다. 간호사 국가시험 출제모형의 8개 직무영역과 직무영역에 연계된 134개 직무항목, 직무항목과 연계하여 간호학 전공교과목의 표준 학습목표에서 도출된 481개 범주의 지식항목에 대해 시험문제로의 개발가능성과 평가 실행 가능성에 대한 타당성과 적절성을 유관 단체를 대상으로 의견 조사를 실시 하였다. 유관 단체를 대상으로 한 타당성과 적절성에 대한 의견조사는 한국간호과학회 7개 회원학회(이하, '7개 회원학회'라 한다)를 대상으로 1차, 2차에 걸쳐서 진행하였다. 7개 회원학회에서 제시한 의견은 다시 한국간호과학회, 한국간호대학(과)장협의회, 한국간호전문대학(부)장협의회, 병원간호사회 등 유관 단체에 검토를 요청하면서 이에 대한 의견조사를 실시하였다.

국가시험 관련 문헌고찰, 출제모형의 직무항목에 대한 타당성과 적절성 평가결과, 임상실무 전문가 인터뷰 및 내용타당도 조사결과, 유관 단체를 대상으로 출제모형의

타당도와 적절성에 대한 의견조사 결과를 기반으로 신규간호사 직무기반 통합형 간호사 국가시험 출제모형을 연구진 합의하에 제시하였다.

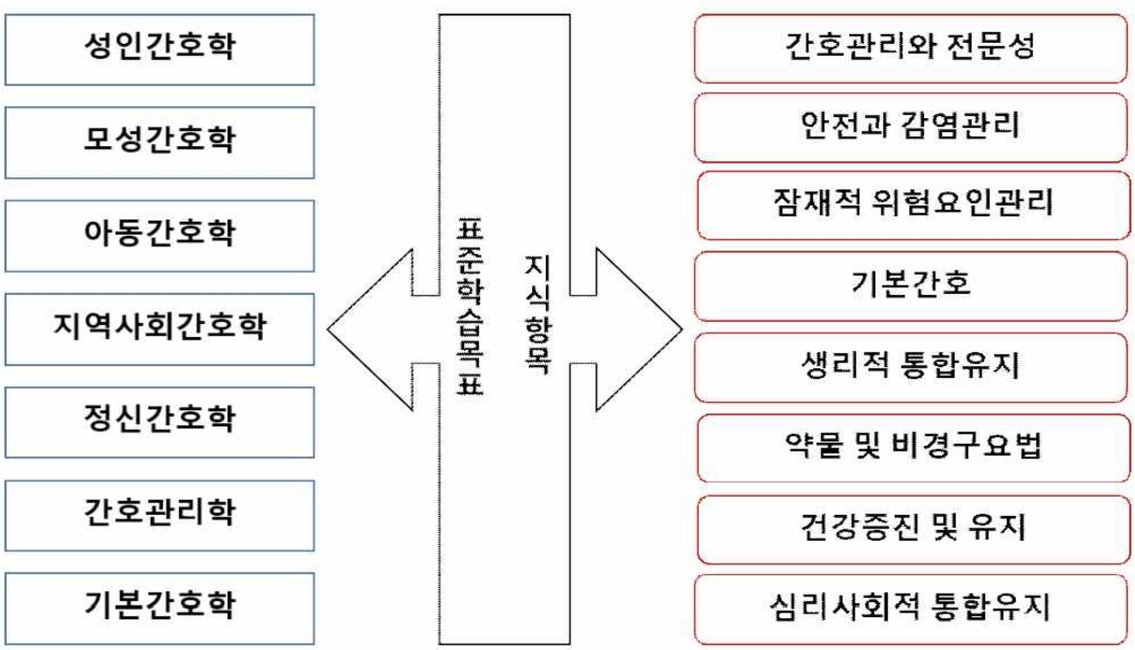

[그림 II-1] 신규간호사 직무역량 평가를 위한 국가시험 통합모형 (출처: 송라윤 등, 2018, p.19)

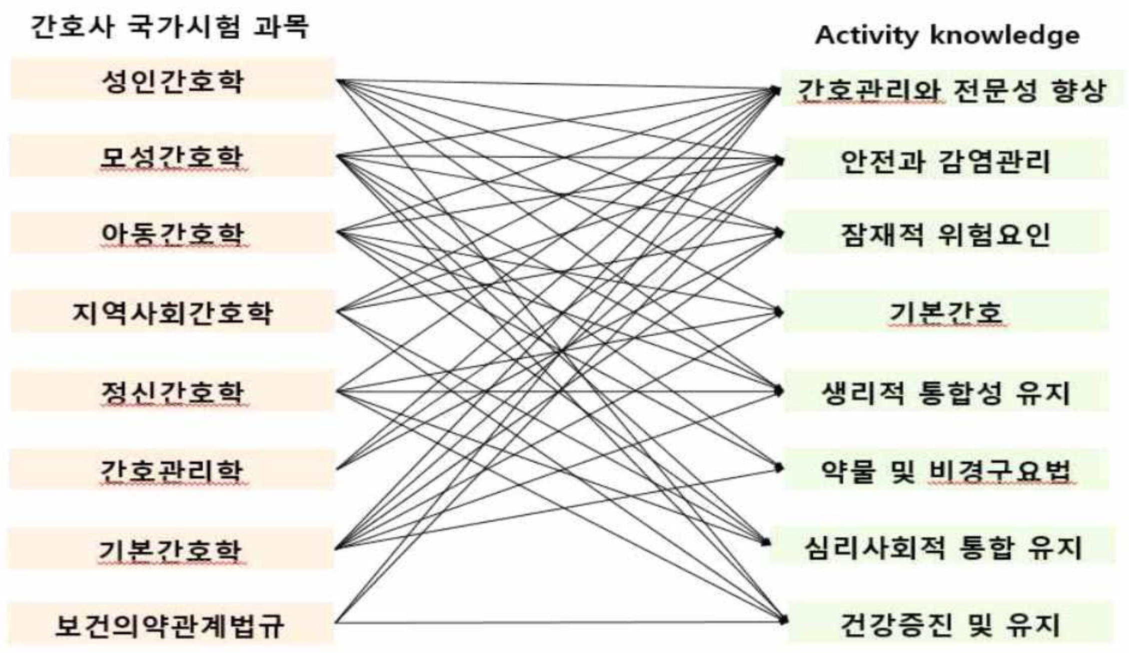

[그림 II-2] 국가시험 교과목과 신규간호사 직무항목의 연관성 (출처: 송라윤 등, 2018, p.49)

다. 유관 단체 전문가 의견조사

코로나 19로 대면으로 워크숍을 개최할 수 없는 상황에서 전문가 집단의 다양한 의견을 들을 수 있는 대안으로, 유관 단체를 대상으로 의견조사를 실시하였다. 7개 회원학회를 대상으로 본 연구진 합의 하에 제시 한 직무기반 통합형 간호사 국가시험 출제모형에 근거한 출제기준안의 8개 직무영역(대분류), 134개 직무항목(중분류), 481개 범주의 지식항목(소분류)에 대한 검토와 8개 직무영역의 영역별 국가시험 교과목의 출제 비율에 대한 의견을 1차 조사하였고, 7개 회원학회의 1차 조사 결과를 공유하면서 2차 조사를 실시하였다. 한국간호과학회, 한국간호대학(과)장협의회, 한국전문대간호학(부)장협의회, 병원간호사회를 대상으로 7개 회원학회에서 제시한 1차, 2차 의견 조사 결과를 공유하면서 간호사 국가시험 출제기준안의 8개 직무영역(대분류), 134개 직무항목(중분류), 481개 범주의 지식항목(소분류) 에 대한 검토와 8개 직무영역의 영역별 출제비율의 타당성과 적절성에 대한 의견을 조사하였다. 연구진 합의하에 설문조사, 전문가 인터뷰 및 내용타당도 조사, 유관 단체 의견조사 결과에 기반하여 8개 직무영역별 국가시험 출제비율을 제시하였다.

라. 공청회를 통한 전문가 의견수렴

전국 간호대학 교수, 임상 및 지역사회 간호 실무현장 전문가를 대상으로 공청회를 개최하여 직무기반 통합형 간호사 국가시험 출제모형에 근거한 출제기준(안)에 대한 의견을 듣고, 향후 출제기준 개정작업에 반영할 수 있도록 교육전문가, 임상 및 지역사회 간호 실무현장 전문가가 제시한 의견을 정리하였다.

III. 문헌고찰

1. 국내 간호사 국가시험 관련 문헌고찰

간호사 국가시험은 자격을 부여하기 위한 준거참조평가를 근간으로 하는 시험이며, 신규간호사의 역량을 준거로 한다. 따라서 간호사 국가시험은 신규간호사가 갖추어야 할 최소역량을 평가하는 것을 목표로 신규간호사의 최소직무능력을 규정하고 직무항목을 시험으로 평가하는 것이 얼마나 타당한가를 검토한 후 최종적인 직무기반 Test plan을 개발하는 것이 필요하다. 이러한 목표를 가지고 한국보건의료인국가시험원은 지난 10년간 간호사 국가시험의 기본구성을 현장에서 간호사가 수행하는 직무중심으로 전환하기 위해 지속적인 연구를 추진해왔다.

가. 간호사 2차 직무분석 연구 (한국보건의료인국가시험원, 박인숙 외, 2012)

2차 간호사 직무분석은 현재 보건의료현장에서 요구하는 기본직무와 문제해결능력, 종합적 사고력 등을 평가하고 검증할 수 있는 국가시험제도를 준비하기 위해 현장직무에 대한 명확한 개념과 직무의 범위를 규명하기 위해 시행되었다.

즉 간호사가 수행하는 직무의 내용을 분석하고 범주화하였으며, 각각의 직무에 대한 일의 요소와 직무수행에 필요한 지식, 기술, 태도를 기술한 직무명세서를 작성하였다.

연구진행방법은 직무분석에 가장 적합한 DACUM 방법으로 시행하였다.

1단계는 선행연구 고찰 및 비교분석 후 현장에서의 간호업무와 관련된 간호사의 경험을 확인하기 위해 포커스 그룹 인터뷰를 실시하였다. 면담 자료에서 '현재 간호사로서의 업무'와 관련된 진술을 추출하였고, 추출내용을 상위 범주화하여 '간호술기의 적용', '다양한 대상자 간호', '정보제공과 교육', '관찰과 모니터링', '동료업무 지원 활동', '관리 활동', '학습과 연구', '교류와 자문'의 8개 범주로 구분하였다.

2단계는 DACUM 방법으로 간호사의 경험과 직무를 설명해 줄 수 있는 현장실무전문가에 의해 도출된 직무를 교육전문가와 연구위원회의 회의를 통해 유사한 직무는 통합하고, 중요한 일(task)의 속성은 분리하여 제시, 동일한 직무는 포괄적인 직무를 나타내는 명칭으로 수정 보완하였다.

각 단계에 적합한 직무를 검토하는 과정에서 중점을 둔 것은 실제적인 간호활동(activity) 내용을 반영하고자 하였고, 실무전문가와 이론적 근거를 고려하여 49개의

일(task)에 대해 선행연구 결과와 미국 NCLEX-RN의 분류기준을 참고하여 8개의 임무(duty)로 분류하였다. 각각의 일(task)에 대한 직무명세서는 도출된 일의 요소(task element)를 포함시켜 작성하였고, 일의 요소를 충족시키기 위한 지식(knowledge), 기술(skill), 태도(attitude)를 기술하였다.

3단계는 DACUM 방법으로 도출된 직무기술서를 전국 간호사를 대상으로 설문조사하였다. 즉 <간호사직무분석>의 임무내용별 중요도, 난이도, 타당도를 조사하였다.

2차 간호사 직무분석 연구결과 신규간호사를 포함한 간호사의 최종 직무내용은 8개의 임무(duty), 49개의 일(task), 303개의 일의 요소(task element)로 구성되었다<표 III-1>.

<표 III-1> 간호사 2차 직무분석에서 도출된 간호사 직무 (박인숙 외, 2012)

8개 임무(Duty)	49개 일(Task)
A. 간호관리와 전문성 향상	A1 인수인계하기
	A2 간호기록하기
	A3 처방전 관리하기
	A4 입퇴원 관리하기
	A5 물품 및 장비 관리하기
	A6 문서 작성 및 보고하기
	A8 간호의 질 향상에 참여하기
	A9 전문직 역량 강화하기
	A10 보건의료서비스자원 활용하기
	A11 보건의료팀과 협력하기
B. 안전과 감염관리	B1 환경관리하기
	B2 감염관리하기
	B3 손상 및 사고 예방, 관리하기
	B4 재해대응하기
C. 잠재적 위험요인 관리	C1 대상자 상태 모니터링 하기
	C2 응급상황 대처하기
	C3 검사 전•중•후 간호하기
	C4 수술 전•중•후 간호하기
	C5 출산 전•중•후 간호하기
D. 기본간호	D1 개인위생 제공하기
	D2 영양관리하기
	D3 배뇨관리하기
	D4 배변관리하기
	D5 수면 및 휴식 제공하기
	D6 욕창예방 및 관리하기
	D7 활동 유지 간호하기
E. 생리적 통합 유지	E1 호흡 유지 간호하기
	E2 순환 유지 간호하기
	E3 조절과 대사 유지하기
	E4 감각 유지 간호하기
	E5 상처 관리하기
	E6 배액관 관리하기

	E7 통증 관리하기
F. 약물 및 비경구 요법	F1 투약하기
	F2 약품관리하기
	F3 수혈 간호하기
	F4 항암요법 환자 관리하기
	F5 비경구적 영양요법 관리하기
G. 심리사회적 통합 유지	G1 대상자 및 가족 지지하기
	G2 호스피스 간호하기
	G3 삶의 가치와 신념 유지 돕기
	G4 스트레스 관리하기
	G5 위기 관리하기
	G6 물질남용•의존 관리하기
H. 건강증진 및 유지	H1 교육하기
	H2 건강상태 파악하기
	H3 건강위험요소 확인하기
	H4 성과 생식건강 유지하기

나. 간호사 직무분석, 학습목표, 국가시험 연계성 분석연구 (박인숙 외, 2014)

박인숙 외(2014)는 간호사의 직무분석 결과(박인숙 외, 2012)를 토대로 간호학 학습목표(한국간호과학회, 2012), 직무별 국가시험 과목별 출제범위(2016년)를 비교분석함으로써 간호사 직무-학습목표/국가시험 출제범위가 서로 상호 연계성이 있다는 결과를 도출하였다. 즉 2차 직무분석에서 도출된 8개 간호임무(duty)를 출제영역으로, 49개 일(task)을 세부영역으로, 학습목표/국가시험 출제범위에서 도출된 308개의 간호사 지식을 출제내용으로 하는 직무중심 간호사 국가시험의 출제기준안을 제안하였다. 그러나 직무별 간호지식 분석에 관한 추가연구가 필요하며, 전공 간 학습목표나 출제범위 분류의 일관성 부족을 제한점으로 제시하였다.

다. 간호대학 졸업자의 최소직무능력규정 및 국가시험 적용방안 연구 (강소영 외, 2015)

강소영 외(2015)는 선행연구 결과(박인숙 외, 2012, 박인숙 외, 2010)를 기초자료로 활용하여 신규간호사의 직무 중에서 국가시험에서 평가해야 하는 간호대학 졸업자의 최소직무능력을 규정하였고, 직무와 학습목표와의 연계성을 분석함으로써 국가시험에서의 평가목표의 근간을 마련하였다.

신규간호사의 최소직무역량을 규명한 것은 간호대학 교육과정에서 학습한 내용을 국가시험에서 모두 평가해야 하는 것이 아니라는 것을 전제로 간호사직무역량(nursing practice competency, NPC)기반 국가시험의 시작점을 마련하였다. 즉 간

호대학 졸업자의 최소직무능력안의 8개 직무영역을 출제범위로, 49개 직무를 출제영역으로, 최소직무능력안의 직무목표를 출제범위별 출제준거로, 직무기준을 출제표준으로, 그리고 372개 직무능력내용을 출제내용으로 적용할 것을 제안하였다.

이로서 임상현장에서의 간호사 직무와 교육간, 실무와 국가시험간의 괴리를 연결할 수 있는 가능성을 보여주었다. 그러나 최소직무능력안의 틀이 2010년과 2012년 직무분석 결과로 현재 시점의 간호사 직무를 반영하고 있다고 간주하기 어렵고, 직무영역-직무 분류와 정의에 대한 타당성 재검토가 필요함을 제한점으로 제시하였다.

라. 직무기반 간호사 국가시험을 위한 문항개발기준 및 문항개발절차 개선 연구 (김영경 외, 2018)

김영경 외(2018)는 현재의 의료환경의 변화가 반영된 직무기반 문항개발기준 및 문항개발절차(안)을 제시하기 위하여 Activity statement를 도출하였다.

미국의 간호사 국가시험을 주관하고 있는 The National Council of State Board Nursing(NCSBN)에서는 3년마다 신규간호사를 대상으로 직무분석 연구를 시행하여 신규간호사가 기본적으로 수행해야 하는 직무를 간호활동(nursing activity)으로 제시하고 Activity statement로 명명하여 평가기준으로 활용하고 있다. 따라서 연구진은 2015년 NCSBN의 Activity statement 140개와 박인숙 등(2012)의 직무분석 결과 도출된 일(task) 49개, 박인숙 등(2014)의 직무분석-간호지식 연계 출제내용 308개, 강소영 등(2015)의 최소직무능력내용 372개를 검토 및 분석하였다. 또한 국가시험 출제경험이 있는 간호대학 교수 및 임상간호사 20명을 대상으로 현재 출제기준의 문제점을 확인하기 위해 초점집단 인터뷰를 실시하였고, 임상경력 1년 이상의 간호사 482명을 대상으로 의료법 개정으로 인한 간호사의 직무 변화 및 교육과정에 추가되어야 하는 내용에 대해 설문조사를 실시하였다.

연구진의 집중회의를 통해 국내와 미국의 신규간호사 직무관련 선행연구를 바탕으로 8개 영역, 총 242개의 Activity statement가 도출되었고, 온라인을 통해 각 Activity statement에 대한 직무, 시험 간 연계타당도와 관련 교과목 타당도를 조사하였다.

1차 전문가 패널조사 결과 242개 Activity statement 중 직무-시험 연계타당도 관련 선별기준에 의해 32개 항목이 삭제되었고, 20개 항목이 수정 후 채택되었다. 또한 4개 항목의 관련 교과목이 교과목관련 타당도 선별기준에 의해 수정되어 210개 항목이 선별되었다.

2차 전문가 패널조사도 1차 패널조사와 같은 방식으로 각 Activity statement에

대한 직무, 시험 간 연계타당도와 관련 교과목 타당도를 조사하였고, 해당 항목의 기타 의견을 종합하여 1차 선별된 210개 Activity statement 중 직무-시험 연계타당도 관련 선별기준에 의해 5개 항목이 삭제되었고, 64개 항목의 관련 교과목이 수정되었다.

그 결과 한국의 실정에 맞는 최종 205개의 행위 진술(activity statement; AS)이 도출되었고, 간호 관리와 전문성 향상 36개, 안전과 감염관리 20개, 잠재적 위험요인 39개, 기본간호 28개, 생리적 통합유지 35개, 약물과 비경구요법 18개, 심리사회적 통합유지 10개, 건강증진 및 유지 19개로 구성되어 있다.

Activity statement의 8개 대분류는 그대로 유지하였으며, 박인숙 등(2014)의 연구에서 제시한 308개 출제내용, 강소영 등(2015)의 연구결과 제시한 372개 최소직무능력으로부터 신규간호사가 알아야 할 필수적인 내용을 선별하여 205개의 항목으로 축소된 Activity statement를 도출하였다.

이는 직무분석의 결과를 토대로 한 8개의 임무 영역별(대분류)로 구체적인 직무에 대한 진술(중분류)로 향후 직무기반 문항개발기준으로 활용될 수 있음을 보여주었다.

마. 간호사 국가시험 통합출제를 위한 모형구축 (송라윤 외, 2018)

1) 간호사 국가시험 통합모형에 따른 직무항목과 지식항목

간호사 국가시험 통합출제를 위한 모형구축 연구는 최신 임상현장을 기반으로 타당도가 제시된 신규간호사 직무항목(Activity statement, AS)과 2015년 7개 교과목의 표준 학습목표를 연계하고, 표준 학습목표로부터 신규간호사 직무항목별 문항개발을 위한 지식항목(Knowledge statement, KS)을 추출하였다. 추출 된 지식항목에 대한 타당도를 평가함으로써 신규간호사의 직무와 전공과목 표준 학습목표가 연계된 한국의 간호사 국가시험 통합모형을 제시하고자 하였다.

즉 1단계로 신규간호사의 직무기반 직무항목을 도출하기위해 선행연구(김영경 외, 2018)의 신규간호사 직무기준 205개 Activity statement(AS)로부터 분류의 적절성 및 문항의 구체성 정도에 따라 통합 가능한 문항을 정리하여 87개 AS가 제외되었다.

또한 잠재적 위험요인 관리영역에서 ‘신생아부터 청소년기 대상자에 대한 간호 및 교육 제공’ 항목을 ‘신생아 간호’, ‘영아 간호’, ‘유아 간호’ 등으로 세분화하면서 4개 AS가 추가되었고, 기본간호 영역에서는 ‘대상자의 일상생활활동(ADL)의 정도를 사정하고 중재’ 항목을 ‘활동과 자기돌봄장애 사정 및 간호’, ‘활동지속성 장애 대상자 사정 및 간호’, ‘운동기능장애 간호중재’로 세분화하여 2개 AS가 추가되었다.

이후 연구진 간 검토 및 논의를 거쳐 각 교과목에는 포함되나 AS에 포함되지 않은

직무항목 10개(위험요인 사정 4개, 생리적 통합유지 2개, 심리사회적 통합유지 2개, 건강증진 및 유지 2개)를 추가하고, 분류의 적절성과 직무 항목 간 추상도를 조정하여 최종 8개 영역, 134개의 신규간호사 직무항목(AS 개정안)을 확정하였다.

2단계로 확정된 134개 신규간호사 직무항목과 전공 교과목별 제시된 표준 학습목표(한국간호과학회, 2017)을 연계함으로써 문항개발 기준이 될 지식항목(Knowledge statement, KS)을 추출하였다.

또한 직무항목과 지식항목의 연관성, 지식항목의 구체성 즉, 통합형 직무항목별 지식항목에 대한 타당도를 조사하여 최종 1,303개 지식항목(KS)이 확정됨으로써 신규간호사 직무항목 기준의 국가시험 교과목 통합모형을 제시하였다.

국가시험 통합모형은 현행 간호사 국가시험의 출제기준인 8개 교과목으로 되어있는 간호교육과정을 신규간호사 실무역량의 관점으로 통합한 것으로 8개 대분류(간호관리와 전문성 향상, 안전과 감염관리, 위험요인 사정, 기본간호, 생리적 통합유지, 약물과 비경구요법, 심리사회적 통합유지, 건강증진 및 유지)로 재분류되었다. 또한 각 교과목의 표준교육목표와 신규간호사 직무역량 행동지표(134개 AS)를 연계하여 8개 대분류의 행동목표에서 요구하는 지식항목(1,303개 KS)을 추출하였다. 구체적인 내용은 <표 III-2>와 같다.

<표 III-2> 간호사 국가시험 통합모형에 따른 실무항목과 지식항목

1영역. 간호관리와 전문성 향상

대분류	No	신규간호사 직무항목 (Activity Statement 개정안)	지식항목 (Knowledge statement)
I. 간호 관리와 전문성 향상	1	인수인계시행	업무 인수인계
	2	법적 직무범위 내에서 간호 수행	간호표준실무, 간호관리표준실무
	3	기록 시 표준화된 약어 사용	간호기록 시 표준약어
	4	지침에 따라 간호기록	간호기록지침
			환자 개인정보 보호
			간호정보시스템
	5	입원, 전동, 퇴원	입원관리, 퇴원관리 및 교육, 전과전동업무
			입퇴원, 전과전동시 인수인계
			고위험 환자관리체계
	6	장비를 적절하고 안전하게 사용	물품관리
	7	간호단위 물품교환체계에 따른 물품관리	약품관리
			마약 및 고위험 약품관리
	8	질 향상(QI) 활동에 참여	의료의 질관리, 간호의 질관리 및 질향상활동
			질관리 전략 및 절차
			의료기관인증제도 및 인증기준
			질관리 평가기준 및 도구

대분류	No	신규간호사 직무항목<br>(Activity Statement 개정안)	지식항목<br>(Knowledge statement)
	9	간호사업 평가 관련 업무수행<br>(도구개발, 자료조사, 분석, 비교,사업개선)	간호사업평가
			간호관리 통제기능
	10	간호전문직 윤리 준수와 역할	간호윤리, 생명의료윤리 원칙,<br>윤리이론 및 윤리적 사고
			한국간호사 윤리강령
			연구 윤리
			전문직 협력관련 간호윤리
			자원분배 관련 간호윤리
			간호전문직관
			간호사의 임무와 역할
			소수집단 인권보호 및 윤리적 간호
			정신건강복지법
			공중보건관리 법률
	11	대상자의 개인정보 및<br>사생활 보호	대상자의 개인정보 및 사생화 보호권리<br>(사생활, 인격권, 정보보호권, 평등권)
			대상자의 법적 권리 보호방안
			공중보건상 재난 시 자료의 비밀보장
	12	대상자에게 치료 및 절차에 따라 적절한 설명을 하고 동의를 획득하였는지 확인	간호사의 법적 의무
	13	환자의 권리와 책임에 관하여<br>대상자에게 교육 제공	간호와 치료에 관한 환자권리 및 책임
			간호사의 법적 의무와 책임
			환자권리존중 및 보호
	14	억제대 사용 시 법적 및 윤리적 간호	신체억제대 사용지침, 억제대 사용관련 윤리적 사고 및 결정
	15	일차의료기반의 보건간호	일차보건의료
			지역사회간호의 정의
			지역사회와 건강개념
			건강의 결정요인
			국제보건기구의 간호정책 및 활동
			보건의료체계의 이해
			진료비 지불보상제도
	16	지역사회 간호사업의 법적 기준 및 지침에 따른 활동 참여	보건관리자의 역할 및 업무
			산업전문간호사의 역할
			보건/간호사업 기획 및 수행
			보건교육자, 보건진료원의 역할
			보건정보 및 기술계획
			건강증진 종합계획
			간호사업의 법적 기준 및 지침
	17	사례관리활동 참여	사례관리 정의 및 원칙
			조정과 협력
			간호관리 체계 및 과정
			사례관리모델, 사례관리과정
	18	업무를 조직화하여 일을 효율적으로 관리	간호관리업무, 역량
			관리이론
			기획 원칙 및 구성요소, 기획단계
			계획안과 목표관리
			의사결정
			예산관리
			조직화 원리, 조직구조 및 권한

대분류	No	신규간호사 직무항목 (Activity Statement 개정안)	지식항목 (Knowledge statement)
			직무설계, 직무분석, 직무평가
			간호전달체계
			간호조직문화와 조직변화
			간호인적자원 관리
	19	환자분류체계 관련 정보수집 및 활용	환자분류체계
			간호업무량 및 인력산정, 간호업무배정
	20	간호대상에 적합한 다양한 자원 및 매체선택	간호서비스마케팅
	21	취약가족에 필요한 지역사회 자원 활용	취약가족과 간호, 가족의 개념
			가족생활주기의 특성
			가족관련이론
			가족간호과정
	22	전문직 간 협업	조정과 협력
			리더십
			동기부여이론 및 동기부여 적용
			임파워먼트
			효과적인 의사소통
			주장행동
			갈등관리, 직무스트레스 관리

2영역. 안전과 감염관리

대분류	No	신규간호사 직무항목 (Activity Statement 개정안)	지식항목 (Knowledge statement)
II. 안전과 감염 관리	23	안전한 환경 제공	사생활유지관리
			환경관리 및 환자안전관리
			환자안전사고 예방(관련법 포함)
			환자안전사고 보고체계 및 사고기록
			환자안전문제 발생 시 대응방안 및 운영체계
			조직의 환자안전문화
			보안계획(신생아실 안전, 폭력, 통제된 접근) 및 절차 준수
			안전사고 유형, 위험요인과 예방
			낙상예방 및 관리
			안전사고 시 기록
			억제대 적응증, 억제대 종류, 억제대 적용 시 주의점, 적용방법
	24	감염관리	병원감염관리
			감염관리지침
			무균술의 기본원리
			소독방법 / 소독수준, 소독방법, 소독제의 종류와 용도
			멸균방법 / 멸균준비, 멸균방법과 적용
			무균술의 원리, 상처감염예방법
			외과적 무균술 정의, 외과적 무균술의 기본원리와 적용
			표준주의지침

대분류	No	신규간호사 직무항목 (Activity Statement 개정안)	지식항목 (Knowledge statement)
			감염위험요인 사정 / 감염유형, 감염회로, 감염에 대한 신체방어, 감염 감수성에 영향을 미치는 요인
			의료관련감염 정의, 종류, 위험요인, 교차감염
			병원감염(요로감염, 폐렴, 수술창상감염, 혈액감염 등) 관리 및 결과 평가
			전파경로별 주의지침 (공기주의, 접촉주의, 비말주의), 다제내성균 감염관리, 면역저하 대상자 관리(역격리), 보호장구 착용과 벗기
			의료폐기물 종류별 관리 및 주의점
	25	위험물질과 유해물질 관리	고위험약품 및 마약관리

3영역. 위험요인 사정

대분류	No	신규간호사 직무항목 (Activity Statement 개정안)	지식항목 (Knowledge statement)
III. 위험 요인 사정	26	간호계획, 진료지침 수행	간호과정
	27	대상자의 건강문제에 대해 우선순위 결정	건강문제의 우선순위 결정
	28	활력징후 사정	활력징후의 중요성과 영향요인, 각 활력징후 정상범위, 측정법, 의미와 해석
			말초산소포화도에 영향을 미치는 요인, 측정방법 및 주의점, 정상범위, 의미와 해석
	29	신생아 간호	청결한 기도유지, 고무흡인기의 적절한 사용, 호흡측정 및 호흡양상 관찰
			체온유지간호
			신생아목욕간호, 제대간호, 기저귀발지 예방
			신생아의 영양, 모유수유/인공수유 방법, 신생아 수유행동, 체중측정
			감염과 상해로부터 보호, 예방접종
			애착행동사정, 부모-신생아 애착증진 간호
			신생아 퇴원기준목록, 신생아 돌봄지도, 수면과 활동, 신생아 선별검사와 예방접종
	30	영아 간호	고형식이의 선택과 준비, 고형식이의 시작, 이유, 스스로 먹기, 음식 알레르기
			치아발달, 치아건강관리, 저충치식이, 유아기 우식증 예방
			낯가림, 분리불안 대처 방안
			영아산통의 증상, 치료관리, 간호중재
			영아돌연사증후군 원인, 위험요인, 보호요소, 간호중재
	31	유아 간호	대소변가리기 훈련 준비, 방법, 단계
			분노발작 대처 방안
			거부증 대처 방안
			퇴행 대처 방안
	32	학령전기 간호	언어문제 예방과 조기발견, 말더듬 관리
			학령전기아동의 공포특성, 극복방안

대분류	No	신규간호사 직무항목<br>(Activity Statement 개정안)	지식항목<br>(Knowledge statement)
			악몽과 야경증 대처방안
	33	학령기 간호	학령기 아동의 사회적 관계, 또래집단의 정체성과 협력, 부적절한 또래와의 관계
			학교공포증 대처방안
			성장통 관리
			영양, 수면과 휴식, 운동과 활동, 치아건강, 안전한 습관, 사고예방
	34	청소년 간호	사춘기 여성 건강사정 /2차 성징, 테너단계, 여아와 남아의 성적 성숙
			초경
			사춘기의 신체, 심리적 발달 특성
			건강에 대한 관점, 청소년기의 위험행동(중독, 자살, 학교폭력, 섭식장애) 간호관리
	35	폐경기 여성 간호	완(폐)경 여성 건강사정과 간호
	36	노인 간호	노화에 따른 생리적 변화
			노인 대상자 건강교육
	37	성 건강 간호	월경전 증후군 간호
	38	건강력사정	건강력 사정을 위한 문진요소
			기관별 review of system
	39	신체검진 수행 및 결과해석	초점 사정(focused assessment) 수행
			기관별 신체검진 (두경부, 흉부, 복부, 근골격계, 호흡기계, 심혈관계, 신경계, 생식기계)
	40	생식기 건강사정	여성생식기 사정
			생식기 검진 전후 간호(질경검진, 자궁경부세포검사, 세포도말검사)
			유방건강사정
	41	태아 건강사정 및 간호	태아건강사정(태아초음파, 태동측정, 신체계측, 생물리학적 계수, 모체혈청검사, 태아전자감시, 양수천차, 융모막생검)
	42	신생아 건강사정 및 간호	출생시 신생아 평가-심박동수, 호흡노력, 근력, 자극에 대한 반응, 피부색
			머리둘레, 가슴둘레, 머리엉덩길이, 복부둘레, 머리발꿈치 길이, 체중
			신생아 신체사정을 위한 환경, 방법과 특징적 소견, 신생아 반사 사정
			신생아 선별검사 종류, 방법 및 시기
			신생아의 울음 및 수면의 특성, 여아의 마유, 혈액성 분비물, 남아의 통증성 지속성 발기
			생리적 황달원인, 기간, 관리
	43	고위험신생아 건강사정 및 간호	고위험 신생아의 생리적 기능과 특성, 신체사정
			고위험 신생아의 체온, 호흡유지, 영양유지, 피부간호
			신생아 통증조절, 광선요법, 교환수혈 시 간호
			출생시 손상 신생아 간호
			미숙아와 과숙아의 특성

대분류	No	신규간호사 직무항목<br>(Activity Statement 개정안)	지식항목<br>(Knowledge statement)
			고위험(용혈성질환, 패혈증, 근골격계장애, 소화기계장애, 유전질환, 대사질환)간호
			당뇨병, 중독, 모체요인과 관련된 감염산모의 신생아 간호
	44	심폐소생술 간호	심정지 환자의 심폐소생술, 세동제거술
	45	응급간호	응급의료전달체계, 응급간호의 원칙, 응급환자 분류기준
			외상의 유형에 따른 간호, 출혈시 간호, 쇼크의 유형, 쇼크의 유형에 따른 간호
			중독의 유형, 중독의 유형에 따른 간호
	46	진단검사 간호	진단검사 수행, 진단검사결과 확인,<br>진단검사 전후 간호
			침습적 시술<br>(중심정맥관, 흉강천자, 기관지내시경)
			침습적 검사방법(양수천자, 경피제대혈채취, 유모막융모생검) 검사 전후 간호
			생식기 검진 전후 간호(질경검진, 자궁경부세포검사, 세포도말검사)
	47	수술 간호	수술동의서, 수술전/중/후 간호
			여성 생식기 수술 간호
			유방절제술 환자간호
			중증도 진전(moderate sedation)시술 중 또는 후에 대상자 관리
			마취의 종류 및 방법, 합병증
			국소마취의 종류(척추마취, 경막외 마취)에 따른 간호과정
	48	산전간호와 교육	임부의 생리적 변화
			임부의 건강사정과 산전간호
			임부간호(임신주기별 간호)
			임부와 가족의 사회심리적 적응과 간호
	49	분만중 간호와 교육	분만관련 주요개념(분만요소, 산도, 골반경선, 아두경선, 태세, 태향, 태위, 선진부)
			분만생리(분만과정, 분만기전, 자궁수축, 분만전구증상)
			통증완화간호(통증사정, 비약물적방법, 약물적 방법)
			산부 간호(1기, 2기, 3기, 4기, 간호, 상호작용증진)
	50	산후관리와 교육	산후생리적 변화
			산모와 가족의 사회심리적 적응과 간호<br>(사회심리적 변화, 모성역할, 애착증진간호)
			산모 간호(산후건강사정과 건강관리)
			모유수유 간호
	51	고위험임부 간호	고위험 임신요인 사정
			출혈성 임신 건강문제가 있는 임부 간호(유산, 자궁경관무력증, 자궁외임신, 포상기태, 전치태반, 태반조기박리)
			임신성고혈압 임부 간호
			내과적 임신 건강문제가 있는 임부 간호<br>(임신성 당뇨, 갑상선기능장애, 심장질환, 빈혈, 성전파성질환, 비뇨기질환, TORCH 감염)
			분만과정 관련 건강문제가 있는 산부 간호 (난산, 급

대분류	No	신규간호사 직무항목<br>(Activity Statement 개정안)	지식항목<br>(Knowledge statement)
			속분만, 지연분만, 조기진통 자궁파열, 자궁내번증, 태반유착, 다태분만, 과숙아분만, 조기분만)
	52	고위험 산부간호	태아부속물 관련 건강문제가 있는 산부간호(양수과다증, 양수과소증, 조기파막, 제대탈출)
			대안적분만 간호<br>(유도분만, 흡입분만, 제왕절개분만)
	53	고위험 산모간호	산후감염 산모간호<br>(산욕감염, 자궁내막염, 폐색전증, 유방염)
			산후출혈 산모간호<br>(산후출혈, 자궁이완, 산도열상, 산후혈종)
			산후 우울 산모 간호

4영역. 기본간호

대분류	No	신규간호사 직무항목<br>(Activity Statement 개정안)	지식항목<br>(Knowledge statement)
IV.<br>기본<br>간호	54	체온유지간호	간호력, 체온측정(활력징후에 포함),<br>신체검진(피부상태 조사), 임상검사(혈액배양검사),<br>영향요인(활력징후에 포함)
			체온유지 간호진단
			체온조절 문제 간호계획, 발열단계에 따른 간호,<br>고체온 및 저체온 대상자 간호
			체온유지 간호결과 평가
			열냉요법 적응증, 금기증,<br>적용-건열, 습열, 건냉, 습냉
	55	세척(irrigation) 수행<br>(방광, 귀, 눈)	세척(irrigation) (방광, 귀, 눈) 수행
	56	개인위생간호	구강간호, 등간호, 침상세발, 회음부간호
	57	섭취 및 배설량 사정과 간호	섭취량과 배설량 측정방법, 결과해석
	58	영양사정 및 관리	경관 영양 종류, 적응증, 합병증
			영양상태지표, 영양 및 대사기능의 특징
			소화기능 사정(병력, 신체사정, 진단검사)
			영양과잉, 영양결핍
			흡인, 설사, 변비, 비만, 과체중, 영양부족
			영양요구 간호계획, 치료식이(일반 치료식이, 특별치료식이), 금식, 식욕촉진, 식사돕기
			영양간호 결과 평가
			비경관 영양 종류, 적응증, 합병증
			중심 비경구영양, 말초 비경구영양,<br>완전 비경구영양
			비경구영양(TPN)주입 및 대상자의 반응 평가
			지속적, 간헐적 위관 영양 제공
			경관영양간호 결과 평가
			취장염, 담낭염, 항암화학요법, PCA, 장폐색, 메니에르병, 두개내압상승, 대사성 알칼리증
	59	질환별 영양문제의 사정 및 관리	건강문제별 영양 불균형 특성<br>핵심질환: 식도출혈, 위장관출혈, 위루술, 간이식,

대분류	No	신규간호사 직무항목<br>(Activity Statement 개정안)	지식항목<br>(Knowledge statement)
			장루간호, 비장관, 담관관리
			영양장애 아동간호
			비만 아동간호: 식이상담, 행동요법, 집단 및 가족 참여, 신체활동, 예방
			일반치료식이, 특별치료식이
	60	섭취장애 대상자 사정 및 간호	연하장애 간호
	61	배뇨장애 대상자 사정 및 간호	간호력, 배뇨 영향요인, 배뇨양상,<br>검사물 채취, 신체검진, 진단검사
			배뇨장애와 관련된 간호진단
			배뇨장애 간호- 자연배뇨 촉진방법, 수분섭취,<br>요실금간호(방광조절훈련, 케겔운동)
			단순도뇨, 유치도뇨, 자가도뇨교육, 콘돔카테터,<br>치골상부도뇨관 간호,<br>요로전환술, 요로감염 예방간호, 방광세척
			배뇨간호 결과 평가
	62	투석대상자 관리	복막투석, 혈액투석, CRRT,<br>동정맥루 관리, 투석간호
	63	요루 및 장루 관리	요루 관리
			장루 관리
	64	기관절개부 관리	기관절개관의 적응증 및 간호
	65	배변관리	배변요구와 관련된 간호진단
			관장종류 별 목적 및 간호(청결관장, 정체관장, 구풍관장, 투약관장, 용수관장, 역류관장)
			배변장애 간호
			변비, 설사, 고창, 변실금, 분변매복
			염증성장질환(궤양성대장염, 크론병),<br>대장암, 직장암, 치핵
			배변장애간호 결과평가
	66	수면과 휴식 간호	수면력, 수면에 영향을 미치는 요인,<br>수면일지, 신체검진, 진단검사
			수면증진간호 및 결과평가
			수면간호 결과평가
			수면생리, 수면각성 장애
			수면각성장애
			피로
			암, 간염, 만성폐쇄성폐질환, 빈혈, 투석, 결핵, 울혈성 심부전, 갑상샘기능 항진/저하증
	67	피부통합성 사정 및 간호	면역성피부질환(건선,아토피피부염),<br>감염성피부질환(무좀, 단순포진, 대상포진, 농가진, 연조직염), 화상, 피부종양
	68	이동 간호	신체역학 원리, 이동절차의 일반적 지침, 신체역학을 이용한 들어올리기, 기구 이용한 이동법,<br>보행 및 보행보조,<br>보행 보조기구의 종류 및 사용방법
	69	활동과 자기돌봄장애 사정 및 간호	간호력, 활동과 운동 영향요인, 신체검진, ADL,<br>부동으로 인한 문제
			활동 및 운동 간호진단, 활동 및 운동 간호계획

대분류	No	신규간호사 직무항목 (Activity Statement 개정안)	지식항목 (Knowledge statement)
			관절범위운동의 종류, 목적, 주의점, 각 관절별 수행방법
			체위의 종류, 올바른 체위 유지목적
			활동과 운동 간호 결과평가
			운동유형, 운동효과, 부동의 영향
			골절, 고정, 뇌졸중, 무의식, 파킨슨병, 길랑-바레증후군, 근위축측삭경화증, 다발성경화증, 척수손상
	70	외과적 장치 관리	외과적 견인장치(splints, braces, casts)의 목적, 종류, 간호
	71	척추손상 및 질환대상자의 간호	절단, 의식장애, 뇌졸중, 파킨슨병, 근무력증, 다발경화증, 근위축측삭경화증, 길랑-바레 증후군, 척수손상, 추간판탈출, 척추관협착
	72	관절대치술 환자 간호	관절대치술의 적응증, 수술 전후 간호

5영역. 생리적 통합유지

대분류	No	신규간호사 직무항목 (Activity Statement 개정안)	지식항목 (Knowledge statement)
V. 생리적 통합 유지	73	호흡기능장애 대상자 간호	호흡기계 해부생리, 호흡과정 및 호흡조절기전, 관류와 환기
			호흡기계 건강사정(정상과 비정상 구별)
			호흡기계 장애 핵심질환: 폐쇄성호흡기질환(천식, 만성폐쇄성폐질환), 염증성호흡기질환(폐렴, 흉막염), 감염성호흡기질환(상기도감염, 폐결핵), 외상성호흡기질환(기흉, 늑골골절), 호흡기중증질환(폐색전증, 급성호흡부전, 성인호흡곤란증후군)
			산소요법
			흉부물리요법, 강화폐활량계, 체위배액, 심호흡과 기침, nebulizer
			동맥혈가스분석 해석, 호흡성/대사성 알카리증과 산증
			아동기 호흡특성, 호흡기계 건강사정, 아동의 비인두염
			핵심질환(인두염, 편도염, 중이염, 크루프, 기관지염, 세기관지염, 폐렴, 결핵) 아동간호
	74	호흡증진중재	기도관리, 검체채취, 흉부물리요법, 강화폐활량계, 체위배액, 심호흡과 기침, nebulizer
			핵심질환: 호흡기 중증질환(폐색전증, 급성호흡부전, 성인호흡곤란증후군) 간호
			산소요법, 흉부물리요법(타진, 진동, 체위배액), 강화폐활량계, 심호흡과 기침, nebulizer
			흡인의 적응증, 절차 및 간호(주의사항 포함)
			심호흡, 기침, 입술 오므리기호흡, 횡격막 호흡, 강화폐활량계 목적 및 교육방법
			타진, 진동, 체위배액의 목적, 원리, 적용방법 및

대분류	No	신규간호사 직무항목 (Activity Statement 개정안)	지식항목 (Knowledge statement)
			주의점
			산소요법의 종류별 장단점, 목적, 적응증 및 간호
			인공기도관의 적응증 및 관리
	75	호흡보조장치 관리	산소전달시스템, 진단검사, 약물, 인공호흡기 모드 및 관리방법, 감염관리
	76	중심정맥관 관리	무균술 적용과 삽입부위 사정, 주입장치, 약물주입, 혈액체취시 주의점, 삽입부위드레싱, 합병증 종류 및 간호
			중심정맥주사(비터널카테터 말초삽입중심정맥관, 터널카테터, 이식포트) 유형 및 적응증
	77	태아질식 증상과 징후 사정 및 간호	신생아 호흡 특성, 호흡기계 건강사정(정상, 비정상 구분), 고위험신생아 호흡기계 관련장애(호흡곤란증후군, 무호흡, 태변흡인, 기관지폐형성이상, 미숙아망막병)
	78	고위험신생아 보육기 적용간호	고위험 신생아의 통증조절, 광선요법, 교환수혈
	79	호흡재활관리	단계별 호흡재활 간호중재
	80	심전도관리	심장리듬, 심전도 전극 위치
	81	순환보조장치관리	심실보조장치,<br>인공심박동기를 가지고 있는 대상자의 간호
	82	동맥관 관리	동맥관 모니터, 혈역학, 침습적 라인 관리(동맥관 합병증), 동맥혈 가스
	83	조직관류장애 대상자 사정 및 간호	고혈압, 동맥경화, 대동맥류, 말초동맥질환, 심부정맥혈전증, 정맥류, 림프부종
	84	체액전해질 불균형<br>사정 및 간호	부종, 탈수, 화상 간호
			전해질 균형의 생리기전과 증상 및 징후,<br>관련질환(고/저칼륨혈증, 고/저나트륨혈증, 고/저칼슘혈증, 부갑상샘기능항진/저하증, 항이뇨호르몬부적절분비증후군)
	85	활동지속성 장애<br>대상자 사정 및 간호	순환운동과 감각평가, 조기이상, 순환기구,<br>신체역학, 체위변경 및 가동화 기법
			관련질환(심부전, 관상동맥병, 판막, 빈혈, 호흡기계)
	86	정맥순환증진장치 간호	순환증진 전략, 조직관류, 혈전색전증 예방
	87	혈액기능장애 대상자 간호	핵심질환 : 빈혈, 백혈별, 파종성혈관내응고, 혈우병, 림프종, 다발골수종, 응고장애의 병태생리, 증상과 징후
			혈액의 기능 및 구성성분
			혈액계 건강사정, 각 진단검사의 목적/결과 해석
			혈액제제 투여와 효과평가,<br>혈액기능장애 간호문제에 따른 간호중재
			항응고요법, 혈전용해요법관련 간호중재
			아동기 혈액 특성, 혈액계 건강사정,<br>진단검사의 목적 및 결과해석
			철결핍성빈혈, 재생불량성빈혈 환자, 혈우병, 특발성 저혈소판 백반병 간호
	88	순환기능장애 대상자 간호	심박출량 감소, 심장의 구조 및 기능, 심장주기, 심박동 조절 및 심박출량 영향요인

대분류	No	신규간호사 직무항목<br>(Activity Statement 개정안)	지식항목<br>(Knowledge statement)
			심혈관계 건강사정<br>(정상과 비정상 구별, 흉통, 심계항진, 심잡음)
			핵심질환 : 심부전, 심장판막질환, 심근근육병, 심장눌림증, 심내막염, 심근염, 심장염
			핵심질환 : 심부전, 관상동맥병, 부정맥, 심장눌림증
			아동 심장의 구조와 기능 및 심박출량 영향요인, 선천심장병의 병태생리
			아동 심혈관계 건강사정(정상과 비정상 구분)
			선천 심장병(심방중격결손, 심실중격결손, 대동맥축착, 대동맥판협착, 폐동맥판협착 팔로네증후, 대혈관전위, 심내막염) 간호
	89	심장수술 후 간호	심장수술 후 합병증(예방) 관리
			심장이식의 적응증과 간호관리
	90	소화기능장애 대상자 간호	소화기계의 구조 및 기능, 소화와 흡수기전
			소화기계 건강사정(정상과 비정상 구분)
			위장질환(위염, 위암, 소화성궤양) 간호
			간질환(간염, 간경화, 간암, 담석증, 담낭염, 담낭암, 췌장염, 췌장암) 간호
			장질환(과민대장증후군, 크론병, 궤양성대장염, 소장흡수불량증후군, 장폐색증) 간호
			복막염 대상자 간호
			소화기계 질환별 치료식이
			아동기 소화기능의 특성과 소화기계 건강사정
			아동의 급성위장관염, 변비와 유분증,<br>장중첩증 간호
			고위험신생아 소화기능의 특성과 건강사정
			신생아 간호 :<br>소화기계장애(구순/구개열, 기관식도루, 항문직장기형, 선천성유문협착증, 선천거대결장, 담도폐쇄, 복벽손상, 탈장, 괴사소장대장염)
	91	배뇨장애를 가진<br>대상자 간호	비뇨기계 구조 및 기능, 배뇨기전 및 영향요인, 비뇨기계 건강사정. 비뇨기계 관련질환(방광염, 사구체신염, 급성. 만성신부전, 신장암, 방광암, 신대체요법, 결석, 대사성산증, 전립샘장애)
			아동의 비뇨생식기 특성, 건강사정,<br>비뇨기계 기형 아동 간호
			아동의 비뇨생식기 건강문제 간호 (비뇨기계 기형, 요로감염, 방광요관역류, 사구체신염, 신증후군)
	92	당질대사장애 대상자 간호	당질대사기전, 저혈당의 원인, 위험요인, 증상, 간호, 고위험신생아의 혈당조절
			소아당뇨병의 증상 및 징후, 진단검사, 혈당감시 및 조절(식이, 약물), 당뇨합병증 예방 및 관리
	93	당뇨병 합병증 예방을 위한 간호	당뇨병의 합병증 증상 및 징후
	94	내분비계장애 대상자 간호	내분비계 해부생리 및 기능(호르몬),<br>내분비계 건강사정(정상과 비정상 구별)
			뇌하수체질환(뇌하수체 종양, 요붕증, 항이뇨호르몬부적절분비증후군) 간호

대분류	No	신규간호사 직무항목<br>(Activity Statement 개정안)	지식항목<br>(Knowledge statement)
			부신질환(쿠싱증후군, 알도스테론증, 에디슨병, 갈색세포종) 간호
			갑상샘기능 항진/저하증, 부갑상샘기능 항진/저하증 간호
			성장장애, 성조숙 아동 간호
			선천성 갑상선 저하증 아동간호
	95	생식기 질환/생식기 건강문제를 가진 대상자 간호	생식작용과 호르몬, 월경주기, 월경장애의 종류(무월경, 기능성 자궁출혈, 월경전증후군, 월경곤란증), 정의, 원인, 증상 및 징후, 치료방법
			무월경, 무월경 치료 및 간호
			기능성 자궁출혈의 치료 및 간호
			자궁의 구조 및 기능, 여성 생식기 건강사정
			자궁내막증, 자궁선근증, 자궁내막증식증, 자궁내막폴립 간호
			자궁탈수, 자궁전방전위, 생식기누공 간호
			복압성 요실금 간호
			난(불)임의 정의 및 진단, 원인, 치료과정
	96	면역손상 대상자 사정 및 간호	면역반응의 기전 및 건강사정
			면역손상관련질환(자가면역, 후천면역결핍증후군, 과민반응-알러지, 장기이식, 전신홍반성루프스)의 종류와 간호
			아동의 면역기전 및 건강사정 (정상과 비정상 구분)
			아동의 면역손상관련질환<br>(아토피피부염, 알레르기비염, 천식, 류마티스열, 가와사키병, 헤노흐-쇤라인자색반)
	97	감각기능장애를 가진 대상자 사정 및 간호	감각기계 구조 및 기능, 건강사정(정상과 비정상 구분)
			시각장애 관련질환(염증성눈질환, 망막박리, 녹내장/백내장, 각막이식)간호
			청각장애 관련질환(염증성귀질환, 메니에르병, 난청)
	98	두개내압 상승 환자의 간호	뇌의 구조 및 기능, 뇌 혈액공급, 두개내압 조절기전, 의식수준 사정
			두개내압상승 환자간호
	99	신경계 질환별 간호중재	신경계 신체사정의 정상과 비정상 구별
			신경운동장애 관련질환(뇌졸중, 간질, 파킨슨병, 근무력증, 다발성경화증, 길랑-바레증후군, 안면신경마비, 삼차신경통, 추간판탈출, 척추외상, 척추관협착)
			뇌동맥류, 외상성뇌손상, 두통, 뇌종양, 뇌막염 간호
			뇌졸중 간호
			인지기능 장애의 원인, 위험요인, 인지기능장애 관련질환(무의식, 뇌졸중, 외상성뇌손상)
			치매 관련요인, 행동특성, 간호
			아동기 뇌의 특성, 신경계 건강사정
			아동관련 신경계 장애 관련질환(수막염, 열발작, 뇌전증)간호
			고위험 신생아 신경계 관련질환(뇌실내 출혈, 경련, 이분척추, 수두증)
			섬망 관련요인, 행동특성, 간호
	100	운동기능장애	근골격계 구조 및 기능, 근골격계 건강사정

대분류	No	신규간호사 직무항목 (Activity Statement 개정안)	지식항목 (Knowledge statement)
		간호중재	부동, 운동유형, 관절범위운동, 체위 유지 및 변경, 이동간호, 자가간호결핍 간호
			운동기능장애 관련 근골격계질환(골절, 골관절염, 골다공증, 류마티스관절염, 요통, 통풍, 골수염, 염좌, 강직척추염, 관절대치술, 절단) 간호
			운동기능장애 관련 신경계질환(뇌졸증, 길랑-바레증후군, 근위축측삭경화증, 파킨슨병, 척수손상, 추간판탈출, 척추관협착, 의식장애) 간호
			아동의 근골격계 특성 및 관련질환(척추만곡, 근육디스트로피, 뇌성마비, 소아류마티스관절염) 간호
	101	상처간호 수행 및 드레싱 교환	상처유형, 영향요인, 상처의 상태
			욕창단계, 욕창위험요인, 욕창위험요인 측정도구, 욕창간호
			드레싱 종류별 적응증, 상처세척, 배액관 관리, 붕대 및 바인더 적용
	102	화상환자 간호중재	화상의 정의, 원인, 병태생리, 임상증상, 치료과정, 간호중재
	103	피부질환 대상자의 간호중재	피부계 구조 및 기능, 피부계 건강사정(정상과 비정상 구별)
			아동피부질환(접촉피부염, 기저귀피부염, 농가진, 옴, 머릿니, 칸디다증, 혈관종, 여드름) 간호
			피부통합성장애 관련질환(면역성피부질환, 감염성 피부질환, 화상, 피부종양) 간호
	104	배액장치관리	흉관, 상처 음압장치 유지 및 관리, 밀봉흉곽배액을 가진 대상자 간호
	105	계통별 신생물 질환 대상자의 간호	관련기관의 구조 및 기능, 각 종양의 원인, 증상 및 징후, 검사
			신생물질환 치료방법, 방사선요법, 항암화학요법, 수술전 간호, 수술 후 간호
			생식기종양(외음양성질환, 외음암, 자궁경관 상피내종양, 자궁경부암, 자궁근종, 자궁내막암, 융모상피암, 난소난종, 난소양성종양 난소암) 간호
			소아종양(조혈모세포이식/골수이식, 골수생검, 백혈병, 뇌종양, 신경모세포종, 빌름스종양, 골육종) 간호
	106	통증간호	통증의 원인 및 영향요인, 통증사정, 통증 완화를 위한 약물요법 및 비약물요법
	107	재활간호서비스	재활간호
			정신사회재활
	108	(법정) 감염질환자의 전파예방 및 간호	대상 기관의 구조 및 기능, 건강사정, 전파경로별 격리주의, 의료폐기물관리
			여성생식기의 구조 및 기능, 건강사정, 외음의 감염성 질환
			외음의 감염성 질환, 세균성 질염, 트리코모나스 질염, 캔디다성 질염, 노인성 질염, 자궁경부염, 난관염, 골반감염 간호
			성전파성 질환(후천성면역결핍증, 단순포진 바이러스성 질환, 인유두종 바이러스성 질환, 매독 세균성 질환, 임질 세균성 질환, 클라미디아 세균성 질환) 간호

대분류	No	신규간호사 직무항목 (Activity Statement 개정안)	지식항목 (Knowledge statement)
			아동기 면역의 특성
			아동기 호발 전파성질환(수두, 풍진, 홍역, 볼거리, 일본뇌염, 돌발피진(장미진), 성홍열, 손발입병, 파상품, 디프테리아, 백일해)
			질병의 자연사와 예방수준
			집단면역
			감염성 질환의 잠복기
			질병발현의 역학적 요인
			역학적 측정지표(유병률, 사망률)
			진단검사 및 역학적 연구
			법정전염병의 정의, 종류, 지정 목적 및 관리방법

6영역. 약물 및 비경구 요법

대분류	No	신규간호사 직무항목 (Activity Statement 개정안)	지식항목 (Knowledge statement)
VI. 약물 및 비경구 요법	109	투약의 적절성과 정확성	투약의 5원칙
			약물작용기전과 유형
			약물투여경로별 목적 및 특성
			경구투여절차 및 평가
			처방된 약품의 적절성과 처방의 정확성 평가
			근육/피하/피내/국소투여/정맥주사 절차 및 평가
			검진이나 검사 결과에 기반하여 약물의 투여량 확인 (혈당수치에 따른 인슐린 투여, 특정 혈압을 유지하기 위해 약물의 투여)
	110	약물 투여에 필요한 계산 시행	약용량 계산(성인/아동)
	111	정맥주입장치 관리	중심정맥관, 이식형 포트 관리
	112	약품관리	지침에 따라 규제약물을 관리 (증인, 잔량폐기), 안전하고 통제된 환경에서 의약품 취급 및 관리
			투약오류 모고 및 예방
	113	말초정맥관 삽입, 유지, 제거	말초정맥관 삽입/유지/제거
	114	대상자에게 약물에 대해 교육	약물치료에 대한 대상자의 반응 (치료효과, 부작용, 이상반응) 평가
	115	고위험 약품관리(마약관리)	마약관리방법

7영역. 심리사회적 통합유지

대분류	No	신규간호사 직무항목 (Activity Statement 개정안)	지식항목 (Knowledge statement)
VII.	116	이상행동 사정 및 간호	정신간호의 원리(정신간호의 개념, 정신간호사의 역할과 기능, 치료적 자기이용, 자기인식, 대상자-간호

대분류	No	신규간호사 직무항목<br>(Activity Statement 개정안)	지식항목<br>(Knowledge statement)
심리 사회적 통합 유지			사의 상호작용)
			이상행동 사정과 간호
			정신간호의 발달
			정신간호의 이론적 모형(정신분석모형, 대인관계모형, 사회적 모형, 신존적 모형, 행동모형, 의사소통모형, 간호모형 등)
			스트레스 대처 및 관리(스트레스 개념, 반응 및 이론, 스트레스 대처기전 및 관리기법)
			정신건강사정(정신건강 개념, 신체와 정신과의 관계, 정신건강과 정신질환의 연속성, 정신건강 영향요인, 정신건강 상태 사정)
	117	폭력의 잠재성 평가 및 예방	위기사정 및 관리(위기의 정의, 특성, 종류 및 중재, 자살의 단서, 정신역동, 위험요인 및 예방적 간호중재, 자살 시도자 및 유가족 간호중재, 공격행동 사정 및 대처방안)
	118	학대 또는 방임 대상자를 확인하고 적절하게 중재	성폭력의 정의, 원인, 영향 및 성폭력 피해 대상자 간호
			가정폭력의 개념, 유형(아동학대, 배우자학대, 노인학대), 가해자와 피해자의 특성, 예방 및 대처방안
			물질관련 및 중독장애의 정의, 유형(물질관련장애, 도박장애), 관련요인, 정신역동, 행동특성과 물질관련 및 중독장애 대상자 간호
	119	중독 사정 및 간호	중독의 유형, 중독의 유형에 따른 간호
	120	정신사회 건강문제 간호 및 교육	인간의 정신생물학적 이해(뇌신경계의 구조와 기능, 신경전달물질의 기능, 정신질환과 신경전달 물질, 신경계 및 기타 생물학적 관련성, 뇌영상기법)
			인간의 정신심리적 이해(정신역동, Freud의 의식구조, 성격구조, 방어기전 및 정신성적 발달이론, Erikson의 정신사회적 발달이론, Sullivan의 대인관계이론, Mahler의 분리개별화 이론)
			정신치료기법의 이해(약물치료, 기타 생물학적 치료, 환경치료, 활동치료, 개인 정신치료, 집단 정신치료, 가족치료, 인지행동치료 등)
			간호사정, 진단 시 지지제공, 가족의 대처방법에 대한 지지, 만성질환과 일반적인 건강관리에 대한 교육, 정상발달 증진, 현실적인 미래의 목표 설정
			아동의 만성질환이 미치는 영향(부모, 형제자매), 지속되는 스트레스와 주기적인 위기의 대처, 가족구성원의 감정을 조절하도록 돕기, 지지체계확립
			치료적 환경의 개념, 요소 및 치료적 환경 조성
			섭식장애의 정의, 유형(신경성 식욕부진증, 신경성 폭식증, 폭식장애 등), 관련요인, 정신역동, 행동특성 및 섭식장애 대상자 간호
			정상 성반응과 성정체감 발달
			성 변태장애의 정의, 유형(성기능부전, 성별불편감, 변태성욕장애), 관련요인, 행동특성 및 성 관련장애 대상자 간호
	121	정신질환자 간호	조현병 장애의 정의, 관련요인, 행동특성 및 조현병 장애 환자 간호
			기타 정신병적 장애의 정의, 유형(조현양상장애, 단기 정신병적 장애, 조현정동장애, 망상장애 등), 행동특성 및 기타 정신병적 장애 환자 간호
			우울장애의 정의, 유형(주요우울장애, 기분저하장애),

대분류	No	신규간호사 직무항목 (Activity Statement 개정안)	지식항목 (Knowledge statement)
			관련요인, 행동특성 및 우울장애 환자 간호
			양극성 및 관련장애의 정의, 유형(제1형 양극성장애, 제2형 양극성장애, 순환성장애), 관련요인, 행동특성 및 양극성장애 환자 간호
			불안정도 사정 및 불안 수준별 간호
			불안장애의 정의, 유형(공황장애, 광장공포증, 특정공포증, 범불안장애, 사회불안장애), 관련요인, 행동특성 및 불안장애환자 간호
			강박 및 강박 관련 장애의 개념, 유형(강박장애, 신체변형장애 등), 관련요인, 행동특성 및 강박 및 강박 관련 장애 환자 간호
			외상 및 스트레스 관련 장애의 개념, 유형(외상후스트레스장애, 적응장애 등), 관련요인, 행동특성 및 외상 및 스트레스 관련 장애 환자 간호
			신체증상 및 관련 장애의 정의, 유형(신체증상장애, 전환장애, 질병불안장애, 인위성장애), 관련요인, 행동특성 및 신체증상 및 관련 장애 환자 간호
			해리장애의 정의, 유형(해리성 정체성장애, 해리성 기억상실, 이인성/비현실감 장애), 관련요인, 행동특성 및 해리장애 환자 간호
			인격장애의 정의, 유형(편집성, 조현성, 조현형, 반사회성, 경계성, 연극성, 자기애성, 의존성, 강박성, 회피성 인격장애), 관련요인, 정신역동, 행동특성 및 인격장애 환자 간호
	122	아동정신질환자 간호	신경발달장애의 정의, 유형(지적장애, 자폐스펙트럼장애, 주의력결핍 과잉행동장애, 특정학습장애, 틱/뚜렛장애 등), 행동특성 및 신경발달장애 환자 간호
			반응성 애착장애 대상자의 행동특성과 간호
			분리불안장애 대상자의 행동특성과 간호
			파괴적, 충동조절 및 품행장애의 정의, 유형(적대적 반항장애, 품행장애 등), 행동특성 및 파괴적, 충동조절 및 품행장애 대상자 간호
			배설장애 대상자의 행동특성과 간호
	123	임종간호와 교육	사후관리(post-mortem care), 사전연명의료의향서(advance directives)
			임종징후 사정
			품위있는 죽음, 임종시 신체적 간호, 정신적, 영적간호, 가족지지
			사후 신체적 변화, 사체관리, 사별가족지지 / 발달단계에 따른 아동의 죽음에 대한 이해, 아동의 사망과 관련된 윤리적 쟁점: 임종을 앞둔 아동의 간호, 애도(부모, 형제자매), 간호사의 반응
	124	치료적 의사소통 기법	치료적 인간관계와 간호사의 역할
			의사소통의 개념과 치료적, 비치료적 의사소통 기법
			상담기술

8영역. 건강증진 및 유지

대분류	No	신규간호사 직무항목<br>(Activity Statement 개정안)	지식항목<br>(Knowledge statement)
VIII. 건강 증진 및 유지	125	(지역사회)건강교육계획	역학의 정의와 목적, 질병의 역학모형
			보건교육 개념과 보건교육
			학습이론, 건강행위이론, 보건교육 관련이론
			보건교육, 학교보건교육
	126	건강증진 및 유지관리에 대한 정보(예방접종 등) 제공	정신건강증진의 개념, 취약집단 및 정신건강증진사업
			개인/가족/지역사회/인구집단의 건강위험
			건강증진요인 및 위험요인
			인구구조의 유형별 특징
			한국의 인구정책
			생활습관개선을 위한 건강증진사업
	127	고위험 건강 행위의 예방 및 치료에 대한 정보 제공 (금연, 안전한 성행위, 바늘교환)	만성질환관리
			영유아보건사업
			여성보건사업
			노인보건사업
			맞춤형 방문간호사업
			가족계획
			성의 개념과 성건강
	128	성건강 증진간호	피임의 원리와 유형
	129	문화간호	우리나라 가족
			다문화사회의 건강행태와 신념
			문화적 특성에 따른 건강과 질병, 다문화가족의 건강특성
	130	산업과 환경 간호	산업재해관리, 작업환경관리
			환경보건, 환경건강정보
			지역사회/산업장/가정 환경사정
			risk communication의 원칙
			근로자 건강진단
			작업관련성 질환의 예방 및 관리
			산업보건과 산업간호
	131	재난간호	공중보건상의 재난 시 지켜야할 원칙의 적용과 활용
			공중보건법
			위기/재난 시 의사소통체계
			국제재난구조단체
	132	치료계획을 결정하기 위해 가족역동성(가족구조, 결속, 의사소통, 경계, 대처기전) 사정	가족역동성(family dynamics) 사정
	133	가정환경에서 대상자를 관리할 수 있는 역량 (장비, 지역사회 자원) 평가	지역사회 자원 평가
	134	건강위험요인 사정과 교육	학교건강검사
			학교보건교육
			지역사회 정신보건

2) 간호사 국가시험 통합모형에 따른 직무항목별 지식항목수의 교과목별 분포

현재 국가시험은 8개 각 과목별로 보건의약관계법규 20문항, 기본간호학 30문항, 성인간호학 70문항, 지역사회간호학/모성간호학/정신간호학/아동간호학/간호관리학 각 35문항으로 총 295문항으로 구성되어 있다. 이를 기준으로 각 과목별 지식분포의 기준이 되는 백분율(%)을 추정해 볼 수 있다. 즉 간호관리학의 경우 국가시험에서 35문항이 배정되어 있음에도 직무항목별로 구성된 학습목표는 27개로 배정 문제 수 대비 학습목표 비율이 0.8로 학습목표가 배정문제수보다 적은 것으로 나타났다. 반면 모성간호학의 경우 배정문항 대비 학습목표 비율은 가장 높은 8.1로 배정 문제 수에 비해 학습목표가 지나치게 구체적임을 알 수 있다<표 III-3>.

이와 같이 각 과목별 국가시험 문항 수 대비 지식항목 비율이 0.7~8.1까지 다양하게 나타난 이유는 각 교과목의 학습목표/지식항목의 구체성 수준이 과목별로 다르기 때문이다. 따라서 국가시험 문항을 배정할 때 각 과목별로 제시된 지식항목의 구체성 정도를 고려하여 문항수를 지정할 필요성이 제시되었다(송라윤 외, 2018).

<표 III-3> 직무항목별 행위지식(Knowledge statement, KS) 수의 교과목별 분포 (송라윤 외, 2018)

직무 영역	행위지식 항목수	영역별 KS 비중	관리	법규	기본	지역사회	성인	모성	정신	아동
간호관리와 전문성 향상	185	14%	24	6	2	120	1	1	31	0
안전과 감염관리	58	4%	1	3	14	1	5	30	0	4
위험요인 사정	272	21%	1	0	6	0	34	184	0	47
기본간호	93	7%	0	0	38	1	45	0	5	5
생리적 통합유지	237	18%	0	0	18	0	114	26	10	69
약물 및 비경구 요법	55	4%	0	0	21	0	0	24	0	10
심리사회적 통합유지	218	17%	0	0	4	1	0	8	200	5
건강증진 및 유지	185	14%	0	4	0	143	12	9	17	0
**총합**	**1303**	**100%**	**27**	**13**	**103**	**275**	**211**	**282**	**264**	**140**
국가시험 문항수		295	35	20	30	35	70	35	35	35
총 문항수대비 과목 문항수 (%)			12	7	10	12	24	12	12	12
**현행 배정문항 대비 학습목표비율**			**0.8**	**0.7**	**3.4**	**7.9**	**3.0**	**8.1**	**7.5**	**4.0**

3) 간호사 국가시험 통합모형에서 각 과목별 누락된 학습목표

송라윤 외(2018) 연구에서 134개 직무항목과 교과목 학습목표를 연계하였는데, 학

습목표는 각 과목별로 분류기준이 다양하여 의미의 혼돈을 방지하기 위해 과목별 분류방식을 보존한 채로 직무항목과 연결하였다.

연결과정에서 다른 학습목표와 중복되거나 신규간호사의 직무와 관련성이 낮고, 시험과의 연계성이 낮다고 판단된 학습목표는 누락되었다. 각 과목별로 누락된 학습목표 개수는 다음과 같다<표 III-4>.

각 과목별로 누락된 학습목표를 살펴보면, 성인간호학에서는 안위변화와 체액불균형/배뇨장애, 모성간호학에서는 여성건강의 이해, 아동간호학에서는 아동간호학의 개념, 아동의 성장발달과 건강유지, 증진, 아동의 건강회복과 관련된 학습목표가 제외되었고, 지역사회 간호학의 경우 지역사회간호 요구사정, 기본간호학의 경우 산소화요구, 영양요구, 배설요구, 안위요구와 관련된 학습목표가 연계 과정에서 제외되었다. 정신 간호학과 간호관리학 과목의 학습목표는 누락 없이 모두 반영되었다<표 III-5>.

<표 III-4> 각 과목별 누락된 학습목표 개수 (송라윤 외, 2018)

과목	성인 간호학	모성 간호학	아동 간호학	지역사회 간호학	기본 간호학	정신 간호학	간호 관리학
누락 수 (개)	6	2	52	11	11	0	0

<표 III-5> 각 과목별 누락된 학습목표 (송라윤 외, 2018)

과목 (개수)	대분류	중분류	소분류 (학습목표)
성인 (6개)	2. 안위변화	3) 고통	(1) 고통을 사정한다
			(2) 고통과 관련된 간호목표와 중재를 계획한다.
			(3) 고통과 관련된 문제를 중재하고 평가한다.
	4. 체액불균형 /배뇨장애	3) 배뇨장애	(1) 배뇨장애를 사정한다.
			(2) 배뇨장애와 관련된 간호목표와 중재를 계획한다.
			(3) 배뇨장애를 중재하고 평가한다.
모성 (2개)	1. 여성건강의 이해	1) 여성건강개념	(1) 여성건강간호의 개념, 철학, 목적을 설명한다.
			(2) 여성건강간호에 적용되는 이론을 설명한다. (가족이론, 여성주의이론)
아동 (52개)	1.아동간호학의 개념	1) 아동간호학의 개념	(1) 아동간호의 철학을 설명한다.
			(2) 아동의 주요 건강 지표를 설명한다.
			(3) 아동의 건강에 영향을 미치는 요인을 설명한다.
			(4) 아동간호사의 역할을 설명한다.
		2) 아동과 가족	(1) 부모역할을 설명한다.
			(2) 가족이 아동의 성장발달에 미치는 영향을 설명한다.
			(3) 가족의 다양한 특성이 아동에게 미치는 영향을 설명한다.
			(4) 아동 학대를 설명한다.
		3) 아동간호의	(1) 건강력을 사정한다.

과목 (개수)	대분류	중분류	소분류 (학습목표)
		기본원리	(2) 생리적 상태를 사정한다.
			(3) 신체의 각 기관을 사정한다.
			(4) 아동에게 입원과 질병이 갖는 의미를 설명한다.
			(5) 아동과 가족에게 의사소통기술을 수행한다.
			(6) 입원아동에게 기본간호를 수행한다.
			(7) 입원 중 안전관리를 수행한다.
			(8) 심폐소생술을 수행한다.
			(9) 퇴원간호를 수행한다.
	2.아동의 성장발달과 건강유지, 증진	1) 아동의 성장발달 특성	(1) 아동의 성장과 발달을 설명한다.
			(2) 성장발달에 영향을 미치는 요인을 설명한다.
			(3) 발달단계별 성장의 특성(키, 몸무게, 머리둘레, 가슴둘레, 배둘레, 외모, 체형, 신체 각 기관의 성장, 감각, 생리적 발달)을 설명한다.
			(4) 발달단계별 발달의 특성(운동능력, 심리사회성, 인지, 언어, 성, 영성, 도덕, 신체상, 자아개념)을 설명한다.
			(5) 아동의 기질을 설명한다.
		2) 아동의 성장발달 사정	(1) 신체적 성장 상태를 사정한다.
			(2) 발달검사를 수행한다.
		3) 성장발달 단계별 건강 유지, 증진	(1) 발달단계별 활동과 휴식의 특성을 설명한다.
			(2) 예방접종의 종류와 시기를 설명한다.
			(3) 발달단계별 영양과 식습관 교육을 수행한다.
			(4) 발달단계별 성교육을 수행한다.
			(5) 발달단계별 안전사고의 예방과 대처 교육을 수행한다.
	3. 아동의 건강회복	1)고위험신생아와 가족의 간호	(9) 고위험 신생아의 부모에게 부모역할 증진 교육을 수행한다.
		2) 호흡기능장애 아동의 간호	(1) 아동기 호흡기의 특성을 설명한다.
			(2) 호흡기능을 사정한다(병력, 신체사정, 진단검사).
		3) 소화기능장애 아동의 간호	(1) 아동기 소화기관의 특성을 설명한다.
			(2) 소화기능을 사정한다(병력, 신체사정, 진단검사).
			(3) 수분전해질 불균형(구토, 설사, 탈수) 아동에게 간호과정을 적용한다.
		5) 순환기능장애 아동의 간호	(1) 아동기 순환기의 특성을 설명한다.
			(2) 순환기능을 사정한다(병력, 신체사정, 진단검사).
			(3) 선천심장병의 병태생리를 설명한다.
		6) 혈액기능장애 아동의 간호	(1) 아동기 혈액의 특성을 설명한다.
			(2) 혈액기능을 사정한다(병력, 신체사정, 진단검사).
		8) 피부기능장애 아동의 간호	(1) 아동기 피부의 특성을 설명한다.
		9) 뇌기능장애 아동의 간호	(1) 아동기 뇌의 특성을 설명한다.
			(2) 뇌기능을 사정한다(병력, 신체사정, 진단검사).
		10)운동기능장애 아동의 간호	(1) 아동기 근골격의 특성을 설명한다.
			(2) 근골격기능을 사정한다(병력, 신체사정, 진단검사).
		11) 비뇨생식 기능장애 아동의 간호	(1) 아동기 비뇨생식기의 특성을 설명한다.
			(2) 비뇨생식기능을 사정한다. (병력, 신체사정, 진단검사)
		12)내분비기능장애 아동의 간호	(1) 아동기 내분비의 특성을 설명한다.
			(2) 내분비기능을 사정한다(병력, 신체사정, 진단검사).

과목 (개수)	대분류	중분류	소분류 (학습목표)
		14) 종양아동과 호스피스 완화 간호	(1) 아동기 종양의 특성을 설명한다.
		15) 만성질환 및 장애 아동의 간호	(5) 지체장애 아동에게 간호과정을 적용한다.
			(8) 만성질환 및 장애아동의 가족 간호를 수행한다.
지역 (11개)	1. 지역사회 간호요구 사정	1) 국내외 보건정책 이해_(3)지역사회 간호의 역사	서양 근대 공중보건의 발전이 간호에 미친 영향을 설명한다.
			영국 방문간호의 발전과 나이팅게일이 이에 미친 영향을 설명한다.
			미국 방문간호사업의 발전을 설명한다.
			20세기 이후 보건의료 환경의 변화가 지역사회간호의 발전에 미친 영향을 설명한다.
			일차보건의료와 건강증진 개념이 지역사회간호에 미친 영향을 설명한다.
			일제강점기 주요 보건간호사업에 대하여 설명한다.
			우리나라 현대 보건의료환경의 변화가 지역사회간호에 미친 영향을 설명한다.
			우리나라 현대 보건의료 제도의 주요 변화가 지역사회간호에 미친 영향을 설명한다.
			우리나라 지역사회간호의 중요 이정표가 된 사건들을 통해 지역사회간호의 발전을 설명한다.
		1) 국내외 보건정책 이해_(5)국제보건의 이해	국제 환경의 상황에서 간호 및 보건의료서비스를 제공한다.
		2) 역학지식 및 통계기술 실무 적용_(2) 질병의 자연사와 예방수준	질병의 자연사를 설명한다.
기본 (11개)	1. 산소화 요구	1) 산소화 요구 사정	(1) 활력징후 사정: 혈압의 측정원리를 설명한다.
			(2) 산소화 요구 사정: 호흡과정을 설명한다.
	2. 영양요구	2) 영양요구 사정	(1) 영양 개요: 영양소별 대사과정을 설명한다.
	3. 배설요구	1) 배설요구 사정	(1) 배뇨 사정: 배뇨기전을 설명한다. 배뇨장애를 사정한다.
			(2) 배변 사정: 배변기전을 설명한다. 배변요구를 사정한다.
	5. 안위요구	1) 수면 사정 및 간호	(1) 수면 사정: 수면-각성주기에 따른 특성을 설명한다.
		2) 체온 사정 및 조절 간호	(2) 체온 사정: 체온조절기전을 설명한다.<br>체온의 변화를 설명한다.<br>열과 냉의 생리적 효과를 설명한다.

바. 보건의료환경 변화에 따른 간호사 국가시험 및 면허관리체계 개선방안 (탁영란 외, 2019).

탁영란 외(2019) 연구에서는 교과목 통합모형에서 신규간호사의 역량에 기반을 둔 국가시험의 출제기준표 작성을 위한 핵심역량 영역별 가중치를 제시하고자 하였다.

1) 선행연구의 신규간호사 직무기반 직무항목의 직무 영역별 비중

먼저 신규간호사 직무기반 직무항목의 영역별 비중을 고려하였다<표 III-6>.

즉 김영경 외(2018) 연구의 205개 Activity statement에서는 선행연구(박인숙 외, 2014, 강소영 외, 2015)에 비해 ‘간호관리와 전문성 향상’, ‘안전과 감염관리’, ‘위험요인 사정’, ‘기본간호’, ‘생리적 통합유지’ 항목의 비중이 증가하였고, 심리사회적 통합유지, 건강증진 및 유지 항목의 비중은 감소하였다. 이는 도출된 Activity statement에 신규간호사의 직무 및 교육을 실시하는 대학의 의견이 충분히 반영되어야 한다는 선행연구의 제한점이 반영된 것이라고 할 수 있다. 한편 송라윤 외(2018)의 134개 Activity statement 개정안에서는 ‘간호관리와 전문성 향상’ 22개(16.4%), ‘안전과 감염관리’ 3개(2.2%), ‘잠재적 위험요인’ 28개(20.9%), ‘기본간호’ 19개(14.2%), ‘생리적 통합유지’ 36개(26.9%), ‘약물과 비경구요법’ 7개(5.2%), ‘심리사회적 통합유지’ 9개(6.7%), ‘건강증진 및 유지’ 10개(7.5%) 비중을 차지한다. 이는 Activity statement의 추상적 수준이 모든 영역에서 동일하지 않다는 점과 Activity statement가 많이 서술되어 있다고 해서 각 영역의 직무중요도를 반영하다고 보기 어렵다는 점을 고려할 때 신규간호사 직무기반의 영역별 배정 Activity statement만으로 가중치를 고려하는 것은 부족하다고 판단되었다.

<표 III-6> 선행연구의 직무영역별 비중 차이 비교

(단위 : %)

선행 연구	박인숙 외 (2014)	강소영 외 (2015)	김영경 외 (2018)	송라윤 외 (2018)	
직무 영역	출제내용 (308개)	직무능력 (372개)	Activity statement (205개)	Activity statement (개정안) (134개)	Knowledge statement (1,303개)
간호관리와 전문성향상	12	15	18	16.4	14
안전과 감염관리	6	8	10	2.2	4
위험요인 사정	17	15	19	20.9	21
기본간호	11	10	14	14.2	7
생리적 통합유지	9	11	17	26.9	18
약물 및 비경구 요법	10	7	9	5.2	4
심리사회적 통합유지	12	17	5	6.7	17

선행 연구	박인숙 외 (2014)	강소영 외 (2015)	김영경 외 (2018)	송라윤 외 (2018)	
직무 영역	출제내용 (308개)	직무능력 (372개)	Activity statement (205개)	Activity statement (개정안) (134개)	Knowledge statement (1,303개)
건강증진 및 유지	24	18	9	7.5	14

2) 신규간호사 직무 영역별 지식항목의 교과목별 분포

탁영란 외(2019)의 연구에서는 신규간호사 역량을 평가하기 위해 제시된 직무영역별 직무항목(Activity statement) 134개를 각 교과목 교육목표와 매칭하여 최종적으로 제시된 행위지식(Knowledge statement)은 481개로 나타났다.

각 직무항목수와 행위지식에 대한 교육목표의 배정은 유사한 영역도 있지만, 영역에 따라 추상도와 임상적 중요도에서 차이가 있었다. 예를 들어 간호관리와 전문성 향상 영역의 경우 직무항목은 총 134개 중 22개로 16.4%가 포함되었고, 행위지식은 총 481개 중 79개로 16.4%로 직무항목수와 행위지식수가 유사한 분포를 보였다. 그러나 위험요인 사정 영역에서는 직무항목이 10개(7.5%)로 구성되어 있으나 행위지식은 93개(19.3%)가 분포되어 직무항목 수만을 기준으로 할 경우 출제가중치가 과소평가될 우려가 있다. 반면 기본간호영역은 직무항목은 28개(20.9%)가 배정되어 있으나 행위지식에서는 60개(12.4%)가 배정되어 있어 직무항목만을 기준으로 할 경우 출제가중치가 과대평가될 우려를 보인다.

따라서 신규간호사 직무영역별 출제기준을 위한 영역별 최종 가중치를 결정하기 위해 각 영역별 행위지식의 분포를 분석하였다.

<표 III-7>에 의하면 간호관리 과목에서 추출된 행위지식은 총 41개로 주로 간호관리와 전문성 향상영역에 31개, 안전과 감염관리에 10개 배정되어 있다. 국가고시 문항으로 간호관리에 35문제가 배정되어 있으므로 간호관리 행위지식 41개의 가중치(35/45)는 0.85이다. 이와 같은 방법으로 각 교과목의 가중치를 산출하였다.

<표 III-7> 간호사 직무 영역별 지식항목(교과목 교육목표)의 배정표 (탁영란 외, 2019)

직무 영역	관리	기본	모성	법규	성인	아동	정신	지역사회	합계
간호관리와 전문성 향상	31	2		20			4	22	79
건강증진 및 유지			3					34	37
기본간호		31			26	3			60
생리적 통합유지		13	12	2	68	28	1	6	130
심리사회적 통합유지					3		40		43
안전과 감염관리	10	8		5	2				25
약물 및 비경구 요법		13		1					14
위험요인 사정		8	32		12	41			93
**합계**	**41**	**75**	**47**	**28**	**111**	**72**	**45**	**62**	**481**
국가시험 문항 분포	35	30	35	20	70	35	35	35	295
**가중치**	**0.85**	**0.40**	**0.74**	**0.71**	**0.63**	**0.49**	**0.78**	**0.56**	**0.61**

3) 직무영역별 배정 교육목표에 근거한 영역별 총 가중치 추정

각 직무 영역별로 구성된 교과목의 가중치를 계산한 후 이를 통합하여 직무 영역별 가중치를 추산하였다.

<표 III-8>에 의하면 '간호관리와 전문성 향상' 영역에서는 5개 교과목 즉 간호관리학, 기본간호학, 간호법규, 정신간호학, 지역사회간호학의 행위지식이 포함되어 있다. 따라서 각 과목별로 행위지식 수와 배정가중치를 곱하여 가중치를 계산한 후 5개 과목의 가중치를 합산하여 '간호관리와 전문성 영역'의 총 가중치를 구하였다.

<표 III-8> 직무영역별 배정 교육목표에 근거한 영역별 총 가중치 (탁영란 외, 2019)

직무 영역	교과목	행위지식수	배정가중치	지식수*가중치	총가중치
간호관리와 전문성 향상	관리	31	0.85	26.35	**56.79**
	기본	2	0.40	0.80	
	법규	20	0.71	14.20	
	정신	4	0.78	3.12	
	지역	22	0.56	12.32	

건강증진 및 유지	모성	3	0.74	2.23	**21.43**
	지역	34	0.56	19.19	
기본간호	기본	31	0.40	12.40	**30.25**
	성인	26	0.63	16.40	
	아동	3	0.49	1.46	
생리적 통합유지	기본	13	0.40	5.20	**76.22**
	모성	12	0.74	8.94	
	법규	2	0.71	1.42	
	성인	68	0.63	42.88	
	아동	28	0.49	13.61	
	정신	1	0.78	0.78	
	지역	6	0.56	3.39	
심리사회적 통합유지	성인	3	0.63	1.89	**33.09**
	정신	40	0.78	31.20	
안전과 감염관리	관리	10	0.85	8.50	**16.51**
	기본	8	0.40	3.20	
	법규	5	0.71	3.55	
	성인	2	0.63	1.26	
약물 및 비경구요법	기본	13	0.40	5.20	**5.91**
	법규	1	0.71	0.71	
위험요인 사정	기본	8	0.40	3.20	**54.53**
	모성	32	0.74	23.83	
	성인	12	0.63	7.57	
	아동	41	0.49	19.93	
**총합**		**481**		**295**	**295**

이와 같은 단계를 거쳐 도출된 근거자료를 연구진에 의해 검토, 수렴된 의견을 반영하여 간호사 국가시험 출제기준표가 제시되었다<표 III-9>.

그러나 8개 직무영역에서 추출된 지식항목을 최종 출제기준으로 확정하기 위해서는 추후 신규간호사에게 적용되는 임상적 중요성을 고려하기 위한 임상타당도 연구가 요구된다고 하였다(탁영란 외, 2019).

<표 III-9> 직무영역에 따른 직무항목과 행위지식 분포에 기반한 최종 가중치. 간호사 국가시험 출제기준표 (탁영란 외, 2019)

단위 : n(%)

직무 영역	NCLEX-RN 출제비율	직무항목수	행위지식수	교과목별 가중치 적용	최종안
간호관리와 전문성 향상	(20)	22(16.4)	79(16.4)	53.8(19.3)	**(18)**
안전과 감염관리	(12)	7(5.2)	25(5.2)	16.5(5.6)	**(10)**
위험요인 사정	(9)	10(7.5)	93(19.3)	54.5(18.5)	**(16)**
기본간호	(12)	28(20.9)	60(12.4)	30.3(10.3)	**(10)**
생리적 통합유지	(14)	19(14.2)	130(27.0)	76.2(25.9)	**(20)**
약물 및 비경구 요법	(15)	9(6.7)	14(2.9)	5.9(2.0)	**(10)**
심리사회적 통합유지	(9)	36(26.9)	43(8.9)	33.1(11.2)	**(9)**
건강증진 및 유지	(9)	3(2.2)	37(7.7)	21.3(7.2)	**(7)**
**총합**	**100**	**134**	**481**	**295(100)**	**100**

2. 국외 간호사 국가시험 관련 문헌고찰

가. 미국과 캐나다

캐나다의 간호사 면허시험은 2014년 10월 1일 시험을 마지막으로 폐지되었고, 2015년부터 캐나다의 10개 주를 대상으로 미국의 NCLEX-RN 시험을 채택하였다.

시험방법은 CAT(Computerized Adaptive Testing)으로 진행되며 실기시험은 시행하고 있지 않다.

미국의 간호사 국가시험(NCLEX-RN: the National Council Licensure Examination for Practical Nurses)은 신규간호사가 안전하고 효과적인 간호활동에 참여할 수 있는 최소한의 능력을 선별하기 위해 요구되는 기본 지식과 기술, 능력을 평가하며 응시자의 특정능력에 맞춰 가변길이 형식으로 적용된다.

1978년 설립된 비영리단체인 The National Council of State Board Nursing(NCSBN)에서 시험을 주관하고, 각 과목별 문제가 아닌 통합시험과목으로 운영하며, 2002년부터 3년마다 신규간호사 대상으로 직무분석 연구를 시행하여 신규간호사가 기본적으로 수행해야 하는 간호활동(nursing activity)을 제시하고 Activity statement로 명명하여 평가기준으로 활용하고 있다.

1) 시험문제 구성과 분포

시험문제는 미국의 간호대학에서 교육하는 5개 과목(내과, 외과, 산부인과, 정신과, 소아과)의 Nursing Model의 통합적 사고를 요구하는 문제들과 인간의 기본욕구에 대한 포괄적 접근이 포함된 문제들이 출제되고 있다.

NCLEX-RN 시험은 대상자의 요구에 따라 4개 범주로 구성되며, 그 중 2개 범주는 하위영역으로 구분되어 있다. 즉 4개 범주는 '안전하고 효율적인 간호환경(Safe and Effective Care Environment)', '건강증진 및 유지(Health Promotion and Maintenance)', '심리사회적 통합(Psychosocial Integrity)', '생리적 통합(Physiological Integrity)'이다. 이 중 '안전하고 효율적인 간호환경'의 하위영역으로 '간호 관리(Management of Care)'와 '안전과 감염관리(Safety and Infection Control)'가 있다. 또한 '생리적 통합'의 하위영역으로 '기본간호와 안위(Basic Care and Comfort)', '약물과 비경구 치료(Pharmacological and Parenteral Therapies)', '잠재위험의 감소(Reduction of Risk Potential)', '생리적 적응(Physiological Adaptation)'이 포함되어 있다.

시험을 주관하는 NCSBN에서는 3년마다 시행되는 직무분석 결과에 따라 각 범주별 출제 비율을 조정하여 수정된 시험계획을 발표한다(NCSBN, 2019).

<표 III-10>에서는 NCLEX-RN 시험 문항 출제를 위한 대상자 요구에 따라 분류된 범주와 각 범주별 출제 비율을 나타내었다.

<표 III-10> NCLEX-RN 문항출제의 직무분석 분류와 출제 비율

Client Needs	Percentage of items from each category/subcategory	
	2008(%)	2019(%)
**1. Safe and Effective Care Environment (안전하고 효율적인 간호환경)**		
1-1. Management of Carer (간호관리)	13-19	17-23
1-2 Safety and Infection Control (안전과 감염관리)	8-14	9-15
**2. Health Promotion and Maintenance (건강증진 및 유지)**	6-12	6-12
**3. Psychosocial Integrity (심리사회적 통합)**	6-12	6-12
**4. Physiological Integrity (생리적 통합)**		
4-1. Basic Care and Comfort (기본간호와 안위)	6-12	6-12
4-2. Pharmacological and Parenteral Therapies (약물과 비경구 치료)	13-19	12-18
4-3. Reduction of Risk Potential (잠재위험의 감소)	13-19	9-15
4-4. Physiological Adaptation (생리적 적응)	11-17	11-17

2) NCSBN의 Knowledge statement

NCSBN은 3년마다 신규간호사가 간호현장에서 직무를 수행하기 위해 필요로 하는 지식을 도출하고 검증하는 연구를 시행하고 있다. 2018년에 보고된 'Report of Findings from the 2017 RN Nursing Knowledge Survey'연구에서는 295개 Knowledge statements를 도출하고 타당성 조사를 실시하였다<표 III-11>. 도출된 지식 중에서 신규간호사가 생각하는 가장 중요한 지식은 'Airway Management', 'Medication Administration', 'Vital Signs', Medication Safety', 'Change Client Condition'으로 나타났다.

NCSBN에서는 8개의 출제범주 내의 142개 Activity statements를 295개 Knowledge statements와 연계하여 문항개발의 근거를 마련하고 구체적인 지침으로 활용하고 있다.

<표 III-11> NCLEX-RN Knowledge statements (NCSBN, 2018)

No	Knowledge statement
1	Abnormal Test Results 비정상 검사결과
2	Activities of Daily Living (ADLs) 일상생활활동
3	Acute Conditions 급성 상태
4	Admission Process 입원 절차
5	Advance Directives 사전연명의료의향서
6	Adverse Reactions 이상 반응(유해 반응)
7	Age Specific Care 연령 특이적 간호
8	Airway Management 기도 관리
9	Alteration in Elimination 배설 장애
10	Alternatives to Restraints 억제대 사용 대체방안
11	Arterial Line Maintenance 동맥관(A-line) 유지
12	Arterial Line Monitoring 동맥관(A-line) 관찰
13	Aseptic Technique 무균술
14	Aspiration Precautions 기도흡인 주의사항
15	Assessment Techniques 사정 기술
16	Assessment Tools 사정도구
17	Assistive Devices 보조기구
18	Auditory Distortions 청각 왜곡
19	Basic Cardiac Rhythms 정상 심장리듬
20	Behavioral Management Techniques 행동 관리 기술
21	Biohazardous Material Handling 생물학적 유해물질 관리(취급)
22	Blood Product Administration 혈액제제 투여
23	Blood Products 혈액제제
24	Breathing Techniques 호흡법
25	Care and Coordination 간호와 조정력
26	Care Giver Resources 간병인력 자원
27	Care Plan Process 치료계획 과정
28	Catheter Care 카테터 관리
29	Central Venous Access Devices 중심정맥관 장치

No	Knowledge statement
30	Chain of Command 보고체계
31	Change Management 변화관리
32	Changes in Client Condition 대상자 상태 변화
33	Chronic Conditions 만성 상태
34	Circulation 순환
35	Circulation Devices 순환 장치
36	Client Acuity 대상자 예민성
37	Client Advance Directives 대상자 사전연명의료의향서
38	Client Advocacy 대상자 옹호
39	Client Allergies 대상자 알러지
40	Client Appropriate Interventions 대상자에게 적합한 중재
41	Client Assessment 대상자 사정
42	Client Background 대상자 배경
43	Client Body Image 대상자 신체상
44	Client Care for Adolescents 청소년기 대상자 간호
45	Client Care for Age 18 through 64 Years 18세~64세 성인 간호
46	Client Care for Age 65 Years and Over 65세 이상 노인 간호
47	Client Care for Infants 영아 간호
48	Client Care for Newborns 신생아 간호
49	Client Care for Preschool Aged Child 학령전기 아동 간호
50	Client Care for School Aged Child 학령기 아동 간호
51	Client Care for Toddlers 유아 간호
52	Client Centered Care 대상자 중심 간호
53	Client Confidentiality 대상자 비밀유지
54	Client Consent 대상자 동의
55	Client Demographic Considerations (e.g., Age, Gender, Community)<br>인구통계학적 고려(예, 나이, 성별, 지역공동체)
56	Client Dignity 대상자 존엄
57	Client Disease Process 대상자 질병과정
58	Client Education 대상자 교육
59	Client Hand-off Process 대상자 인수인계
60	Client Health History 대상자 건강력
61	Client Health Literacy 대상자 건강정보 이해능력
62	Client Identification 대상자 확인
63	Client Learning Assessment 대상자 학습능력 사정
64	Client Needs Assessment 대상자 요구 사정
65	Client Occupational Factors 대장자의 직업적 요인
66	Client Plan of Care 대상자 치료 계획
67	Client Positioning 대상자 체위
68	Client Privacy 대상자 사생활 보호
69	Client Psychosocial Factors 대상자의 심리사회적 요인
70	Client Religion and Spirituality Considerations 대상자의 종교, 영적 고려

No	Knowledge statement
71	Client Response to Plan of Care 치료계획에 대한 대상자 반응
72	Client Response to Treatment 치료에 대한 대상자 반응
73	Client Rights and Responsibilities 대상자의 권리와 의무
74	Client Risk Assessment 대상자 위험 사정
75	Client Risk Factors 대상자 위험요인
76	Client Safety 대상자 안전
77	Client Safety Goals 대상자 안전 목표
78	Client Self Determination 대상자 자기 결정
79	Client Status 대상자 상태
80	Client Support System 대상자 지지체계
81	Clinical Judgment 임상적 판단
82	Cognitive Distortions 인지적 왜곡
83	Communication Barriers 의사소통의 장해
84	Communication Strategies 지역사회 전략
85	Community Health Education 지역사회 건강교육
86	Community Needs Assessment 지역사회 요구 사정
87	Community Resources 지역사회 자원
88	Competency of Delegatee 위임자의 역량
89	Complementary Therapies 보완대체 요법
90	Complications of Labor 분만 합병증
91	Comprehensive Health Assessments 포괄적 건강사정
92	Conflict Management Strategies 갈등 관리 전략
93	Contraindications 금기사항
94	Controlled Substances 규제 약물
95	Coping Mechanisms 대처 기전
96	Coping Techniques 대처 기술
97	Cost-effective Care 비용 효과적 간호
98	Crisis Intervention 위기 중재
99	Cultural Considerations 문화적 고려
100	Death and Dying 죽음과 임종
101	De-escalation Techniques 하강기법(단계적 축소기법)
102	Delegation of Care 간호 위임
103	Delegation of Responsibilities 책임 위임
104	Developmental Stages and Milestones 발달 단계와 발달 과정
105	Device Assessment 장비 사정
106	Device Complications 장치 합병증
107	Diagnostic Tests and Procedures 진단 검사와 절차
108	Discharge Process 퇴원 절차
109	Disease Process 질병 과정
110	Dosage Calculations 용량 계산
111	Drainage Devices 배액 장치

No	Knowledge statement
112	Dressing Changes 드레싱 교환
113	Early Client Mobilization 대상자 조기이상
114	Electronic Health Records 전자의무기록
115	Elimination 배설
116	Elimination Devices 배설 장치
117	Emergency Intervention 응급상황 중재
118	Emergency Response Plan 응급상황 대처 계획
119	Emergency Response Roles and Responsibilities 응급상황 대처 시 역할 및 책임
120	End-of-life Care 임종 간호
121	Enteral Feeding Tubes 장관 급식 튜브
122	Enteral Tube Feedings 장관 튜브 급식
123	Environmental Hazards 환경위험
124	Environmental Safety 환경안전
125	Equipment Safety 장비안전
126	Ergonomic Equipment 신체역학을 이용한 기기(인체공학적 기기)
127	Ethical and Legal Considerations 윤리적 법적 고려
128	Ethical Dilemmas 윤리적 딜레마
129	Evidence Based Practice 근거기반실무
130	Expected and Unexpected Outcomes 예상된 결과, 예상치 못한 결과
131	Expected and Unexpected Treatment Outcomes 예상된, 예상치 못한 치료결과
132	Family Dynamics 가족 역동성
133	Fistula Care 동정맥루 관리
134	Fluid and Electrolytes 수분과 전해질
135	Focused Assessment 초점 사정(집중 사정)
136	Gastrointestinal Tube Insertion 위장관 튜브 삽입
137	Gastrointestinal Tube Maintenance 위장관 튜브 유지
138	Gastrointestinal Tube Removal 위장관 튜브 제거
139	Hazardous Material Handling 유해물질관리
140	Health Promotion Behavior 건강증진행위
141	Hemodialysis 혈액투석
142	Hemodynamics 혈류역학(혈역학)
143	Hemostasis 울혈
144	High-risk Health Behaviors 고위험 건강행위
145	High-risk Medications 고위험 약물
146	Immunization/Vaccination Recommendations 예방접종 권장
147	Incident and Error Reporting 사건, 오류 보고
148	Incontinence Care 실금 관리
149	Infection Control 감염 관리
150	Information Technology 정보기술
151	Infusion Delivery Devices 주입장치
152	Initial Care for Newborn 신생아 초기 간호

No	Knowledge statement
153	Injury Risk Precautions 상해위험 주의사항
154	Intake and Output 섭취량과 배설량
155	Interpersonal Communication 상호간 의사소통
156	Invasive Line Care 침습적 라인 관리
157	Invasive Procedures 침습적 처치(시술)
158	Irrigation 세척
159	Irrigation Techniques 세척 기법
160	Isolation Precautions 격리 주의사항
161	Life Changes 삶의 변화
162	Life Transitions 삶의 전환
163	Mandatory Reporting Requirements 의무 보고 요건
164	Material Safety Data Sheets (MSDS) 물질안전보건자료
165	Medical Terminology 의학용어
166	Medication Administration 약물 투여
167	Medication Diversion 약물 교란
168	Medication Interactions 약물상호작용
169	Medication Reconciliation 약물 조정
170	Medication Reconciliation Process 약물 조정 과정
171	Medication Safety 약물 안전
172	Medication Storage 약물 보관
173	Medication Titration 약물 적정화
174	Moderate Sedation 중등도 진정
175	Multidisciplinary Teams 다학제팀
176	New Parent Education 예비부모 교육
177	Non-pharmacological Comfort Measures비약물적 안위 제공
178	Nurse Practice Act 간호사 실천법
179	Nursing Code of Ethics 간호윤리강령
180	Nursing Practice Guidelines 간호실무지침
181	Nutrition Assessment 영양 사정
182	Oral Hygiene 구강 위생
183	Order Verification Process 처방 확인절차
184	Orthopedic Devices 정형외과적 장치
185	Ostomy Care 인공개방 간호
186	Oxygen Delivery Systems 산소전달 시스템
187	Pacing Devices 심박동 조절장치
188	Pain Management 통증 관리
189	Palliative Care 완화 치료
190	Parenteral Nutrition 비경구 영양
191	Pathophysiology 병태생리
192	Percutaneous Feeding Tubes 경피적 급식튜브
193	Peripheral Intravenous Line Insertion 말초정맥주사 삽입

No	Knowledge statement
194	Peripheral Intravenous Line Maintenance 말초정맥주사 유지
195	Peripheral Intravenous Line Removal 말초정맥주사 제거
196	Peritoneal Dialysis Care 복막투석 간호
197	Personal Protective Equipment (PPE) 개인보호장비
198	Pharmacology 약리학
199	Phototherapy 광선 치료
200	Physical Impairment 신체 장애
201	Physical Stressors 물리적 스트레스 요인
202	Policy and Procedures 정책과 절차
203	Positioning Devices 체위 장치
204	Postmortem Care 사후 관리
205	Postoperative Care 수술 후 간호
206	Postoperative Education 수술 후 교육
207	Postpartum Care 산후 관리
208	Postpartum Complications 산후 합병증
209	Practice Outcome Metrics 실무결과지표
210	Prenatal Care Education 태아 간호 교육
211	Prenatal Complications 태아 합병증
212	Preoperative Care 수술 전 간호
213	Preoperative Education 수술 전 교육
214	Preventative Care 예방적 간호
215	Prioritization of Care 우선순위 결정
216	Process of Consent 동의서 작성 절차
217	Professional Competency 전문역량
218	Program Planning 프로그램 기획
219	Proper Body Mechanics 적정 신체 기전
220	(Health care)Provider Credentials 치료제공자(의사) 자격
221	(Health care)Provider Orders 치료제공자(의사) 처방
222	(Health care)Provider Scopes of Practice 체료제공자(의사) 실무범위
223	Psychological Stressors 심리적 스트레스 요인
224	Psychosocial Assessment 심리사회적 사정
225	Psychosocial Factors of Substance Abuse/ Dependency 약물남용/약물 의존의 심리사회적 요인
226	Psychosocial Health Issues 심리사회적 건강 이슈
227	Pulmonary Hygiene Techniques 흉부물리요법
228	Quality Improvement Process 의료의 질 향상
229	Range of Motion 관절가동범위
230	Referral Process 의뢰 절차
231	Regulatory Guidelines 규제지침(관리지침)
232	Religious and Spiritual Considerations 종교적 영적 고려
233	Renal Assessment 신장 사정

No	Knowledge statement
234	Renal Replacement Therapy 신장 대체 요법(신대체 요법)
235	Reporting Processes 보고체계
236	Resource Availability 자원 이용 가능성
237	Restraints 억제법
238	Risk Assessment (e.g., Fall, Suicide, Pressure, Ulcer) 위험사정(낙상, 자살, 욕창)
239	Risk Assessment for Abuse and Neglect 학대와 방임에 대한 위험사정
240	Risk Management 위험 관리
241	Safe Client Handling 안전하게 대상자를 다룸
242	Safety Considerations 안전 고려사항
243	Safety Precautions 안전 주의사항
244	Scope of Practice 실무 범위
245	Screening Assessments 스크리닝 사정
246	Security Plan 보안 계획
247	Seizure Protocol 발작 프로토콜
248	Self-awareness 자기 인식
249	Sequential Compression Devices 순차적 압박장치
250	Signs and Symptoms of Abuse 학대 징후와 증상
251	Signs and Symptoms of Complications 합병증 징후와 증상
252	Signs and Symptoms of Dependency 의존 징후와 증상
253	Signs and Symptoms of Neglect 방임 징후와 증상
254	Signs and Symptoms of Substance Abuse 약물남용 징후와 증상
255	Signs and Symptoms of Substance Overdose 약물과다 징후와 증상
256	Signs and Symptoms of Withdrawal 금단 징후와 증상
257	Skin Assessment 피부 사정
258	Skin Integrity 피부 통합성
259	Sleep/Rest Pattern 수면/휴식 양상
260	Specimen Collection 검체 채취
261	Specimen Handling 검체 취급(관리)
262	Staff Education 직원 교육
263	Staff Safety 직원 안전
264	Staff Support 직원 지지
265	Staff to Client Ratio 직원 대 대상자 비율
266	Stages of Grief 슬픔 단계
267	Stages of Labor 분만 단계
268	Standard Precautions 표준주의지침
269	Sterile Technique 무균술
270	Stoma Care 장루 간호
271	Suction Equipment 흡인 장비
272	Suction Techniques 흡인 기법
273	Telemetry Equipment 원격측정 장비
274	Telemetry Lead Placement 원격측정 심전극 위치

No	Knowledge statement
275	Therapeutic Environment 치료적 환경
276	Therapeutic Interventions 치료적 중재
277	Thermoregulation Techniques 온도 조절 기법
278	Time Management Strategies 시간 관리 전략
279	Tissue Perfusion 조직 관류
280	Tracheostomy Care 기관절개관 간호
281	Transfer Process 전동 절차
282	Tube Placement Verification 튜브 위치 확인
283	Types of Dressings 드레싱 유형
284	Urinary Catheter Insertion 도뇨관 삽입
285	Urinary Catheter Maintenance 도뇨관 유지
286	Urinary Catheter Removal 도뇨관 제거
287	Venipuncture 정맥천자
288	Venous Access Devices 정맥주사 장치
289	Venous Thromboembolism Prophylaxis 혈전색전증 예방
290	Ventilator Equipment 인공호흡기 장비
291	Verbal and Nonverbal Cues 언어적, 비언어적 단서
292	Visual Distortions 시각적 왜곡
293	Vital Signs 활력징후
294	Wound Assessment 상처 사정
295	Wound Care 상처 간호

나. 일본

일본 간호사 국가시험은 후생노동성에서 주관하며, 지필평가 형식으로 1회/년 진행되고 있으며, 실기시험은 시행하지 않는다.

시험 과목은 기초의학(인체의 구조와 기능, 질병의 성립과 회복 촉진), 기초 간호학, 성인간호학 총론, 노년 간호학, 소아 간호학, 모성 간호학, 정신간호학, 재택 간호학, 간호의 통합과 실천, 건강지원과 사회보장제도이다.

그러나 시험은 교과목별로 나누어 보는 것이 아니고 세 가지 유형의 문제로 통합출제 된다. 즉 필수문제 50문항, 일반문제 130문항, 상황설정문제 60문항으로 총 240문항으로 구성되어 있다.

필수문제는 주로 일본에 대한 통계관련 문제로 총인구수, 노인/청년비율, 노령화 지수, 출산율, 사망률, 사망 원인/질병순위, 남녀 흡연율, 남녀 나트륨 섭취 권장량 등 건강지표가 출제된다. 또한 간호사가 반드시 알아야하는 기본간호에 필요한 지식, 해부생리와 건강 장애, 간호대상자와 간호활동의 장에 대한 내용이 출제된다.

일반문제는 성인, 노인, 여성, 소아 간호와 지역, 법률 등 모든 교과목의 내용이

통합되어 출제된다.

상황설정문제는 활력징후, 검사지표 등이 포함된 지문이 주어지고 그 상황에 알맞은 답을 찾는 문제로 실제 임상과 비슷한 상황이 주어진다.

시험은 오전과 오후로 나누어 두 차례에 걸쳐 치러지며, 각 시험은 2시간 40분씩 120문항, 즉 필수문제 25문항, 일반문제 65문항, 상황설정 30문항이다.

채점 기준은 필수문제와 일반문제는 각 1점, 상황설정문제 각 2점으로 300점 만점이다.

합격 기준은 필수문제는 절대평가로 50문제 중 80%인 40문제 이상 맞아야 한다. 11문제 이상 틀렸다면 일반문제와 상황설정 문제 점수와 상관없이 무조건 불합격이다. 일반문제와 상황설정 문제는 상대평가로 총 190문항인데 합격기준은 매년 다르다. 후생노동성에서 그 해 간호사를 몇 명 뽑을지 정하면 전체 응시생 수 중 몇 %의 수험자가 합격하는 형태이므로 합격기준은 매해 다르다. 과거 국가고시 합격률은 2020년(109회): 89.2%, 2019년(108회): 89.3%, 2018년(107회): 91%, 2017년(106회): 88.5%, 2016년(105회): 89.4%이다.

Ⅳ. 연구결과

1. 간호사 국가시험 관련 국내외 문헌고찰

가. 국내 간호사 국가시험 관련 문헌고찰

국내 간호사 국가시험 관련 문헌을 고찰한 결과, 간호사 국가시험 관련 출제범위와 관련된 주개념은 8개 직무영역, 134개의 직무항목, 통합형 표준학습목표, 481개 범주의 지식항목 등 이었다. 문제 유형과 관련된 주개념 암기형, 해석형, 문제해결형 이었고, 출제관리와 관련된 주개념은 시험과목, 시험위원회, 난이도, 변별도, 합격 기준 등이었다. 박인숙 외(2012)은 간호관리와 전문성 향상, 안전과 감염관리, 잠재적 위험요인, 기본간호, 생리적 통합성 유지, 약물 및 비경구요법, 심리 사회적 통합유지, 건강증진 및 유지 등의 8개 직무영역을 개발하였다. 다음 단계에서 박인숙 외(2014), 강소영 외(2015)는 8개 직무영역과 연계된 신규간호사의 직무를 확인하기 위한 연구를 진행하였다. 이어서 송라윤 외(2018)는 김영경 외(2018)의 연구에서 선행연구 결과에 기반하여 개발한 직무기준 Activity statement 분류의 적절성을 검토하면서 항목의 구체성 정도에 따라 통합 가능한 항목을 정리하여 8개 직무영역과 연계된 134개의 직무항목을 확정하였다. 그리고 간호사 국가시험 교과목 학습목표(한국간호과학회, 2017)로 부터 통합형의 표준학습목표를 설정하여, 표준학습목표를 8개 직무영역와 연계된 134개 직무항목과 연계하였다. 134개 직무항목과 연계된 표준학습목표로부터 1,303개의 지식항목을 도출하였다. 이어서 진행된 탁영란 외(2019) 연구에서는 통합형 간호사 국가시험 도입을 위한 초기 작업으로, 134개 직무행위와 연계하여 통합형의 표준학습목표로부터 481개 범주의 지식항목을 도출하였다. 또한 간호사의 역량 중심 평가를 위하여 국가시험 교과목별로 해당 지식항목에 국가시험 문항 분포의 가중치를 계산하여 8개 직무영역별로 추산된 가중치를 적용하여 간호사 국가시험 출제기준(안)을 제시하였다. 이와 같이 단계적으로 순차적으로 진행된 간호사 국가시험 출제모형의 개발과정은 <표 Ⅳ-1>, [그림 Ⅳ-1]과 같다.

나. 국외 간호사 국가시험 관련 문헌고찰

국외 간호사 국가시험 관련 문헌을 고찰한 결과, 간호사 국가시험 출제범위와 관련된 주개념은 ‘Category’, ‘Activity statements’, ‘Knowledge statements’, ‘안전하

고 효율적인 간호환경(Safe and Effective Care Environment)', '건강증진 및 유지(Health Promotion and Maintenance)', '심리사회적 통합(Psychosocial Integrity)', '생리적 통합(Physiological Integrity)', 그리고 '안전하고 효율적인 간호환경'의 하위영역으로 '간호 관리(Management of Care)'와 '안전과 감염관리(Safety and Infection Control)', '생리적 통합'의 하위영역으로 '기본간호와 안위(Basic Care and Comfort)', '약물과 비경구 치료(Pharmacological and Parenteral Therapies)', '잠재위험의 감소(Reduction of Risk Potential)', '생리적 적응(Physiological Adaptation)' 등이었다. 문제 유형과 관련된 주개념은 필수문제, 일반문제, 상황설정 문제이었고, 출제관리와 관련된 주개념은 채점 기준과 합격 기준이었다.

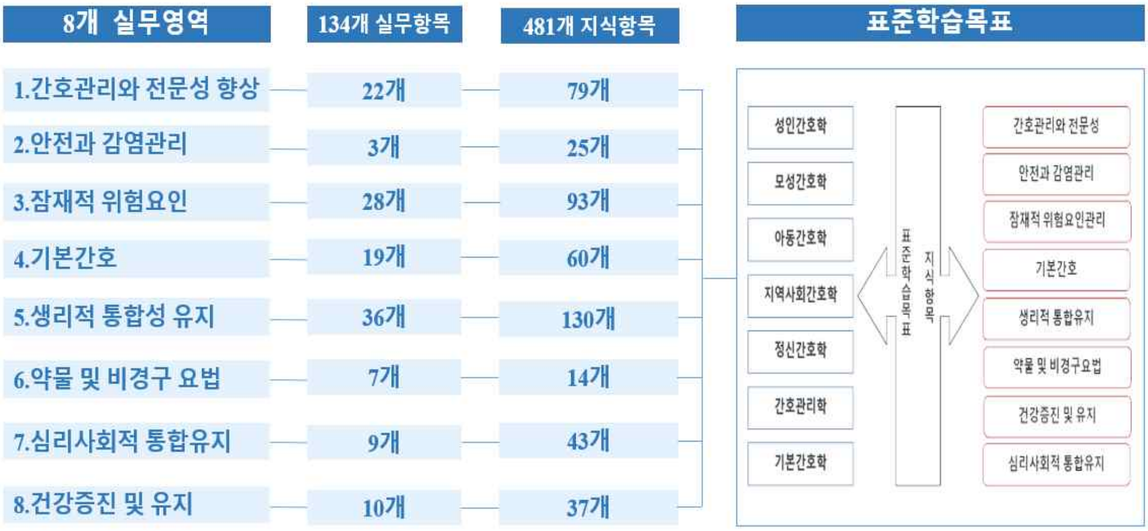

[그림 Ⅳ-1] 직무기반 통합형 간호사 국가시험 출제모형 개발과정

<표 Ⅳ-1> 간호사 국가시험 출제모형 개발을 위한 선행연구

	주제	연구목적	연구방법		연구결과
			연구대상	자료수집방법	
**박인숙 외 (2012)**	신규간호사 직무분석	• 간호사의 정의 및 간호사 직무에 대한 개념과 범위 정립<br>• 간호사 직무 내용을 분석하여 기술하고 체계화하여 직무기술서 작성<br>• 간호사 직무 수행에 필요한 관련 지식, 기술, 태도 기술<br>• 간호사 직무 수행 실태 및 직무 인식 조사	• 포커스그룹:<br>병원간호사(13), 보건소간호사(2), 간호관리자(7) 교수(6)<br>• 설문대상:<br>간호사 5000명	• 포커스그룹 연구<br>• 직무분석 연구<br>• 설문조사(간호사 직무수행 타당도, 난이도 및 중요도 조사)<br>• 전문가 자문회의<br>• 공청회	• 간호요구(needs)에 맞춘 간호대상자와 간호성과 중심의 가치 체계가 반영된 임무를 중심으로 함<br><br>- 8개 임무(duty)<br>- 49개 일(task)<br>- 303개 일의 요소(task element)
**박인숙 외 (2014)**	간호사직무분석, 학습목표, 국가시험 연계성 분석	• 간호사 직무분석과 학습목표 및 국가시험 출제범위를 비교하여 연계성 분석<br>• 간호현장에서 요구하는 기본 직무 반영 및 문제해결능력 등을 고려한 간호사국가시험 개선(안) 도출	• 전문가자문:<br>교육경력 10년이상 간호교육전문가<br>• 설문대상:<br>현장실무전문가와 간호교육전문가 946명	• 1차분석(연구진)<br>• 전문가자문<br>• 설문조사: 내용타당도검증<br>• 공청회	• 간호사 임무를 국가시험 출제영역으로, 일을 세부영역으로, 간호지식을 출제내용으로 하는 출제기준안 제시<br>- 8개 출제영역<br>- 49개 세부영역<br>- 308개 출제 내용
**강소영 외 (2015)**	최소직무능력 규정 & 국가시험 적용방안	• 간호대학 졸업자의 직무내용 분석을 통해 현장중심 최소직무능력규정 개발<br>• 최소직무능력 내용을 바탕으로 국가시험 적용·개선(안) 도출<br>• 최소직무능력 및 관련내용의 타당도와 신뢰도 분석	• 간호사 직무관련 자료의 질적 비교·분석<br>• 간호사 직무분석 자료의 양적 비교·분석<br>• 직무능력표준개발자료 고찰		• '간호학총론' 단일시험과목으로<br>• 기존 36개 출제분야 → 8가지 직무영역으로<br>• 기존 113개 출제범위 → 49개 직무로<br>• 간무실무역량기반 국가시험 개편 제안<br>- 8개 직무영역<br>- 49개 직무<br>- 372개 직무능력내용

김영경 외 (2018)	직무기반 간호사 국가시험을 위한 문항개발 기준 및 절차 개선	• 간호사 국가시험의 신뢰도와 타당도 향상을 위해 직무기반의 문항개발기준 및 절차 개선방안 도출	• 문항개발기준 분석: 초점집단인터뷰(교수&간호사 20명), 설문조사(1년이상 간호사 482명)<br>• 문항개발기준 절차: 전문가패널, 설문조사(222명 교수)	• 문항개발기준 분석<br>• 문항개발기준 절차	• 직무기반 문항개발의 대분류와 중분류로 활용 가능한 8개 임무 영역별(대분류)로 구체적인 직무에 대한 진술(중분류) 제시<br>- 8개 임무(duty)<br>- 205개 AS (Activity Statement)
송라윤 외 (2018)	간호사 국가시험 통합출제를 위한 모형구축	• 신규간호사 실무항목과 전공과목 관련성 파악<br>• 신규간호사 실무항목과 전공과목 표준학습목표 연계<br>• 신규간호사 실무항목별 문항개발 위한 지식항목 추출<br>• 지식항목에 대한 타당도 평가<br>• 신규간호사의 직무와 표준학습목표가 연계된 간호사 국가시험 통합모형 개발	• 전문가 회의<br>• 타당도 검증: 임상간호사 24명	• 지식 항목 도출(문헌<br>• 지식 항목에 대한 타당도 조사	• 신규간호사의 실무항목과 학습목표를 연계시킨 통합모형에 기반한 국가시험 문항개발 방향 제시<br>• 일부 학습목표는 실무항목과 지식항목 연계에서 제외함<br>- 8개 직무영역<br>- 134개 AS 개정안<br>- 1303개 지식항목(Knowledge statement)
탁영란 외 (2019)	국가시험 및 면허체계 개선방안	• 신규간호사 실무역량 현황조사 교육요구도 확인<br>• 교과목 통합모형과 역량중심평가를 통한 간호사 국가시험 개선 방향 도출<br>• 국가시험 핵심역량별 가중치 설정과 컴퓨터화를 위한 준비과제 도출<br>• 간호사 면허관리체계 필요성 고찰 및 개선안 도출	• 설문조사(신규간호사 대상)<br>• 8개 기관 방문	• 설문조사<br>• 선진사례 기관방문<br>• 자문회의	• 교과목 통합모형과 역량 중심의 간호사 국가시험 개선 방향 도출<br>• 교과목 교육목표와 국시 문항분포에 따라 지식항목에 가중치를 부여함<br>- 8개 대분류<br>- 134개 AS<br>- 1301개 KS
NCSBN (2019)	Test Plan				• 4개 대상자 요구, 2개의 범주는<br>• 세부 영역 포함
**[8가지 영역]**<br>A. 간호관리와 전문성 향상, B. 안전과 감염관리, C. 위험요인 사정, D. 기본간호, E. 생리적 통합유지, F. 약물 및 비경구요법, G. 심리사회적 통합유지, H. 건강증진 및 유지					

2. 간호사 국가시험 출제모형 제시

간호사 국가시험 출제기준(standard of test construction)은 시험문제를 출제하는 기준으로, 시험과목 및 대항목, 분야(또는 영역), 시험문제수, 배점 등으로 구성된다(한국보건의료인국가시험원 홈페이지, 2020). 출제기준의 근거가 되는 표준으로 간호사 국가시험 출제모형(model of test construction)을 제시하기 위하여 국내외 국가시험 관련 문헌을 고찰하였다. 문헌고찰 결과, <표 Ⅳ-1>와 같이 신규간호사 직무에 기반한 출제기준 개발을 위해 단계별로 순차적으로 2012년에 개발된 8개 신규간호사 직무영역, 2018년 8개 직무영역과 연계하여 개발된 134개 직무항목, 7개 국가시험 교과목 학습목표를 통합한 표준학습목표, 2019년 직무영역, 직무항목 및 통합형 표준학습목표와 연계하여 개발된 481개 범주의 지식항목 등의 주개념으로 구성된 출제모형을 확인하였다. 문헌고찰을 통해서 확인된 최신의 신규간호사 직무에 기반한 통합형 간호사 국가시험 출제모형을 제시하기 위해서는 출제모형을 구성하는 주개념으로 2018년에 개발된 134개의 신규간호사 직무항목에 대한 타당성 및 적절성 검증이 우선되어야 할 필요성이 제기되어 신규간호사 직무항목에 대한 타당성과 적절성 평가를 실시하였다.

가. 출제모형의 간호사 직무항목에 대한 타당성과 적절성 평가

최신의 직무에 기반한 통합형 간호사 국가시험 출제모형을 제시하기 위해, 우선적으로 2018년에 개발된 134개 신규간호사 직무항목(Activity Statement)에 대한 타당성과 적절성 평가를 하였다. 임상 및 지역사회 간호 실무현장의 간호사 264명을 대상으로 설문조사를 실시하였고, 이 중 260부를 자료분석에 활용하였다.

1) 인구사회학적 특성

대상자의 인구사회학적 특성은 다음과 같다 <표 Ⅳ-2>. 직위별로는 일반간호사가 75.38%, 주임 및 책임간호사 8.08%, 수간호사 5.00%, 보건교사 4.23%, 간호직 공무원 3.46%, 기타 3.08% 순으로 나타났다. 기관 소재지별로는 수도권 34.61%, 충청권 30.77%, 강원권 11.54%, 전라권 11.54%, 경상권 11.54% 순이었다. 기관별로는 상급종합병원이 49.62%로 가장 많았고, 종합병원 36.92%, 보건소 6.92%, 학교(보건교사) 3.46% 순으로 나타났다. 간호사로 근무한 기간의 경우 7년 이상이 38.85%로 가장 높게 나타났고, 3~5년 미만이 21.92%, 1~3년 미만이 19.23%, 5~7년 미만이 13.46% 순으로 나타났으며, 현 직장 근무 기간의 경우 7년 이상이 33.46%, 1~3년 미만이 24.62%, 3~5년 미만이 19.62%, 5~7년 미만이 11.54% 순이었으며, 1년 미만이 10.76%로 가장 낮게 나타났다. 근무부서별로는 중환자실이 13.85%로 가장 높았고, 소아청소년과 병동 11.15%, 외과계 병동 11.15%, 수술실/마취회복실 10.77%, 응급

실 10.00%, 정신과 병동 9.23%, 내과계 병동 9.23%, 분만실, 산부인과 병동 8.08%, 보건소 6.92% 순으로 나타났다.

<표 Ⅳ-2> 대상자의 인구사회학적 특성

(N=260)

구분	변수	빈도 (n)	백분율 (%)
직위	일반간호사	196	75.38
	주임, 책임간호사	21	8.08
	수간호사	13	5.00
	간호관리자	2	0.77
	보건교사	11	4.23
	간호직 공무원	9	3.46
	기타	8	3.08
기관 소재지	수도권	90	34.61
	강원권	30	11.54
	충청권	80	30.77
	전라권	30	11.54
	경상권	30	11.54
기관	상급종합병원	129	49.62
	종합병원	96	36.92
	병·의원	7	2.69
	학교(보건교사)	9	3.46
	보건소	18	6.92
	기타	1	0.39
간호사 근무기간	1년 미만	17	6.54
	1 ~ 3년 미만	50	19.23
	3 ~ 5년 미만	57	21.92
	5 ~ 7년 미만	35	13.46
	7년 이상	101	38.85
현 직장 근무기간	1년 미만	28	10.76
	1 ~ 3년 미만	64	24.62
	3 ~ 5년 미만	51	19.62
	5 ~ 7년 미만	30	11.54
	7년 이상	87	33.46
근무부서	내과계 병동	24	9.23
	외과계 병동	29	11.15
	중환자실	36	13.85
	소아청소년과 병동	29	11.15
	분만실, 산부인과병동	21	8.08
	신생아실	12	4.62
	응급실	26	10.00
	정신과병동	24	9.23
	수술실/마취회복실	28	10.77
	학교	10	3.85
	보건소	18	6.92
	기타	3	1.15

2) 8개 직무영역별 신규간호사 직무항목의 중요도와 수행도

가) 간호관리와 전문성 향상

간호관리와 전문성 향상 영역의 직무에 대한 신규간호사 직무 중요도와 수행도는 다음과 같다<표 Ⅳ-3>.

신규간호사 직무 중요도 순으로 살펴본 결과, 「6. 장비를 적절하고 안전하게 사용」이 4.67±0.56, 「법적 직무 범위 내에서 간호 수행」이 4.66±0.57, 「11. 대상자의 개인정보 및 사생활 보호」가 4.66±0.57, 「12. 대상자에게 치료 및 절차에 따라 적절한 설명을 하고 동의를 획득하였는지 확인」이 4.58±0.60, 「4. 지침에 따라 간호기록」이 4.51±0.66, 「1. 인수인계 시행」이 4.39±0.80, 「13. 환자의 권리와 책임에 관하여 대상자에게 교육 제공」이 4.38±0.74, 「14. 억제대 사용 시 법적 및 윤리적 간호」가 4.35±0.75, 「5. 입원, 전동, 퇴원」이 4.34±0.81, 「10. 간호전문직 윤리 준수와 역할」이 4.34±0.78. 「3. 기록 시 표준화된 약어사용」이 4.28±0.77 순이었다.

신규 간호사 직무 수행도 순으로 살펴본 결과, 「11. 대상자의 개인정보 및 사생활 보호」가 4.19±0.84, 「13. 환자의 권리와 책임에 관하여 대상자에게 교육 제공」이 3.88±0.93, 「12. 대상자에게 치료 및 절차에 따라 적절한 설명을 하고 동의를 획득하였는지 확인」이 3.88±0.91, 「14. 억제대 사용 시 법적 및 윤리적 간호」가 3.82±0.88, 「10. 간호전문직 윤리 준수와 역할」 3.81±0.92, 「6. 장비를 적절하고 안전하게 사용」이 3.80±3.34, 「4. 지침에 따라 간호기록」이 3.76±0.92, 「2. 기록 시 표준화된 약어사용」이 3.76±0.92, 「2. 법적 직무 범위 내에서 간호수행」이 3.68±0.90, 「5. 입원, 전동, 퇴원」이 3.55±0.95, 「7. 간호단위 물품교환체계에 따른 물품관리」가 3.39±1.01, 「15. 일차의료기반의 보건간호」가 3.38±1.01, 「1. 인수인계시행」이 3.25±0.99, 「22. 전문직간 협업」이 3.07±0.99, 「16. 지역사회간호사업의 법적 기준 및 지침에 따른 활동 참여」가 3.05±1.09 순이었다.

<표 Ⅳ-3> 간호관리와 전문성 향상

번호	항목	중요도 (Mean±SD)	수행도 (Mean±SD)
1	인수인계시행	4.39±0.80	3.25±0.99
2	법적 직무범위 내에서 간호 수행	4.66±0.57	3.68±0.90
3	기록시 표준화된 약어사용	4.28±0.77	3.70±0.89
4	지침에 따라 간호기록	4.51±0.66	3.76±0.92
5	입원, 전동, 퇴원	4.34±0.81	3.55±0.95
6	장비를 적절하고 안전하게 사용	4.67±0.56	3.80±3.34

번호	항목	중요도 (Mean±SD)	수행도 (Mean±SD)
7	간호단위 물품교환체계에 따른 물품관리	3.91±0.95	3.39±1.01
8	질 향상(QI) 활동에 참여	3.16±1.08	2.64±0.96
9	간호사업 평가 관련 업무수행(도구개발, 자료조사, 분석, 비교 및 사업개선)	2.81±1.15	2.42±0.98
10	간호전문직 윤리 준수와 역할	4.34±0.78	3.81±0.92
11	대상자의 개인정보 및 사생활 보호	4.66±0.56	4.19±0.84
12	대상자에게 치료 및 절차에 따라 적절한 설명을 하고 동의를 획득하였는지 확인	4.58±0.60	3.88±0.91
13	환자의 권리와 책임에 관하여 대상자에게 교육 제공	4.38±0.74	3.88±0.93
14	억제대 사용 시 법적 및 윤리적 간호	4.35±0.75	3.82±0.88
15	일차의료기반의 보건간호	3.78±1.08	3.38±1.01
16	지역사회 간호사업의 법적 기준 및 지침에 따른 활동 참여	3.35±1.18	3.05±1.09
17	사례관리활동 참여	3.12±1.05	2.78±0.95
18	업무를 조직화하여 일을 효율적으로 관리	3.70±1.07	2.88±0.96
19	환자분류체계 관련 정보수집 및 활용	3.54±1.02	3.00±0.94
20	간호대상에 적합한 다양한 자원 및 매체선택	3.40±1.00	2.86±0.91
21	취약가족에게 필요한 지역사회 자원활용	2.97±1.08	2.58±0.98
22	전문직간 협업	3.90±0.90	3.07±0.99

※문항별 결측치 제외

간호관리와 전문성 향상의 직무 항목에 대해 신규 간호사의 직무 중요도 및 수행도를 y축은 중요도, x축을 수행도로 설정하고 3점을 기준선으로 IPA 분석한 결과는 [그림 Ⅳ-2]과 같다. 우선, 1사분위에 위치하고 있는「8. 질 향상(QI) 활동에 참여」,「17. 사례관리활동 참여」,「18. 업무를 조직화하여 일을 효율적으로 관리」,「20. 간호대상에 적합한 다양한 자원 및 매체선택」항목은 중요도는 3점 이상이나 수행도가 3점 이하인 직무로 나타났다. 3사분위에 위치하고 있는「9. 간호사업 평가 관련 업무수행(도구개발, 자료조사, 분석, 비교 및 사업개선)」,「21. 취약가족에게 필요한 지역사회 자원활용」, 은 중요도와 수행도 모두 3점 이하로 나타났으며 그 이외의 직무들은 모두 2사분위에 위치하고 있어 신규 간호사의 직무 중요도와 수행도 모두 3점 이상인 것으로 나타났다.

나) 안전과 감염관리

안전과 감염관리 영역의 직무에 대한 신규간호사 직무 중요도와 수행도는 다음과 같다<표 Ⅳ-4>.

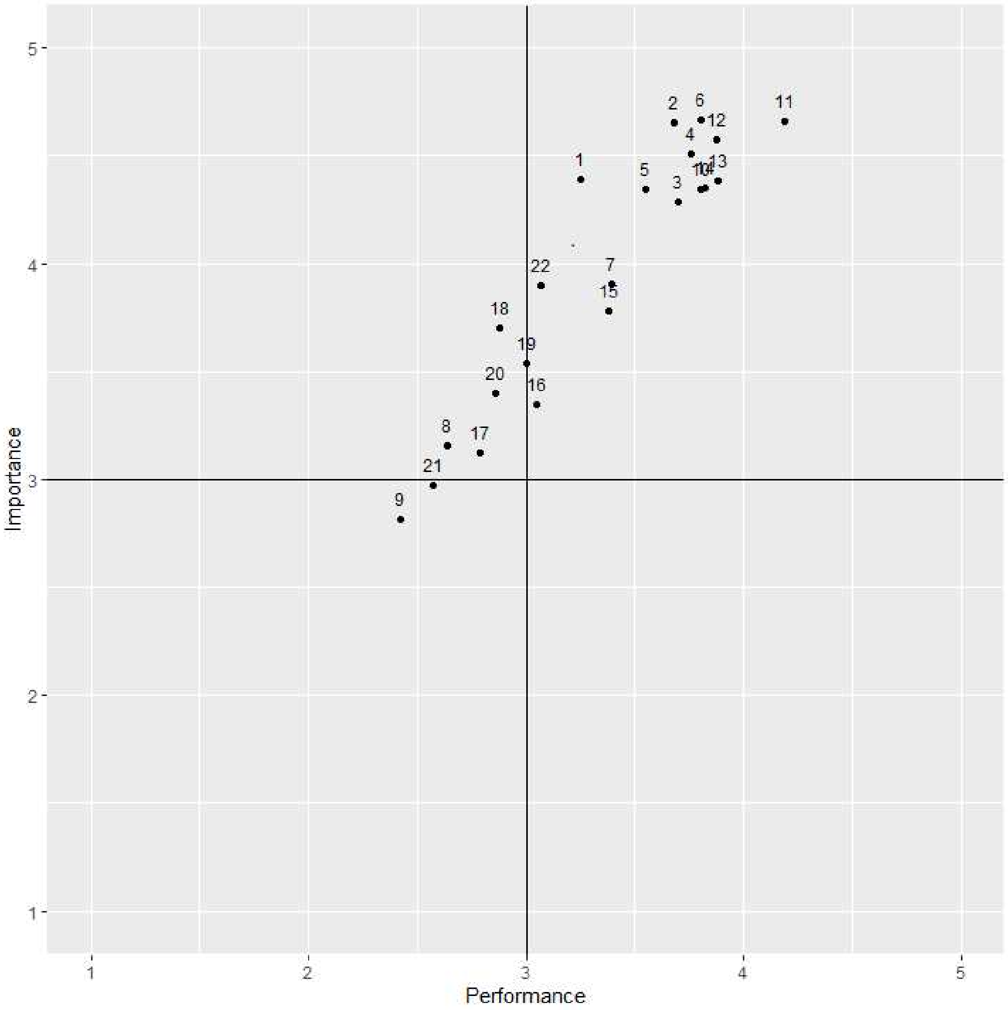

[그림 Ⅳ-2] 간호관리와 전문성 향상 직무의 IPA

신규 간호사 직무 중요도 순으로 살펴본 결과, 「24. 감염관리」가 4.76±0.51「23. 안전한 환경 제공」이 4.67±0.58, 「25. 위험물질과 유해물질 관리」가 4.59±0.69 순이었다.

신규 간호사 직무 수행도 순으로 살펴본 결과, 「23. 안전한 환경 제공」이 4.02±0.81, 「24. 감염관리」가 4.00±0.88, 「25. 위험물질과 유해물질 관리」가 3.71±0.96 순으로 나타났다.

<표 Ⅳ-4> 안전과 감염관리

번호	항목	중요도 (Mean±SD)	수행도 (Mean±SD)
23	안전한 환경 제공	4.67±0.58	4.02±0.81
24	감염관리	4.76±0.51	4.00±0.88
25	위험물질과 유해물질 관리	4.59±0.69	3.71±0.96

※문항별 결측치 제외

안전과 감염관리 직무 항목에 대해 신규 간호사의 직무 중요도 및 수행도를 y축은 중요도, x축을 수행도로 설정하고 3점을 기준선으로 IPA 분석한 결과는 [그림 Ⅳ-3]과 같다. 「23. 안전한 환경 제공」, 「24. 감염관리」, 「25. 위험물질과 유해물질 관리」 모두 2사분위에 위치하고 있어 신규 간호사의 직무 중요도와 수행도 모두 3점 이상인 것으로 나타났다.

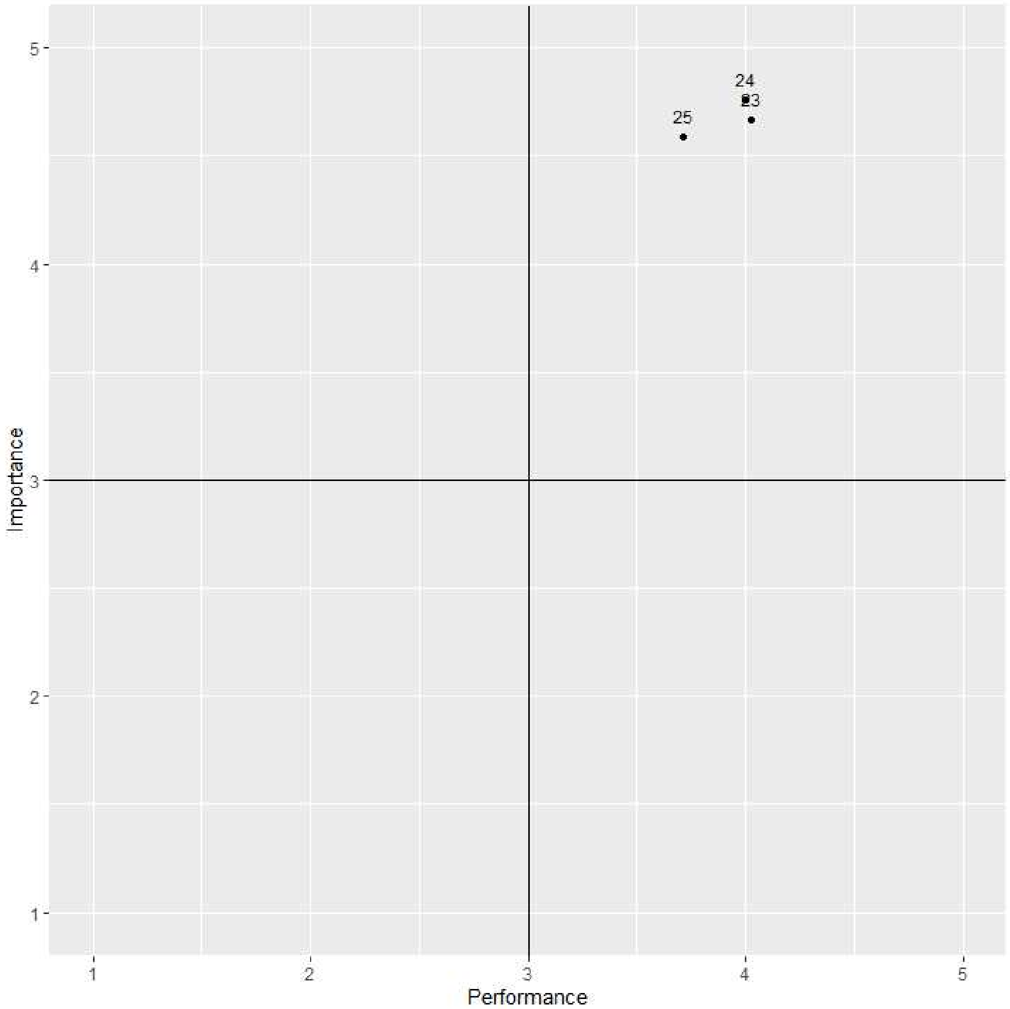

[그림 Ⅳ-3] 안전과 감염관리 직무의 IPA

다) 위험요인 사정

위험요인 사정 영역의 직무에 대한 신규간호사 직무 중요도와 수행도는 다음과 같다<표 Ⅳ-5>.

신규 간호사 직무 중요도 순으로 살펴본 결과, 「28. 활력징후 사정」이 4.83±0.43,

「44. 심폐소생술 간호」가 4.81±0.47,「45. 응급간호」가 4.76±0.50,「38. 건강력 사정」이 4.53±0.68,「47. 수술간호」가 4.51±0.72,「39. 신체검진 수행 및 결과 해석」이 4.47±0.70,「27. 대상자의 건강문제에 대해 우선순위 결정」이 4.46±0.74,「46. 진단검사 간호」가 4.42±0.75,「26. 간호계획, 진료지침 수행」이 4.35±0.76,「36. 노인간호」가 4.32±0.81 순이었다.

신규 간호사 직무 수행도 순으로 살펴본 결과,「28. 활력징후 사정」이 4.47±0.75,「38. 건강력 사정」이 3.81±0.86,「26. 간호계획, 진료지침 수행」이 3.69±0.90,「39. 신체검진 수행 및 결과 해석」이 3.64±0.89,「36. 노인간호」가 3.63±0.91,「34. 청소년 간호」가 3.54±0.93,「35. 폐경기 여성 간호」가 3.52±0.91,「33. 학령기 간호」가 3.51±0.95,「47. 수술간호」가 3.50±0.94 순이었다.

<표 Ⅳ-5> 위험요인 사정

번호	항목	중요도 (Mean±SD)	수행도 (Mean±SD)
26	간호계획, 진료지침 수행	4.35±0.76	3.69±0.90
27	대상자의 건강문제에 대해 우선순위 결정	4.46±0.74	3.47±0.95
28	활력징후 사정	4.83±0.43	4.47±0.75
29	신생아 간호	4.24±0.92	3.40±1.01
30	영아 간호	4.19±0.94	3.39±1.02
31	유아 간호	4.20±0.94	3.42±1.01
32	학령전기 간호	4.17±0.95	3.49±0.95
33	학령기 간호	4.17±0.95	3.51±0.95
34	청소년 간호	4.17±0.94	3.54±0.93
35	폐경기 여성 간호	4.17±0.92	3.52±0.91
36	노인간호	4.32±0.81	3.63±0.91
37	성 건강 간호	4.10±0.93	3.48±0.94
38	건강력 사정	4.53±0.68	3.81±0.86
39	신체검진 수행 및 결과 해석	4.47±0.70	3.64±0.89
40	생식기 건강사정	4.13±0.88	3.44±0.96
41	태아건강사정 및 간호	4.10±1.02	3.25±1.03
42	신생아 건강사정 및 간호	4.12±1.00	3.29±1.03
43	고위험신생아 건강사정 및 간호	4.18±1.01	3.1±1.07
44	심폐소생술 간호	4.81±0.47	3.34±1.09
45	응급간호	4.76±0.50	3.24±1.02
46	진단검사 간호	4.42±0.75	3.33±0.95
47	수술간호	4.51±0.72	3.50±0.94
48	산전간호와 교육	4.09±0.99	3.34±0.99
49	분만 중 간호와 교육	4.11±0.98	3.27±1.01

번호	항목	중요도<br>(Mean±SD)	수행도<br>(Mean±SD)
50	산후관리와 교육	4.11±0.98	3.29±0.99
51	고위험 임부간호	4.15±1.00	3.16±1.02
52	고위험 산부간호	4.14±1.00	3.15±1.01
53	고위험 산모간호	4.14±1.00	3.15±1.01

※문항별 결측치 제외

위험요인 사정 직무 항목에 대해 신규 간호사의 직무 중요도 및 수행도를 y축은 중요도, x축을 수행도로 설정하고 3점을 기준선으로 IPA 분석한 결과는 [그림 Ⅳ-4]과 같다. 위험요인 사정 영역의 28개 직무 모두 2사분위에 위치하고 있어 신규 간호사의 직무 중요도와 수행도 모두 3점 이상인 것으로 나타났다.

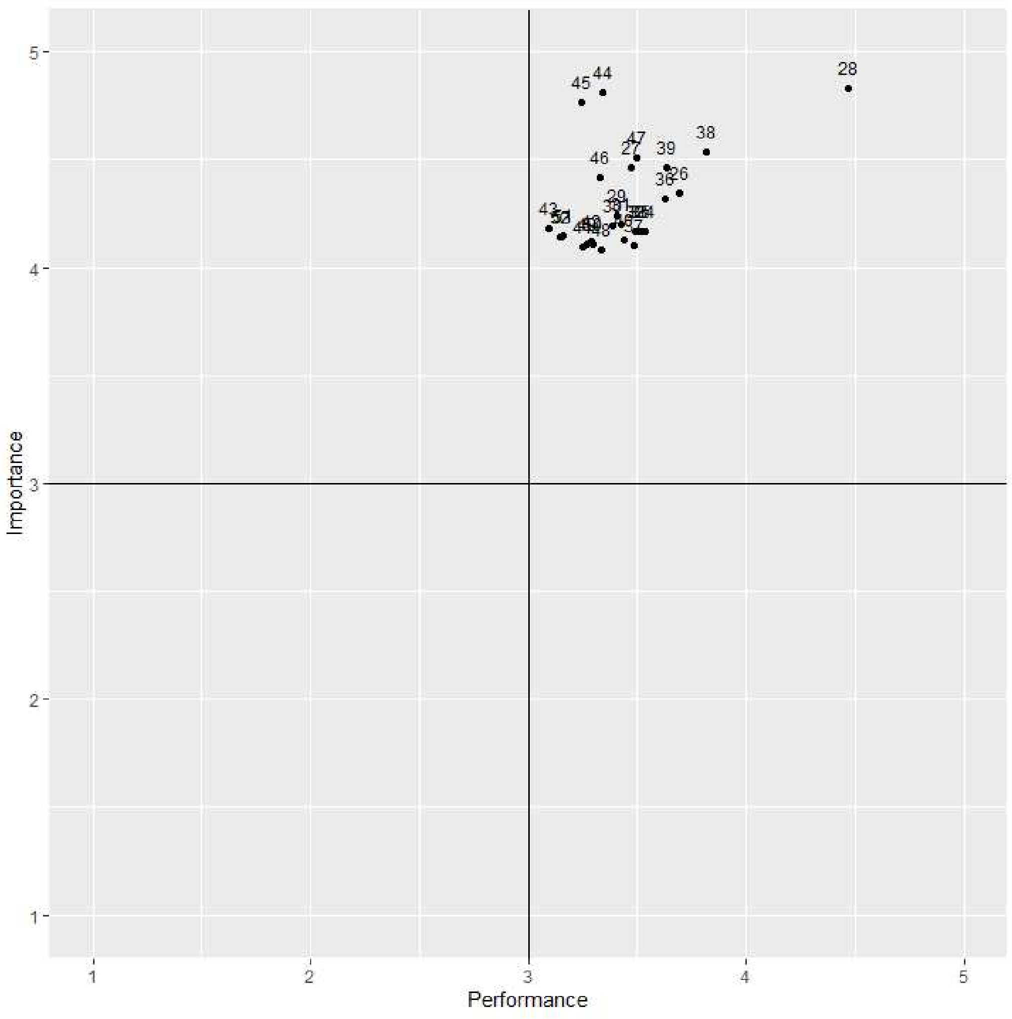

[그림 Ⅳ-4] 위험요인 사정 직무의 IPA

라) 기본간호

기본간호 영역의 직무에 대한 신규간호사 직무 중요도와 수행도는 다음과 같다<표 Ⅳ-6>.

신규 간호사 직무 중요도 순으로 살펴본 결과, 「57. 섭취량 및 배설량 사정과 간호」가 4.60±0.60, 「54. 체온유지간호」가 4.49±0.66, 「64. 기관절개부 관리」가 4.38±0.81, 「61. 배뇨장애 대상자 사정 및 간호」가 4.38±0.74, 「67. 피부통합성 사정 및 간호」가 4.35±0.77, 「62. 투석대상자 관리」가 4.34±0.81, 「56. 개인위생간호」가4.34±0.76, 「60. 섭취장애 대상자 사정 및 간호」가 4.32±0.77, 「65. 배변관리」가 4.32±0.76, 「59. 질환별 영양문제의 사정 및 관리」가 4.31±0.80 순이었다.

신규 간호사 직무 수행도 순으로 살펴본 결과, 「54. 체온유지간호」가 4.19±0.81, 「56. 개인위생간호」 가 4.17±0.78, 「57. 섭취량 및 배설량 사정과 간호」 가 4.16±0.76, 「66. 수면과 휴식 간호」 가 4.02±0.81, 「58. 영양사정 및 관리」 가 3.95±0.85, 「68. 이동간호」가 3.91±0.91, 「65. 배변관리」가 3.90±0.87, 「67. 피부통합성 사정 및 간호」가 3.85±0.91, 「55. 세척(irrigation)(방광, 귀, 눈) 수행」이 3.81±0.95, 「61. 배뇨장애 대상자 사정 및 간호」가 3.80±0.86 순이었다.

<표 Ⅳ-6> 기본간호

번호	항목	중요도 (Mean±SD)	수행도 (Mean±SD)
54	체온유지간호	4.49±0.66	4.19±0.81
55	세척(irrigation)(방광, 귀, 눈) 수행	4.19±0.86	3.81±0.95
56	개인위생간호	4.34±0.76	4.17±0.78
57	섭취량 및 배설량 사정과 간호	4.60±0.60	4.16±0.76
58	영양사정 및 관리	4.29±0.80	3.95±0.85
59	질환별 영양문제의 사정 및 관리	4.31±0.80	3.66±0.88
60	섭취장애 대상자 사정 및 간호	4.32±0.77	3.76±0.83
61	배뇨장애 대상자 사정 및 간호	4.38±0.74	3.80±0.86
62	투석대상자 관리	4.34±0.81	3.34±0.97
63	요루 및 장루관리	4.28±0.84	3.27±1.00
64	기관절개부 관리	4.38±0.81	3.34±1.05
65	배변관리	4.32±0.76	3.90±0.87
66	수면과 휴식 간호	4.19±0.89	4.02±0.81
67	피부통합성 사정 및 간호	4.35±0.77	3.85±0.91

번호	항목	중요도 (Mean±SD)	수행도 (Mean±SD)
68	이동간호	4.08±0.96	3.91±0.91
69	활동과 자기돌봄 장애 사정 및 간호	4.02±0.96	3.76±0.91
70	외과적 장치 관리	4.25±0.85	3.45±0.97
71	척추손상 및 신경질환 대상자의 간호	4.25±0.85	3.39±0.99
72	관절대치술 환자 간호	4.16±0.93	3.44±0.97

※문항별 결측치 제외

기본간호 직무 항목에 대해 신규 간호사의 직무 중요도 및 수행도를 y축은 중요도, x축을 수행도로 설정하고 3점을 기준선으로 IPA 분석한 결과는 [그림 Ⅳ-5]와 같다. 기본간호 영역의 19개 직무 모두 2사분위에 위치하고 있어 신규 간호사의 직무 중요도와 수행도 모두 3점 이상인 것으로 나타났다.

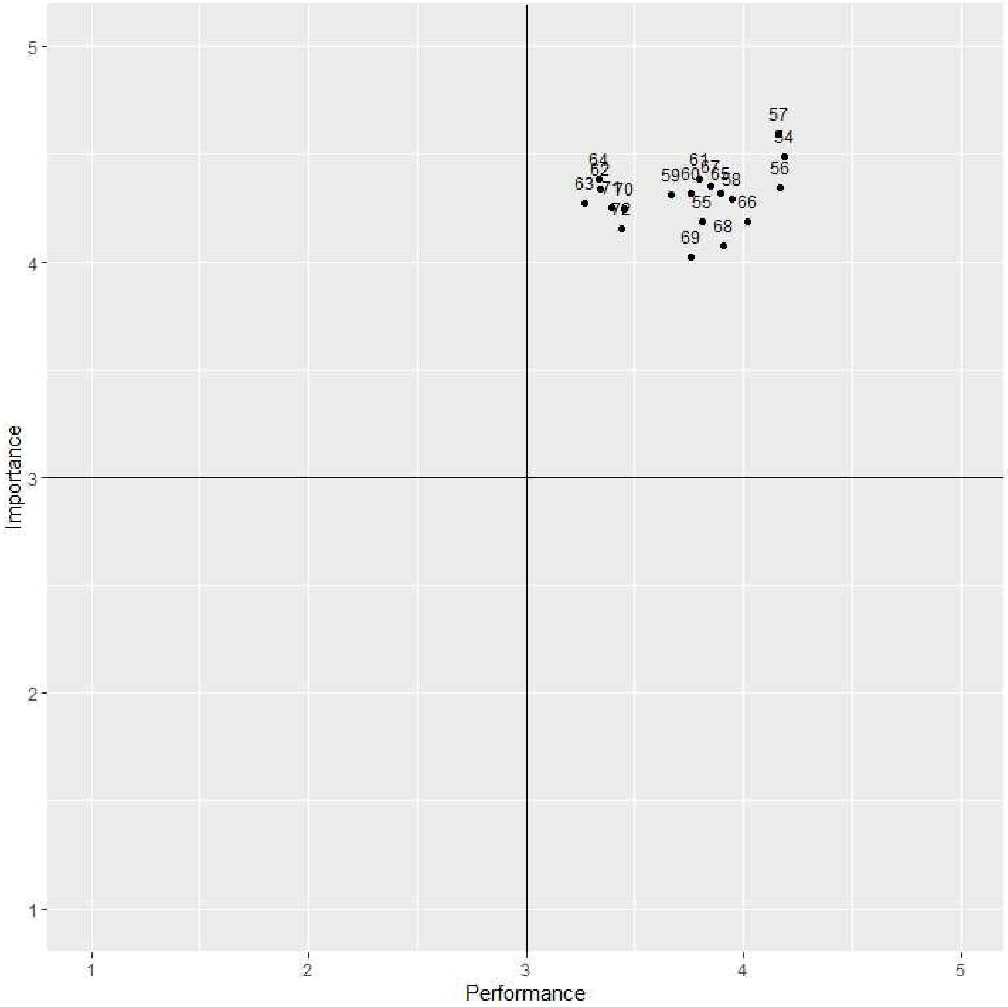

[그림 Ⅳ-5] 기본간호 직무의 IPA

마) 생리적 통합유지

생리적 통합유지 영역의 직무에 대한 신규간호사 직무 중요도와 수행도는 다음과 같다<표 Ⅳ-7>.

신규 간호사 직무 중요도 순으로 살펴본 결과, 「73. 호흡기능장애 대상자 간호」가 4.60±0.62, 「76. 중심정맥관 관리」 가 4.57±0.64, 「74. 호흡증진 중재」 가 4.57±0.61, 「75. 호흡보조장치 관리」가 4.53±0.67, 「106. 통증간호」가 4.50±0.68, 「104. 배액장치 관리」가 4.48±0.67, 「84. 체액 전해질 불균형 사정 및 간호」가 4.47±0.72, 「108. (법정) 감염질환자의 전파예방 및 간호」가 4.43±0.78, 「98. 두개내압 상승 환자의 간호」가 4.40±0.83 순이었다.

신규 간호사 직무 수행도 순으로 살펴본 결과, 「106. 통증간호」가 3.87±0.85, 「101. 상처간호 수행 및 드레싱교환」이 3.65±0.96, 「93. 당뇨병 합병증 예방을 위한 간호」가 3.64±0.90, 「104. 배액장치 관리」가 3.63±0.95, 「74. 호흡증진 중재」가 3.57±0.90, 「91. 배뇨장애를 가진 대상자 간호」가 3.56±0.91, 「103. 피부질환 대상자의 간호중재」가 3.55±0.89, 「94. 내분비계 장애 대상자 간호」가 3.53±0.90, 「73. 호흡기능장애 대상자 간호」가 3.52±0.86, 「92. 당질 대사장애 대상자 간호」가 3.50±0.95 순이었다.

<표 Ⅳ-7> 생리적 통합유지

번호	항목	중요도 (Mean±SD)	수행도 (Mean±SD)
73	호흡기능장애 대상자 간호	4.60±0.62	3.52±0.86
74	호흡증진 중재	4.57±0.61	3.57±0.90
75	호흡보조장치 관리	4.53±0.67	3.32±0.98
76	중심정맥관 관리	4.57±0.64	3.45±0.99
77	태아질식 증상과 징후 사정 및 간호	4.25±0.94	3.11±1.02
78	고위험 신생아 보육기 적용간호	4.05±1.04	3.02±1.08
79	호흡재활관리	4.12±0.89	3.24±0.98
80	심전도 관리	4.38±0.78	3.29±1.02
81	순환 보조장치 관리	4.25±0.86	3.05±1.06
82	동맥관 관리	4.30±0.85	3.07±1.06
83	조직 관류 장애 대상자 사정 및 간호	4.32±0.82	3.26±0.99
84	체액 전해질 불균형 사정 및 간호	4.47±0.72	3.42±0.94

번호	항목	중요도 (Mean±SD)	수행도 (Mean±SD)
85	활동 지속성 장애 대상자 사정 및 간호	4.18±0.89	3.42±0.94
86	정맥 순환증진 장치 간호	4.30±0.78	3.38±1.00
87	혈액 기능장애 대상자 간호	4.36±0.77	3.32±0.95
88	순환 기능장애 대상자 간호	4.38±0.75	3.33±0.96
89	심장 수술 후 간호	4.34±0.84	3.12±1.03
90	소화 기능장애 대상자 간호	4.35±0.76	3.46±0.92
91	배뇨장애를 가진 대상자 간호	4.35±0.76	3.56±0.91
92	당질 대사장애 대상자 간호	4.34±0.76	3.50±0.95
93	당뇨병 합병증 예방을 위한 간호	4.35±0.77	3.64±0.90
94	내분비계 장애 대상자 간호	4.37±0.74	3.53±0.90
95	생식기 질환/생식기 건강문제를 가진 대상자 간호	4.17±0.86	3.49±0.89
96	면역손상 대상자 사정 및 간호	4.32±0.78	3.46±0.91
97	감각기능 장애를 가진 대상자 사정 및 간호	4.22±0.83	3.45±0.93
98	두개내압 상승 환자의 간호	4.4±0.83	3.25±1.00
99	신경계 질환별 간호중재	4.37±0.79	3.32±0.97
100	운동기능 장애 간호중재	4.17±0.89	3.47±0.92
101	상처간호 수행 및 드레싱교환	4.39±0.76	3.65±0.96
102	화상환자 간호중재	4.17±0.93	3.30±0.96
103	피부질환 대상자의 간호중재	4.20±0.83	3.55±0.89
104	배액장치 관리	4.48±0.67	3.63±0.95
105	계통별 신생물 질환 대상자 간호	4.07±0.96	3.31±0.94
106	통증간호	4.50±0.68	3.87±0.85
107	재활간호서비스	3.90±1.04	3.34±0.97
108	(법정) 감염질환자의 전파예방 및 간호	4.43±0.78	3.47±0.97

※문항별 결측치 제외

생리적 통합유지 직무 항목에 대해 신규 간호사의 직무 중요도 및 수행도를 y축은 중요도, x축을 수행도로 설정하고 3점을 기준선으로 IPA 분석한 결과는 [그림 Ⅳ-6]와 같다. 기본간호 영역의 36개 직무 모두 2사분위에 위치하고 있어 신규 간호사의 직무 중요도와 수행도 모두 3점 이상인 것으로 나타났다.

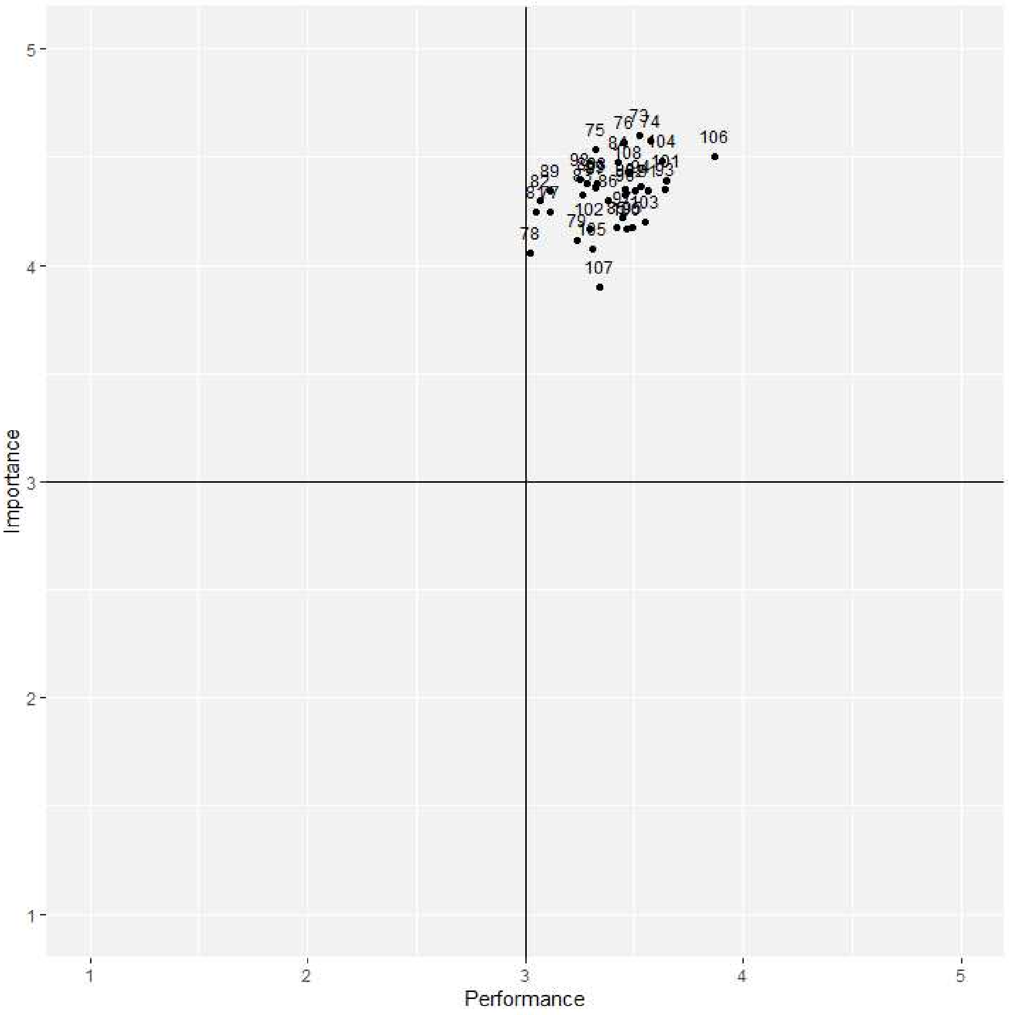

[그림 Ⅳ-6] 생리적 통합유지 직무의 IPA

바) 약물 및 비경구요법

약물 및 비경구요법 영역의 직무에 대한 신규간호사 직무 중요도와 수행도는 다음과 같다<표 Ⅳ-8>.

신규 간호사 직무 중요도 순으로 살펴본 결과, 「109. 투약의 적절성과 정확성」이 4.85±0.44, 「111. 정맥주입 장치 관리」가 4.85±0.44, 「110. 약물투여에 필요한 계산 시행」가 4.83±0.47, 「114. 대상자에게 약물에 대해 교육」이 4.78±0.47, 「113. 말초정맥관 삽입, 유지, 제거」가 4.77±0.55, 「115. 고위험 약품관리(마약관리)」가 4.75±0.54, 「112. 약품관리」가 4.73±0.57 순으로 모든 항목에서 4점을 상회하였다.

신규 간호사 직무 수행도 순으로 살펴본 결과, 「109. 투약의 적절성과 정확성」이 4.11±0.89, 「111. 정맥주입 장치 관리」가 4.04±0.93, 「110. 약물투여에 필요한 계

산 시행」이 3.97±0.97,「114. 대상자에게 약물에 대해 교육」이 3.99±0.99,「113. 말초정맥관 삽입, 유지, 제거」가 3.93±1.01,「115. 고위험 약품관리(마약관리)」가 3.76±1.09,「112. 약품관리」가 3.97±0.98 순이었다.

<표 Ⅳ-8> 약물 및 비경구요법

번호	항목	중요도 (Mean±SD)	수행도 (Mean±SD)
109	투약의 적절성과 정확성	4.85±0.44	4.11±0.89
110	약물투여에 필요한 계산 시행	4.83±0.47	3.97±0.97
111	정맥주입 장치 관리	4.85±0.44	4.04±0.93
112	약품관리	4.73±0.57	3.97±0.98
113	말초정맥관 삽입, 유지, 제거	4.77±0.55	3.93±1.01
114	대상자에게 약물에 대해 교육	4.78±0.47	3.99±0.99
115	고위험 약품관리(마약관리)	4.75±0.54	3.76±1.09

※문항별 결측치 제외

약물 및 비경구요법 직무 항목에 대해 신규 간호사의 직무 중요도 및 수행도를 y축은 중요도, x축을 수행도로 설정하고 3점을 기준선으로 IPA 분석한 결과는 [그림 Ⅳ-7]과 같다. 약물 및 비경구요법 영역의 7개 직무 모두 2사분위에 위치하고 있어 신규 간호사의 직무 중요도와 수행도 모두 3점 이상인 것으로 나타났다.

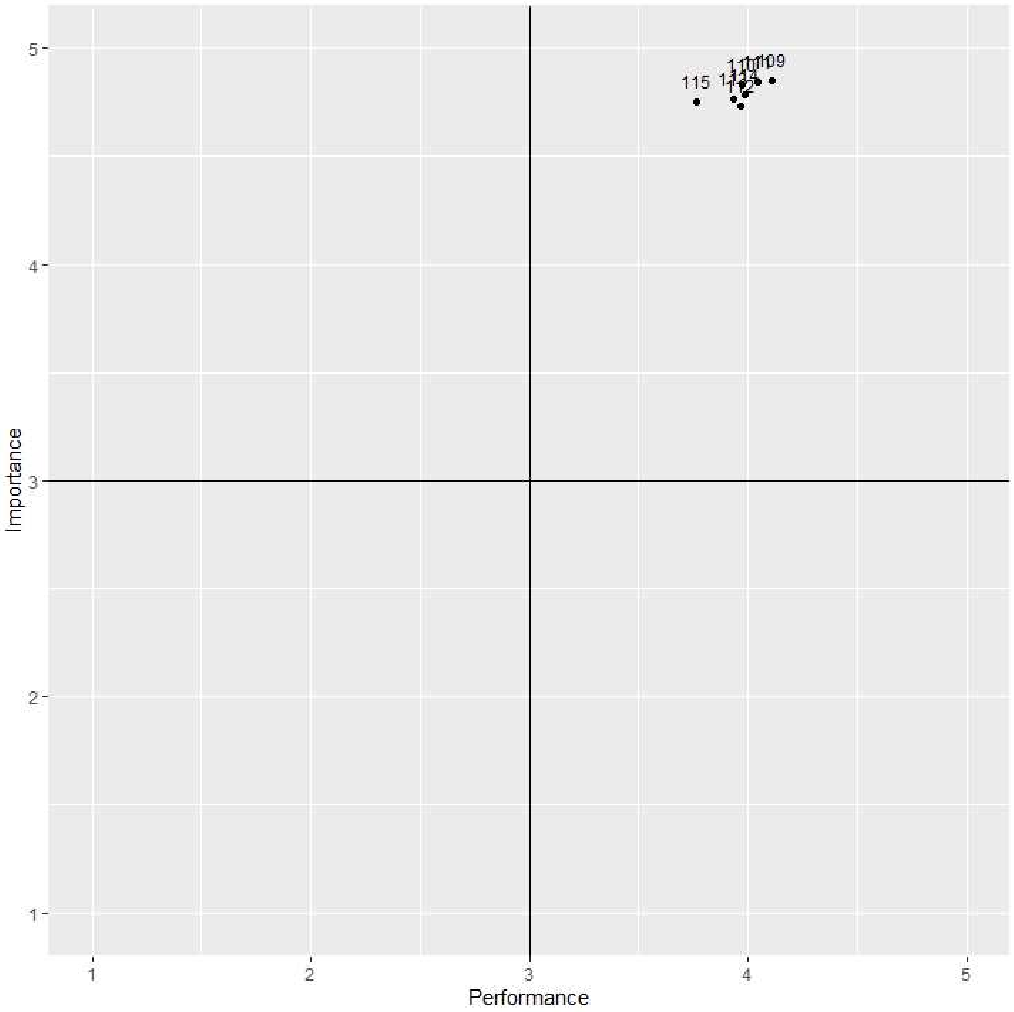

[그림 Ⅳ-7] 약물 및 비경구요법 직무의 IPA

사) 심리사회적 통합 유지

심리사회적 통합유지 영역의 직무에 대한 신규간호사 직무 중요도와 수행도는 다음과 같다<표 Ⅳ-9>.

신규 간호사 직무 중요도 순으로 살펴본 결과, 「124. 치료적 의사소통 기법」이 4.26±0.87, 「123. 임종간호와 교육」이 4.13±0.90, 「116. 이상행동 사정 및 간호」가 4.12±0.84, 「118. 학대 또는 방임 대상자를 확인하고 적절하게 중재」 가 4.06±0.91, 「117. 폭력의 잠재성 평가 및 예방」이 4.02±0.93, 「121. 정신질환자 간호」가 3.98±0.99, 「119. 중독 사정 및 간호」가 3.97±0.96, 「122. 아동 정신질환자 간호」가 3.95±1.01, 「120. 정신사회건강문제 간호 및 교육」이 3.86±1.01 순이었다.

신규 간호사 직무 수행도 순으로 살펴본 결과, 「124. 치료적 의사소통 기법」이 3.37±1.00, 「116. 이상행동 사정 및 간호」가 3.28±0.96, 「123. 임종간호와 교육」이 3.20±1.00, 「117. 폭력의 잠재성 평가 및 예방」이 3.16±0.94, 「119. 중독 사정 및 간호」가 3.14±1.01, 「118. 학대 또는 방임 대상자를 확인하고 적절하게 중재」가 3.12±0.98, 「121. 정신질환자 간호」가 3.11±0.97, 「120. 정신사회건강문제 간호 및 교육」이 3.10±0.98, 「122. 아동 정신질환자 간호」가 3.05±1.00 순이었다.

<표 Ⅳ-9> 심리사회적 통합 유지

번호	항목	중요도 (Mean±SD)	수행도 (Mean±SD)
116	이상행동 사정 및 간호	4.12±0.84	3.28±0.96
117	폭력의 잠재성 평가 및 예방	4.02±0.93	3.16±0.94
118	학대 또는 방임 대상자를 확인하고 적절하게 중재	4.06±0.91	3.12±0.98
119	중독 사정 및 간호	3.97±0.96	3.14±1.01
120	정신사회건강문제 간호 및 교육	3.86±1.01	3.10±0.98
121	정신질환자 간호	3.98±0.99	3.11±0.97
122	아동 정신질환자 간호	3.95±1.01	3.05±1.00
123	임종간호와 교육	4.13±0.90	3.20±1.00
124	치료적 의사소통 기법	4.26±0.87	3.37±1.00

※문항별 결측치 제외

심리사회적 통합 유지 직무 항목에 대해 신규 간호사의 직무 중요도 및 수행도를 y축은 중요도, x축을 수행도로 설정하고 3점을 기준선으로 IPA 분석한 결과는 [그림 Ⅳ-8]과 같다. 심리사회적 통합 유지 영역의 9개 직무 모두 2사분위에 위치하고 있어 신규 간호사의 직무 중요도와 수행도 모두 3점 이상인 것으로 나타났다.

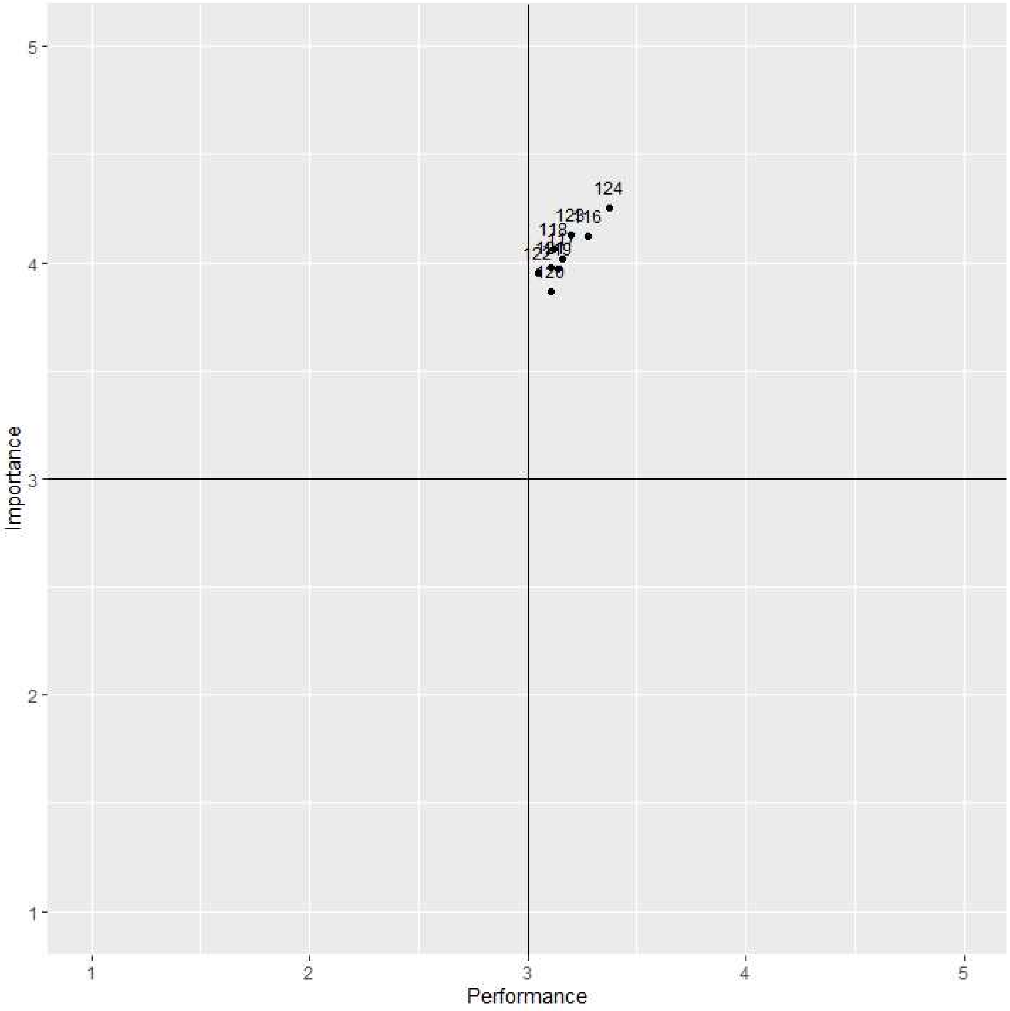

[그림 Ⅳ-8] 심리사회적 통합 유지 직무의 IPA

아) 건강유지 및 증진

건강유지 및 증진 영역의 직무에 대한 신규간호사 직무 중요도와 수행도는 다음과 같다<표 Ⅳ-10>.

신규 간호사 직무 중요도 순으로 살펴본 결과, 「127. 고위험 건강행위의 예방 및 치료에 대한 정보(금연, 안전한 성 행위, 바늘교환)제공」이 3.70±0.97, 「126. 건강증진 및 유지관리에 대한 정보(예방접종 등)제공」이 3.52±1.01, 「132. 치료계획을 결정하기 위해 가족 역동성(family dynamics) (가족구조, 결속, 의사소통, 경계, 대처기전) 사정」이 3.39±1.07, 「131. 재난간호」가 3.35±1.14, 「125. (지역사회)건강교육계획」이 3.33±1.10, 「128. 성 건강증진 간호」가 3.33±1.04, 「129. 문화간호」가

3.14±1.13,「130. 산업과 환경 간호」가 3.14±1.12 순이었다.

신규 간호사 직무 수행도 순으로 살펴본 결과,「127. 고위험 건강행위의 예방 및 치료에 대한 정보(금연, 안전한 성 행위, 바늘교환)제공」이 3.28±1.01,「126. 건강증진 및 유지관리에 대한 정보(예방접종 등)제공」이 3.17±1.00,「134. 건강위험요인 사정과 교육」이 3.11±1.01,「128. 성 건강증진 간호」가 3.03±0.99로 4항목에서 평균 3점을 상회하셨으나,「129. 문화간호」가 2.96±1.05,「125. (지역사회)건강교육 계획」 이 2.96±1.02, 「132. 치료계획을 결정하기 위해 가족 역동성(family dynamics) (가족구조, 결속, 의사소통, 경계, 대처기전) 사정」이 2.94±1.01,「130. 산업과 환경 간호」가 2.91±1.06,「131. 재난간호」가 2.90±1.10,「133. 가정환경에서 대상자를 관리할 수 있는 역량(장비, 지역사회 자원) 평가 2.90±1.02」순으로 6개 항목에서 평균 3점 미만인 것으로 나타났다.

<표 Ⅳ-10> 건강유지 및 증진

번호	항목	중요도 (Mean±SD)	수행도 (Mean±SD)
125	(지역사회)건강교육 계획	3.33±1.10	2.96±1.02
126	건강증진 및 유지관리에 대한 정보(예방접종 등)제공	3.52±1.01	3.17±1.00
127	고위험 건강행위의 예방 및 치료에 대한 정보(금연, 안전한 성 행위, 바늘교환)제공	3.70±0.97	3.28±1.01
128	성 건강증진 간호	3.33±1.04	3.03±0.99
129	문화간호	3.14±1.13	2.96±1.05
130	산업과 환경 간호	3.14±1.12	2.91±1.06
131	재난간호	3.35±1.14	2.90±1.10
132	치료계획을 결정하기 위해 가족 역동성(family dynamics) (가족구조, 결속, 의사소통, 경계, 대처기전) 사정	3.39±1.07	2.94±1.01
133	가정환경에서 대상자를 관리할 수 있는 역량(장비, 지역사회 자원) 평가	3.33±1.07	2.90±1.02
134	건강위험요인 사정과 교육	3.64±1.03	3.11±1.01

※문항별 결측치 제외

건강유지 및 증진 직무 항목에 대해 신규 간호사의 직무 중요도 및 수행도를 y축은 중요도, x축을 수행도로 설정하고 3점을 기준선으로 IPA 분석한 결과는 [그림 Ⅳ-9]과 같다. 우선, 1사분위에 위치하고 있는「125. (지역사회)건강교육 계획」,「129. 문

화간호」,「130. 산업과 환경 간호」,「131. 재난간호」,「132. 치료계획을 결정하기 위해 가족 역동성(family dynamics) (가족구조, 결속, 의사소통, 경계, 대처기전) 사정」,「133. 가정환경에서 대상자를 관리할 수 있는 역량(장비, 지역사회 자원)」항목의 중요도는 3점 이상이나 수행도가 3점 이하인 직무로 나타났다. 그 이외의「126. 건강증진 및 유지관리에 대한 정보(예방접종 등)제공」,「127. 고위험 건강행위의 예방 및 치료에 대한 정보(금연, 안전한 성 행위, 바늘교환)제공」,「128. 성 건강증진 간호」,「134. 건강위험요인 사정과 교육」항목은 모두 2사분위에 위치하고 있어 신규간호사의 직무 중요도와 수행도 모두 3점 이상인 것으로 나타났다.

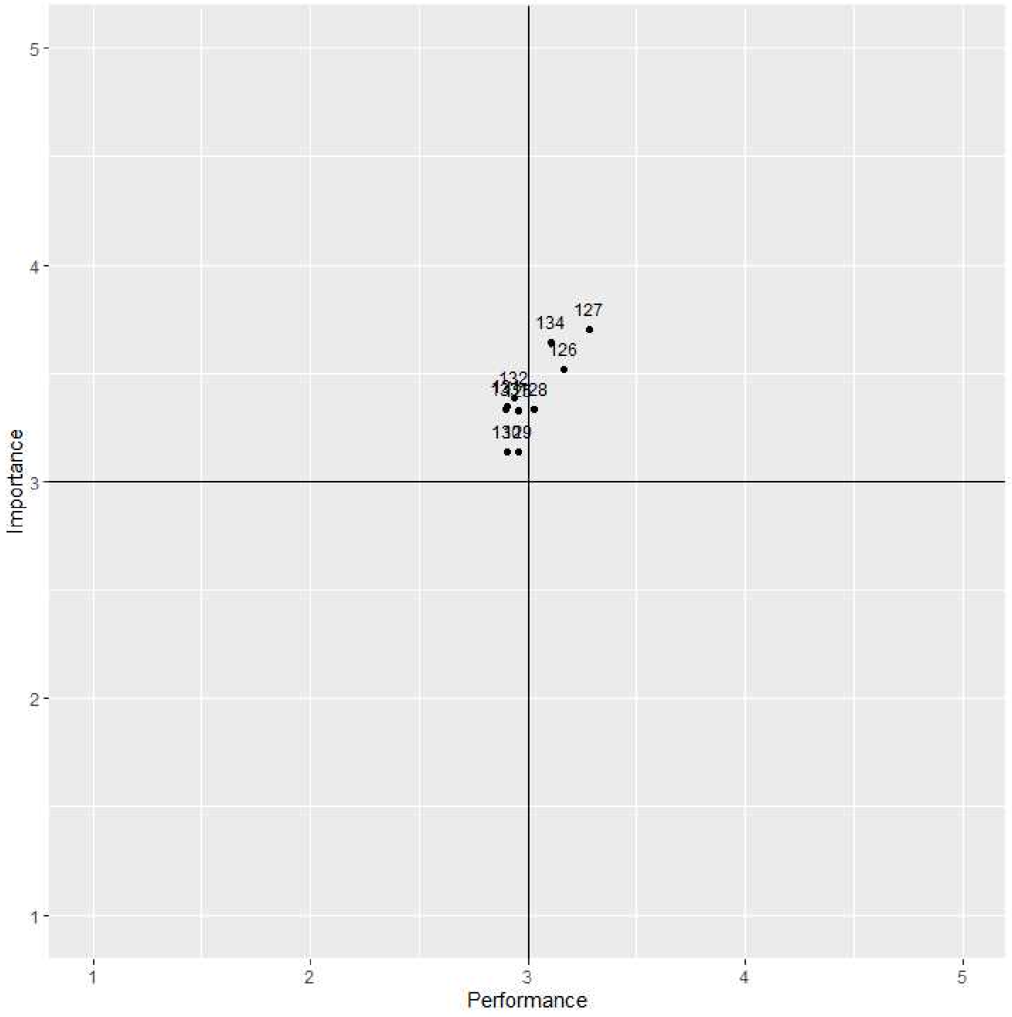

[그림 Ⅳ-9] 건강유지 및 증진 직무의 IPA

8개 직무영역과 연계된 134개 신규간호사 직무항목에 대한 IPA 분석한 결과, 간호관리와 전문성 향상 영역은 22개의 직무항목 중 16개 항목은 2사분위, 4개 항목은 1사분위, 2개 항목은 3사분위에 위치하였다. 건강증진 및 유지 영역의 10개 직무항목 중 4개 항목은 2사분위, 6개 항목은 1사분위에 위치하였다. 안전과 감염관리 영역의 3개 직무항목, 위험요인 사정 영역의 28개 직무항목, 기본간호 영역의 19개의 직무항목, 생리적 통합유지 영역의 36개 직무항목, 약물 및 비경구 요법 영역의 7개 직무항목, 심리적 통합유지 영역의 9개 직무항목은 2사분위에 위치하였다.

2사분위에 위치한 직무항목 중에서 안전과 감염관리 영역과 약물 및 비경구 요법 영역의 직무항목은 중요도와 수행도가 다른 영역에 비해서 상대적으로 높은 위치에 분포하였다. 반면에 생리적 통합유지 영역, 심리사회적 통합유지 영역, 건강유지 및 증진 영역의 직무항목의 중요도와 수행도는 안전과 감염관리 영역과 약물 및 비경구 요법 영역의 직무항목에 비해서 상대적으로 낮은 위치에 분포하였다[그림 Ⅳ-10].

1사분위에 위치하고 있는「8. 질 향상(QI) 활동에 참여」,「17. 사례관리활동 참여」,「18. 업무를 조직화하여 일을 효율적으로 관리」,「20. 간호대상에 적합한 다양한 자원 및 매체선택」,「125. (지역사회)건강교육 계획」,「129. 문화간호」,「130. 산업과 환경 간호」,「131. 재난간호」,「132. 치료계획을 결정하기 위해 가족 역동성(family dynamics) (가족구조, 결속, 의사소통, 경계, 대처기전) 사정」,「134. 건강위험요인 사정과 교육」등 10개 직무항목은 중요도는 3점 이상이나 수행도가 3점 미만이었다. 1사분위에 위치하고 있는 10개 직무항목 중「8. 질 향상(QI) 활동에 참여」,「17. 사례관리활동 참여」,「18. 업무를 조직화하여 일을 효율적으로 관리」,「20. 간호대상에 적합한 다양한 자원 및 매체선택」등 4개 직무항목은 간호관리와 전문성 향상 영역이었고,「125. (지역사회)건강교육 계획」,「129. 문화간호」,「130. 산업과 환경 간호」,「131. 재난간호」,「132. 치료계획을 결정하기 위해 가족 역동성(family dynamics) (가족구조, 결속, 의사소통, 경계, 대처기전) 사정」,「134. 건강위험요인 사정과 교육」등 6개 직무항목은 건강유지 및 증진 영역이었다. 3사분위에 위치하고 있는「9. 간호사업 평가 관련 업무수행(도구개발, 자료조사, 분석, 비교 및 사업개선)」,「21. 취약가족에게 필요한 지역사회 자원활용」등 2개 직무항목은 중요도와 수행도 모두 3점 미만으로 나타났다. 3사분위에 위치하고 있는 2개 직무항목은 건강유지 및 증진 영역이었다. 1사분위, 3사분위에 위치한 12개 직무항목 이외의 122개의 직무항목은 2사분위에 위치하고 있어서 직무 중요도와 수행도 모두 3점 이상인 것으로 나타났다.

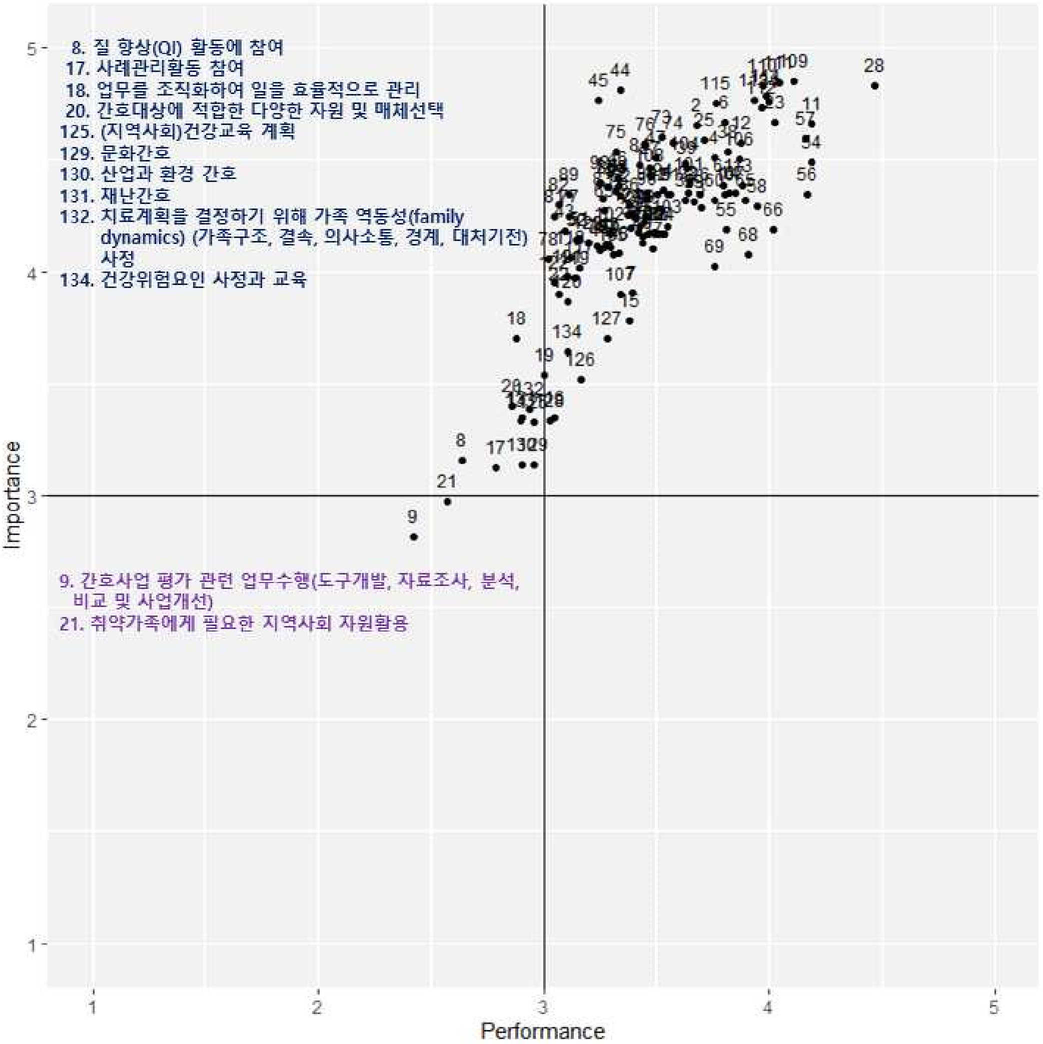

[그림 Ⅳ-10] 간호사 직무의 IPA

3) 8개 직무영역별 신규간호사 직무항목의 개선 요구도

가) 간호관리와 전문성 향상

간호관리와 전문성 향상 영역에 대한 신규간호사 직무 개선 요구도는 <표 Ⅳ-11>과 같다. 대부분의 직무에서 유지에 대한 요구도가 높았으며, 분리에 대한 요구도는 「21. 취약가족에게 필요한 지역사회 자원활용」이 35.00%, 「17. 사례관리활동 참여」가 34.62%, 「8. 질 향상(QI) 활동에 참여」가 34.62%, 「9. 간호사업 평가 관련 업무수행(도구개발, 자료조사, 분석, 비교 및 사업개선)」이 33.08%, 「16. 지역사회 간호사업의 법적 기준 및 지침에 따른 활동 참여」가 25.38%, 「20. 간호대상에 적합한 다양한 자원 및 매체선택」이 25.38%, 「18. 업무를 조직화하여 일을 효율적으로 관리」

가 19.62%,「19. 환자분류체계 관련 정보수집 및 활용」이 18.46%,「7. 간호단위 물품교환체계에 따른 물품관리」가 18.08%,「22. 전문직간 협업」이 17.31% 순으로 높게 나타났다.

통합에 대한 요구도는「20. 간호대상에 적합한 다양한 자원 및 매체선택」이 21.92%,「19. 환자분류체계 관련 정보수집 및 활용」이 18.46%,「18. 업무를 조직화하여 일을 효율적으로 관리」가 17.31%,「22. 전문직간 협업」이 17.31%,「21. 취약가족에게 필요한 지역사회 자원활용」이 13.85%,「14. 억제대 사용 시 법적 및 윤리적 간호」가 13.85%,「15. 일차의료기반의 보건간호」가 13.46%,「16. 지역사회 간호사업의 법적 기준 및 지침에 따른 활동 참여」가 13.08%,「7. 간호단위 물품교환체계에 따른 물품관리」가 13.08%,「10. 간호전문직 윤리 준수와 역할」이 13.08% 순으로 높게 나타났다.

삭제에 대한 요구도는「9. 간호사업 평가 관련 업무수행(도구개발, 자료조사, 분석, 비교 및 사업개선)」이 23.08%,「8. 질 향상(QI) 활동에 참여」가 16.54%,「16. 지역사회 간호사업의 법적 기준 및 지침에 따른 활동 참여」가 13.46%,「21. 취약가족에게 필요한 지역사회 자원활용」이 12.31%,「17. 사례관리활동 참여」가 10.77%,「15. 일차의료기반의 보건간호」가 9.23%,「20. 간호대상에 적합한 다양한 자원 및 매체선택」이 8.08%,「19. 환자분류체계 관련 정보수집 및 활용」이 7.69%,「7. 간호단위 물품교환체계에 따른 물품관리」가 5.38%,「18. 업무를 조직화하여 일을 효율적으로 관리」가 4.23% 순으로 높게 나타났다.

<표 Ⅳ-11> 간호관리와 전문성 향상

(N=260)

번호	항목	유지 n (%)	분리 n (%)	통합 n (%)	삭제 n (%)	결측 n (%)
1	인수인계시행	209 (80.38)	25 (9.62)	21 (8.08)	4 (1.54)	1 (0.38)
2	법적 실무범위 내에서 간호 수행	219 (84.23)	16 (6.15)	24 (9.23)	0 (0.00)	1 (0.38)
3	기록시 표준화된 약어사용	216 (83.08)	13 (5.00)	29 (11.15)	2 (0.77)	0 (0.00)
4	지침에 따라 간호기록	215 (82.69)	19 (7.31)	25 (9.62)	1 (0.38)	0 (0.00)
5	입원, 전동, 퇴원	200 (76.92)	35 (13.46)	25 (9.62)	0 (0.00)	0 (0.00)
6	장비를 적절하고 안전하게 사용	207 (79.62)	23 (8.85)	28 (10.77)	0 (0.00)	2 (0.77)
7	간호단위 물품교환체계에 따른 물품관리	165 (63.46)	47 (18.08)	34 (13.08)	14 (5.38)	0 (0.00)
8	질 향상(QI) 활동에 참여	94 (36.15)	90 (34.62)	32 (12.31)	43 (16.54)	1 (0.38)

번호	항목	유지<br>n (%)	분리<br>n (%)	통합<br>n (%)	삭제<br>n (%)	결측<br>n (%)
9	간호사업 평가 관련 업무수행(도구개발, 자료조사, 분석, 비교 및 사업개선)	86 (33.08)	86 (33.08)	26 (10.00)	60 (23.08)	2 (0.77)
10	간호전문직 윤리 준수와 역할	200 (76.92)	23 (8.85)	34 (13.08)	2 (0.77)	1 (0.38)
11	대상자의 개인정보 및 사생활 보호	223 (85.77)	11 (4.23)	25 (9.62)	1 (0.38)	0 (0.00)
12	대상자에게 치료 및 절차에 따라 적절한 설명을 하고 동의를 획득하였는지 확인	218 (83.85)	12 (4.62)	27 (10.38)	2 (0.77)	1 (0.38)
13	환자의 권리와 책임에 관하여 대상자에게 교육 제공	213 (81.92)	13 (5.00)	30 (11.54)	3 (1.15)	1 (0.38)
14	억제대 사용 시 법적 및 윤리적 간호	209 (80.38)	10 (3.85)	36 (13.85)	1 (0.38)	4 (1.54)
15	일차의료기반의 보건간호	157 (60.38)	43 (16.54)	35 (13.46)	24 (9.23)	1 (0.38)
16	지역사회 간호사업의 법적 기준 및 지침에 따른 활동 참여	124 (47.69)	66 (25.38)	34 (13.08)	35 (13.46)	1 (0.38)
17	사례관리활동 참여	111 (42.69)	90 (34.62)	29 (11.15)	28 (10.77)	2 (0.77)
18	업무를 조직화하여 일을 효율적으로 관리	152 (58.46)	51 (19.62)	45 (17.31)	11 (4.23)	1 (0.38)
19	환자분류체계 관련 정보수집 및 활용	143 (55.00)	48 (18.46)	48 (18.46)	20 (7.69)	1 (0.38)
20	간호대상에 적합한 다양한 자원 및 매체 선택	115 (44.23)	66 (25.38)	57 (21.92)	21 (8.08)	1 (0.38)
21	취약가족에게 필요한 지역사회 자원활용	100 (38.46)	91 (35.00)	36 (13.85)	32 (12.31)	1 (0.38)
22	전문직간 협업	165 (63.46)	45 (17.31)	45 (17.31)	5 (1.92)	0 (0.00)

나) 안전과 감염관리

안전과 감염관리 영역에 대한 신규간호사 직무 개선 요구도는 다음과 같이 나타났다<표 Ⅳ-12>. 유지에 대한 요구도는「23. 안전한 환경 제공」이 83.85%,「24. 감염관리」가 83.46%,「25. 위험물질과 유해물질 관리」가 75.38% 순으로 높게 나타났으며, 분리에 대한 요구도는「25. 위험물질과 유해물질 관리」가 11.54%,「23. 안전한 환경 제공」이 5.77%,「24. 감염관리」가 5.77% 순으로 나타났다. 통합에 대한 요구도는「25. 위험물질과 유해물질 관리」가 11.92%,「24. 감염관리」가 10.77%,「23. 안전한 환경 제공」이 10.00% 순이었고, 삭제에 대한 요구도는「25. 위험물질과 유해물질 관리」가 1.15%,「23. 안전한 환경 제공」이 0.38%,「24. 감염관리」가 0.00% 순으로 나타났다.

<표 Ⅳ-12> 안전과 감염관리

번호	항목	유지 n (%)	분리 n (%)	통합 n (%)	삭제 n (%)	결측 n (%)
23	안전한 환경 제공	218 (83.85)	15 (5.77)	26 (10.00)	1 (0.38)	0 (0.00)
24	감염관리	217 (83.46)	15 (5.77)	28 (10.77)	0 (0.00)	0 (0.00)
25	위험물질과 유해물질 관리	196 (75.38)	30 (11.54)	31 (11.92)	3 (1.15)	0 (0.00)

다) 위험요인 사정

위험요인 사정 영역에 대한 신규간호사 직무 개선 요구도는 다음과 같이 나타났다 <표 Ⅳ-13>.

대부분의 직무에서 유지에 대한 요구도가 높았으며, 분리에 대한 요구도는「50. 산후관리와 교육」이 16.54%,「51. 고위험 임부간호」가 16.54%,「53. 고위험 산모간호」가 16.54%,「52. 고위험 산부간호」가 16.54%,「49. 분만 중 간호와 교육」이 15.77%, 「48. 산전간호와 교육」이 14.62%, 「41. 태아건강사정 및 간호」가 13.85%, 「37. 성 건강 간호」가 13.46%, 「42. 신생아 건강사정 및 간호」가 13.08%,「43. 고위험신생아 건강사정 및 간호」가 13.08% 순으로 높게 나타났다.

통합에 대한 요구도는「52. 고위험 산부간호」가 18.46%,「51. 고위험 임부간호」가 18.08%,「53. 고위험 산모간호」가 18.08%,「43. 고위험신생아 건강사정 및 간호」가 18.08%,「33. 학령기 간호」가 17.31%,「32. 학령전기 간호」가 17.31%,「43. 신생아 건강사정 및 간호」가 16.92%,「34. 청소년 간호」가 16.92% 순으로

나타났으며, 삭제에 대한 요구도는「43. 고위험신생아 건강사정 및 간호」가 2.69%,「42. 신생아 건강사정 및 간호」가 2.69%,「41. 태아건강사정 및 간호」가 2.69%,「49. 분만 중 간호와 교육」이 1.92%,「40. 생식기 건강사정」이 1.92%,「37. 성 건강 간호」가 1.54%,「30. 영아 간호」가 1.54%,「46. 진단검사 간호」가 1.54%,「52. 고위험 산부간호」가 1.15%,「51. 고위험 임부간호」가 1.15%,「53. 고위험 산모간호」가 1.15%,「31. 유아 간호」가 1.15%,「29. 신생아 간호」가 1.15%,「50. 산후관리와 교육」가 1.15% 순으로 높게 나타났다.

<표 Ⅳ-13> 위험요인 사정

번호	항목	유지<br>n (%)	분리<br>n (%)	통합<br>n (%)	삭제<br>n (%)	결측<br>n (%)
26	간호계획, 진료지침 수행	199<br>(76.54)	14<br>(5.38)	40<br>(15.38)	2<br>(0.77)	5<br>(1.92)
27	대상자의 건강문제에 대해 우선순위 결정	205<br>(78.85)	16<br>(6.15)	35<br>(13.46)	0<br>(0.00)	4<br>(1.54)
28	활력징후 사정	226<br>(86.92)	5<br>(1.92)	24<br>(9.23)	2<br>(0.77)	3<br>(1.15)
29	신생아 간호	183<br>(70.38)	31<br>(11.92)	42<br>(16.15)	3<br>(1.15)	1<br>(0.38)
30	영아 간호	182<br>(70.00)	31<br>(11.92)	42<br>(16.15)	4<br>(1.54)	1<br>(0.38)
31	유아 간호	182<br>(70.00)	32<br>(12.31)	42<br>(16.15)	3<br>(1.15)	1<br>(0.38)
32	학령전기 간호	183<br>(70.38)	31<br>(11.92)	45<br>(17.31)	0<br>(0.00)	1<br>(0.38)
33	학령기 간호	181<br>(69.62)	32<br>(12.31)	45<br>(17.31)	1<br>(0.38)	1<br>(0.38)
34	청소년 간호	181<br>(69.62)	33<br>(12.69)	44<br>(16.92)	1<br>(0.38)	1<br>(0.38)
35	폐경기 여성 간호	184<br>(70.77)	31<br>(11.92)	42<br>(16.15)	2<br>(0.77)	1<br>(0.38)
36	노인간호	193<br>(74.23)	29<br>(11.15)	36<br>(13.85)	1<br>(0.38)	1<br>(0.38)
37	성 건강 간호	177<br>(68.08)	35<br>(13.46)	42<br>(16.15)	4<br>(1.54)	2<br>(0.77)
38	건강력 사정	201<br>(77.31)	22<br>(8.46)	35<br>(13.46)	1<br>(0.38)	1<br>(0.38)
39	신체검진 수행 및 결과 해석	199<br>(76.54)	18<br>(6.92)	40<br>(15.38)	2<br>(0.77)	1<br>(0.38)
40	생식기 건강사정	183<br>(70.38)	33<br>(12.69)	37<br>(14.23)	5<br>(1.92)	2<br>(0.77)
41	태아건강사정 및 간호	175<br>(67.31)	36<br>(13.85)	41<br>(15.77)	7<br>(2.69)	1<br>(0.38)
42	신생아 건강사정 및 간호	174<br>(66.92)	34<br>(13.08)	44<br>(16.92)	7<br>(2.69)	1<br>(0.38)
43	고위험신생아 건강사정 및 간호	171<br>(65.77)	34<br>(13.08)	47<br>(18.08)	7<br>(2.69)	1<br>(0.38)
44	심폐소생술 간호	193<br>(74.23)	28<br>(10.77)	36<br>(13.85)	1<br>(0.38)	2<br>(0.77)
45	응급간호	196<br>(75.38)	27<br>(10.38)	35<br>(13.46)	1<br>(0.38)	1<br>(0.38)
46	진단검사 간호	190<br>(73.08)	32<br>(12.31)	33<br>(12.69)	4<br>(1.54)	1<br>(0.38)

번호	항목	유지<br>n (%)	분리<br>n (%)	통합<br>n (%)	삭제<br>n (%)	결측<br>n (%)
47	수술간호	196<br>(75.38)	26<br>(10.00)	35<br>(13.46)	2<br>(0.77)	1<br>(0.38)
48	산전간호와 교육	179<br>(68.85)	38<br>(14.62)	41<br>(15.77)	1<br>(0.38)	1<br>40.38)
49	분만 중 간호와 교육	175<br>(67.31)	41<br>(15.77)	38<br>(14.62)	5<br>(1.92)	1<br>(0.38)
50	산후관리와 교육	174<br>(66.92)	43<br>(16.54)	39<br>(15.00)	3<br>(1.15)	1<br>(0.38)
51	고위험 임부간호	166<br>(63.85)	43<br>(16.54)	47<br>(18.08)	3<br>(1.15)	1<br>(0.38)
52	고위험 산부간호	165<br>(63.46)	43<br>(16.54)	48<br>(18.46)	3<br>(1.15)	1<br>(0.38)
53	고위험 산모간호	166<br>(63.85)	43<br>(16.54)	47<br>(18.08)	3<br>(1.15)	1<br>(0.38)

라) 기본간호

기본간호 영역에 대한 신규간호사 직무 개선 요구도는 다음과 같다<표 Ⅳ-14>.

대부분의 질문에서 유지에 대한 요구도가 높았으며, 분리에 대한 요구도는 「63. 요루 및 장루관리」가 18.08%, 「62. 투석대상자 관리」가 17.31%, 「64. 기관절개부 관리」가 15.77%, 「72. 관절대치술 환자 간호」가 13.46%, 「71. 척추손상 및 신경질환 대상자의 간호」가 13.46%, 「70. 외과적 장치 관리」가 13.08%, 「59. 질환별 영양문제의 사정 및 관리」가 10.38%, 「61. 배뇨장애 대상자 사정 및 간호」가 9.62%, 「58. 영양사정 및 관리」가 9.23%, 「68. 이동간호」가 9.23% 순으로 높게 나타났다.

통합에 대한 요구도는 「63. 요루 및 장루관리」가 10.77%, 「62. 투석대상자 관리」가 11.15%, 「64. 기관절개부 관리」가 11.54%, 「72. 관절대치술 환자 간호」가 13.46%, 「71. 척추손상 및 신경질환 대상자의 간호」가 13.85%, 「70. 외과적 장치 관리」가 13.08%, 「59. 질환별 영양문제의 사정 및 관리」가 15.77%, 「61. 배뇨장애 대상자 사정 및 간호」가 14.23%, 「58. 영양사정 및 관리」가 16.15%, 「68. 이동간호」가 13.46% 순으로 높게 나타났다.

삭제에 대한 요구도는 「68. 이동간호」가 3.85%, 「70. 외과적 장치 관리」가 2.69%, 「69. 활동과 자기돌봄 장애 사정 및 간호」가 2.69%, 「62. 투석대상자 관리」가 1.92%, 「71. 척추손상 및 신경질환 대상자의 간호」가 1.92%, 「64. 기관절개부 관리」가 1.54%, 「72. 관절대치술 환자 간호」가 1.54%, 「59. 질환별 영양문제의 사정 및 관리」가 1.54%, 「65. 배변관리」가 1.54%, 「66. 수면과 휴식 간호」가 1.54% 순으로 나타났다.

<표 Ⅳ-14> 기본간호

번호	항목	유지 n (%)	분리 n (%)	통합 n (%)	삭제 n (%)	결측 n (%)
54	체온유지간호	212 (81.54)	5 (1.92)	38 (14.62)	1 (0.38)	4 (1.54)
55	세척(irrigation)(방광, 귀, 눈) 수행	202 (77.69)	15 (5.77)	37 (14.23)	3 (1.15)	3 (1.15)
56	개인위생간호	208 (80)	5 (1.92)	42 (16.15)	2 (0.77)	3 (1.15)
57	섭취량 및 배설량 사정과 간호	210 (80.77)	11 (4.23)	36 (13.85)	0 (0.00)	3 (1.15)
58	영양사정 및 관리	188 (72.31)	24 (9.23)	42 (16.15)	1 (0.38)	5 (1.92)
59	질환별 영양문제의 사정 및 관리	184 (70.77)	27 (10.38)	41 (15.77)	4 (1.54)	4 (1.54)
60	섭취장애 대상자 사정 및 간호	191 (73.46)	21 (8.08)	42 (16.15)	1 (0.38)	5 (1.92)
61	배뇨장애 대상자 사정 및 간호	193 (74.23)	25 (9.62)	37 (14.23)	1 (0.38)	4 (1.54)
62	투석대상자 관리	177 (68.08)	45 (17.31)	29 (11.15)	5 (1.92)	4 (1.54)
63	요루 및 장루관리	179 (68.85)	47 (18.08)	28 (10.77)	1 (0.38)	5 (1.92)
64	기관절개부 관리	181 (69.62)	41 (15.77)	30 (11.54)	4 (1.54)	4 (1.54)
65	배변관리	190 (73.08)	19 (7.31)	41 (15.77)	4 (1.54)	6 (2.31)
66	수면과 휴식 간호	194 (74.62)	11 (4.23)	46 (17.69)	4 (1.54)	5 (1.92)
67	피부통합성 사정 및 간호	200 (76.92)	16 (6.15)	37 (14.23)	1 (0.38)	6 (2.31)
68	이동간호	186 (71.54)	24 (9.23)	35 (13.46)	10 (3.85)	5 (1.92)
69	활동과 자기돌봄 장애 사정 및 간호	183 (70.38)	20 (7.69)	45 (17.31)	7 (2.69)	5 (1.92)
70	외과적 장치 관리	180 (69.23)	34 (13.08)	34 (13.08)	7 (2.69)	5 (1.92)
71	척추손상 및 신경질환 대상자의 간호	179 (68.85)	35 (13.46)	36 (13.85)	5 (1.92)	5 (1.92)
72	관절대치술 환자 간호	181 (69.62)	35 (13.46)	35 (13.46)	4 (1.54)	5 (1.92)

마) 생리적 통합유지

생리적 통합유지 영역에 대한 신규간호사 직무 개선 요구도는 다음과 같다<표 Ⅳ-15>.

대부분의 질문에서 유지에 대한 요구도가 높았으며, 분리에 대한 요구도는 「78. 고위험 신생아 보육기 적용간호」가 17.69%, 「77. 태아질식 증상과 징후 사정 및 간호」가 16.54%, 「107. 재활간호서비스」가 15.00%, 「79. 호흡재활관리」가 14.62%, 「81. 순환 보조장치 관리」가 14.62%, 「108. (법정) 감염질환자의 전파예방 및 간호」가 14.23%, 「98. 두개내압 상승 환자의 간호」가 13.46%, 「89. 심장 수술 후 간호」가 13.08%, 「102. 화상환자 간호중재」가 13.08%, 「103. 계통별 신생물 질환 대상자

간호」가 13.08% 순으로 높게 나타났다.

통합에 대한 요구도는「74. 호흡증진 중재」가 16.15%,「108. (법정) 감염질환자의 전파예방 및 간호」가 15.00%,「83. 조직 관류 장애 대상자 사정 및 간호」가 14.23%,「73. 호흡기능장애 대상자 간호」가 14.23%,「107. 재활간호서비스」가 13.85%,「75. 호흡보조장치 관리」가 13.85%,「95. 생식기 질환/생식기 건강문제를 가진 대상자 간호」가 13.85%,「84. 체액 전해질 불균형 사정 및 간호」가 13.46%,「103. 피부질환 대상자의 간호중재」가 13.46%,「105. 배액장치 관리」가 13.46% 순으로 높게 나타났다.

삭제에 대한 요구도는「105. 계통별 신생물 질환 대상자 간호」가 4.23%,「78. 고위험 신생아 보육기 적용간호」가 3.85%,「107. 재활간호서비스」가 3.08%,「7. 호흡재활관리」가 3.08%,「9. 순환 보조장치 관리」가 2.31%,「30. 화상환자 간호중재」가 2.31%,「89. 심장 수술 후 간호」가 2.31%,「77. 태아질식 증상과 징후 사정 및 간호」가 1.92%,「75. 호흡보조장치 관리」가 1.54%,「80. 심전도 관리」가 1.54%,「82. 동맥관 관리」가 1.15%,「97. 감각기능 장애를 가진 대상자 사정 및 간호」가 1.15% 순으로 높게 나타났다.

<표 Ⅳ-15> 생리적 통합유지

번호	항목	유지	분리	통합	삭제	결측
		n (%)	n (%)	n (%)	n (%)	n (%)
73	호흡기능장애 대상자 간호	199 (76.54)	21 (8.08)	37 (14.23)	0 (0.00)	3 (1.15)
74	호흡증진 중재	193 (74.23)	22 (8.46)	42 (16.15)	0 (0.00)	3 (1.15)
75	호흡보조장치 관리	187 (71.92)	30 (11.54)	36 (13.85)	4 (1.54)	3 (1.15)
76	중심정맥관 관리	198 (76.15)	27 (10.38)	32 (12.31)	0 (0.00)	3 (1.15)
77	태아질식 증상과 징후 사정 및 간호	177 (68.08)	43 (16.54)	33 (12.69)	5 (1.92)	2 (0.77)
78	고위험 신생아 보육기 적용간호	168 (64.62)	46 (17.69)	32 (12.31)	10 (3.85)	4 (1.54)
79	호흡재활관리	180 (69.23)	38 (14.62)	31 (11.92)	8 (3.08)	3 (1.15)
80	심전도 관리	193 (74.23)	26 (10.00)	34 (13.08)	4 (1.54)	3 (1.15)
81	순환 보조장치 관리	181 (69.62)	38 (14.62)	33 (12.69)	6 (2.31)	2 (0.77)
82	동맥관 관리	189 (72.69)	33 (12.69)	33 (12.69)	3 (1.15)	2 (0.77)
83	조직 관류 장애 대상자 사정 및 간호	196 (75.38)	23 (8.85)	37 (14.23)	2 (0.77)	2 (0.77)
84	체액 전해질 불균형 사정 및 간호	202 (77.69)	20 (7.69)	35 (13.46)	1 (0.38)	2 (0.77)
85	활동 지속성 장애 대상자 사정 및 간호	205 (78.85)	23 (8.85)	29 (11.15)	1 (0.38)	2 (0.77)

번호	항목	유지<br>n (%)	분리<br>n (%)	통합<br>n (%)	삭제<br>n (%)	결측<br>n (%)
86	정맥 순환증진 장치 간호	205 (78.85)	19 (7.31)	33 (12.69)	1 (0.38)	2 (0.77)
87	혈액 기능장애 대상자 간호	205 (78.85)	19 (7.31)	34 (13.08)	0 (0.00)	2 (0.77)
88	순환 기능장애 대상자 간호	206 (79.23)	18 (6.92)	33 (12.69)	1 (0.38)	2 (0.77)
89	심장 수술 후 간호	194 (74.62)	34 (13.08)	24 (9.23)	6 (2.31)	2 (0.77)
90	소화 기능장애 대상자 간호	214 (82.31)	16 (6.15)	26 (10.00)	0 (0.00)	4 (1.54)
91	배뇨장애를 가진 대상자 간호	213 (81.92)	16 (6.15)	28 (10.77)	0 (0.00)	3 (1.15)
92	당질 대사장애 대상자 간호	210 (80.77)	18 (6.92)	30 (11.54)	0 (0.00)	2 (0.77)
93	당뇨병 합병증 예방을 위한 간호	213 (81.92)	12 (4.62)	33 (12.69)	1 (0.38)	1 (0.38)
94	내분비계 장애 대상자 간호	212 (81.54)	15 (5.77)	32 (12.31)	0 (0.00)	1 (0.38)
95	생식기 질환/생식기 건강문제를 가진 대상자 간호	201 (77.31)	20 (7.69)	36 (13.85)	1 (0.38)	2 (0.77)
96	면역손상 대상자 사정 및 간호	203 (78.08)	24 (9.23)	32 (12.31)	0 (0.00)	1 (0.38)
97	감각기능 장애를 가진 대상자 사정 및 간호	199 (76.54)	25 (9.62)	32 (12.31)	3 (1.15)	1 (0.38)
98	두개내압 상승 환자의 간호	195 (75)	35 (13.46)	28 (10.77)	1 (0.38)	1 (0.38)
99	신경계 질환별 간호중재	206 (79.23)	25 (9.62)	28 (10.77)	0 (0.00)	1 (0.38)
100	운동기능 장애 간호중재	201 (77.31)	28 (10.77)	28 (10.77)	2 (0.77)	1 (0.38)
101	상처간호 수행 및 드레싱교환	197 (75.77)	30 (11.54)	29 (11.15)	2 (0.77)	2 (0.77)
102	화상환자 간호중재	190 (73.08)	34 (13.08)	29 (11.15)	6 (2.31)	1 (0.38)
103	피부질환 대상자의 간호중재	202 (77.69)	19 (7.31)	35 (13.46)	2 (0.77)	2 (0.77)
104	배액장치 관리	204 (78.46)	17 (6.54)	35 (13.46)	1 (0.38)	3 (1.15)
105	계통별 신생물 질환 대상자 간호	184 (70.77)	34 (13.08)	29 (11.15)	11 (4.23)	2 (0.77)
106	통증간호	212 (81.54)	12 (4.62)	34 (13.08)	0 (0.00)	2 (0.77)
107	재활간호서비스	175 (67.31)	39 (15.00)	36 (13.85)	8 (3.08)	2 (0.77)
108	(법정) 감염질환자의 전파예방 및 간호	181 (69.62)	37 (14.23)	39 (15.00)	2 (0.77)	1 (0.38)

바) 약물 및 비경구요법

약물 및 비경구요법 항목에 대한 신규간호사 직무 개선 요구도는 다음과 같다<표 Ⅳ-16>.

대부분의 질문에서 유지에 대한 요구도가 높았으며, 분리에 대한 요구도는 「115. 고위험 약품관리(마약관리)」가 9.62%, 「114. 대상자에게 약물에 대해 교육」이

6.92%, 「110. 약물투여에 필요한 계산 시행」이 5.38%, 「112. 약품관리」가 5.00%, 「113. 말초정맥관 삽입, 유지, 제거」가 4.62%, 「111. 정맥주입 장치 관리」가 3.85%, 「109. 투약의 적절성과 정확성」이 3.46% 순으로 나타났다.

통합에 대한 요구도는 「112. 약품관리」가 12.31%, 「115. 고위험 약품관리(마약관리)」가 11.92%, 「114. 대상자에게 약물에 대해 교육」이 10.38%, 「110. 약물투여에 필요한 계산 시행」이 10.38%, 「113. 말초정맥관 삽입, 유지, 제거」가 10.00%, 「111. 정맥주입 장치 관리」가 10.00%, 「109. 투약의 적절성과 정확성」이 10.00% 순으로 나타났다.

삭제에 대한 요구도는 「115. 고위험 약품관리(마약관리)」가 0.77%, 「113. 말초정맥관 삽입, 유지, 제거」가 0.38%, 「109. 투약의 적절성과 정확성」이 0.38% 순으로 나타났으며, 「111. 정맥주입 장치 관리」, 「112. 약품관리」, 「114. 대상자에게 약물에 대해 교육」, 「110. 약물투여에 필요한 계산 시행」에서 0.00%로 나타났다.

<표 Ⅳ-16> 약물 및 비경구요법

번호	항목	유지<br>n (%)	분리<br>n (%)	통합<br>n (%)	삭제<br>n (%)	결측<br>n (%)
109	투약의 적절성과 정확성	222<br>(85.38)	9<br>(3.46)	26<br>(10.00	1<br>(0.38)	2<br>(0.77)
110	약물투여에 필요한 계산 시행	218<br>(83.85)	14<br>(5.38)	27<br>(10.38)	0<br>(0.00)	1<br>(0.38)
111	정맥주입 장치 관리	222<br>(85.38)	10<br>(3.85)	26<br>(10.00	0<br>(0.00)	2<br>(0.77)
112	약품관리	213<br>(81.92)	13<br>(5)	32<br>(12.31)	0<br>(0.00)	2<br>(0.77)
113	말초정맥관 삽입, 유지, 제거	220<br>(84.62)	12<br>(4.62)	26<br>(10.00	1<br>(0.38)	1<br>(0.38)
114	대상자에게 약물에 대해 교육	214<br>(82.31)	18<br>(6.92)	27<br>(10.38)	0<br>(0.00)	1<br>(0.38)
115	고위험 약품관리(마약관리)	201<br>(77.31)	25<br>(9.62)	31<br>(11.92)	2<br>(0.77)	1<br>(0.38)

사) 심리사회적 통합 유지

심리사회적 통합 유지 항목에 대한 신규간호사 직무 개선 요구도는 다음과 같이 나타났다<표 Ⅳ-17>.

대부분의 질문에서 유지에 대한 요구도가 높았으며, 분리에 대한 요구도는 「122. 아동 정신질환자 간호」가 23.08%, 「120. 정신사회건강문제 간호 및 교육」이 21.92%, 「121. 정신질환자 간호」가 21.54%, 「118. 학대 또는 방임 대상자를 확인하고 적절하게 중재」가 21.15%, 「119. 중독 사정 및 간호」가 20.77%, 「117. 폭력의

잠재성 평가 및 예방」이 19.23%, 「123. 임종간호와 교육」이 18.46%, 「116. 이상행동 사정 및 간호」가 16.92%, 「124. 치료적 의사소통 기법」이 14.62% 순으로 높게 나타났다.

통합에 대한 요구도는 「118. 학대 또는 방임 대상자를 확인하고 적절하게 중재」가 16.15%, 「117. 폭력의 잠재성 평가 및 예방」이 16.15%, 「116. 이상행동 사정 및 간호」가 16.15%, 「120. 정신사회건강문제 간호 및 교육」이 15.77%, 「124. 치료적 의사소통 기법」이 15.00%, 「119. 중독 사정 및 간호」가 14.23%, 「122. 아동 정신질환자 간호」가 13.85%, 「121. 정신질환자 간호」가 13.85%, 「123. 임종간호와 교육」이 11.92% 순으로 나타났다.

삭제에 대한 요구도는 「118. 학대 또는 방임 대상자를 확인하고 적절하게 중재」가 2.31%, 「117. 폭력의 잠재성 평가 및 예방」이 2.31%, 「120. 정신사회건강문제 간호 및 교육」이 1.92%, 「122. 아동 정신질환자 간호」가 1.92%, 「123. 임종간호와 교육」이 1.92%, 「119. 중독 사정 및 간호」가 1.54%, 「121. 정신질환자 간호」가 1.15% 순으로 나타났으며, 「116. 이상행동 사정 및 간호」, 「124. 치료적 의사소통 기법」이 0.00%로 나타났다.

<표 Ⅳ-17> 심리사회적 통합 유지

번호	항목	유지 n (%)	분리 n (%)	통합 n (%)	삭제 n (%)	결측 n (%)
116	이상행동 사정 및 간호	171 (65.77)	44 (16.92)	42 (16.15)	0 (0.00)	3 (1.15)
117	폭력의 잠재성 평가 및 예방	161 (61.92)	50 (19.23)	42 (16.15)	6 (2.31)	1 (0.38)
118	학대 또는 방임 대상자를 확인하고 적절하게 중재	156 (60.00)	55 (21.15)	42 (16.15)	6 (2.31)	1 (0.38)
119	중독 사정 및 간호	164 (63.08)	54 (20.77)	37 (14.23)	4 (1.54)	1 (0.38)
120	정신사회건강문제 간호 및 교육	156 (60.00	57 (21.92)	41 (15.77)	5 (1.92)	1 (0.38)
121	정신질환자 간호	164 (63.08)	56 (21.54)	36 (13.85)	3 (1.15)	1 (0.38)
122	아동 정신질환자 간호	158 (60.77)	60 (23.08)	36 (13.85)	5 (1.92)	1 (0.38)
123	임종간호와 교육	174 (66.92)	48 (18.46)	31 (11.92)	5 (1.92)	2 (0.77)
124	치료적 의사소통 기법	181 (69.62)	38 (14.62)	39 (15.00)	0 (0.00)	2 (0.77)

아) 건강유지 및 증진

건강유지 및 증진 항목에 대한 신규간호사 직무 개선 요구도는 다음과 같다<표 Ⅳ-18>.

유지에 대한 요구도는「127. 고위험 건강행위의 예방 및 치료에 대한 정보(금연, 안전한 성 행위, 바늘교환)제공」이 52.69%,「126. 건강증진 및 유지관리에 대한 정보(예방접종 등)제공」이 52.31%,「134. 건강위험요인 사정과 교육」이 52.31%,「125. (지역사회)건강교육 계획」이 44.62%,「132. 치료계획을 결정하기 위해 가족 역동성(family dynamics) (가족구조, 결속, 의사소통, 경계, 대처기전) 사정」이 44.62%,「128. 성 건강증진 간호 」가 43.85%,「130. 산업과 환경 간호」가 42.69%,「131. 재난간호」가 41.92%,「133. 가정환경에서 대상자를 관리할 수 있는 역량(장비, 지역사회 자원) 평가」가 41.92%,「129. 문화간호」가 38.08% 순으로 나타났다.

분리에 대한 요구도는「133. 가정환경에서 대상자를 관리할 수 있는 역량(장비, 지역사회 자원) 평가」가 31.92%,「129. 문화간호」가 31.92%,「130. 산업과 환경 간호」가 30.38%,「131. 재난간호」가 30.38%,「128. 성 건강증진 간호」가 29.62%,「132. 치료계획을 결정하기 위해 가족 역동성(family dynamics) (가족구조, 결속, 의사소통, 경계, 대처기전) 사정」이 28.08%,「125. (지역사회)건강교육 계획」이 25.77%,「127. 고위험 건강행위의 예방 및 치료에 대한 정보(금연, 안전한 성 행위, 바늘교환)제공」이 23.08%,「134. 건강위험요인 사정과 교육」이 21.92%,「126. 건강증진 및 유지관리에 대한 정보(예방접종 등)제공」이 21.15% 순으로 나타났다.

통합에 대한 요구도는「134. 건강위험요인 사정과 교육」이 19.62%,「126. 건강증진 및 유지관리에 대한 정보(예방접종 등)제공」이 19.62%,「132. 치료계획을 결정하기 위해 가족 역동성(family dynamics) (가족구조, 결속, 의사소통, 경계, 대처기전) 사정」이 18.85%,「127. 고위험 건강행위의 예방 및 치료에 대한 정보(금연, 안전한 성 행위, 바늘교환)제공」이 18.46%,「133. 가정환경에서 대상자를 관리할 수 있는 역량(장비, 지역사회 자원) 평가」가 17.69%,「125. (지역사회)건강교육 계획」이 17.31%,「131. 재난간호」가 16.92%,「128. 성 건강증진 간호」가 16.54%,「130. 산업과 환경 간호」가 14.62%,「129. 문화간호」가 14.23% 순으로 높게 나타났다.

삭제에 대한 요구도는「129. 문화간호」가 14.62%,「125. (지역사회)건강교육 계획」이 11.15%,「130. 산업과 환경 간호」가 11.15%,「131. 재난간호」가 9.62%,「128. 성 건강증진 간호」가 8.46%,「132. 치료계획을 결정하기 위해 가족 역동성(family dynamics) (가족구조, 결속, 의사소통, 경계, 대처기전) 사정」이 7.31%,「133. 가정환경에서 대상자를 관리할 수 있는 역량(장비, 지역사회 자원) 평가」가

7.31%,「126. 건강증진 및 유지관리에 대한 정보(예방접종 등)제공」이 6.15%,「134. 건강위험요인 사정과 교육」이 4.62%,「127. 고위험 건강행위의 예방 및 치료에 대한 정보(금연, 안전한 성 행위, 바늘교환)제공」이 4.62% 순으로 나타났다.

<표 Ⅳ-18> 건강유지 및 증진

번호	항목	유지<br>n (%)	분리<br>n (%)	통합<br>n (%)	삭제<br>n (%)	결측<br>n (%)
125	(지역사회)건강교육 계획	116<br>(44.62)	67<br>(25.77)	45<br>(17.31)	29<br>(11.15)	3<br>(1.15)
126	건강증진 및 유지관리에 대한 정보(예방접종 등)제공	136<br>(52.31)	55<br>(21.15)	51<br>(19.62)	16<br>(6.15)	2<br>(0.77)
127	고위험 건강행위의 예방 및 치료에 대한 정보(금연, 안전한 성 행위, 바늘교환)제공	137<br>(52.69)	60<br>(23.08)	48<br>(18.46)	12<br>(4.62)	3<br>(1.15)
128	성 건강증진 간호	114<br>(43.85)	77<br>(29.62)	43<br>(16.54)	22<br>(8.46)	4<br>(1.54)
129	문화간호	99<br>(38.08)	83<br>(31.92)	37<br>(14.23)	38<br>(14.62)	3<br>(1.15)
130	산업과 환경 간호	111<br>(42.69)	79<br>(30.38)	38<br>(14.62)	29<br>(11.15)	3<br>(1.15)
131	재난간호	109<br>(41.92)	79<br>(30.38)	44<br>(16.92)	25<br>(9.62)	3<br>(1.15)
132	치료계획을 결정하기 위해 가족 역동성(family dynamics) (가족구조, 결속, 의사소통, 경계, 대처기전) 사정	116<br>(44.62)	73<br>(28.08)	49<br>(18.85)	19<br>(7.31)	3<br>(1.15)
133	가정환경에서 대상자를 관리할 수 있는 역량(장비, 지역사회 자원) 평가	109<br>(41.92)	83<br>(31.92)	46<br>(17.69)	19<br>(7.31)	3<br>(1.15)
134	건강위험요인 사정과 교육	136<br>(52.31)	57<br>(21.92)	51<br>(19.62)	12<br>(4.62)	4<br>(1.54)

8개 직무영역별 134개 신규간호사 직무항목의 개선 요구도에 대한 자료분석 결과, 간호관리와 전문성 향상 영역의 22개 직무항목에서 항목별로 삭제에 응답한 응답율은「9. 간호사업 평가 관련 업무수행(도구개발, 자료조사, 분석, 비교 및 사업개선)」23.08%,「8. 질 향상(QI) 활동에 참여」16.54%,「16. 지역사회 간호사업의 법적 기준 및 지침에 따른 활동 참여」13.46%,「21. 취약가족에게 필요한 지역사회 자원활용」12.31%,「17. 사례관리활동 참여」10.77% 순이었다. 건강증진 및 유지 영역의 10개 직무항목에서 항목별로 삭제에 응답한 응답율은「129. 문화간호」14.62%,「130. 산업과 환경 간호」11.15%,「125. (지역사회)건강교육 계획」11.15%,「131. 재난간호」9.62%,「128. 성 건강 증진 간호」8.46% 순이었다. 안전과 감염관리 영역의 3개 직무항목에서 항목별로 삭제에 응답한 응답률은 1.15% 이하, 위험요인 사정 영역의 28개 직무항목에서 항목별로 삭제에 응답한 응답률은 2.69% 이하, 기본간호 영역의 19

개 직무항목에서 항목별로 삭제에 응답한 응답률은 3.85% 이하, 생리적 통합유지 영역의 19개 직무항목에서 항목별로 삭제에 응답한 응답률은 4.23%이하, 약물 및 비경구 요법 영역의 7개 직무항목에서 항목별로 삭제에 응답한 응답률은 0.77% 이하, 심리사회적 통합유지 영역의 9개 직무항목에서 항목별로 삭제에 응답한 응답률은 2.31% 이하이었다.

이상에서 살펴본 바와 같이 출제모형의 간호사 직무항목에 대한 타당성과 적절성 평가를 위해 신규간호사 직무항목의 중요도와 수행도 IPA 분석에서는 간호관리와 전문성 향상 영역의 「8. 질 향상(QI) 활동에 참여」, 「17. 사례관리활동 참여」, 「18. 업무를 조직화하여 일을 효율적으로 관리」, 「20. 간호대상에 적합한 다양한 자원 및 매체선택」 등 4개 직무항목, 건강증진 및 유지 영역의 「125. (지역사회)건강교육 계획」, 「129. 문화간호」, 「130. 산업과 환경 간호」, 「131. 재난간호」, 「132. 치료계획을 결정하기 위해 가족 역동성(family dynamics) (가족구조, 결속, 의사소통, 경계, 대처기전) 사정」, 「134. 건강위험요인 사정과 교육」 등 6개 직무항목의 중요도는 3점 이상이었으나 수행도는 3점 미만으로 1사분위에 위치하였다. 그리고 간호관리와 전문성 향상 영역의 「9. 간호사업 평가 관련 업무수행(도구개발, 자료조사, 분석, 비교 및 사업개선)」, 「21. 취약가족에게 필요한 지역사회 자원활용」 등 2개 직무항목은 중요도와 수행도 모두 3점 미만으로 3사분위에 위치하였다.

신규간호사 직무항목의 중요도 및 수행도 IPA 분석결과 와 개선 요구도 분석 결과를 비교해보면, 「8. 질 향상(QI) 활동에 참여」, 「9. 간호사업 평가 관련 업무수행(도구개발, 자료조사, 분석, 비교 및 사업개선)」, 「17. 사례관리활동 참여」, 「21. 취약가족에게 필요한 지역사회 자원활용」 등 간호관리와 전문성 향상 영역의 4개 직무항목, 「125. (지역사회)건강교육 계획」, 「129. 문화간호」,「130. 산업과 환경 간호」, 「131. 재난간호」 등 건강유지 및 증진 영역의 4개 직무항목은 중요도와 수행도 IPA 분석결과 1사분위, 3사분위에 위치하는 직무항목이면서 개선요구도 분석에서 삭제에 응답한 응답률 상위 5위 범주에도 포함되는 직무항목 이었다. 따라서 IPA 분석결과 1사분위, 3사분위에 위치하는 12개 직무항목은 개선 요구도에서 삭제에 응답한 응답율로 분석한 상위 5위 범주의 직무항목과 8개 항목이 중복되었다.

이와 같은 설문조사 분석 결과에 기반하여 임상 및 지역사회 간호실무 현장에 근무하는 전문가를 대상으로 12개 직무항목(8,9,17,18,20,21,125,129,130,131,132,134)의 타당성과 적절성을 확인하기 위해 인터뷰를 하였고, 134개 직무항목에 대한 내용타당도 조사를 실시하였다.

나. 출제모형의 간호사 직무항목에 대한 전문가 인터뷰 및 타당도 조사

1) 전문가 인터뷰

134개 신규간호사 직무항목의 시의성과 타당성, 적절성 평가를 위해 임상 및 지역사회 간호실무 현장에서 근무하는 간호사 260명을 대상으로 134개 신규간호사 직무항목에 대한 설문조사를 실시하여 직무 중요도 및 수행도 3점을 기준으로 IPA 분석결과, 134개의 직무항목 중에서 122개 항목은 중요도와 수행도 모두 3점 이상이었고, 「8. 질 향상(QI) 활동에 참여」,「17. 사례관리활동 참여」,「18. 업무를 조직화하여 일을 효율적으로 관리」,「20. 간호대상에 적합한 다양한 자원 및 매체선택」,「125. (지역사회)건강교육 계획」, 129. 문화간호」,「130. 산업과 환경 간호」,「131. 재난간호」,「132. 치료계획을 결정하기 위해 가족 역동성(family dynamics) (가족구조, 결속, 의사소통, 경계, 대처기전) 사정」,「134. 건강위험요인 사정과 교육」 등 10개 직무항목은 중요도는 3점 이상이었으나 수행도가 3점 미만이었지만, 거의 3점에 근접하였다. 「9. 간호사업 평가 관련 업무수행(도구개발, 자료조사, 분석, 비교 및 사업개선)」,「21. 취약가족에게 필요한 지역사회 자원활용」은 등 2개 직무항목은 중요도와 수행도 모두 3점 미만이었으나 거의 3점에 근접하였다.

134개 신규간호사 직무항목에 대한 개선 요구도 분석결과, 간호관리와 전문성 향상 영역에서 삭제에 응답한 직무항목별 응답율은 「9. 간호사업 평가 관련 업무수행(도구개발, 자료조사, 분석, 비교 및 사업개선)」, 「8. 질 향상(QI) 활동에 참여」, 「16. 지역사회 간호사업의 법적 기준 및 지침에 따른 활동 참여」, 「21. 취약가족에게 필요한 지역사회 자원활용」, 「17. 사례관리활동 참여」 순이었다. 건강증진 및 유지 영역에서 삭제에 응답한 직무항목별 응답율은 「129. 문화간호」,「130. 산업과 환경 간호」, 「125. (지역사회)건강교육 계획」, 「131. 재난간호」, 「128. 성 건강 증진 간호」 순이었다.

이와 같은 분석 결과에 대해 임상 및 지역사회 간호실무현장 전문가를 대상으로 전화 인터뷰를 하였다. 총 7인의 전문가가 참여하였으며, 소속 기관은 학교 보건교사 1인(14.3%), 상급종합병원 간호사 6인(85.7%) 이었다. 근무기간은 7년 이상 6인(85.7%), 보건교사 16년, 현재 근무하고 있는 부서는 내·외과계 4인(57.1%), 소아청소년과, 정신과, 학교가 각 1인(14.3%)이었다.

신규간호사 12개 직무항목(8,9,17,18,20,21,125,129,130,131,132,134)에 대해 임상 및 지역사회 간호 실무현장 전문가 집단을 대상으로 전화 인터뷰를 실시한 결과, 대부분 '모든 간호사의 책임과 의무(8.17.125,134)', '확대되고 있는 간호사의 역할에서

중요한 역할이 될 것(20,129,130,132)', '임상 및 지역사회 간호사에게 필요한 내용(21,132)', '간호과정 적용과 유사한 과정(9)', '이직을 감소시킬 수 있는 역량(18) 등의 긍정적 답변이었다[부록 2]. 따라서 본 연구진은 전문가 인터뷰 결과와 더불어 변화하는 보건의료환경과 간호사의 역할 확대 측면에서 고려해 볼 때 12개 직무항목은 타당하면서 적절하다고 평가하였다.

2) 내용타당도 조사

134개 직무항목의 타당도는 4점 척도로 평가하고 수정의견이 있을 경우에는 기술하도록 하였다. 평가 결과 3, 4점(타당함, 매우 타당함)인 항목은 1점, 1, 2점(매우 타당하지 않음, 타당하지 않음)인 항목은 0점으로 계산하여 산출한 문항 내용타당도 지수(Item-level Content Validity Index, I-CVI)에서 0.8 미만인 항목은 없었다.

가) 간호관리와 전문성 향상

간호관리와 전문성 향상 영역의 직무항목 내용타당도 지수는 다음과 같다<표 Ⅳ-19>. '7. 간호단위 물품교환체계에 따른 물품관리' 항목에 대해 신규간호사의 경우 관리자처럼 포괄적인 물품관리가 명확하지 않으므로 '환자처치관련 물품 확인', '처치 등 관리영역 일지'로 수정의견을 제시하였다.

<표 Ⅳ-19> 간호관리와 전문성 향상

번호	항목	Mean±SD	I-CVI
1	인수인계시행	4.00±0.000	1.00
2	법적 실무범위 내에서 간호 수행	4.00±0.000	1.00
3	기록시 표준화된 약어사용	4.00±0.000	1.00
4	지침에 따라 간호기록	4.00±0.000	1.00
5	입원, 전동, 퇴원	3.86±0.378	1.00
6	장비를 적절하고 안전하게 사용	3.71±0.488	1.00
7	간호단위 물품교환체계에 따른 물품관리	3.43±0.787	0.86
8	질 향상(QI) 활동에 참여	3.29±0.488	1.00
9	간호사업 평가 관련 업무수행(도구개발, 자료조사, 분석, 비교 및 사업개선)	3.29±0.488	1.00

번호	항목	Mean±SD	I-CVI
10	간호전문직 윤리 준수와 역할	4.00±0.000	1.00
11	대상자의 개인정보 및 사생활 보호	4.00±0.000	1.00
12	대상자에게 치료 및 절차에 따라 적절한 설명을 하고 동의를 획득하였는지 확인	4.00±0.000	1.00
13	환자의 권리와 책임에 관하여 대상자에게 교육 제공	3.86±0.378	1.00
14	억제대 사용 시 법적 및 윤리적 간호	3.86±0.378	1.00
15	일차의료기반의 보건간호	3.57±0.535	1.00
16	지역사회 간호사업의 법적 기준 및 지침에 따른 활동 참여	3.57±0.535	1.00
17	사례관리활동 참여	3.43±0.535	1.00
18	업무를 조직화하여 일을 효율적으로 관리	3.43±0.535	1.00
19	환자분류체계 관련 정보수집 및 활용	3.71±0.488	1.00
20	간호대상에 적합한 다양한 자원 및 매체선택	3.57±0.535	1.00
21	취약가족에게 필요한 지역사회 자원활용	3.29±0.488	1.00
22	전문직간 협업	3.71±0.488	1.00

나) 안전과 감염관리

안전과 감염관리 영역의 직무항목 내용타당도 지수는 다음과 같다<표 Ⅳ-20>.

<표Ⅳ-20> 안전과 감염관리

번호	항목	Mean±SD	I-CVI
23	안전한 환경 제공	4.00±0.000	1.00
24	감염관리	4.00±0.000	1.00
25	위험물질과 유해물질 관리	3.86±0.378	1.00

다) 위험요인 사정

위험요인 사정 영역의 직무항목 내용타당도 지수는 다음과 같다<표 Ⅳ-21>.

<표 Ⅳ-21> 위험요인 사정

번호	항목	Mean±SD	I-CVI
26	간호계획, 진료지침 수행	4.00±0.000	1.00
27	대상자의 건강문제에 대해 우선순위 결정	4.00±0.000	1.00
28	활력징후 사정	4.00±0.000	1.00
29	신생아 간호	3.86±0.378	1.00
30	영아 간호	3.86±0.378	1.00
31	유아 간호	3.86±0.378	1.00
32	학령전기 간호	3.86±0.378	1.00
33	학령기 간호	3.86±0.378	1.00
34	청소년 간호	3.86±0.378	1.00
35	폐경기 여성 간호	3.71±0.488	1.00
36	노인간호	3.86±0.378	1.00
37	성 건강 간호	3.71±0.488	1.00
38	건강력 사정	4.00±0.000	1.00
39	신체검진 수행 및 결과 해석	4.00±0.000	1.00
40	생식기 건강사정	3.71±0.488	1.00
41	태아건강사정 및 간호	3.71±0.488	1.00
42	신생아 건강사정 및 간호	3.86±0.378	1.00
43	고위험신생아 건강사정 및 간호	3.71±0.488	1.00
44	심폐소생술 간호	4.00±0.000	1.00
45	응급간호	4.00±0.000	1.00
46	진단검사 간호	3.86±0.378	1.00
47	수술간호	4.00±0.000	1.00
48	산전간호와 교육	3.86±0.378	1.00
49	분만 중 간호와 교육	3.86±0.378	1.00
50	산후관리와 교육	3.86±0.378	1.00
51	고위험 임부간호	3.71±0.488	1.00
52	고위험 산부간호	3.71±0.488	1.00
53	고위험 산모간호	3.71±0.488	1.00

라) 기본간호

기본간호 영역의 직무항목 내용타당도 지수는 다음과 같다<표 Ⅳ-22>.

<표 Ⅳ-22> 기본간호

번호	항목	Mean±SD	I-CVI
54	체온유지간호	4.00±0.000	1.00
55	세척(irrigation)(방광, 귀, 눈) 수행	3.71±0.488	1.00
56	개인위생간호	4.00±0.000	1.00
57	섭취량 및 배설량 사정과 간호	4.00±0.000	1.00
58	영양사정 및 관리	4.00±0.000	1.00
59	질환별 영양문제의 사정 및 관리	4.00±0.000	1.00
60	섭취장애 대상자 사정 및 간호	3.86±0.378	1.00
61	배뇨장애 대상자 사정 및 간호	3.86±0.378	1.00
62	투석대상자 관리	3.71±0.488	1.00
63	요루 및 장루관리	3.71±0.488	1.00
64	기관절개부 관리	3.86±0.378	1.00
65	배변관리	3.71±0.488	1.00
66	수면과 휴식 간호	3.86±0.378	1.00
67	피부통합성 사정 및 간호	3.86±0.378	1.00
68	이동간호	3.86±0.378	1.00
69	활동과 자기돌봄 장애 사정 및 간호	3.71±0.488	1.00
70	외과적 장치 관리	3.57±0.535	1.00
71	척추손상 및 신경질환 대상자의 간호	3.86±0.378	1.00
72	관절대치술 환자 간호	3.86±0.378	1.00

마) 생리적 통합유지

생리적 통합유지 영역의 직무항목 내용타당도 지수는 다음과 같다<표 Ⅳ-23>.

<표 Ⅳ-23> 생리적 통합유지

번호	항목	Mean±SD	I-CVI
73	호흡기능장애 대상자 간호	3.86±0.378	1.00
74	호흡증진 중재	3.86±0.378	1.00
75	호흡보조장치 관리	3.71±0.488	1.00
76	중심정맥관 관리	3.86±0.378	1.00
77	태아질식 증상과 징후 사정 및 간호	3.71±0.488	1.00
78	고위험 신생아 보육기 적용간호	3.71±0.488	1.00
79	호흡재활관리	3.71±0.488	1.00

번호	항목	Mean±SD	I-CVI
80	심전도 관리	3.86±0.378	1.00
81	순환 보조장치 관리	3.71±0.488	1.00
82	동맥관 관리	3.71±0.488	1.00
83	조직 관류 장애 대상자 사정 및 간호	3.86±0.378	1.00
84	체액 전해질 불균형 사정 및 간호	4.00±0.000	1.00
85	활동 지속성 장애 대상자 사정 및 간호	4.00±0.000	1.00
86	정맥 순환증진 장치 간호	3.86±0.378	1.00
87	혈액 기능장애 대상자 간호	3.71±0.488	1.00
88	순환 기능장애 대상자 간호	3.71±0.488	1.00
89	심장 수술 후 간호	3.86±0.378	1.00
90	소화 기능장애 대상자 간호	3.86±0.378	1.00
91	배뇨장애를 가진 대상자 간호	3.86±0.378	1.00
92	당질 대사장애 대상자 간호	3.86±0.378	1.00
93	당뇨병 합병증 예방을 위한 간호	3.86±0.378	1.00
94	내분비계 장애 대상자 간호	3.86±0.378	1.00
95	생식기 질환/생식기 건강문제를 가진 대상자 간호	3.71±0.488	1.00
96	면역손상 대상자 사정 및 간호	3.71±0.488	1.00
97	감각기능 장애를 가진 대상자 사정 및 간호	3.71±0.488	1.00
98	두개내압 상승 환자의 간호	3.86±0.378	1.00
99	신경계 질환별 간호중재	3.86±0.378	1.00
100	운동기능 장애 간호중재	3.86±0.378	1.00
101	상처간호 수행 및 드레싱교환	3.71±0.488	1.00
102	화상환자 간호중재	3.71±0.488	1.00
103	피부질환 대상자의 간호중재	3.71±0.488	1.00
104	배액장치 관리	3.86±0.378	1.00
105	계통별 신생물 질환 대상자 간호	3.57±0.535	1.00
106	통증간호	4.00±0.000	1.00
107	재활간호서비스	3.71±0.488	1.00
108	(법정) 감염질환자의 전파예방 및 간호	3.86±0.378	1.00

바) 약물 및 비경구요법

약물 및 비경구요법 영역의 직무항목 내용타당도 지수는 다음과 같다<표 Ⅳ-24>.

<표 Ⅳ-24> 약물 및 비경구요법

번호	항목	Mean±SD	I-CVI
109	투약의 적절성과 정확성	4.00±0.000	1.00
110	약물투여에 필요한 계산 시행	4.00±0.000	1.00
111	정맥주입 장치 관리	4.00±0.000	1.00
112	약품관리	4.00±0.000	1.00
113	말초정맥관 삽입, 유지, 제거	4.00±0.000	1.00
114	대상자에게 약물에 대해 교육	3.86±0.378	1.00
115	고위험 약품관리(마약관리)	4.00±0.000	1.00

사) 심리사회적 통합 유지

심리사회적 통합 유지 영역의 직무항목 내용타당도 지수는 다음과 같다<표 Ⅳ-25>.

<표 Ⅳ-25> 심리사회적 통합 유지

번호	항목	Mean±SD	I-CVI
116	이상행동 사정 및 간호	3.86±0.378	1.00
117	폭력의 잠재성 평가 및 예방	3.86±0.378	1.00
118	학대 또는 방임 대상자를 확인하고 적절하게 중재	3.86±0.378	1.00
119	중독 사정 및 간호	3.86±0.378	1.00
120	정신사회건강문제 간호 및 교육	3.86±0.378	1.00
121	정신질환자 간호	3.86±0.378	1.00
122	아동 정신질환자 간호	3.86±0.378	1.00
123	임종간호와 교육	3.86±0.378	1.00
124	치료적 의사소통 기법	4.00±0.000	1.00

아) 건강유지 및 증진

건강유지 및 증진 영역의 직무항목 내용타당도 지수는 다음과 같다<표 Ⅳ-26>.

<표 Ⅳ-26> 건강유지 및 증진

번호	항목	Mean±SD	I-CVI
125	(지역사회)건강교육 계획	3.86±0.378	1.00
126	건강증진 및 유지관리에 대한 정보(예방접종 등)제공	3.86±0.378	1.00
127	고위험 건강행위의 예방 및 치료에 대한 정보(금연, 안전한 성 행위, 바늘교환)제공	3.86±0.378	1.00
128	성 건강증진 간호	3.71±0.488	1.00
129	문화간호	3.71±0.488	1.00
130	산업과 환경 간호	3.71±0.488	1.00
131	재난간호	3.71±0.488	1.00
132	치료계획을 결정하기 위해 가족 역동성(family dynamics) (가족구조, 결속, 의사소통, 경계, 대처기전) 사정	3.71±0.488	1.00
133	가정환경에서 대상자를 관리할 수 있는 역량(장비, 지역사회 자원) 평가	3.57±0.535	1.00
134	건강위험요인 사정과 교육	4.00±0.000	1.00

2012년부터 국가시험 출제기준 개발을 위해 단계별로 순차적으로 진행된 연구를 통하여 도출된 신규간호사 직무기반 통합형 국가시험 출제모형의 주개념은 직무영역(대분류), 직무항목(중분류), 표준 학습목표, 지식항목(소분류)이다. 신규간호사 직무항목은 직무영역, 표준학습목표, 지식항목과 연계되어 있으므로, 우선적으로 출제모형의 주개념인 직무항목의 시의성을 확인하기 위하여 타당성과 적절성 평가를 실시하였다. 설문조사, 전문가 인터뷰 및 내용타당도 조사에서 직무항목의 타당성과 적절성이 확인되어, 연구진 합의하에 직무항목의 용어만 일관성을 유지할 수 있도록 간결하게 정리하고 134개 직무항목은 출제모형에 반영하기로 하였다.

다. 간호사 국가시험 출제모형에 대한 유관 단체 전문가 의견조사

문헌고찰, 설문조사 결과, 임상실무 전문가 인터뷰, 타당도 조사결과에 근거하여 연구진의 합의에 의해 국내 신규간호사 직무기반 통합형 간호사 국가시험 출제모형을 확인하였다. 확인된 출제모형의 8개 신규간호사 직무영역과 직무영역에 연계된 134개 직무항목, 직무항목과 연계하여 간호학 전공 교과목의 표준학습목표에서 도출된 481개 범주의 지식항목에 대해 시험문제로의 개발가능성과 평가실행 가능성에 대한 타당

성과 적절성을 조사하기 위해 유관 단체를 대상으로 의견조사를 실시하였다. 7개 회원학회를 대상으로 공문을 발송하여 1차 의견조사(2021. 03.05 ~ 2021.03.19.)를 실시하였다. 타 학회에서 제시한 의견을 공유하면서 학회별로 제시한 의견을 수정 보완할 수 있도록 공문을 발송하여 481개 범주의 지식항목에 대해 시험문제로의 개발가능성과 평가실행 가능성에 대한 2차 의견조사(2021.04.30 ~ 2021.05.14.)를 실시하였다. 7개 학회에서 제시한 의견은 한국간호과학회, 한국간호대학(과)장협의회, 한국간호전문대학(부)장협의회, 병원간호사회 등 유관 단체에 검토를 요청하면서 이에 대한 의견조사(2021. 05.21 ~ 06.04.)를 실시하였다. 직무기반 통합형 간호사 국가시험 출제모형에 대해 타당성과 적절성을 검토하면서 시험문제로의 개발가능성과 평가실행 가능성에 대한 7개 학회의 1차 의견을 수정 보완하여 2차에 제시한 7개 회원학회의 의견조사 결과, 출제모형의 8개 신규간호사 직무영역과 직무영역에 연계된 134개 직무항목, 직무항목과 연계하여 간호학 전공 교과목의 표준학습목표에서 도출된 481개 범주의 지식항목에 대해 시험문제로의 개발가능성과 평가실행 가능성은 확인되었다.

간호관리학은 134개 직무항목에서는 23개(17.2%), 481개의 지식항목에서는 75개 항목(15.6%)이 시험문제로의 개발가능성과 평가실행 가능성이 있는 것으로 표기하였다. 기본간호학은 134개 직무항목에서는 24개(17.9%), 481개의 지식항목에서는 87개 항목(18.1%), 모성간호학은 134개 직무항목에서는 29개(21.6%), 481개의 지식항목에서는 72개 항목(15.0%), 성인간호학은 134개 직무항목에서는 59개(44.0%), 481개의 지식항목에서는 138개 항목(28.7%), 아동간호학은 134개 직무항목에서는 64개(47.8%), 481개의 지식항목에서는 205개 항목(42.6%), 정신간호학은 134개 직무항목에서는 38개(28.4%), 481개의 지식항목에서는 93개 항목(19.3%), 지역사회간호학은 134개 직무항목에서는 39개(29.1%), 481개의 지식항목에서는 127개 항목(26.4%)이 시험문제로의 개발가능성과 평가실행 가능성이 있는 것으로 표기하였다[부록3]. 134개 직무항목에 대한 분포는 아동간호학, 성인간호학.지역사회간호학, 정신간호학, 모성간호학, 기본간호학, 간호관리학 순이었고, 481범주의 지식항목에 대한 분포는 아동간호학, 성인간호학, 지역사회간호학, 정신간호학, 기본간호학, 간호관리학, 모성간호학 순이었다. 8개 직무영역, 134개 신규간호사 직무항목, 481개 범주의 지식항목에 대한 7개 학회의 2차 의견조사 결과를 요약, 정리한 결과는 <표IV-29>와 같다.

481개 범주의 지식항목에 대한 국가시험 교과목 배정을 선행연구(탁영란 외,2019)와 7개 회원학회 의견을 비교해 보면, 탁영란 외(2019) 연구에서 지식항목에 대한 교과목 배정은 간호관리학은 481개의 지식항목에서 41개 항목(8.5%), 기본간호학은 75개 항목(15.6%), 모성간호학은 47개 항목(9.8%), 성인간호학은 111개 항목(23.1%), 아동간호학은 72개 항목(15.0%), 정신간호학은 45개 항목(9.4%), 지역사회간호학은

62개 항목(12.9%)이었다. 탁영란 외(2019) 연구와 7개 학회의 지식항목 교과목 배정에서 가장 많은 차이가 있는 교과목은 아동간호학으로 관련 학회의 배정은 250개 항목(42.6%), 선행연구의 배정은 72개 항목(15.0%)으로 배정 분포의 차이가 27.6%이었다. 지식항목 배정 분포의 차이는 지역사회간호학13.5%, 정신간호학 9.9%, 간호관리학 7.1%, 성인간호학 5.6%, 모성간호학 5.2%, 기본간호학 2.5% 순이었다<표IV-27>.

481개 범주의 지식항목 중에서 243개(50.5%) 범주는 1개 학회, 167개(34.7%) 범주는 2개 학회에서, 67개(13.9%) 범주는 3개 이상 학회의 관련 교과목에서 국가시험 문제로의 개발가능성과 평가실행 가능성이 있다고 하였다. 기본간호 영역의「세척(방광, 귀, 눈) 수행」지식항목의「세척(방광, 귀, 눈) 수행」지식범주, 생리적 통합유지 영역의「(법정) 감염질환자의 전파예방 및 간호」지식항목의「대상 기관의 구조 및 기능, 건강사정, 전파경로별 격리주의 의료폐기물 관리」지식범주, 약물 및 비경구 요법 영역의「약물관리」지식항목의「지침에 따라 규제 약물을 관리(증인, 잔량폐기), 안전하고 통제된 환경에서 의약품 취급관리」지식범주와「투약오류 보고 및 예방」지식범주 등 4개(0.9%) 범주는 관련 교과목에서 국가시험 문제로의 개발가능성과 평가실행 가능성이 있다고 표기한 학회가 없었다[부록3]. 국가시험 교과목에서 시험문제로의 개발가능성과 평가실행 가능성이 있는 것으로 표기하지 않은 4개의 지식범주는 환자안전과 감염관리 차원에서 임상 및 지역사회 간호실무 현장에서 간호사의 주요한 직무이다.

7개 회원학회에서 선정한 지식항목의 수를 국가시험 문제 출제수와 비교해보면, 선정한 지식항목 수가 출제 문제수의 간호관리학 2.14배, 기본간호학 2.90배, 모성 간호학 2.60, 성인간호학 1.97, 아동간호학 5.86, 정신간호학 2.66, 지역사회간호학은 3.63배수 이었다.

이와 같은 의견조사 결과에 대해 7개 학회, 협의회, 병원간호사회 등의 유관 단체에서는 추후 교육전문가, 임상 및 지역사회 간호실무 현장 전문가로 구성된 TFT(Task Force Team)에서 교과목별로 직무영역 및 직무항목과 연계된 지식항목의 분포, 2개 이상 및 3개 이상 학회에서 관련 교과목에서 국가시험 문제로의 개발가능성과 평가실행 가능성이 있다고 표기한 지식항목에 대한 충분한 논의가 필요하다는 의견을 제시하였다.

추후 TFT에서 국가시험 출제기준 개발을 위한 재검토 과정에서는 학회별로 교과목의 학습목표와 연계된 지식항목의 범주를 명확히 정리하면서 134개 직무항목, 481개 범주의 지식항목에서 중복되는 항목과 범주의 통합 및 범주의 구성 항목의 적절성에 대한 검토도 동시에 이루어져야 한다.

7개 회원학회, 협의회 및 병원간호사회 등 유관 단체에서 제시한 의견에 따라 본

연구에서는 134개 직무항목의 용어가 일관성과 명료성을 유지할 수 있도록 교육전문가 6인의 자문과 검토과정을 거쳐서 간결하게 정리하여 출제모형에 반영하였다<표 Ⅳ-28>.

본 연구는 간호사 국가시험 출제기준 개발을 위한 기초연구이므로, 추후 출제기준 개발을 위해 출제모형에 대한 재검토 과정에서 중점적으로 논의되어야 할 사항에 대한 기초자료를 제공하였다. 출제모형에서 중복되는 직무항목 및 지식항목, 통합되어야 할 지식항목의 범주, 범주의 구성 항목의 적절성에 대해 7개 회원학회별로 제출한 의견을 정리하여 제시함으로써 간호사 국가시험 출제모형에 근거한 출제기준 개발에 반영될 수 있도록 하였다[부록3], [부록4], [부록5], [부록6], [부록7].

<표 Ⅳ-27> 지식항목의 교과목 배정에 대한 선행연구(탁영란 외, 2019와)와 7개 회원학회 의견 비교

(단위: 개)

대분류	선행연구(탁영란 외, 2019)									7개 회원학회 2차 의견								
	지식항목	관리	기본	모성	성인	아동	정신	지역	계	지식항목	관리	기본	모성	성인	아동	정신	지역	계
Ⅰ. 간호관리와 전문성 향상	**79**	31	2	-	-	-	4	22	59	**79**	58	-	3	-	4	9	53	127
Ⅱ. 안전과 감염관리	**25**	10	8	-	2	-	-	-	20	**25**	17	16	-	2	2	12	13	62
Ⅲ. 위험요인 사정	**93**	-	8	32	12	41	-	-	93	**93**	-	3	48	20	48	13	6	138
Ⅳ. 기본간호	**60**	-	31	-	26	3	-	-	60	**60**	-	42	-	32	42	8	-	124
Ⅴ. 생리적 통합 유지	**130**	-	13	12	68	28	1	6	128	**130**	-	16	15	79	85	4	9	208
Ⅵ. 약물 및 비경구 요법	**14**	-	13	-	-	-	-	-	13	**14**	-	10	1	1	5	3	8	28
Ⅶ. 심리적 통합 유지	**43**	-	-	-	3	-	40	-	43	**43**	-	-	2	4	9	40	2	57
Ⅷ. 건강증진 및 유지	**37**	-	-	3	-	-	-	34	37	**37**	-	-	3	-	10	4	36	53
합계	481	41	75	47	111	72	45	62	453	481	75	87	72	138	205	93	127	797

* 선행연구(탁영란외, 2019) 지식항목 배정에서 보건의약관계법규 교과목 배정은 제외하고 제시함

<표 Ⅳ-28> 134개 신규간호사 직무항목 용어정리

134개 직무항목			용어 수정
Ⅰ 간호관리와 전문성향상	1	인수인계시행	인수인계
	2	법적 직무범위 내에서 간호 수행	간호표준과 실무
	3	기록 시 표준화된 약어 사용	표준화된 약어 사용
	4	지침에 따라 간호기록	간호정보와 기록
	5	입원, 전동, 퇴원	입원, 전동, 퇴원 간호
	6	장비를 적절하고 안전하게 사용	물품 및 약품관리
	7	간호단위 물품교환체계에 따른 물품관리	간호단위 물품교환체계에 따른 물품관리
	8	질 향상(QI) 활동에 참여	질 향상(QI) 활동
	9	간호사업 평가 관련 업무수행 (도구개발, 자료조사, 분석, 비교,사업개선)	간호사업 평가
	10	간호전문직 윤리 준수와 역할	간호전문직 윤리
	11	대상자의 개인정보 및 사생활 보호	대상자의 개인정보 및 사생활 보호
	12	대상자에게 치료 및 절차에 따라 적절한 설명을 하고 동의를 획득하였는지 확인	치료 및 절차 설명/대상자 동의 확인
	13	환자의 권리와 책임에 관하여 대상자에게 교육 제공	환자권리와 책임에 대한 대상자 교육
	14	억제대 사용 시 법적 및 윤리적 간호	억제대 사용 관련 법적 및 윤리적 간호
	15	일차의료기반의 보건간호	일차의료기반의 보건간호
	16	지역사회 간호사업의 법적 기준 및 지침에 따른 활동 참여	지역사회 간호사업
	17	사례관리활동 참여	사례관리 활동
	18	업무를 조직화하여 일을 효율적으로 관리	기획과 조직화
	19	환자분류체계 관련 정보수집 및 활용	환자분류체계
	20	간호대상에 적합한 다양한 자원 및 매체선택	간호서비스 마케팅
	21	취약가족에 필요한 지역사회 자원 활용	취약가족 대상 지역사회 자원 활용
	22	전문직 간 협업	전문직 간 협업
Ⅱ 안전과 감염관리	23	안전한 환경 제공	안전간호
	24	감염관리	감염관리
	25	위험물질과 유해물질 관리	위험물질과 유해물질 관리
Ⅲ 위험요인사정	26	간호계획, 진료지침 수행	간호계획, 진료지침 수행
	27	대상자의 건강문제에 대해 우선순위 결정	건강문제 우선순위 결정
	28	활력징후 사정	활력징후 사정
	29	신생아 간호	신생아 간호
	30	영아 간호	영아 간호
	31	유아 간호	유아 간호
	32	학령전기 간호	학령전기 간호
	33	학령기 간호	학령기 간호
	34	청소년 간호	청소년 간호
	35	폐경기 여성 간호	폐경기 여성 간호
	36	노인 간호	노인 간호
	37	성 건강 간호	성 건강 간호
	38	건강력 사정	건강력 사정
	39	신체검진 수행 및 결과해석	신체검진
	40	생식기 건강사정	생식기 건강사정
	41	태아 건강사정 및 간호	태아 건강사정 및 간호
	42	신생아 건강사정 및 간호	신생아 건강사정 및 간호
	43	고위험신생아 건강사정 및 간호	고위험신생아 건강사정 및 간호
	44	심폐소생술 간호	심폐소생술 간호
	45	응급간호	응급간호

134개 직무항목			용어 수정
	46	진단검사 간호	진단검사 간호
	47	수술 간호	수술 간호
	48	산전간호와 교육	산전간호와 교육
	49	분만중 간호와 교육	분만중 간호와 교육
	50	산후관리와 교육	산후간호와 교육
	51	고위험임부 간호	고위험임부 간호
	52	고위험 산부간호	고위험 산부간호
	53	고위험 산모간호	고위험 산모간호
Ⅳ 기본간호	54	체온유지간호	체온유지간호
	55	세척(irrigation) 수행 (방광, 귀, 눈)	세척(irrigation) 수행
	56	개인위생간호	개인위생간호
	57	섭취 및 배설량 사정과 간호	섭취 및 배설 관련 간호
	58	영양사정 및 관리	영양사정 및 간호
	59	질환별 영양문제의 사정 및 관리	질환별 영양문제 사정 및 간호
	60	섭취장애 대상자 사정 및 간호	섭취장애 대상자 사정 및 간호
	61	배뇨장애 대상자 사정 및 간호	배뇨장애 대상자 사정 및 간호
	62	투석대상자 관리	투석대상자 간호
	63	요루 및 장루 관리	요루 및 장루 대상자 간호
	64	기관절개부 관리	기관절개관 간호
	65	배변관리	배변장애 대상자 간호
	66	수면과 휴식 간호	수면각성장애 대상자 간호
	67	피부통합성 사정 및 간호	피부통합성 사정 및 간호
	68	이동 간호	이동 간호
	69	활동과 자기돌봄장애 사정 및 간호	활동과 운동 사정 및 간호
	70	외과적 장치 관리	외과적 장치 관리
	71	척추손상 및 질환대상자의 간호	척추 손상 및 질환 대상자 간호
	72	관절대치술 환자 간호	관절대치술 대상자 간호
Ⅴ 생리적통합유지	73	호흡기능장애 대상자 간호	호흡기능장애 대상자 간호
	74	호흡증진중재	호흡증진중재
	75	호흡보조장치 관리	호흡보조장치 관리
	76	중심정맥관 관리	중심정맥관 관리
	77	태아질식 증상과 징후 사정 및 간호	태아질식 증상과 징후 사정 및 간호
	78	고위험신생아 보육기 적용간호	고위험신생아 보육기 적용간호
	79	호흡재활관리	호흡재활관리
	80	심전도관리	심전도관리
	81	순환보조장치관리	순환보조장치관리
	82	동맥관 관리	동맥관 관리
	83	조직관류장애 대상자 사정 및 간호	조직관류장애 대상자 사정 및 간호
	84	체액전해질 불균형 사정 및 간호	체액전해질 불균형 사정 및 간호
	85	활동지속성 장애 대상자 사정 및 간호	활동지속성 장애 대상자 사정 및 간호
	86	정맥순환증진장치 간호	정맥순환증진 간호
	87	혈액기능장애 대상자 간호	혈액기능장애 대상자 간호
	88	순환기능장애 대상자 간호	순환기능장애 대상자 간호
	89	심장수술 후 간호	심장수술 후 간호
	90	소화기능장애 대상자 간호	소화기능장애 대상자 간호

134개 직무항목			용어 수정
	91	배뇨장애를 가진 대상자 간호	배뇨장애 대상자 간호
	92	당질대사장애 대상자 간호	당질대사장애 대상자 간호
	93	당뇨병 합병증 예방을 위한 간호	당뇨병 합병증 예방간호
	94	내분비계장애 대상자 간호	내분비계장애 대상자 간호
	95	생식기 질환/생식기 건강문제를 가진 대상자 간호	생식기 질환/생식기 건강문제 대상자 간호
	96	면역손상 대상자 사정 및 간호	면역손상 대상자 간호
	97	감각기능장애를 가진 대상자 사정 및 간호	감각기능장애 대상자 간호
	98	두개내압 상승 환자의 간호	두개내압 상승 대상자 간호
	99	신경계 질환별 간호중재	신경계 질환별 대상자 간호
	100	운동기능장애 간호중재	운동기능장애 대상자 간호
	101	상처간호 수행 및 드레싱 교환	상처간호
	102	화상환자 간호중재	화상간호
	103	피부질환 대상자의 간호중재	피부질환 대상자 간호
	104	배액장치관리	배액장치관리
	105	계통별 신생물 질환 대상자의 간호	계통별 신생물 질환 대상자 간호
	106	통증간호	통증간호
	107	재활간호서비스	재활간호
	108	(법정) 감염질환자의 전파예방 및 간호	(법정) 감염질환자의 전파예방 및 간호
Ⅵ 약물 및 비경구요법	109	투약의 적절성과 정확성	투약간호
	110	약물 투여에 필요한 계산 시행	약 용량 계산
	111	정맥주입장치 관리	정맥주입장치 관리
	112	약품관리	약품관리
	113	말초정맥관 삽입, 유지, 제거	말초정맥관 관리
	114	대상자에게 약물에 대해 교육	투약관련 대상자 교육
	115	고위험 약품관리(마약관리)	고위험 약품관리(마약관리)
Ⅶ 심리적 통합 유지	116	이상행동 사정 및 간호	이상행동 사정 및 간호
	117	폭력의 잠재성 평가 및 예방	폭력 잠재성 평가 및 예방
	118	학대 또는 방임 대상자를 확인하고 적절하게 중재	학대 또는 방임 확인 및 중재
	119	중독 사정 및 간호	중독 사정 및 간호
	120	정신사회 건강문제 간호 및 교육	정신사회 건강문제 간호 및 교육
	121	정신질환자 간호	정신질환자 간호
	122	아동정신질환자 간호	아동정신질환자 간호
	123	임종간호와 교육	임종간호 및 교육
	124	치료적 의사소통 기법	치료적 의사소통 기법
Ⅷ 건강증진 및 유지	125	(지역사회)건강교육계획	(지역사회)건강교육계획
	126	건강증진 및 유지관리에 대한 정보(예방접종 등) 제공	건강증진 및 유지관련 정보제공
	127	고위험 건강 행위의 예방 및 치료에 대한 정보 제공 (금연, 안전한 성행위, 바늘교환)	고위험 건강 행위 예방 및 치료관련 정보제공
	128	성건강 증진간호	성건강 간호
	129	문화간호	문화간호
	130	산업과 환경 간호	산업간호 및 환경 간호
	131	재난간호	재난간호
	132	치료계획을 결정하기 위해 가족 역동성(가족구조, 결속, 의사소통, 경계, 대처기전) 사정	치료계획 결정을 위한 가족 역동성 사정
	133	가정환경에서 대상자를 관리할 수 있는 역량 (장비, 지역사회 자원) 평가	지역사회 자원평가
	134	건강위험요인 사정과 교육	건강위험요인 사정 및 교육

라. 직무기반 통합형 간호사 국가시험 출제모형 제시

국가시험 관련 국내외 문헌고찰, 출제모형의 직무항목에 대한 타당성과 적절성 평가를 위한 설문조사 결과, 임상실무 전문가 인터뷰 및 내용타당도 조사결과, 7개 학회, 협의회 및 병원간호사회 등 유관 단체를 대상으로 실시 한 출제모형의 타당도와 적절성에 대한 의견조사 결과에 기반하여 신규간호사 직무기반 통합형 간호사 국가시험 출제모형을 연구진 합의하에 제시하였다. 간호사 국가시험 출제모형은 [그림 Ⅳ-11]와 같다.

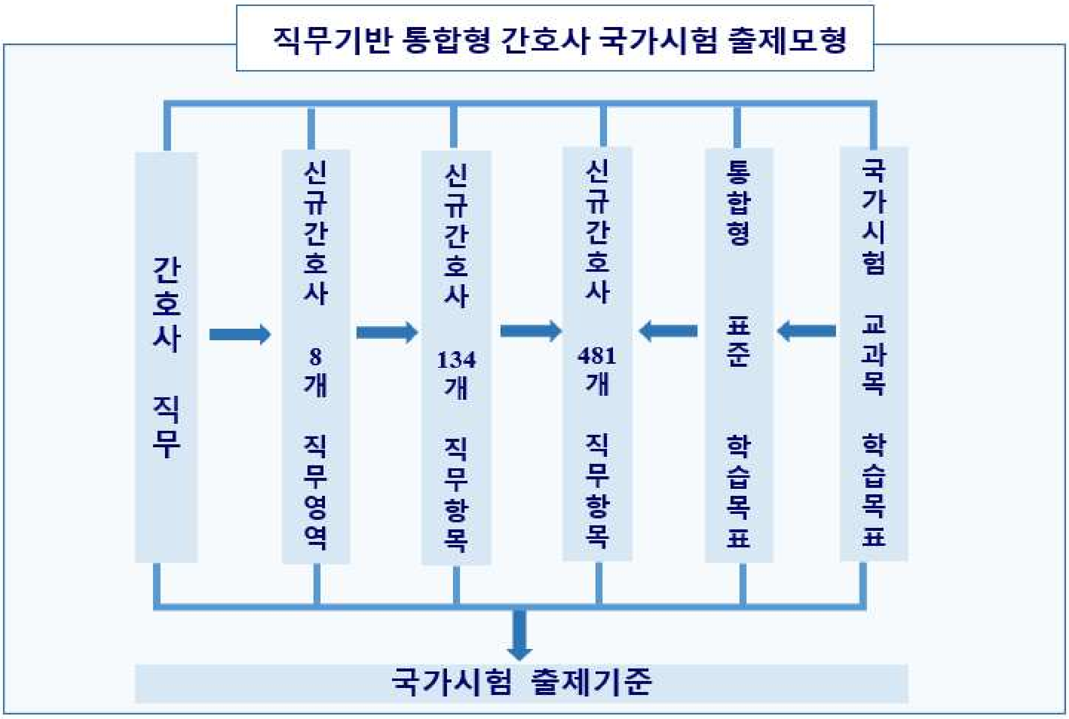

[그림 Ⅳ-11] 간호사 국가시험 출제모형

본 연구에서 제시한 출제모형은 단계별로 순차적으로 진행된 선행연구 결과에 기반한 모형이다. 선행연구에서는 먼저, 간호사 직무를 분석하여 신규간호사의 8개 직무영역을 확인하였다, 그 다음 단계에서 순차적으로 확인한 직무영역을 보다 구체적인 수준의 134개의 직무항목으로 연계하였다(직무기반). 국가시험 교과목 학습목표의 중복성을 해결하면서 직무에 기반한 통합형 표준학습목표를 개발하여(통합형), 신규간호사 직무항목과 연계함으로써 시험 문항으로 개발할 수 있는 481개 범주의 지식항목을 도출하였다. 이와 같은 과정을 통하여 신규간호사 직무기반 통합형 출제모형이 개발되었다.

출제모형은 직무영역(대분류), 직무항목(중분류), 통합형 표준학습목표, 지식항목(소분류) 등의 주개념으로 구성된다. 직무영역은 박인숙 외(2012)의 연구에서 간호사 직무를 분석하여 신규간호사 직무를 영역별로 분류한 간호관리와 전문성 향상, 안전과 감염관리, 잠재적 위험요인 관리, 기본간호, 생리적 통합유지, 약물 및 비경구 요법, 심리사회적 통합유지, 건강증진 및 유지 등 8개 영역이다. 직무항목(중분류)은 송라윤 외(2018년)의 연구에서 신규간호사 직무를 8개 직무영역에 연계하여 분류한 134개의 직무항목이다. 그리고 간호사 국가시험 7개 교과목의 학습목표를 통합하여 통합형 표준 학습목표를 만들어서 직무영역과 연계된 간호사 직무항목에 연계하여 1,303개의 지식항목을 도출하였다. 탁영란 외(2019) 연구에서는 직무영역, 직무항목 및 통합형 표준 학습목표와 연계하여 도출한 1,303개 지식항목을 481개로 범주화하였다. 이와 같이 단계별로 순차적으로 진행된 선행연구에 근거하여 간호사 국가시험 출제기준의 근거가 되는 직무영역(대분류), 직무항목(중분류), 지식항목(소분류)으로 구성된 출제모형을 제시하였다.

8개 직무영역의 간호관리와 전문성 향상 영역에는 22개의 직무항목(16.4%), 안전과 감염관리 영역에는 3개의 직무항목(2.2%), 위험요인 사정 영역에는 28개의 직무항목(20.9%), 기본간호 영역에는 19개의 직무항목(14.2%), 생리적 통합유지 영역에는 36개의 직무항목(26.9%), 약물 및 비경구 요법 영역에는 7개의 직무항목(5.2%), 심리적 통합유지 영역에는 9개의 직무항목(6.7%), 건강증진 및 유지 영역에는 10개의 직무항목(7.5%)이 연계되어 있다. 8개 직무영역과 연계된 134개의 직무항목은 7개 간호사 국가시험 교과목 학습목표를 통합하여 개발한 표준학습목표를 134개의 직무항목과 연계하여 도출한 481개 범주의 지식항목과 연계된다.

직무영역 및 직무항목과 연계된 국가시험 교과목 통합형 표준 학습목표로부터 도출한 481개 범주의 지식항목은 간호관리와 전문성 향상 영역에는 79개(16.4%), 안전과 감염관리 영역에는 25개(5.2%), 위험요인 사정 영역에는 93개(19.3%), 기본간호 영역에는 60개(12.5%), 생리적 통합유지 영역에는 130개(27.0%), 약물 및 비경구 요법 영역에는 14개, 심리적 통합유지 영역에는 43개(9.0%), 건강증진 및 유지 영역에는 37개(7.7%) 지식항목이 연계되어 있다.

3. 간호사 국가시험 출제모형에 근거한 출제기준(안) 제시

가. 직무영역별 국가시험 출제 비율에 대한 유관 단체 전문가 의견조사

출제모형에 근거한 출제기준(안)을 도출하기 위하여 간호사 국가시험 전공교과목 관련 7개 회원학회를 대상으로 출제모형의 직무영역별 국가시험 출제 비율에 대한 의견조사를 실시하였다. 1차 조사(2021.3.5.~2021.3.19.)에서는 8개 직무영역(대분류), 134개 직무항목(중분류), 481개 범주의 지식항목(소분류)에 대한 검토와 회원학회 관련 교과목의 8개 직무영역별 국가시험 출제 비율에 대한 의견을 조사하였다. 7개 회원학회의 1차 조사 결과를 공유하면서 2차 조사(2021.4.30.~2021.5.14.)를 실시하였다.

7개 회원학회의 2차 의견조사 결과에 따른 8개 직무영역별 국가시험 출제분포는 <표IV-29>와 같다. 간호관리와 전문성 향상 영역 41문제(15.0%), 건강증진 및 유지 영역 33문제(12.1%), 기본간호 영역 37문제(13.5%), 생리적 통합유지 영역 63문제(23.1%), 심리 사회적 통합유지 영역 41문제(15.0%), 안전과 감염관리 영역 16문제(5.9%), 약물 및 비경구요법 영역 11문제(4.0%), 위험요인 사정 영역 31문제(11.4%) 등 보건의약관계법규 20문제를 제외한 275문제이었다. 이와 같은 출제 비율은 탁영란 외(2019)의 연구에서 직무영역에 분포한 지식항목에 따라 교과목별로 가중치를 적용한 출제기준(안)과 차이가 있었다. 탁영란 외(2019) 연구에서는 간호관리와 전문성 향상영역 50문제(18.0%), 건강증진 및 유지영역 20문제(7.0%), 기본간호 영역 27문제(10.0%), 생리적 통합유지 영역 55문제(20.0%), 심리 사회적 통합유지 영역 25문제(9.0%), 안전과 감염관리 영역 27문제(10.0%), 약물 및 비경구요법 영역 27문제(10.0%), 위험요인 사정 영역 44문제(16.0%) 등 보건의약관계법규 20문항을 제외한 275문제에 대한 교과목별 비율을 제시하였다. 7개 회원학회에서 제시한 8개 직무영역별 출제비율을 NCLEX-RN(2019), 탁영란 외(2019)와 비교 분석해 보면, 건강증진 및 유지, 기본간호, 생리적 통합유지, 심리사회적 통합유지 등 4개 영역의 비율은 5%~9% 정도 높았고, 간호관리와 전문성 향상, 안전과 감염관리, 약물 및 비경구요법, 위험요인 사정 등 4개 영역의 비율은 5%~11% 정도 낮은 것으로 나타났다.

8개 직무영역별 지식항목의 출제 비율을 살펴보면, 안전과 감염관리 영역의 출제 비율을 탁영란 외(2019)와 NCLEX-RN(2019)은 10~12%로 제시하고 있는데, 본 연구에서 도출한 출제모형에서 안전과 감염관리 영역의 지식항목 분포는 5.2% 정도이고 7개 학회에서 제시한 출제 비율은 6.2%이었다. 약물 및 비경구 요법 영역의 출제 비율을 탁영란 외(2019)와 NCLEX-RN(2019)은 10~15%로 제시하고 있는데, 출제모형의 약물 및 비경구 요법 영역의 지식항목 분포는 2.9% 정도이고 7개 학회에서 제시

한 출제 비율은 4.0%이었다. 반면에 생리적 통합 유지 영역의 출제 비율을 탁영란 외(2019)와 NCLEX-RN(2019)은 14~20% 로 제시하고 있는데, 출제모형의 생리적 통합 유지 영역의 지식항목 분포는 27.0% 정도이고 7개 회원학회에서 제시한 출제 비율은 22.9%이었다. 심리 사회적 통합유지 영역의 출제 비율을 탁영란 외(2019)와 NCLEX-RN(2019)은 9%로 제시하고 있고, 출제모형의 심리 사회적 통합유지 영역의 지식항목 분포도 9% 정도이지만, 7개 회원학회에서 제시한 출제 비율은 14.9%이었다. 그리고 건강증진 및 유지 영역의 출제 비율을 탁영란 외(2019)와 NCLEX-RN(2019)은 7~9%로 제시하고 있는데, 출제모형의 건강증진 및 유지영역의 지식항목 분포도 7.7% 정도이었지만 7개 학회에서 제시한 출제 비율은 12.0%이었다.

간호사 직무항목의 중요도와 수행도를 분석한 IPA 분석 결과에서도 안전과 감염관리 영역[그림 IV-3], 약물과 비경구요법 영역[그림 IV-7]의 중요도와 수행도는 심리사회적 통합유지 영역[그림 IV-8], 건강증진 및 유지 영역[그림 IV-9]보다 높게 나타났다. 7개 회원학회에서 제시한 출제 비율에 대해 협의회 및 병원간호사 등 유관 단체에서도 안전과 감염관리, 약물 및 비경구요법 영역은 임상 및 지역사회 간호 실무현장에서 간호사의 주요한 직무임을 강조하면서, 추후 교육전문가, 임상 및 지역사회 간호 실무현장 전문가로 구성된 TFT에서 출제기준 개발을 위해 8개 직무영역별 지식항목의 출제 비율에 대한 재검토 과정에서 회원학회 간에 충분한 논의가 필요하다는 의견을 제시하였다[부록9].

<표 Ⅳ-29> 출제모형의 직무영역별 국가시험 출제 비율에 대한 7개 회원학회 의견조사

(단위 :개)

직무 영역	직무항목	지식항목	구분	관리	기본	모성	성인	아동	정신	지역	2차 총합
간호관리와전문성향상	22	79	1차	29					2		41
			2차	30				1	2	8	
안전과 감염관리	3	25	1차	4	6		2		2		16
			2차	3	6		2	1	2	2	
위험요인 사정	28	93	1차		2		10		2		31
			2차		2	13	10	2	2	2	
기본간호	19	60	1차		12		15				37
			2차		12		15	8	1	1	
생리적 통합 유지	36	130	1차		6		39		2		63
			2차		6	8	39	7	2	1	
약물 및 비경구요법	7	14	1차		4		2		2		11
			2차		4		2	2	2	1	
심리사회적 통합유지	9	43	1차				2		22		41
			2차			1	2	14	22	2	
건강증진 및 유지	10	37	1차						2		33
			2차			13			2	18	
총합	134	481		33(2)*	30	35	70	35	35	35	273(2)**

* 간호관리학 35문항 중 2문은 간호역사(세계간호사, 한국간호사) 영역 문항으로 현재 제시된 영역에는 포함되어 있지 않음

** 보건의약관계법규 20문항 제외한 275문항임

나. 직무영역별 국가시험 출제 비율 대한 전문가 타당도 조사

직무기반 통합형 간호사 국가시험 출제모형에 근거한 직무영역별 국가시험 출제 비율 타당도를 조사하기 위해서 한국간호과학회, 한국간호대학(과)장협의회, 한국전문대간호학(부)장협의회, 병원간호사회 등 유관 단체를 대상으로 7개 회원학회를 대상으로 실시한 1차, 2차 의견조사 결과를 공유하면서 직무기반 통합형 간호사 국가시험 출제모형에 근거한 출제기준안의 대분류, 중분류, 소분류의 검토와 더불어 8개 직무영역별 출제 비율 범위에 대한 타당성과 적절성에 대한 의견을 조사하였다. 한국간호과학회, 한국간호대학(과)장협의회, 한국전문대간호학(부)장협의회, 병원간호사회 등의 유관 단체 의견조사 결과는 2021년 6월 2일(수)에 개최한 공청회에서 발표하였다[부록 9].

다. 간호사 국가시험 출제모형에 근거한 출제기준(안) 제시

간호사 국가시험 출제모형에 근거한 출제기준(안)에 대한 한국간호과학회 7개 회원학회, 한국간호대학(과)장협의회, 한국전문대간호학(부)장협의회, 병원간호사회 등의 유관 단체 의견조사 결과와 임상 및 지역사회 간호 실무현장 간호사를 대상으로 한 설문조사, 임상전문가 인터뷰 및 내용타당도 조사결과 등에 근거하여 연구진 합의하에 제시한 본 연구에서의 출제기준(안)은 <표 IV-30>과 같다. 보건의약관계법규 20문항은 제외하고, 전체 275문항 중 “간호관리와 전문성 향상“ 영역 50문항, ”안전과 감염관리“영역 33문항, ”위험요인 사정“ 영역 40문항, ”기본간호“ 영역 28문항, ”생리적 통합 유지“ 영역 47문항, ”약물 및 비경구요법“ 영역 33문항, ”심리사회적 통합유지“ 영역 24문항, 그리고 ”건강증진 및 유지“ 영역 20문항이다.

출제기준(안)은 개발과정은 [그림 IV-12]와 같다. 선행연구를 통해서 먼저 출제모형을 확인하고, 직무영역과 연계된 직무항목에 대해 임상 및 지역사회 간호 실무현장 간호사 대상의 설문조사, 임상 전문가 인터뷰 및 내용타당도 조사와 7개 회원학회를 대상으로 출제모형의 직무영역 및 직무항목과 연계된 지식항목의 문항 개발가능성을 확인하여 출제모형의 타당도와 적절성을 규명한 다음에 출제모형을 제시하였다. 출제모형에 근거한 출제기준(안)을 제시하기 위하여 직무영역별 국가시험 출제 비율에 대해 유관 단체의 전문가를 대상으로 의견조사를 하였고, 이에 대한 타당도 조사를 하였다. 이와 같은 의견조사 분석 결과와 더불어 임상 및 지역사회 간호 실무현장 간호사를 대상으로 한 설문조사 결과, 임상전문가 인터뷰 및 내용타당도 조사 결과 등에 근거하여 탁영란 외(2019)의 출제기준안을 기반으로 하면서 NCLEX-RN® Test

Plan(2019)의 영역별 출제 비율을 기준으로 출제기준(안)을 제시하였다.

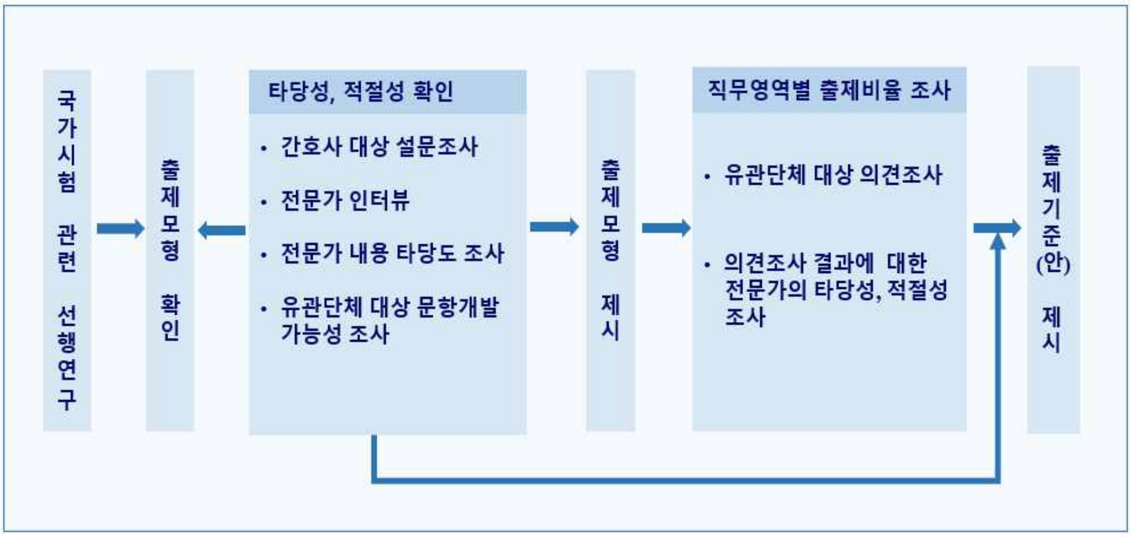

[그림 Ⅳ-12] 출제모형에 근거한 출제기준(안) 개발과정

7개 회원학회에서 제시한 국가시험 직무영역별 출제 비율에서 생리적 통합유지 영역의 비율(23%)이 가장 높고, 안전과 감염관리(6%), 약물 및 비경구 요법 영역(4%)의 비율이 낮은 것은 8개 직무영역에 연계된 지식항목 481개 범주의 직무영역별 분포가 영향을 미치고 있음을 보여준다<표Ⅳ-29>. 7개 회원학회에서 제시한 출제 비율을 NCLEX-RN(2019), 탁영란 외(2019)와 비교 분석해 보면, 건강증진 및 유지, 기본간호, 생리적 통합유지, 심리사회적 통합유지 등 4개 영역의 비율은 5%~9% 정도 높았고, 간호관리와 전문성 향상, 안전과 감염관리, 약물 및 비경구요법, 위험요인 사정 등 4개 영역의 비율은 5%~11% 정도 낮은 것으로 나타났다. 이와 같은 7개 회원학회의 의견조사 결과는 임상 및 지역사회 간호실무 현장 간호사를 대상으로 직무의 중요도와 수행도를 파악하기 위해 실시 한 설문조사 결과와는 차이가 있었다. 간호사 직무항목의 중요도와 수행도를 분석한 IPA 분석 결과에서 안전과 감염관리 영역[그림 Ⅳ-3], 약물과 비경구요법 영역[그림 Ⅳ-7]의 중요도와 수행도는 심리사회적 통합유지 영역[그림 Ⅳ-8], 건강증진 및 유지영역[그림 Ⅳ-9]보다 상대적으로 높게 나타났다. 8개 직무영역별 134개 신규간호사 직무항목의 개선 요구도에 대한 자료분석 결과에서도 삭제에 응답한 응답자의 10% 이상이 항목별로 삭제로 표기한 항목은 간호관리와 전문성 향상 영역에서 「9. 간호사업 평가 관련 업무수행(도구개발, 자료조사, 분석, 비교 및 사업개선)」, 「8. 질 향상(QI) 활동에 참여」, 「16. 지역사회 간호사업의 법적 기준 및 지침에 따른 활동 참여」, 「21. 취약가족에게 필요한 지역사회 자원활용」, 「17. 사례관리활동 참여」 등 5개 항목이었다. 건강증진 및 유지 영역에서는 「129. 문

화간호」,「130. 산업과 환경 간호」,「125. (지역사회)건강교육 계획」 등 3개 항목이었다. 안전과 감염관리, 위험요인 사정, 기본간호, 생리적 통합유지, 약물 및 비경구 요법, 심리사회적 통합유지 영역에서 삭제에 응답한 응답자가 항목별로 삭제로 표기한 항목은 0.77% ~ 4.23% 이었다. 그 중에서도 안전과 감염관리 영역의 3개 직무항목은 1.15% 이하, 약물 및 비경구 요법 영역의 7개 직무항목은 0.77% 이하이었다. 따라서 7개 회원학회의 의견을 출제기준(안)에 반영하기에는 다양한 측면에서 제한이 있었다. 협의회 및 병원간호사회 등 유관 단체에서도 안전과 감염관리, 약물 및 비경구요법은 임상 및 지역사회 간호 실무현장에서 간호사의 주요한 직무임을 강조하면서, 출제기준(안)이 시의성, 객관적 신뢰성을 확보하기 위해서는 추후 교육전문가, 임상 및 지역사회 간호 실무현장 전문가로 구성된 TFT를 구성하여 회원학회 간에 충분한 논의과정을 거쳐야 한다는 의견을 제출하였다[부록9].

이와 같은 분석 결과에 근거하여, 탁영란 외(2019)의 출제기준(안)과 NCLEX-RN® Test Plan(2019 NCLEX-RN®)의 영역별 출제분포를 기반으로, 본 연구에서의 출제기준(안)을 제시하였다. 직무영역과 연계한 지식항목의 분포에 따라 교과목별로 가중치를 적용한 탁영란 외(2019) 출제기준안의 간호관리와 전문성 향상, 기본간호, 심리사회적 통합유지, 건강증진 및 유지 영역 등 4개 직무영역의 출제 비율은 유지하기로 하였다. 탁영란 외(2019)의 출제기준안에서 위험요인 사정 영역은 16%, 생리적 통합유지 영역 20%로 NCLEX-RN® Test Plan(2019)의 영역별 분포 범위보다 높았다. IPA 분석결과, 위험요인 사정, 생리적 통합유지 영역과 연계된 직무항목의 중요도와 수행도는 안전과 감염관리, 약물 및 비경구 요법 영역과 연계된 직무항목의 중요도와 수행도에 비해 상대적으로 낮았다. 이에 따라 위험요인 사정 영역은 NCLEX-RN® Test Plan(2019)의 위험요인 사정 영역 분포(9~15%)의 최대범위 15%로, 생리적 통합유지 영역 분포(11~17%)의 최대범위 17%로 조정하였다. 안전과 감염관리, 약물 및 비경구 요법 등 2개 영역에서 약물 및 경구요법은 NCLEX-RN® Test Plan(2019) 영역의 분포(12%~18%)보다 낮았다. IPA 분석결과 안전과 감염관리, 약물 및 비경구 요법 영역은 신규간호사 직무항목의 중요도와 수행도가 다른 영역에 비해 높게 나타났으며, 실제 임상 및 지역사회에서 간호사의 주요 직무이다. 직무에 기반한 출제기준안임을 고려하여 NCLEX-RN® Test Plan(2019)의 약물 및 비경구요법 영역분포(12~18%)에 따라 10%에서 12%로 상향 조정하였고 안전과 감염관리 영역도10%에서 12%로 상향 조정하였다. 7개 회원학회에서 제시한 직무영역별 국가시험 출제 비율에 대한 유관 단체 전문가 의견조사 결과는 정리하여 향후 교육전문가, 임상 및 지역사회 간호 실무현장 전문가로 구성된 TFT가 진행하는 출제기준 개정작업에 반영할 수 있도록 하였다[부록8], [부록9].

<표 Ⅳ-30> **간호사 국가시험 출제모형에 근거한 출제기준(안)**

직무 영역	NCLEX-RN (2019) 단위:(%)	지식 항목 단위:n(%)	탁영란 외 (2019) 단위:n(%)	본 연구의 출제기준(안) 단위:n(%)
간호관리와 전문성 향상	(17~23, 20)	79(16)	50(18)	50(18)
안전과 감염관리	(9~15, 12)	25(5)	28(10)	33(12)
위험요인 사정	(9~15, 12)	93(19)	43(16)	40(15)
기본간호	(6~12, 9)	60(13)	28(10)	28(10)
생리적 통합 유지	(11~17, 14)	130(27)	55(20)	47(17)
약물 및 비경구요법	(12~18, 15)	14(3)	28(10)	33(12)
심리사회적 통합유지	(6~12, 9)	43(9)	24(9)	24(9)
건강증진 및 유지	(6~12, 9)	37(8)	19(7)	20(7)
합	100%	481(100)	275(100)	275(100)

* 보건의료관계법규 20문항은 제외한 275문항임

4. 간호사 국가시험 출제기준 최종(안) 도출 및 전문가 의견수렴

가. 공청회 개최

직무기반 통합형 간호사 국가시험 출제모형의 8개 실무영역 및 134개 직무항목과 연계된 481개 범주의 지식항목에 대한 7개 회원학회의 1차, 2차 의견조사 결과와 국가시험 출제기준안에 대해 전문가 의견을 수렴하여 합의를 도출하기 위하여 전국 간호대학 교수와 임상 및 지역사회 간호 실무현장 전문가를 대상으로 공청회를 개최하였다<표 Ⅳ-31>.

1) 공청회 일정 및 참석자

공청회는 2021년 6월 2일 수요일 오후 1시부터 5시 40분까지 온라인 Zoom을 이용하여 개최되었다. 한국간호과학회, 한국간호대학(과)장협의회, 한국전문대학(부)장협의회, 병원간호사회 등 유관 단체를 통해 이메일로 공청회 일정을 공지하였다<표 Ⅳ-31>. 사전등록자는 273명이었고 참석자는 약 200명이었다.

<표 Ⅳ-31> 공청회 프로그램

시간	내용	발표자	
13:00~13:20	등록		
13:20~13:30	개회 및 인사 말씀	김경희	(국시원 간호사국가시험위원장)
13:30~13:45	발표1<br>간호사 국가시험 출제 기준(안) 기초연구	강윤희<br>김현정<br>김경희	(공동책임자)<br>(공동책임자)<br>(연구책임자)
13:45~14:10	발표2<br>간호사 국가시험 통합의 향후 방향	서연옥	(국시원 간호사 임기제 시험위원)
14:10~15:30	패널토의 1<br>좌장<br>[토론자]	송영신<br>김현찬<br>서연옥<br>송준아<br>유정숙	(대한간협 간호사국가시험 위원)<br>(국시원 출제운영본부장)<br>(국시원 간호사 임기제 시험위원)<br>(대한간협 간호표준위원장)<br>(국시원 간호사 임상실무위원)
15:30~15:40	휴식		
15:40~17:00	패널토의 2<br>좌장<br>[토론자]	송영신<br>김순오<br>은 영<br>조경숙<br>조문숙	(대한간협 간호사국가시험 위원)<br>(한국전문대간호학(부)장 협의회장)<br>(한국간호대학(과)장 협의회장)<br>(한국간호과학회장)<br>(병원간호사회장)
17:00~17:40	종합토론 및 질의응답		
17:40	폐회		

2) 공청회에서 제시된 의견

공청회에서 한국간호과학회, 한국간호대학(과)장협의회, 한국전문대학(부)장 협의회, 병원간호사회 등 유관 단체의 의견과 발표자, 토론자, 참석자들로부터 제시된 의견을 간호사 국가시험 출제모형, 8개 직무영역, 134개 직무항목, 481개 범주의 지식항목, 신규간호사의 간호 실무현장 적응 등으로 범주화하여 정리하였다.

가) 간호사 국가시험 출제모형

간호사 국가시험 출제모형에 대해 제시된 의견을 방향성, 절차의 정당성, 출제관리시스템으로 분류하여 정리하였다.

① 방향성

간호사 면허 취득을 위한 국가시험의 방향성은 첫째, '전공교과목의 지식수준 평가'에서 '실무에서 요구되는 신규간호사의 역량 평가'로 방향성이 전환되어야 한다. 이를 위해서 대한간호협회에서는 신규간호사의 직무에 기반한 통합형 간호사국가시험 출제모형과 출제기준이 객관적 정당성을 확보할 수 있도록 대한간호협회 산하단체로부터 추천된 간호 교육전문가, 임상 및 지역사회 간호 실무현장 전문가들로 구성된 TFT(Task Force Team, TFT)를 구성하여 충분한 논의과정과 의견수렴을 통하여 국가시험 출제모형과 출제기준에 대한 합의를 도출하여야 한다. 둘째, 국가시험은 보건의료환경 변화에 따른 21세기 간호사에게 기대되는 역량을 제대로 평가할 수 있어야 하므로, 최근 인구의 고령화로 노인간호 분야의 중요성이 부각되고 있는 현실적인 상황이 출제기준에 고려되어야 출제문항의 시의성, 객관성, 신뢰성이 확보될 수 있다. 셋째, 국가시험의 질 향상을 위해서는 단계별로 절차의 정당성을 확보하면서 국시원의 적극적인 지원하에 출제관리 시스템이 체계적으로 구축되어야 한다. 출제관리 시스템이 구축되면 컴퓨터 기반 시험(Computer Based Test, CBT)을 위한 사전준비가 착수되어 예비실행 단계를 거쳐서 CBT가 실행됨으로써, 궁극적으로는 컴퓨터 개별적응 시험 CAT(Computerized adaptive testing, CAT)기반을 조성하는 방향으로 나아갈 수 있다[부록9].

② 절차의 정당성

절차의 정당성에 대해 제시된 의견은 간호사 국가시험 출제모형의 8개 직무영역, 134개 신규간호사 직무항목 및 481개 범주의 지식항목 개발 과정에서 충분한 의견수렴 및 신뢰성과 타당성 검증이 이루어져야 한다고 제시하였다[부록9].

③ 출제관리 시스템

출제관리 시스템에 대해 제시된 의견은 첫째, 간호실무의 실제성과 최신성을 반영하는 문항을 개발하고 개정하기 위해서는 실무현장의 전문가들과 교육현장 전문가들의 합의 과정과 질 관리를 위한 지속적인 문항 검토 시스템이 구축되어야 한다. 둘째, 좋은 문항 개발을 위한 인프라가 구축되면 우선적으로 난이도 안정화를 위한 방안이 마련되어야 한다. 마지막으로, 간호 상황에 적합한 임상판단을 할 수 있는 통합적 사고 능력을 평가하기 위한 문항을 개발하기 위해서는 임상 및 지역사회에서 실무경험이 많은 퇴직 간호사들을 적극 활용하여 국시원의 재정적 지원 하에 사례 중심의 문제해결형 문항을 개발하여야 한다[부록9].

나) 8개 직무영역(대분류)

8개 직무영역에 대한 의견으로 첫째, 직무영역에 대한 정확한 이해를 위해 4가지 영역(위험요인 사정, 기본간호, 생리적 통합유지, 약물 및 비경구요법)은 영역별 정의 및 목표를 통해 명확하게 구분되어야 한다. 둘째, 8개의 직무영역이 어떤 배열순서(sequence)를 가지는지 체계 정리가 필요하다[부록9].

다) 134개 신규간호사 직무항목(중분류)

134개 신규간호사 직무항목에 대해 제시된 의견은 일부 직무항목의 중복, 구체성 수준의 차이 및 직무항목과 관련 교과목과의 연계성에 대해서 관련 학회, 유관 단체, 교육전문가, 임상 및 지역사회 간호실무 현장 전문가 집단의 의견수렴과 합의 과정을 거쳐서 재정리 되어야 한다[부록9].

라) 481개 범주의 지식항목(소분류)

481개 범주의 지식항목에 대한 의견으로 첫째, 지식항목이 범주별로 구체성 수준에 차이가 있으므로 1) 지식항목의 구체성 수준, 2) 전공 교과목과 연계되지 않은 지식항

목, 3) 2개 이상의 전공 교과목과 연계된 지식항목, 4) 범주별 지식항목에서 수정, 삭제 및 추가할 항목 등에 대해 교육, 임상 및 지역사회 전문가 집단의 의견수렴과 합의 과정을 거쳐서 재정리하여야 한다. 둘째, 481개 범주에서 간호사 국가시험 275개(보건의약관계법규 20개 문항 포함되지 않음)문제 분포의 객관적 정당성을 확보 할 수 있는 방안을 모색하여야 한다[부록9].

마) 신규간호사의 간호실무 현장 적응

신규간호사의 간호실무 현장 적응에 대해 임상전문가들의 의견으로 간호사 면허를 취득한 신규간호사가 병원에 입사하면, 충분히 준비되지 못한 상태에서 임상실무 현장에서의 업무수행을 힘겨워 하여 여전히 적응에 어려움을 겪고 있다고 제시하였다. 이에 신규간호사의 1년 이내 사직률은 지속적으로 증가 추세를 보이고 있다. 이러한 상황에서 국내 대부분의 병원에서는 신규간호사가 임상 현장에 필요한 역량을 갖추고, 임상 실무에 안정적으로 역할 전이가 일어나도록 실무적응 지원을 위한 프리셉터십을 운영하고 있으며, 일부 상급종합병원과 종합병원에서는 프리셉터십과 더불어 신규간호사의 실무적응 지원을 위한 다양한 교육프로그램을 운영하고 있다. 신규간호사의 역량은 간호의 질, 환자 안전과 직접적인 관련이 있으므로, 간호학생이 졸업 후 신규간호사로서 역할전이를 잘 할 수 있도록 신규간호사의 직무역량을 평가하는 국가시험으로 방향이 전환되어야 할 필요가 있다[부록9].

V. 결론 및 제언

본 연구는 신규간호사 직무기반 통합형 간호사 국가시험 출제모형에 근거한 국가시험 출제기준안을 제시함으로써 국가시험의 패러다임을 전공교과목의 지식검증에서 신규간호사의 직무역량 평가로 전환할 수 있는 기초자료를 제공하기 위함이었다. 본 연구는 단계별로 순차적으로 진행된 선행연구를 바탕으로 신규간호사 직무기반 통합형 출제모형과 출제모형에 근거한 출제기준안을 제시하여 이에 대한 교육전문가, 임상 및 지역사회 간호 실무현장 전문가 집단의 의견을 조사하여 향후 출제기준을 개발하는데 유용하게 활용할 수 있는 기초자료를 제공해 준다는 점에서 의의가 있다. 연구결과를 통해 도출한 결론은 다음과 같다.

1. 간호사 국가시험 출제모형

간호사 국가시험은 간호대학 졸업자가 간호 실무현장에서 최신의 신규간호사 직무에 기반한 직무를 수행하는데 필요한 최소한의 능력을 평가하여야 한다. 이러한 목적에 부합하는 간호사 국가시험 출제모형을 제시하기 위하여 국내외 문헌을 고찰하였다. 문헌고찰을 통해서 선행연구에서 단계별로 순차적으로 개발 한 간호사 국가시험 출제모형을 확인하였다. 출제모형을 구성하는 주개념인 신규간호사 직무영역별 직무항목에 대해 임상 및 지역사회 간호사를 대상으로 한 시의성, 타당성, 적절성 평가와 임상 및 지역사회 간호 실무현장 전문가를 대상으로 내용타당도 검정을 한 후, 직무기반 통합형 간호사 국가시험 출제모형을 확인하였다. 확인된 국가시험 출제모형의 8개 실무영역, 134개의 실무항목, 481개 범주의 지식항목에 대해 시험문제로의 개발가능성과 평가실행 가능성에 대해 간호사 국가시험 교과목 관련 7개 학회를 대상으로 1차, 2차에 걸쳐서 의견조사를 진행하였다. 7개 회원학회의 의견조사에서 출제모형의 시험문제로의 개발가능성과 평가실행 가능성을 확인한 후 직무기반 간호사 국가시험 출제모형을 제시하였다. 출제모형은 직무영역(대분류), 직무항목(중분류), 표준학습목표(교과목 간 학습목표의 중복성을 피하는 통합형 학습목표), 지식항목(소분류) 등의 주개념으로 구성된다. 간호사 직무를 분석하여 개발한 8개 직무영역에 연계된 134개 직무항목, 직무항목 및 통합형 표준학습목표와 연계된 481개 범주의 지식항목으로 구성된 출제모형은 간호사 국가시험의 패러다임이 직무기반 지식검증으로 전환될 수 있는 근거가 된다.

7개 학회, 한국간호과학회, 한국간호대학(과)장협의회, 한국간호전문대학(부)장협의

회, 병원간호사회 등 유관 단체 그리고 공청회에서 전문가들은 직무기반 통합형 간호사 국가시험 출제모형이 객관적 정당성을 확보하기 위해서는 교육전문가, 임상 및 지역사회 간호 실무현장 전문가로 구성된 TFT를 구성하여 충분한 논의과정을 거쳐서 협의회 및 유관 단체, 그리고 공청회에서 제시된 의견이 반영될 수 있도록 하여야 한다는 합의를 도출하였다. 이에 본 연구에서는 출제모형에 대한 학회, 단체 및 협의회의 의견을 정리하여 향후 TFT의 논의과정과 의견수렴 과정에 반영될 수 있도록 하였다.

2. 간호사 국가시험 출제모형에 근거한 출제기준(안)

출제기준은 시험문제를 출제하는 기준으로 시험과목 및 대항목, 분야(또는 영역), 시험문제수, 배점 등으로 구성된다. 연구진 합의하에 제시한 본 연구에서의 출제기준(안)은 탁영란 외(2019)의 출제기준(안)과 NCLEX-RN® Test Plan(2019 NCLEX-RN®)의 영역별 출제분포를 기반으로, 간호사 국가시험 출제모형에 근거한 출제기준(안)에 대한 7개 회원학회, 한국간호대학(과)장협의회, 한국전문대간호학(부)장협의회, 병원간호사회 등의 유관 단체 의견조사 결과와 임상 및 지역사회 간호 실무현장 간호사를 대상으로 한 설문조사, 임상전문가 인터뷰 및 내용타당도 조사결과 등을 근거로 하였다. 직무영역과 연계한 지식항목의 분포에 따라 교과목별로 가중치를 적용한 탁영란 외(2019) 출제기준(안)의 간호관리와 전문성 향상 18%, 기본간호 10%, 심리사회적 통합유지 9%, 건강증진 및 유지 영역 7% 등 4개 직무영역의 출제분포는 유지하기로 하였다. 탁영란 외(2019) 출제기준안에서 위험요인 사정 영역은 16%에서 15%로, 생리적 통합유지 영역 20%에서 15%로 하향 조정하였다. 약물 및 비경구요법 영역은 10%에서 12%로 상향 조정하였고 안전과 감염관리도 10%에서 12%로 상향 조정하였다. 7개 회원학회의 출제기준(안)에 대한 유관 단체의 전문가 의견조사 결과는 정리하여 향후 교육전문가, 임상 및 지역사회 간호 실무현장 전문가로 구성된 TFT가 진행하는 출제기준 개정작업에 반영할 수 있도록 하였다.

국가시험 출제모형과 출제기준(안)의 기초자료를 제시한 본 연구가 신규간호사 직무역량을 평가하는 간호사 국가시험의 선진화를 위한 컴퓨터 기반 시험(Cumputer Based Test, CBT) 사전 준비 작업의 기초자료로 활용될 수 있기를 기대한다.

간호사 국가시험의 질 향상을 위해서는 앞으로 CBT 사전준비 과정을 거쳐서 CBT를 시행하면서 컴퓨터 개별 적응 시험(Computerized Adaptive Testing, CAT)을 위한 기반을 조성하여야 한다. 또한, 난이도 안정화 전략과 출제문제 수 5배수 이상의 문

항개발 방안이 모색되어야 한다. 이를 위해서는 우선적으로 간호사가 갖추어야 할 최소역량을 기술하여 실제 임상능력을 평가할 수 있는 국가시험 평가목표(안)이 개발되어야 할 필요성이 있다. 개발된 국가시험 평가목표(안)에 기반하여 난이도 안정화 전략과 출제 문제 수의 5배수 이상 문항개발 방안이 마련되어야 한다

이상의 연구결과와 공청회에서 제시된 의견을 기반으로 다음과 같은 제언을 하고자 한다.

첫째, 본 연구가 교육, 임상 및 간호실무 현장의 전문가 집단을 대상으로 한 의견조사 및 공청회를 통하여 출제기준이 직무에 기반한 통합형으로 개정되어야 하는 필요성을 확고히 하였으므로, 직무기반 통합형 출제모형에 근거한 출제기준을 개발하여 간호사 국가시험 출제기준 개정작업이 진행되기를 제언한다.

둘째, 출제모형에 대해 중복되는 직무항목 및 지식항목, 통합되어야 할 지식항목의 범주, 지식항목 범주를 구성하는 항목의 적절성 등 유관 단체에서 제출한 의견을 정리한 본 연구 결과를 추후 출제모형 재검토 과정에서 기초자료로 활용하기를 제언한다.

셋째, 출제모형의 직무영역별 국가시험 출제 비율에 대한 유관 단체의 전문가 의견과 이에 대한 분석 내용을 정리한 본 연구 결과를 추후 회원학회 간 의견조율 과정에서 기초자료로 활용하기를 제언한다.

참고문헌

강소영, 김경희, 김광성, 박인숙, 박혜숙, 서연옥, 안수연 (2015). 간호대학 졸업자의 최소직무능력규정 및 국가시험 적용방안연구(보고서 번호 RE02-1607-05). 서울; 한국보건의료인국가시험원.

권인각, 조용애, 조명숙, 이영희, 김미순, 김경숙, 최애선. (2018). 신규간호사의 실무 적응지원 교육 현황 및 신규간호사의 역할이행 경험. 병원간호사회 연구보고서.

김금순, 강윤희, 구현영, 권명진, 김남초, 김옥수, 박정숙, 신혜숙, 안옥희, 장성옥 (2013). 간호사 국가시험과목제도 개선에 관한 연구, 한국보건의료인국가시험원.

김영경, 김경희, 김명희, 김선희, 김연화, 신수진, 안성희, 오상은, 이경미 (2018). 직무기반 간호사 국가시험을 위한 문항개발기준 및 문항개발절차 개선 연구(보고서 번호 RE02-1814-05). 서울; 한국보건의료인국가시험원.

박인숙, 서연옥, 안수연, 박혜숙, 강소영, 김광성 (2014). 간호사 직무분석, 학습목표, 국가시험 연계성 분석연구(보고서 번호 RE02-1533-05). 서울; 한국보건의료인국가시험원.

박인숙, 강소영, 고일선, 박인혜, 박혜숙, 서연옥, 안수연 (2012). 간호사 2차 직무분석연구(보고서 번호 RE3-1210-05). 서울; 한국보건의료인국가시험원.

송라윤, 김영경, 강소영, 박영우, 박현숙, 신수진, 신용순, 오상은, 정금희 (2018). 간호사 국가시험 통합모형 구축. 서울: 대한간호협회.

조정숙, 김명하, 김선례, 박완임, 조재옥, 고미혜 (2020). 2020년 병원간호인력 배치현황 실태조사. 병원간호사회 사업보고서.

탁영란, 강윤희, 송라윤, 송준아, 한민경 (2019). 보건의료환경 변화에 따른 간호사 국가시험 및 면허관리체계 개선방안(보고서 번호 RE02-1913-05). 서울; 한국보건의료인국가시험원.

Ignatavicius, D. D. (2021). Preparing for the new nursing licensure exam: The next-generation NCLEX. Nursing 2021, 51(5), 34-41.

National Council of State Board Nursing. (2018). 2019 NCLEX-RN Test Plan. Chicago: Author. https://www.ncsbn.org/2019_RN_TestPlan-English.pdf

National Council of State Board Nursing. (2018). Report of Findings from the 2017 RN Nursing Knowledge Survey. https://www.ncsbn.org/2017_RN_KSA_final.pdf

Singh-Carlson, S., & May, K. A. (2020). Adoption of NCLEX-RN for licensure in Canada: Faculty concerns and implications for nursing education. Journal of Professional Nursing, 36(2), 77-82.

Victor, J., Chavez, L. S., & Podlesney, C. (2021). Do Predictor Exams Really Predict Readiness for Professional Nursing Practice?. Clinical Simulation in Nursing, 50, 48-54.

【부록1】 간호사 직무분석 관련 설문조사지

안녕하십니까? 귀하의 무궁한 발전을 기원합니다.

대한간호협회 간호사국가시험위원회에서는 한국보건의료인국가시험원 2020년도 자유주제 위탁연구 과제 '간호사 국가시험의 통합모형에 근거한 출제기준개발 기초연구'를 진행하고 있습니다.

본 설문조사는 간호사 국가시험의 출제기준을 개발하기 위해서는 최신의 간호사 직무분석이 선행되어야 하므로 이에 대한 의견을 수렴하고자 실시하고 있습니다.

귀하의 성실한 답변은 향후 간호사 국가시험 출제기준 개발에 소중한 자료로 활용될 것이며, 조사 결과는 오직 조사목적인 간호사 국가시험 출제기준 개발에만 활용될 예정이오니, 빠짐없이 답변해 주시기를 부탁드립니다.

본 조사지는 현재 간호 실무 현장에 근무 중인 간호사를 대상으로 실시하고 있습니다. **2021년 1월 7일(목)까지 응답**해 주셔서 기간 내에 설문이 회수될 수 있도록 협조해 주시기 바랍니다. 바쁘신 중에도 설문에 성실히 응해 주심에 감사드립니다.

설문의 목적을 이해하고 참여에 동의합니다. □
휴대전화번호
귀하의 휴대전화번호는 익명성이 보장된 상태에서 중복 응답의 확인 및 소정의 선물 제공에만 사용되며, 이후 즉시 삭제됩니다.

2020.12.22. 책임연구자 김경희
공동연구자 강소영 강윤희 권영란 김현정 송영신 조주연 최미영

==

✿ 설문과 관련하여 문의사항이 있으시면, 아래 담당자에게 연락 주십시오.
담당: 김 경 희 (Tel. 02-820-5670 ▌ kyung@cau.ac.kr)

▪ **다음은 귀하의 일반적인 사항에 대한 문항입니다. 해당하는 곳에 ✔표하여 주십시오.**

1. 귀하의 직위는 다음 중 어디에 해당합니까?
① 일반간호사 ② 주임, 책임간호사 ③ 수간호사 ④ 간호관리자
⑤ 보건교사 ⑥ 간호직 공무원 ⑦ 기타

2 귀하가 근무하는 기관의 소재지는 어디입니까?
① 수도권 ② 강원권 ③ 충청권
④ 전라권 ⑤ 경상권 ⑥ 제주권

3. 귀하가 근무하는 기관은 다음 중 어디에 해당합니까?
① 상급종합병원 ② 종합병원 ③ 병·의원
④ 학교(보건교사) ⑤ 보건소 ⑥ 기타

4. 귀하의 간호사로서의 근무기간은 다음 중 어디에 해당합니까?
① 1년 미만 ② 1 ~ 3년 미만 ③ 3 ~ 5년 미만
④ 5 ~ 7년 미만 ⑤ 7년 이상

5. 귀하의 현 직장의 근무기간은 다음 중 어디에 해당합니까?
① 1년 미만 ② 1 ~ 3년 미만 ③ 3 ~ 5년 미만
④ 5 ~ 7년 미만 ⑤ 7년 이상

6. 귀하가 현재 근무하고 있는 부서는 다음 중 어디에 해당합니까?
① 내과계 병동 ② 외과계 병동 ③ 중환자실 ④ 소아청소년과 병동
⑤ 분만실, 산부인과병동 ⑥ 신생아실 ⑦ 응급실 ⑧ 정신과 병동
⑨ 수술실/마취회복실 ⑩ 학교 ⑪ 보건소 ⑫ 기타

▪ 다음은 선행연구에서 도출한 영역별 간호사 직무입니다. 각 영역의 직무별로 (1) **해당 직무가 신규간호사 직무로 중요한 정도**, (2) **신규간호사가 실제로 수행할 수 있는 정도**, (3) **신규간호사 직무에 대한 개선 요구를** ✔표 하여 주시기 바랍니다. **각 영역마다 추가되어야 할 직무가 있으면 영역별로 하단의 빈칸에 기술해 주십시오.**

영역	번호	아래의 간호사 직무관련 각 문항별로 오른쪽 3가지 사항 모두에 ✔표 해 주십시오.	(1) 신규간호사 직무로 중요한 정도					(2) 신규간호사가 수행 할 수 있는 정도					(3) 신규간호사 직무 개선 요구			
			중요하지 않다	↔			중요하다	수행할 수 없다		↔		수행할 수 있다	아래 넷 중 하나 선택			
		응답예시	1	2	3	4	✔	1	2	3	✔	5	✔	분리	통합	삭제
I. 간호관리와 전문성 향상	1	인수인계시행	1	2	3	4	5	1	2	3	4	5	유지	분리	통합	삭제
	2	법적 실무범위 내에서 간호 수행	1	2	3	4	5	1	2	3	4	5	유지	분리	통합	삭제
	3	기록시 표준화된 약어사용	1	2	3	4	5	1	2	3	4	5	유지	분리	통합	삭제
	4	지침에 따라 간호기록	1	2	3	4	5	1	2	3	4	5	유지	분리	통합	삭제
	5	입원, 전동, 퇴원	1	2	3	4	5	1	2	3	4	5	유지	분리	통합	삭제
	6	장비를 적절하고 안전하게 사용	1	2	3	4	5	1	2	3	4	5	유지	분리	통합	삭제
	7	간호단위 물품교환체계에 따른 물품관리	1	2	3	4	5	1	2	3	4	5	유지	분리	통합	삭제
	8	질 향상(QI) 활동에 참여	1	2	3	4	5	1	2	3	4	5	유지	분리	통합	삭제
	9	간호사업 평가 관련 업무수행(도구개발, 자료조사, 분석, 비교 및 사업개선)	1	2	3	4	5	1	2	3	4	5	유지	분리	통합	삭제
	10	간호전문직 윤리 준수와 역할	1	2	3	4	5	1	2	3	4	5	유지	분리	통합	삭제
	11	대상자의 개인정보 및 사생활 보호	1	2	3	4	5	1	2	3	4	5	유지	분리	통합	삭제
	12	대상자에게 치료 및 절차에 따라 적절한 설명을 하고 동의를 획득하였는지 확인	1	2	3	4	5	1	2	3	4	5	유지	분리	통합	삭제
	13	환자의 권리와 책임에 관하여 대상자에게 교육 제공	1	2	3	4	5	1	2	3	4	5	유지	분리	통합	삭제
	14	억제대 사용 시 법적 및 윤리적 간호	1	2	3	4	5	1	2	3	4	5	유지	분리	통합	삭제
	15	일차의료기반의 보건간호	1	2	3	4	5	1	2	3	4	5	유지	분리	통합	삭제
	16	지역사회 간호사업의 법적 기준 및 지침에 따른 활동 참여	1	2	3	4	5	1	2	3	4	5	유지	분리	통합	삭제

영역	번호	아래의 간호사 직무관련 각 문항별로 오른쪽 3가지 사항 모두에 ✔표 해 주십시오.	(1) 신규간호사 직무로 중요한 정도					(2) 신규간호사가 수행 할 수 있는 정도					(3) 신규간호사 직무 개선 요구			
			중요하지 않다 ↔ 중요하다					수행할 수 없다 ↔ 수행할 수 있다					아래 넷 중 하나 선택			
	17	사례관리활동 참여	1	2	3	4	5	1	2	3	4	5	유지	분리	통합	삭제
	18	업무를 조직화하여 일을 효율적으로 관리	1	2	3	4	5	1	2	3	4	5	유지	분리	통합	삭제
	19	환자분류체계 관련 정보수집 및 활용	1	2	3	4	5	1	2	3	4	5	유지	분리	통합	삭제
	20	간호대상에 적합한 다양한 자원 및 매체선택	1	2	3	4	5	1	2	3	4	5	유지	분리	통합	삭제
	21	취약가족에게 필요한 지역사회 자원활용	1	2	3	4	5	1	2	3	4	5	유지	분리	통합	삭제
	22	전문직간 협업	1	2	3	4	5	1	2	3	4	5	유지	분리	통합	삭제
	I. 간호관리와 전문성 향상 : 추가 항목															

영역	번호	아래의 간호사 직무관련 각 문항별로 오른쪽 3가지 사항 모두에 ✔표 해 주십시오.	(1) 신규간호사 직무로 중요한 정도					(2) 신규간호사가 수행 할 수 있는 정도					(3) 신규간호사 직무 개선 요구			
			중요하지 않다 ↔ 중요하다					수행할 수 없다 ↔ 수행할 수 있다					아래 넷 중 하나 선택			
II. 안전과 감염관리	23	안전한 환경 제공	1	2	3	4	5	1	2	3	4	5	유지	분리	통합	삭제
	24	감염관리	1	2	3	4	5	1	2	3	4	5	유지	분리	통합	삭제
	25	위험물질과 유해물질 관리	1	2	3	4	5	1	2	3	4	5	유지	분리	통합	삭제
	II. 안전과 감염관리 : 추가 항목															

영역	번호	아래의 간호사 직무관련 각 문항 별로 오른쪽 3가지 사항 모두에 ✔표 해 주십시오.	(1) 신규간호사 직무로 중요한 정도					(2) 신규간호사가 수행 할 수 있는 정도					(3) 신규간호사 직무 개선 요구			
			중요하지 않다 ↔ 중요하다					수행할 수 없다 ↔ 수행할 수 있다					아래 넷 중 하나 선택			
Ⅲ. 위험요인사정	26	간호계획, 진료지침 수행	1	2	3	4	5	1	2	3	4	5	유지	분리	통합	삭제
	27	대상자의 건강문제에 대해 우선순위 결정	1	2	3	4	5	1	2	3	4	5	유지	분리	통합	삭제
	28	활력징후 사정	1	2	3	4	5	1	2	3	4	5	유지	분리	통합	삭제
	29	신생아 간호	1	2	3	4	5	1	2	3	4	5	유지	분리	통합	삭제
	30	영아 간호	1	2	3	4	5	1	2	3	4	5	유지	분리	통합	삭제
	31	유아 간호	1	2	3	4	5	1	2	3	4	5	유지	분리	통합	삭제
	32	학령전기 간호	1	2	3	4	5	1	2	3	4	5	유지	분리	통합	삭제
	33	학령기 간호	1	2	3	4	5	1	2	3	4	5	유지	분리	통합	삭제
	34	청소년 간호	1	2	3	4	5	1	2	3	4	5	유지	분리	통합	삭제
	35	폐경기 여성 간호	1	2	3	4	5	1	2	3	4	5	유지	분리	통합	삭제
	36	노인간호	1	2	3	4	5	1	2	3	4	5	유지	분리	통합	삭제
	37	성 건강 간호	1	2	3	4	5	1	2	3	4	5	유지	분리	통합	삭제
	38	건강력 사정	1	2	3	4	5	1	2	3	4	5	유지	분리	통합	삭제
	39	신체검진 수행 및 결과 해석	1	2	3	4	5	1	2	3	4	5	유지	분리	통합	삭제
	40	생식기 건강사정	1	2	3	4	5	1	2	3	4	5	유지	분리	통합	삭제

영역	번호	아래의 간호사 직무관련 각 문항 별로 오른쪽 3가지 사항 모두에 ✔표 해 주십시오.	(1) 신규간호사 직무로 중요한 정도					(2) 신규간호사가 수행 할 수 있는 정도					(3) 신규간호사 직무 개선 요구			
			중요하지 않다 ↔ 중요하다					수행할 수 없다 ↔ 수행할 수 있다					아래 넷 중 하나 선택			
Ⅲ. 위험요인사정	41	태아건강사정 및 간호	1	2	3	4	5	1	2	3	4	5	유지	분리	통합	삭제
	42	신생아 건강사정 및 간호	1	2	3	4	5	1	2	3	4	5	유지	분리	통합	삭제
	43	고위험신생아 건강사정 및 간호	1	2	3	4	5	1	2	3	4	5	유지	분리	통합	삭제
	44	심폐소생술 간호	1	2	3	4	5	1	2	3	4	5	유지	분리	통합	삭제
	45	응급간호	1	2	3	4	5	1	2	3	4	5	유지	분리	통합	삭제
	46	진단검사 간호	1	2	3	4	5	1	2	3	4	5	유지	분리	통합	삭제
	47	수술간호	1	2	3	4	5	1	2	3	4	5	유지	분리	통합	삭제
	48	산전간호와 교육	1	2	3	4	5	1	2	3	4	5	유지	분리	통합	삭제
	49	분만 중 간호와 교육	1	2	3	4	5	1	2	3	4	5	유지	분리	통합	삭제
	50	산후관리와 교육	1	2	3	4	5	1	2	3	4	5	유지	분리	통합	삭제
	51	고위험 임부간호	1	2	3	4	5	1	2	3	4	5	유지	분리	통합	삭제
	52	고위험 산부간호	1	2	3	4	5	1	2	3	4	5	유지	분리	통합	삭제
	53	고위험 산모간호	1	2	3	4	5	1	2	3	4	5	유지	분리	통합	삭제
	**Ⅲ. 위험요인 사정 : 추가 항목**															

영역	번호	아래의 간호사 직무관련 각 문항 별로 오른쪽 3가지 사항 모두에 ✔표 해 주십시오.	(1) 신규간호사 직무로 중요한 정도					(2) 신규간호사가 수행 할 수 있는 정도					(3) 신규간호사 직무 개선 요구			
			중요하지 않다 ↔ 중요하다					수행할 수 없다 ↔ 수행할 수 있다					아래 넷 중 하나 선택			
Ⅳ. 기본간호	54	체온유지간호	1	2	3	4	5	1	2	3	4	5	유지	분리	통합	삭제
	55	세척(irrigation)(방광, 귀, 눈) 수행	1	2	3	4	5	1	2	3	4	5	유지	분리	통합	삭제
	56	개인위생간호	1	2	3	4	5	1	2	3	4	5	유지	분리	통합	삭제
	57	섭취량 및 배설량 사정과 간호	1	2	3	4	5	1	2	3	4	5	유지	분리	통합	삭제
	58	영양사정 및 관리	1	2	3	4	5	1	2	3	4	5	유지	분리	통합	삭제
	59	질환별 영양문제의 사정 및 관리	1	2	3	4	5	1	2	3	4	5	유지	분리	통합	삭제
	60	섭취장애 대상자 사정 및 간호	1	2	3	4	5	1	2	3	4	5	유지	분리	통합	삭제
	61	배뇨장애 대상자 사정 및 간호	1	2	3	4	5	1	2	3	4	5	유지	분리	통합	삭제
	62	투석대상자 관리	1	2	3	4	5	1	2	3	4	5	유지	분리	통합	삭제
	63	요루 및 장루관리	1	2	3	4	5	1	2	3	4	5	유지	분리	통합	삭제
	64	기관절개부 관리	1	2	3	4	5	1	2	3	4	5	유지	분리	통합	삭제
	65	배변관리	1	2	3	4	5	1	2	3	4	5	유지	분리	통합	삭제
	66	수면과 휴식 간호	1	2	3	4	5	1	2	3	4	5	유지	분리	통합	삭제
	67	피부통합성 사정 및 간호	1	2	3	4	5	1	2	3	4	5	유지	분리	통합	삭제
	68	이동간호	1	2	3	4	5	1	2	3	4	5	유지	분리	통합	삭제
	69	활동과 자기돌봄 장애 사정 및 간호	1	2	3	4	5	1	2	3	4	5	유지	분리	통합	삭제
	70	외과적 장치 관리	1	2	3	4	5	1	2	3	4	5	유지	분리	통합	삭제
	71	척추손상 및 신경질환 대상자의 간호	1	2	3	4	5	1	2	3	4	5	유지	분리	통합	삭제
	72	관절대치술 환자 간호	1	2	3	4	5	1	2	3	4	5	유지	분리	통합	삭제
	Ⅳ. 기본간호 : 추가 항목															

영역	번호	아래의 간호사 직무관련 각 문항 별로 오른쪽 3가지 사항 모두에 ✔표 해 주십시오.	(1) 신규간호사 직무로 중요한 정도					(2) 신규간호사가 수행 할 수 있는 정도					(3) 신규간호사 직무 개선 요구			
			중요하지 않다 ↔ 중요하다					수행할 수 없다 ↔ 수행할 수 있다					아래 넷 중 하나 선택			
V. 생리적 통합 유지	73	호흡기능장애 대상자 간호	1	2	3	4	5	1	2	3	4	5	유지	분리	통합	삭제
	74	호흡증진 중재	1	2	3	4	5	1	2	3	4	5	유지	분리	통합	삭제
	75	호흡보조장치 관리	1	2	3	4	5	1	2	3	4	5	유지	분리	통합	삭제
	76	중심정맥관 관리	1	2	3	4	5	1	2	3	4	5	유지	분리	통합	삭제
	77	태아질식 증상과 징후 사정 및 간호	1	2	3	4	5	1	2	3	4	5	유지	분리	통합	삭제
	78	고위험 신생아 보육기 적용간호	1	2	3	4	5	1	2	3	4	5	유지	분리	통합	삭제
	79	호흡재활관리	1	2	3	4	5	1	2	3	4	5	유지	분리	통합	삭제
	80	심전도 관리	1	2	3	4	5	1	2	3	4	5	유지	분리	통합	삭제
	81	순환 보조장치 관리	1	2	3	4	5	1	2	3	4	5	유지	분리	통합	삭제
	82	동맥관 관리	1	2	3	4	5	1	2	3	4	5	유지	분리	통합	삭제
	83	조직 관류 장애 대상자 사정 및 간호	1	2	3	4	5	1	2	3	4	5	유지	분리	통합	삭제
	84	체액 전해질 불균형 사정 및 간호	1	2	3	4	5	1	2	3	4	5	유지	분리	통합	삭제
	85	활동 지속성 장애 대상자 사정 및 간호	1	2	3	4	5	1	2	3	4	5	유지	분리	통합	삭제
	86	정맥 순환증진 장치 간호	1	2	3	4	5	1	2	3	4	5	유지	분리	통합	삭제
	87	혈액 기능장애 대상자 간호	1	2	3	4	5	1	2	3	4	5	유지	분리	통합	삭제
	88	순환 기능장애 대상자 간호	1	2	3	4	5	1	2	3	4	5	유지	분리	통합	삭제
	89	심장 수술 후 간호	1	2	3	4	5	1	2	3	4	5	유지	분리	통합	삭제
	90	소화 기능장애 대상자 간호	1	2	3	4	5	1	2	3	4	5	유지	분리	통합	삭제
	91	배뇨장애를 가진 대상자 간호	1	2	3	4	5	1	2	3	4	5	유지	분리	통합	삭제
	92	당질 대사장애 대상자 간호	1	2	3	4	5	1	2	3	4	5	유지	분리	통합	삭제

영역	번호	아래의 간호사 직무관련 각 문항 별로 오른쪽 3가지 사항 모두에 ✔표 해 주십시오.	(1) 신규간호사 직무로 중요한 정도					(2) 신규간호사가 수행 할 수 있는 정도					(3) 신규간호사 직무 개선 요구			
			중요하지 않다 ↔ 중요하다					수행할 수 없다 ↔ 수행할 수 있다					아래 넷 중 하나 선택			
V. 생리적 통합유지	93	당뇨병 합병증 예방을 위한 간호	1	2	3	4	5	1	2	3	4	5	유지	분리	통합	삭제
	94	내분비계 장애 대상자 간호	1	2	3	4	5	1	2	3	4	5	유지	분리	통합	삭제
	95	생식기 질환/생식기 건강문제를 가진 대상자 간호	1	2	3	4	5	1	2	3	4	5	유지	분리	통합	삭제
	96	면역손상 대상자 사정 및 간호	1	2	3	4	5	1	2	3	4	5	유지	분리	통합	삭제
	97	감각기능 장애를 가진 대상자 사정 및 간호	1	2	3	4	5	1	2	3	4	5	유지	분리	통합	삭제
	98	두개내압 상승 환자의 간호	1	2	3	4	5	1	2	3	4	5	유지	분리	통합	삭제
	99	신경계 질환별 간호중재	1	2	3	4	5	1	2	3	4	5	유지	분리	통합	삭제
	100	운동기능 장애 간호중재	1	2	3	4	5	1	2	3	4	5	유지	분리	통합	삭제
	101	상처간호 수행 및 드레싱교환	1	2	3	4	5	1	2	3	4	5	유지	분리	통합	삭제
	102	화상환자 간호중재	1	2	3	4	5	1	2	3	4	5	유지	분리	통합	삭제
	103	피부질환 대상자의 간호중재	1	2	3	4	5	1	2	3	4	5	유지	분리	통합	삭제
	104	배액장치 관리	1	2	3	4	5	1	2	3	4	5	유지	분리	통합	삭제
	105	계통별 신생물 질환 대상자 간호	1	2	3	4	5	1	2	3	4	5	유지	분리	통합	삭제
	106	통증간호	1	2	3	4	5	1	2	3	4	5	유지	분리	통합	삭제
	107	재활간호서비스	1	2	3	4	5	1	2	3	4	5	유지	분리	통합	삭제
	108	(법정) 감염질환자의 전파예방 및 간호	1	2	3	4	5	1	2	3	4	5	유지	분리	통합	삭제
	V. 생리적 통합유지 : 추가 항목															

영역	번호	아래의 간호사 직무관련 각 문항 별로 오른쪽 3가지 사항 모두에 ✔표 해 주십시오.	(1) 신규간호사 직무로 중요한 정도					(2) 신규간호사가 수행 할 수 있는 정도					(3) 신규간호사 직무 개선 요구			
			중요하지 않다 ↔ 중요하다					수행할 수 없다 ↔ 수행할 수 있다					아래 넷 중 하나 선택			
Ⅵ. 약물 및 비경구 요법	109	투약의 적절성과 정확성	1	2	3	4	5	1	2	3	4	5	유지	분리	통합	삭제
	110	약물투여에 필요한 계산 시행	1	2	3	4	5	1	2	3	4	5	유지	분리	통합	삭제
	111	정맥주입 장치 관리	1	2	3	4	5	1	2	3	4	5	유지	분리	통합	삭제
	112	약품관리	1	2	3	4	5	1	2	3	4	5	유지	분리	통합	삭제
	113	말초정맥관 삽입, 유지, 제거	1	2	3	4	5	1	2	3	4	5	유지	분리	통합	삭제
	114	대상자에게 약물에 대해 교육	1	2	3	4	5	1	2	3	4	5	유지	분리	통합	삭제
	115	고위험 약품관리(마약관리)	1	2	3	4	5	1	2	3	4	5	유지	분리	통합	삭제
	Ⅵ. 약물 및 비경구 요법 : 추가 항목															

영역	번호	아래의 간호사 직무관련 각 문항 별로 오른쪽 3가지 사항 모두에 ✔표 해 주십시오.	(1) 신규간호사 직무로 중요한 정도					(2) 신규간호사가 수행 할 수 있는 정도					(3) 신규간호사 직무 개선 요구			
			중요하지 않다 ↔ 중요하다					수행할 수 없다 ↔ 수행할 수 있다					아래 넷 중 하나 선택			
Ⅶ. 심리사회적 통합유지	116	이상행동 사정 및 간호	1	2	3	4	5	1	2	3	4	5	유지	분리	통합	삭제
	117	폭력의 잠재성 평가 및 예방	1	2	3	4	5	1	2	3	4	5	유지	분리	통합	삭제
	118	학대 또는 방임 대상자를 확인하고 적절하게 중재	1	2	3	4	5	1	2	3	4	5	유지	분리	통합	삭제
	119	중독 사정 및 간호	1	2	3	4	5	1	2	3	4	5	유지	분리	통합	삭제
	120	정신사회건강문제 간호 및 교육	1	2	3	4	5	1	2	3	4	5	유지	분리	통합	삭제
	121	정신질환자 간호	1	2	3	4	5	1	2	3	4	5	유지	분리	통합	삭제
	122	아동 정신질환자 간호	1	2	3	4	5	1	2	3	4	5	유지	분리	통합	삭제
	123	임종간호와 교육	1	2	3	4	5	1	2	3	4	5	유지	분리	통합	삭제
	124	치료적 의사소통 기법	1	2	3	4	5	1	2	3	4	5	유지	분리	통합	삭제
	Ⅶ. 심리사회적 통합유지 : 추가 항목															

영역	번호	아래의 간호사 직무관련 각 문항 별로 오른쪽 3가지 사항 모두에 ✔표 해 주십시오.	(1) 신규간호사 직무로 중요한 정도					(2) 신규간호사가 수행 할 수 있는 정도					(3) 신규간호사 직무 개선 요구			
			중요하지 않다 ↔ 중요하다					수행할 수 없다 ↔ 수행할 수 있다					아래 넷 중 하나 선택			
Ⅷ. 건강증진 및 유지	125	(지역사회)건강교육 계획	1	2	3	4	5	1	2	3	4	5	유지	분리	통합	삭제
	126	건강증진 및 유지관리에 대한 정보(예방접종 등)제공	1	2	3	4	5	1	2	3	4	5	유지	분리	통합	삭제
	127	고위험 건강행위의 예방 및 치료에 대한 정보(금연, 안전한 성행위, 바늘교환)제공	1	2	3	4	5	1	2	3	4	5	유지	분리	통합	삭제
	128	성 건강증진 간호	1	2	3	4	5	1	2	3	4	5	유지	분리	통합	삭제
	129	문화간호	1	2	3	4	5	1	2	3	4	5	유지	분리	통합	삭제
	130	산업과 환경 간호	1	2	3	4	5	1	2	3	4	5	유지	분리	통합	삭제
	131	재난간호	1	2	3	4	5	1	2	3	4	5	유지	분리	통합	삭제
	132	치료계획을 결정하기 위해 가족역동성(family dynamics) (가족구조, 결속, 의사소통, 경계, 대처기전) 사정	1	2	3	4	5	1	2	3	4	5	유지	분리	통합	삭제
	133	가정환경에서 대상자를 관리할 수 있는 역량(장비, 지역사회 자원) 평가	1	2	3	4	5	1	2	3	4	5	유지	분리	통합	삭제
	134	건강위험요인 사정과 교육	1	2	3	4	5	1	2	3	4	5	유지	분리	통합	삭제
	Ⅷ. 건강유지 및 증진 : 추가 항목															

귀하께서 응답해 주신 자료는 간호사국가시험의 발전에 귀중한 자료가 될 것입니다. 설문에 응답해 주셔서 진심으로 감사드립니다.

【부록2】 전문가 타당도 평가 설문지

안녕하십니까?
바쁘신 중에 귀중한 시간을 내어 주셔서 고맙습니다.
대한간호협회 간호사국가시험위원회에서는 한국보건의료인국가시험원 2020년도 자유주제 위탁연구 과제 ‘간호사 국가시험의 통합 모형에 근거한 출제기준개발 기초연구’를 진행하고 있습니다. 출제기준을 개발하기 위해서는 최신의 간호사 직무분석이 선행되어야 하므로, 그동안 문헌고찰과 선행연구를 통해서 **8개 영역별로 개발된 134개의 신규간호사 실무항목**에 대해 전문가분들께 **타당도(적절성)**를 여쭙고자 합니다. **134개의 신규간호사 실무항목**에 대해서 **빠짐없이** 응답해 주시기를 당부드립니다. **2021년 3월 2일(화)까지 응답**해 주셔서 기간 내에 전문가 타당도 평가 설문이 회수될 수 있도록 협조해 주시기 바랍니다. 바쁘신 중에도 설문에 성실히 응해 주심에 감사드립니다.

설문의 목적을 이해하고 참여에 동의합니다. □

2021. 2.22. 책임연구자 김경희
공동연구자 강소영 강윤희 권영란 김현정 송영신 조주연 최미영

1. 귀하가 근무하는 기관은 다음 중 어디에 해당합니까?

① 상급종합병원 ② 종합병원 ③ 학교(보건교사) ④ 보건소 ⑤ 기타

2. 귀하의 간호사로서의 근무기간은 다음 중 어디에 해당합니까?

① 1년 미만 ② 1 ~ 3년 미만 ③ 3 ~ 5년 미만 ④ 5 ~ 7년 미만 ⑤ 7년 이상

3. 귀하가 현재 근무하고 있는 부서는 다음 중 어디에 해당합니까?

① 내 · 외과 ② 분만실, 산부인과 ③ 소아청소년과 ④ 정신과 ⑤ 학교 또는 보건소

▪ 다음은 8개 영역별로 개발 된 134개 신규간호사 실무항목 입니다. 각 실무항목의 적절성을 아래에 ✔표 하여 주시고, 수정 의견이 있으시면 기술해주시기 바랍니다.

영역	번호	신규간호사 실무항목	실무항목 적절성<br>1. 매우 타당하지 않음<br>2. 타당하지 않음<br>3. 타당함<br>4. 매우 타당함				수정의견
I. 간호관리와 전문성 향상	1	인수인계 시행	1	2	3	4	
	2	법적 실무범위 내에서 간호 수행	1	2	3	4	
	3	기록시 표준화된 약어 사용	1	2	3	4	
	4	지침에 따라 간호기록	1	2	3	4	
	5	입원, 전동, 퇴원	1	2	3	4	
	6	장비를 적절하고 안전하게 사용	1	2	3	4	
	7	간호단위 물품교환체계에 따른 물품관리	1	2	3	4	
	8	질 향상(QI) 활동에 참여	1	2	3	4	
	9	간호사업 평가 관련 업무수행 (도구개발, 자료조사, 분석, 비교 및 사업개선)	1	2	3	4	
	10	간호전문직 윤리 준수와 역할	1	2	3	4	
	11	대상자의 개인정보 및 사생활 보호	1	2	3	4	
	12	대상자에게 치료 및 절차에 따라 적절한 설명을 하고 동의를 획득 하였는지 확인	1	2	3	4	
	13	환자의 권리와 책임에 관하여 대상자에게 교육 제공	1	2	3	4	

	14	억제대 사용시 법적 및 윤리적 간호	1	2	3	4	
	15	일차의료기반의 보건간호	1	2	3	4	
	16	지역사회 간호사업의 법적 기준 및 지침에 따른 활동 참여	1	2	3	4	
	17	사례관리활동 참여	1	2	3	4	
	18	업무를 조직화하여 일을 효율적으로 관리	1	2	3	4	
	19	환자분류체계 관련 정보수집 및 활용	1	2	3	4	
	20	간호대상에 적합한 다양한 자원 및 매체선택	1	2	3	4	
	21	취약가족에 필요한 지역사회 자원활용	1	2	3	4	
	22	전문직간 협업	1	2	3	4	

영역	번호	신규간호사 실무항목	실무항목 적절성<br>1. 매우 타당하지 않음<br>2. 타당하지 않음<br>3. 타당함<br>4. 매우 타당함				수정의견
II. 안전과 감염관리	23	안전한 환경 제공	1	2	3	4	
	24	감염관리	1	2	3	4	
	25	위험물질과 유해물질관리	1	2	3	4	

영역	번호	신규간호사 실무항목	실무항목 적절성<br>1. 매우 타당하지 않음<br>2. 타당하지 않음<br>3. 타당함<br>4. 매우 타당함				수정의견
Ⅲ. 위험요인 사정	26	간호계획, 진료지침 수행	1	2	3	4	
	27	대상자의 건강문제에 대해 우선순위 결정	1	2	3	4	
	28	활력징후 사정	1	2	3	4	

Ⅲ. 위험요인 사정	29	신생아간호	1	2	3	4	
	30	영아간호	1	2	3	4	
	31	유아간호	1	2	3	4	
	32	학령전기간호	1	2	3	4	
	33	학령기간호	1	2	3	4	
	34	청소년간호	1	2	3	4	
	35	폐경기 여성간호	1	2	3	4	
	36	노인간호	1	2	3	4	
	37	성건강간호	1	2	3	4	
	38	건강력 사정	1	2	3	4	
	39	신체검진 수행 및 결과해석	1	2	3	4	
	40	생식기 건강사정	1	2	3	4	
	41	태아 건강사정 및 간호	1	2	3	4	
	42	신생아 건강사정 및 간호	1	2	3	4	
	43	고위험신생아 건강사정 및 간호	1	2	3	4	
	44	심폐소생술 간호	1	2	3	4	
	45	응급간호	1	2	3	4	
	46	진단검사간호	1	2	3	4	
	47	수술간호	1	2	3	4	
	48	산전간호와 교육	1	2	3	4	
	49	분만 중 간호와 교육	1	2	3	4	
	50	산후관리와 교육	1	2	3	4	
	51	고위험임부 간호	1	2	3	4	
	52	고위험산부간호	1	2	3	4	
	53	고위험산모간호	1	2	3	4	

영역	번호	신규간호사 실무항목	실무항목 적절성<br>1. 매우 타당하지 않음<br>2. 타당하지 않음<br>3. 타당함<br>4. 매우 타당함				수정의견
Ⅳ. 기본간호	54	체온유지간호	1	2	3	4	
	55	세척(irrigation)(방광, 귀, 눈) 수행	1	2	3	4	
	56	개인위생간호	1	2	3	4	
	57	섭취 및 배설량 사정과 간호	1	2	3	4	
	58	영양사정 및 관리	1	2	3	4	
	59	질환별 영양문제의 사정 및 관리	1	2	3	4	
	60	섭취장애 대상자 사정 및 간호	1	2	3	4	
	61	배뇨장애 대상자 사정 및 간호	1	2	3	4	
	62	투석대상자 관리	1	2	3	4	
	63	요루 및 장루 관리	1	2	3	4	
	64	기관절개부 관리	1	2	3	4	
	65	배변관리	1	2	3	4	
	66	수면과 휴식 간호	1	2	3	4	
	67	피부통합성 사정 및 간호	1	2	3	4	
	68	이동간호	1	2	3	4	
	69	활동과 자기돌봄장애 사정 및 간호	1	2	3	4	
	70	외과적 장치 관리	1	2	3	4	
	71	척추손상 및 질환대상자의 간호	1	2	3	4	

	72	관절대치술 환자간호	1	2	3	4	
영역	번호	신규간호사 실무항목	실무항목 적절성<br>1. 매우 타당하지 않음<br>2. 타당하지 않음<br>3. 타당함<br>4. 매우 타당함				수정의견
	73	호흡기능장애 대상자 간호	1	2	3	4	
	74	호흡증진중재	1	2	3	4	
	75	호흡보조장치 관리	1	2	3	4	
	76	중심정맥관 관리	1	2	3	4	
	77	태아질식 증상과 징후 사정 및 간호	1	2	3	4	
	78	고위험신생아 보육기 적용간호	1	2	3	4	
	79	호흡재활관리	1	2	3	4	
	80	심전도관리	1	2	3	4	
	81	순환보조장치관리	1	2	3	4	
	82	동맥관관리	1	2	3	4	
	83	조직관류장애 대상자 사정 및 간호	1	2	3	4	

V. 생리적 통합유지	84	체액전해질 불균형 사정 및 간호	1	2	3	4	
	85	활동지속성 장애 대상자 사정 및 간호	1	2	3	4	
	86	정맥순환증진장치 간호	1	2	3	4	
	87	혈액기능장애 대상자 간호	1	2	3	4	
	88	순환기능장애 대상자간호	1	2	3	4	
	89	심장수술후 간호	1	2	3	4	
	90	소화기능장애 대상자 간호	1	2	3	4	
	91	배뇨장애를 가진 대상자 간호	1	2	3	4	
	92	당질대사장애 대상자 간호	1	2	3	4	
	93	당뇨병 합병증 예방을 위한 간호	1	2	3	4	
	94	내분비계장애 대상자 간호	1	2	3	4	
	95	생식기 질환/생식기 건강문제를 가진 대상자 간호	1	2	3	4	
	96	면역손상 대상자 사정 및 간호	1	2	3	4	
	97	감각기능장애를 가진 대상자 사정 및 간호	1	2	3	4	
	98	두개내압 상승 환자의 간호	1	2	3	4	

	99	신경계 질환별 간호중재	1	2	3	4	
	100	운동기능장애 간호중재	1	2	3	4	
	101	상처간호 수행 및 드레싱 교환	1	2	3	4	
	102	화상환자 간호중재	1	2	3	4	
	103	피부질환 대상자의 간호중재	1	2	3	4	
	104	배액장치관리	1	2	3	4	
	105	계통별 신생물 질환 대상자의 간호	1	2	3	4	
	106	통증간호	1	2	3	4	
	107	재활간호서비스	1	2	3	4	
	108	(법정) 감염질환자의 전파예방 및 간호	1	2	3	4	

영역	번호	신규간호사 실무항목	실무항목 적절성<br>1. 매우 타당하지 않음<br>2. 타당하지 않음<br>3. 타당함<br>4. 매우 타당함				수정의견
Ⅵ. 약물 및 비경구요법	109	투약의 적절성과 정확성	1	2	3	4	
	110	약물 투여에 필요한 계산 시행	1	2	3	4	
	111	정맥주입장치 관리	1	2	3	4	
	112	약품관리	1	2	3	4	
	113	말초정맥관 삽입, 유지, 제거	1	2	3	4	
	114	대상자에게 약물에 대해 교육	1	2	3	4	
	115	고위험 약품관리(마약관리)	1	2	3	4	

영역	번호	신규간호사 실무항목	실무항목 적절성<br>1. 매우 타당하지 않음<br>2. 타당하지 않음<br>3. 타당함<br>4. 매우 타당함				수정의견
VII. 심리사회적 통합유지	116	이상행동 사정 및 간호	1	2	3	4	
	117	폭력의 잠재성 평가 및 예방	1	2	3	4	
	118	학대 또는 방임 대상자를 확인하고 적절하게 중재	1	2	3	4	
	119	중독 사정 및 간호	1	2	3	4	
	120	정신사회건강문제 간호 및 교육	1	2	3	4	
	121	정신질환자 간호	1	2	3	4	
	122	아동정신질환자 간호	1	2	3	4	
	123	임종간호와 교육	1	2	3	4	
	124	치료적 의사소통 기법	1	2	3	4	

영역	번호	신규간호사 실무항목	실무항목 적절성<br>1. 매우 타당하지 않음<br>2. 타당하지 않음<br>3. 타당함<br>4. 매우 타당함				수정의견
VIII.	125	(지역사회)건강교육계획	1	2	3	4	

건강증진 및 유지	126	건강증진 및 유지관리에 대한 정보(예방접종 등) 제공	1	2	3	4	
	127	고위험 건강 행위의 예방 및 치료에 대한 정보(금연, 안전한 성 행위, 바늘교환) 제공	1	2	3	4	
	128	성 건강증진 간호	1	2	3	4	
	129	문화간호	1	2	3	4	
	130	산업과 환경 간호	1	2	3	4	
	131	재난간호	1	2	3	4	
	132	치료계획을 결정하기 위해 가족 역동성(family dynamics)(가족 구조, 결속, 의사소통, 경계, 대처기전) 사정	1	2	3	4	
	133	가정환경에서 대상자를 관리할 수 있는 역량(장비, 지역사회 자원) 평가	1	2	3	4	
	134	건강위험요인 사정과 교육	1	2	3	4	

귀하께서 응답해 주신 자료는 간호사국가시험의 발전에 귀중한 자료가 될 것입니다. 설문에 응답해 주셔서 진심으로 감사드립니다.

【부록3】간호사 국가시험 출제모형에 대한 한국간호과학회 7개 회원학회 1, 2 차 의견

대분류	번호	신규간호사 직무항목	지식항목	1차 성인(141)	2차 성인(139)	1,2차 관리(75)	1차 모성(65)	2차 모성(72)	1차 기본(86)	2차 기본(87)	1차 정신(96)	2차 정신(93)	1차 지역(113)	2차 지역(127)	2차 아동(205)
I. 간호관리와 전문성 향상	1	인수인계시행	업무인수인계			관리									
	2	법적 실무범위 내에서 간호 수행	간호표준실무, 간호관리표준실무			관리							지역	지역	
	3	기록시 표준화된 약어 사용	간호기록 시 표준약어			관리									
	4	지침에 따라 간호기록	간호기록지침			관리									
			환자개인정보보호			관리		모성			정신	정신	지역	지역	
			간호정보시스템			관리									
	5	입원, 전동, 퇴원	입원관리, 퇴원관리 및 교육, 전과전동업무			관리									
			입퇴원, 전과전동시 인수인계			관리									
			고위험 환자관리체계			관리									
	6	장비를 적절하고 안전하게 사용	물품관리			관리							지역	지역	
	7	간호단위 물품교환체계에 따른 물품관리	약품관리			관리							지역	지역	
			마약 및 고위험 약품관리			관리					정신	정신		지역	
	8	질 향상(QI) 활동에 참여	의료의 질관리, 간호의 질관리 및 질향상활동			관리							—	지역	
			질관리 전략 및 절차			관리							—	지역	
			의료기관인증제도 및 인증기준			관리							—		
			질관리 평가기준 및 도구			관리							—	지역	
	9	간호사업 평가 관련 업무수행(도구개발, 자료조사, 분석, 비교 및 사업개선)	간호사업평가										지역	지역	
			간호관리 통제기능			관리									

대분류	번호	신규간호사 직무항목	지식항목	1차 성인 (141)	2차 성인 (139)	1,2차 관리 (75)	1차 모성 (65)	2차 모성 (72)	1차 기본 (86)	2차 기본 (87)	1차 정신 (96)	2차 정신 (93)	1차 지역 (113)	2차 지역 (127)	2차 아동 (205)
I. 간호관리와 전문성 향상	10	간호전문직 윤리 준수와 역할	간호윤리, 생명의료윤리 원칙, 윤리 이론 및 윤리적 사고			관리									
			한국간호사 윤리강령			관리									
			연구윤리			관리									
			전문직 협력관련 간호윤리			관리							지역	지역	
			자원분배 관련 간호윤리			관리							지역	지역	
			간호전문직관			관리									
			간호사의 임무와 역할			관리							지역	지역	
			소수집단 인권보호 및 윤리적 간호			관리					정신	정신	지역	지역	아동
			정신건강복지법								정신	정신			
			공중보건관리 법률										지역	지역	
	11	대상자의 개인정보 및 사생활 보호	대상자의 개인정보 빛 사생화 보호권리(사생활, 인격권, 정보보호권, 평등권)			관리					정신	정신	지역	지역	
			대상자의 법적 권리 보호방안			관리							지역	지역	
			공중보건상 재난시 자료의 비밀보장										지역	지역	
	12	대상자에게 치료 및 절차에 따라 적절한 설명을 하고 동의를 획득하였는지 확인	간호사의 법적 의무			관리		모성					지역	지역	
	13	환자의 권리와 책임에 관하여 대상자에게 교육 제공	간호와 치료에 관한 환자권리 및 책임			관리							지역	지역	
			간호사의 법적 의무와 책임			관리							지역	지역	
			환자권리존중 및 보호			관리					정신	정신	지역	지역	아동
	14	억제대 사용시 법적 및 윤리적 간호	신체억제대 사용지침, 억제대 사용관련 윤리적 사고 및 결정			관리					정신	정신			

대분류	번호	신규간호사 직무항목	지식항목	1차 성인 (141)	2차 성인 (139)	1,2차 관리 (75)	1차 모성 (65)	2차 모성 (72)	1차 기본 (86)	2차 기본 (87)	1차 정신 (96)	2차 정신 (93)	1차 지역 (113)	2차 지역 (127)	2차 아동 (205)
I. 간호관리와 전문성 향상	15	일차의료기반의 보건간호	일차보건의료										지역	지역	
			지역사회간호의 정의										지역	지역	
			지역사회와 건강개념										지역	지역	
			건강의 결정요인										지역	지역	
			국제보건기구의 간호정책 및 활동										지역	지역	
			보건의료체계의 이해										지역	지역	
			진료비 지불보상제도			관리							지역	지역	
	16	지역사회 간호사업의 법적 기준 및 지침에 따른 활동 참여	보건관리자의 역할 및 업무										지역	지역	
			산업전문간호사의 역할										지역	지역	
			보건/간호사업기획 및 수행										지역	지역	
			보건교육자, 보건진료원의 역할										지역	지역	
			보건정보 및 기술활용										지역	지역	
			건강증진 종합계획										지역	지역	
			간호사업의 법적 기준 및 지침										지역	지역	
	17	사례관리활동 참여	사례관리 정의 및 원칙			관리							지역	지역	
			조정과 협력			관리							지역	지역	
			간호관리체계 및 과정			관리							지역	지역	
			사례관리모델 및 사례, 사례관리과정			관리							지역	지역	

대분류	번호	신규간호사 직무항목	지식항목	1차	2차	1,2차	1차	2차	1차	2차	1차	2차	1차	2차	2차
				성인 (141)	성인 (139)	관리 (75)	모성 (65)	모성 (72)	기본 (86)	기본 (87)	정신 (96)	정신 (93)	지역 (113)	지역 (127)	아동 (205)
I. 간호관리와 전문성 향상	18	업무를 조직화하여 일을 효율적으로 관리	간호관리업무, 역량			관리							지역		
			관리이론			관리									
			기획 원칙 및 구성요소, 기획단계			관리									
			계획안과 목표관리			관리									
			의사결정			관리									
			예산관리			관리									
			조직화원리, 조직구조 및 권한			관리									
			직무설계, 직무분석, 직무평가			관리									
			간호전달체계			관리									
			간호조직문화와 조직변화			관리									
			간호인적자원관리			관리									
	19	환자분류체계 관련 정보수집 및 활용	환자분류체계			관리							지역	지역	
			간호업무량 및 인력산정, 간호업무배정			관리							지역	지역	
	20	간호대상에 적합한 다양한 자원 및 매체선택	간호서비스마케팅			관리							지역	지역	
	21	취약가족에 필요한 지역사회 자원활용	취약가족과 간호, 가족의 개념					모성			정신	정신	지역	지역	아동
			가족생활주기의 특성										지역	지역	아동
			가족관련이론										지역	지역	
			가족간호과정										지역	지역	
	22	전문직간 협업	조정과 협력			관리							—	지역	
			리더십			관리							—	지역	
			동기부여이론 및 동기부여 적용			관리							—	지역	
			임파워먼트			관리							—	지역	
			효과적인 의사소통			관리					정신	정신	—	지역	
			주장행동			관리							—	지역	
			갈등관리, 직무스트레스 관리			관리							—	지역	

대분류	번호	신규간호사 직무항목	지식항목	1차 성인(141)	2차 성인(139)	1,2차 관리(75)	1차 모성(65)	2차 모성(72)	1차 기본(86)	2차 기본(87)	1차 정신(96)	2차 정신(93)	1차 지역(113)	2차 지역(127)	2차 아동(205)
II. 안전과 감염관리	23	안전한 환경 제공	사생활유지관리			관리					정신	정신	지역	지역	
			환경관리 및 환자안전관리			관리			기본	기본	정신	정신	지역	지역	
			환자안전사고예방(관련법포함)			관리					정신	정신	지역	지역	
			환자안전 사고보고체계 및 사고기록			관리					정신	정신	지역	지역	
			환자안전문제발생 시 대응방안 및 운영체계			관리					정신	정신	지역	지역	
			조직의 환자안전문화			관리					정신	정신			
			보안계획(신생아실 안전, 폭력, 통제된 접근) 및 절차 준수			관리									
			안전사고 유형, 위험요인과 예방			관리			기본	기본	정신	정신	지역	지역	아동
			낙상예방 및 관리			관리			기본	기본	정신	정신	지역	지역	아동
			안전사고시 기록			관리					정신	정신	지역	지역	
			신체보호대 적응증, 신체보호대 종류, 신체보호대 적용 시 주의점, 적용방법						기본	기본	정신	정신			

대분류	번호	신규간호사 직무항목	지식항목	1차 성인 (141)	2차 성인 (139)	1,2차 관리 (75)	1차 모성 (65)	2차 모성 (72)	1차 기본 (86)	2차 기본 (87)	1차 정신 (96)	2차 정신 (93)	1차 지역 (113)	2차 지역 (127)	2차 아동 (205)
II. 안전과 감염관리	24	감염관리	병원감염관리			관리			기본	기본	정신	정신			
			감염관리지침			관리			기본	기본			지역	지역	
			무균술의 기본원리						기본	기본					
			소독방법 / 소독수준, 소독방법, 소독제의 종류와 용도						기본	기본			지역	지역	
			멸균방법 / 멸균준비, 멸균방법과 적용						기본	기본					
			무균술의 원리, 상처감염예방법						기본	기본					
			외과적무균술 정의, 외과적 무균술의 기본원리와 적용						기본	기본					
			표준주의지침			관리			기본	기본			지역	지역	
			감염위험요인사정 / 감염유형, 감염회로, 감염에 대한 신체방어, 감염 감수성에 영향을 미치는 요인	성인	성인				기본	기본			지역	지역	
			의료관련감염 정의, 종류, 위험요인, 교차감염						기본	기본					
			병원감염(요로감염, 폐렴, 수술창상감염, 혈액감염 등) 관리 및 결과 평가	성인	성인	관리			기본	기본					
			전파경로별 주의지침(공기주의, 접촉주의, 비말주의), 다제내성균 감염관리, 면역저하 대상자 관리(역격리), 보호장구 착용과 벗기			관리			기본	기본			지역	지역	
			의료폐기물 종류별 관리 및 주의점			관리									
	25	위험물질과 유해물질관리	고위험약품 및 마약관리			관리					정신	정신			
III.	26	간호계획, 진료지침 수행	간호과정	성인	성인		모성	모성	기본	기본	정신	정신	지역	지역	아동
	27	대상자의 건강문제에 대해 우선순위 결정	건강문제의 우선순위 결정	성인	성인		모성	모성			정신	정신	지역	지역	아동

대분류	번호	신규간호사 직무항목	지식항목	1차 성인 (141)	2차 성인 (139)	1,2차 관리 (75)	1차 모성 (65)	2차 모성 (72)	1차 기본 (86)	2차 기본 (87)	1차 정신 (96)	2차 정신 (93)	1차 지역 (113)	2차 지역 (127)	2차 아동 (205)
III. 위험요인 사정	28	활력징후 사정	활력징후의 중요성과 영향요인, 각 활력징후 정상범위, 측정법, 의미와 해석	성인					기본	기본	정신	정신	—	지역	아동
			말초산소포화도에 영향을 미치는 요인, 측정방법 및 주의점, 정상범위, 의미와 해석	성인	성인				기본	기본					아동
	29	신생아간호	청결한 기도유지, 고무흡인기의 적절한 사용, 호흡측정 및 호흡양상 관찰					모성							아동
			체온유지간호					모성							아동
			신생아목욕간호, 제대간호, 기저귀발진 예방,					모성							아동
			신생아의 영양, 모유수유/인공수유 방법, 신생아 수유행동, 체중측정					모성							아동
			감염과 상해로 부터 보호, 예방접종												아동
			애착행동사정, 부모-신생아 애착증진 간호					모성							아동
			신생아 퇴원기준목록, 신생아 돌봄지도, 수면과 활동, 신생아 선별검사와 예방접종												아동
	30	영아간호	고형식이의 선택과 준비, 고형식이의 시작, 이유, 스스로 먹기, 음식 알레르기												아동
			치아발달, 치아건강관리, 저충치식이, 유아기 우식증 예방												아동
			낯가림, 분리불안 대처 방안												아동
			영아산통의 증상, 치료관리, 간호중재												아동
			영아돌연사증후군 원인, 위험요인, 보호요소, 간호중재												아동
	31	유아간호	대소변가리기 훈련 준비, 방법, 단계												아동
			분노발작 대처 방안												아동
			거부증 대처 방안								정신	정신			아동
			퇴행 대처 방안								정신	정신			아동

대분류	번호	신규간호사 직무항목	지식항목	1차 성인 (141)	2차 성인 (139)	1,2차 관리 (75)	1차 모성 (65)	2차 모성 (72)	1차 기본 (86)	2차 기본 (87)	1차 정신 (96)	2차 정신 (93)	1차 지역 (113)	2차 지역 (127)	2차 아동 (205)
III. 위험요인 사정	32	학령전기간호	언어문제 예방과 조기발견, 말더듬 관리								정신	정신			아동
			학령전기아동의 공포특성, 극복방안								정신	정신			아동
			악몽과 야경증 대처방안								정신	정신			아동
	33	학령기간호	학령기 아동의 사회적 관계, 또래집단의 정체성과 협력, 부적절한 또래와의 관계									정신			아동
			학교공포증 대처방안								정신	정신			아동
			성장통 관리												아동
			영양, 수면과 휴식, 운동과 활동, 치아건강, 안전한 습관, 사고예방									정신			아동
	34	청소년간호	사춘기 여성 건강사정 / 2차성징, 테너단계, 여아와 남아의 성적 성숙				모성	모성							아동
			초경				모성	모성							아동
			사춘기의 신체, 심리적 발달 특성				모성	모성							아동
			건강에 대한 관점, 청소년기의 위험행동(중독, 자살, 학교폭력, 섭식장애) 간호관리								정신	정신			아동
	35	폐경기 여성간호	완(폐)경 여성 건강사정과 간호				모성	모성							
	36	노인간호	노화에 따른 생리적변화	성인	성인								—	지역	
			노인 대상자 건강교육	성인	성인								—	지역	
	37	성건강간호	월경전 증후군 간호				모성	모성							
	38	건강력사정	건강력 사정을 위한 문진요소	성인	성인						정신	정신			아동
			기관별 review of system	성인	성인										아동
	39	신체검진 수행 및 결과해석	초점 사정(focused assessment) 수행	성인	성인										아동
			기관별 신체검진 (두경부, 흉부, 복부, 근골격계, 호흡기계, 심혈관계, 신경계, 생식기계)	성인	성인										아동

대분류	번호	신규간호사 직무항목	지식항목	1차 성인 (141)	2차 성인 (139)	1,2차 관리 (75)	1차 모성 (65)	2차 모성 (72)	1차 기본 (86)	2차 기본 (87)	1차 정신 (96)	2차 정신 (93)	1차 지역 (113)	2차 지역 (127)	2차 아동 (205)
III. 위험요인 사정	40	생식기 건강사정	여성생식기 사정				모성	모성							
			생식기 검진 전후 간호(질경검진, 자궁경부세포검사, 세포도말검사)				모성	모성							
			유방건강사정				모성	모성							
	41	태아건강사정 및 간호	태아건강사정(태아추음파, 태동측정, 신체계측, 생물리학적 계수, 모체혈청검사, 태아전자감시, 양수천차, 융모막생검)				모성	모성							
	42	신생아 건강사정 및 간호	출생시 신생아 평가-심박동수, 호흡노력, 근력, 자극에 대한 반응, 피부색				모성	모성							아동
			머리둘레, 가슴둘레, 머리엉덩길이, 복부둘레, 머리발꿈치 길이, 체중				모성	모성							아동
			신생아 신체사정을 위한 환경, 방법과 특징적 소견, 신생아 반사 사정												아동
			신생아 선별검사 종류, 방법 및 시기												아동
			신생아의 울음 및 수면의 특성, 여아의 마유,혈액성분비물, 남아의 통증성 지속성 발기												아동
			생리적 황달원인, 기간, 관리												아동
	43	고위험신생아 건강사정 및 간호	고위험 신생아의 생리적 기능과 특성, 신체사정												아동
			고위험 신생아의 체온, 호흡유지, 영양유지, 피부간호												아동
			신생아 통증조절, 광선요법, 교환수혈 시 간호												아동
			출생시 손상 신생아 간호												아동
			미숙아와 과숙아의 특성												아동
			고위험(용혈성질환, 패혈증, 근골격계장애, 소화기계 장애, 유전질환, 대사질환)간호												아동
			당뇨병, 중독, 모체요인과 관련된 감염 산모의 신생아 간호												아동

대분류	번호	신규간호사 직무항목	지식항목	1차 성인 (141)	2차 성인 (139)	1,2차 관리 (75)	1차 모성 (65)	2차 모성 (72)	1차 기본 (86)	2차 기본 (87)	1차 정신 (96)	2차 정신 (93)	1차 지역 (113)	2차 지역 (127)	2차 아동 (205)
III. 위험요인 사정	44	심폐소생술 간호	심정지 환자의 심폐소생술, 세동제거술	성인	성인										
	45	응급간호	응급의료전달체계, 응급간호의 원칙, 응급환자 분류기준	성인	성인								—	지역	
			외상의 유형에 따른 간호, 출혈시 간호, 쇼크의 유형, 쇼크의 유형에 따른 간호	성인	성인										
			중독의 유형, 중독의 유형에 따른 간호	성인	성인										
	46	진단검사간호	진단검사 수행, 진단검사결과 확인, 진단검사 전후 간호	성인	성인		모성	모성							
			침습적 시술(중심정맥관, 흉강천자, 기관지내시경)	성인	성인										
			침습적 검사방법(양수천자, 경피제대혈채취, 융모막융모생검) 검사 전후 간호				모성	모성							
			생식기 검진 전후 간호(질경검진, 자궁경부세포검사, 세포도말검사)				모성	모성							
	47	수술간호	수술동의서, 수술 전/중.후 간호	성인	성인		모성	모성							
			여성 생식기 수술 간호				모성	모성							
			유방절제술 환자간호	성인	성인										
			(중증도 진정(moderate sedation) 시술 중 또는 후에 대상자 관리)	성인	성인										
			마취의 종류 및 방법, 합병증	성인	성인		모성	모성							
			국소마취의 종류(척추마취, 경막외 마취)에 따른 간호과정	성인	성인		모성	모성							
	48	산전간호와 교육	임부의 생리적 변화				모성	모성							
			임부의 건강사정과 산전간호				모성	모성							
			임부간호(임신주기별 간호)				모성	모성							
			임부와 가족의 사회심리적 적응과 간호				모성	모성							

대분류	번호	신규간호사 직무항목	지식항목	1차 성인(141)	2차 성인(139)	1,2차 관리(75)	1차 모성(65)	2차 모성(72)	1차 기본(86)	2차 기본(87)	1차 정신(96)	2차 정신(93)	1차 지역(113)	2차 지역(127)	2차 아동(205)
III. 위험요인 사정	49	분만중 간호와 교육	분만관련 주요개념(분만요소, 산도, 골반경선, 아두경선, 태세, 태향, 태위, 선진부)				모성	모성							
			분만생리(분만과정, 분만기전, 자궁수축, 분만전구증상)				모성	모성							
			통증완화간호(통증사정, 비약물적방법, 약물적 방법)				모성	모성							
			산부 간호(1기, 2기, 3기, 4기 간호, 상호작용증진)				모성	모성							
	50	산후관리와 교육	산후생리적 변화				모성	모성							
			산모와 가족의 사회심리적 적응과 간호				모성	모성							
			사회심리적 변화, 모성역할, 애착증진간호				모성	모성							
			산모 간호(산후건강사정과 건강관리)				모성	모성							
			모유수유 간호				모성	모성							
	51	고위험 임부간호	고위험 임신요인 사정				모성	모성							
			출혈성 임신 건강문제가 있는 임부 간호(유산, 자궁경관무력증, 자궁외임신, 포상기태, 전치태반, 태반조기박리)				모성	모성							
			임신성고혈압 임부 간호				모성	모성							
			내과적 임신 건강문제가 있는 임부 간호(임신성 당뇨, 갑상선기능장애, 심장질환, 빈혈, 성전파성질환, 비뇨기질환, TORCH 감염)				모성	모성							
			분만과정 관련 건강문제가 있는 산부 간호 (난산, 급속분만, 지연분만, 조기진통, 자궁파열, 자궁내번증, 태반유착, 다태분만, 과숙아분만, 조기분만)				모성	모성							

대분류	번호	신규간호사 직무항목	지식항목	1차 성인(141)	2차 성인(139)	1,2차 관리(75)	1차 모성(65)	2차 모성(72)	1차 기본(86)	2차 기본(87)	1차 정신(96)	2차 정신(93)	1차 지역(113)	2차 지역(127)	2차 아동(205)
III. 위험요인 사정	52	고위험 산부간호	태아부속물 관련 건강문제가 있는 산부간호(양수과다증, 양수과소증, 조기파막,제대탈출)				모성	모성							
			대안적분만 간호(유도분만, 흡입분만, 제왕절개분만)				모성	모성							
	53	고위험 산모간호	산후감염 산모 간호(산욕감염, 자궁내막염, 폐색전증, 유방염)				모성	모성							
			산후출혈 산모 간호(산후출혈, 자궁이완, 산도열상, 산후혈종)				모성	모성							
			산후우울 산모 간호				모성	모성							
IV. 기본간호	54	체온유지간호	간호력, 체온측정(활력징후에 포함), 신체검진(피부상태 조사), 임상검사(혈액배양검사), 영향요인(활력징후에 포함)						기본	기본					아동
			체온유지 간호진단						기본	기본					아동
			체온조절 문제 간호계획, 발열단계에 따른 간호, 고체온 및 저체온 대상자 간호						기본	기본					아동
			체온유지 간호결과 평가						기본	기본					아동
			열냉요법 적응증, 금기증, 적용-건열, 습열, 건냉, 습냉						기본	기본					
	55	세척(irrigation)(방광, 귀, 눈) 수행	세척(irrigation)(방광, 귀, 눈) 수행												
	56	개인위생간호	구강간호,등간호,침상세발,회음부간호						기본	기본	정신	정신			
	57	섭취 및 배설량 사정과 간호	섭취량과 배설량 측정방법, 결과해석						기본	기본					

대분류	번호	신규간호사 직무항목	지식항목	1차	2차	1,2 차	1차	2차	1차	2차	1차	2차	1차	2차	2차
				성인 (141)	성인 (139)	관리 (75)	모성 (65)	모성 (72)	기본 (86)	기본 (87)	정신 (96)	정신 (93)	지역 (113)	지역 (127)	아동 (205)
IV. 기본간호	58	영양사정 및 관리	경장영양 종류, 적응증, 합병증						기본	기본					
			영양상태지표, 영양 및 대사기능의 특징						기본	기본					아동
			소화기능 사정(병력, 신체사정, 진단검사)	성인	성인										아동
			영양과잉, 영양결핍						기본	기본	정신				아동
			흡인, 설사, 변비, 비만, 과체중, 영양부족	성인	성인				기본	기본	정신				아동
			영양요구 간호계획, 치료식이(일반 치료식이, 특별 치료식이), 금식, 식욕촉진, 식사돕기						기본	기본	정신				아동
			영양간호 결과 평가						기본	기본	정신				아동
			비경장영양 종류, 적응증, 합병증						기본	기본					아동
			중심 비경구영양, 말초 비경구영양, 완전 비경구영양						기본	기본					아동
			비경구영양(TPN)주입 및 대상자의 반응 평가						기본	기본					아동
			지속적, 간헐적 위관 영양 제공						기본	기본					아동
			경장영양간호 결과평가						기본	기본					아동
			췌장염, 담낭염,항암화학요법, PCA, 장폐색, 메니에르병, 두개내압상승, 대사성 알칼리증	성인	성인										
	59	질환별 영양문제의 사정 및 관리	건강문제 별 영양 불균형 특성<br>핵심질환:식도출혈,위장관출혈,위루술,간이식,장루간호,비장관,담관관리	성인	성인										아동
			영양장애 아동간호												아동
			비만 아동간호: 식이상담, 행동요법, 집단 및 가족 참여, 신체활동, 예방								정신	정신			아동
			일반치료식이, 특별치료식이	성인	성인					기본					
	60	섭취장애 대상자 사정 및 간호	연하장애 간호	성인	성인										아동

대분류	번호	신규간호사 직무항목	지식항목	1차<br>성인(141)	2차<br>성인(139)	1,2차<br>관리(75)	1차<br>모성(65)	2차<br>모성(72)	1차<br>기본(86)	2차<br>기본(87)	1차<br>정신(96)	2차<br>정신(93)	1차<br>지역(113)	2차<br>지역(127)	2차<br>아동(205)
Ⅳ. 기본간호	61	배뇨장애 대상자 사정 및 간호	간호력, 배뇨 영향요인, 배뇨양상, 검사물 채취, 신체검진, 진단검사	성인	성인				기본	기본					아동
			배뇨장애와 관련된 간호진단	성인	성인				기본	기본					아동
			배뇨장애 간호- 자연배뇨 촉진방법, 수분섭취, 요실금간호(방광조절훈련, 케겔운동)	성인	성인				기본	기본					아동
			단순도뇨, 유치도뇨, 자가도뇨교육, 콘돔카테터, 치골상부도뇨관 간호, 요로전환술, 요로감염 예방간호, 방광세척	성인	성인				기본	기본					아동
			배뇨간호 결과 평가	성인	성인				기본	기본					아동
	62	투석대상자 관리	복막투석, 혈액투석, CRRT, 동정맥루 관리, 투석간호	성인	성인										
	63	요루 및 장루 관리	요루 관리	성인	성인										
			장루 관리	성인	성인										
	64	기관절개부 관리	기관절개관의 적응증 및 간호	성인	성인				기본	기본					
	65	배변관리	배변요구와 관련된 간호진단	성인	성인				기본	기본					아동
			관장종류(청결관장, 정체관장, 구풍관장, 투약관장, 용수관장, 역류관장)별 목적 및 간호						기본	기본					
			배변장애 간호	성인	성인				기본	기본					아동
			변비, 설사, 고창, 변실금, 분변매복	성인	성인				기본	기본					아동
			염증성장질환(궤양성대장염, 크론병), 대장암, 직장암, 치핵	성인	성인										
			배변장애 간호 결과 평가	성인	성인				기본	기본					아동

대분류	번호	신규간호사 직무항목	지식항목	1차 성인 (141)	2차 성인 (139)	1,2차 관리 (75)	1차 모성 (65)	2차 모성 (72)	1차 기본 (86)	2차 기본 (87)	1차 정신 (96)	2차 정신 (93)	1차 지역 (113)	2차 지역 (127)	2차 아동 (205)
Ⅳ. 기본간호	66	수면과 휴식 간호	수면력, 수면에 영향을 미치는 요인, 수면일지, 신체검진, 진단검사						기본	기본	정신	정신			아동
			수면증진간호 및 결과평가	성인	성인				기본	기본	정신	정신			아동
			수면간호 결과 평가	성인	성인				기본	기본	정신	정신			아동
			수면생리, 수면각성 장애						기본	기본	정신	정신			
			수면각성장애						기본	기본	정신	정신			아동
			피로	성인	성인						정신	정신			
			암, 간염, 만성폐쇄성폐질환, 빈혈, 투석, 결핵, 울혈성 심부전, 갑상샘기능항진/저하증	성인	성인										
	67	피부통합성 사정 및 간호	면역성피부질환(건선,아토피피부염), 감염성피부질환(무좀, 단순포진, 대상포진, 농가진, 연조직염), 화상, 피부종양	성인	성인										아동
	68	이동간호	신체역학의 원리, 이동절차의 일반적 지침, 신체역학을 이용한 들어올리기, 기구 이용한 이동법, 보행 및 보행보조, 보행 보조기구의 종류 및 사용방법						기본	기본					아동
	69	활동과 자기돌봄장애 사정 및 간호	간호력, 활동과 운동 영향요인, 신체검진, ADL, 부동으로 인한 문제	성인	성인				기본	기본					아동
			활동 및 운동 간호진단, 활동 및 운동 간호계획	성인	성인				기본	기본					아동
			관절범위운동의 종류, 목적, 주의점, 각 관절별 수행방법						기본	기본					
			체위의 종류, 올바른 체위 유지목적						기본	기본					아동
			활동과 운동 간호 결과 평가	성인	성인				기본	기본					아동
			운동유형, 운동효과, 부동의 영향						기본	기본					아동
			골절, 고정, 뇌졸중, 무의식, 파킨슨병, 길랑-바레 증후군, 근위축측삭경화증, 다발성경화증, 척수손상	성인	성인										아동

대분류	번호	신규간호사 직무항목	지식항목	1차 성인(141)	2차 성인(139)	1,2차 관리(75)	1차 모성(65)	2차 모성(72)	1차 기본(86)	2차 기본(87)	1차 정신(96)	2차 정신(93)	1차 지역(113)	2차 지역(127)	2차 아동(205)
	70	외과적 장치 관리	외과적 견인장치(splints, braces, casts)이 목적, 종류, 간호	성인	성인										아동
	71	척추손상 및 질환대상자의 간호	절단, 의식장애, 뇌졸중, 파킨슨병, 근무력증, 다발경화증, 근위축측삭경화증, 길랑-바래 증후군, 척수손상, 추간판탈출, 척추관협착	성인	성인										아동
	72	관절대치술 환자간호	관절대치술의 적응증, 수술 전후 간호	성인	성인										
V. 생리적 통합유지	73	호흡기능장애 대상자 간호	호흡기계 해부생리, 호흡과정 및 호흡조절기전, 관류와 환기	성인	성인				기본	기본					아동
			호흡기계 건강사정(정상과 비정상 구별)	성인	성인				기본	기본					아동
			호흡기계 장애 핵심질환: 폐쇄성호흡기질환(천식, 만성폐쇄성폐질환), 염증성호흡기질환(폐렴, 흉막염), 감염성호흡기질환(상기도감염, 폐결핵), 외상성호흡기질환(기흉,늑골골절), 호흡기중증질환(폐색전증, 급성호흡부전, 성인호흡곤란증후군)	성인	성인										아동
			산소요법	성인	성인				기본	기본					아동
			흉부물리요법, 강화폐활량계, 체위배액, 심호흡과 기침, nebulizer	성인	성인				기본	기본					아동
			동맥혈가스분석 해석, 호흡성/대사성 알칼리증과 산증	성인	성인										아동
			아동기 호흡특성, 호흡기계 건강사정, 아동의 비인두염												아동
			핵심질환(인두염, 편도염, 중이염, 크루프, 기관지염, 세기관지염, 폐렴, 결핵) 아동간호												아동

대분류	번호	신규간호사 직무항목	지식항목	1차 성인 (141)	2차 성인 (139)	1,2차 관리 (75)	1차 모성 (65)	2차 모성 (72)	1차 기본 (86)	2차 기본 (87)	1차 정신 (96)	2차 정신 (93)	1차 지역 (113)	2차 지역 (127)	2차 아동 (205)
V. 생리적 통합유지	74	호흡증진중재	기도관리, 검체채취, 흉부물리요법, 강화폐활랑계, 체위배액, 심호흡과 기침, nebulizer	성인	성인				기본	기본					아동
			핵심질환 : 호흡기 중증질환(폐색전증, 급성호흡부전, 성인호흡곤란증후군) 간호	성인	성인										아동
			산소요법, 흉부물리요법(타진, 진동, 체위배액), 강화폐활랑계, 심호흡과 기침, nebulizer	성인	성인				기본	기본					아동
			흡인의 적응증, 절차 및 간호(주의사항 포함)	성인	성인				기본	기본					아동
			심호흡, 기침, 입술오므리기호흡, 횡격막 호흡, 강화폐활량계 목적 및 교육방법	성인	성인				기본	기본					아동
			타진, 진동, 체위배액의 목적, 원리, 적용방법 및 주의점	성인	성인				기본	기본					아동
			산소요법의 종류별 장단점, 목적, 적응증 및 간호	성인	성인				기본	기본					아동
			인공기도관의 적응증 및 관리	성인	성인				기본	기본					
	75	호흡보조장치 관리	산소전달시스템, 진단검사, 약물, 인공호흡기 모드 및 관리방법, 감염관리	성인	성인										
	76	중심정맥관 관리	무균술 적용과 삽입 부위 사정, 주입장치 약물주입,혈액채취시주의점,삽입부위드레싱,합병증종류및간호	성인	성인				기본	기본					
			중심정맥주사(비터널카테터, 말초삽입중심정맥관, 터널카테터, 이식포트) 유형 및 적응증.	성인	성인				기본	기본					아동?
	77	태아질식 증상과 징후 사정 및 간호	신생아 호흡특성, 호흡기계 건강사정(정상, 비정상구분), 고위험신생아 호흡기계 관련장애(호흡곤란증후군, 무호흡, 태변흡인, 기관지폐형성이상, 미숙아망막병)												아동

대분류	번호	신규간호사 직무항목	지식항목	1차 성인 (141)	2차 성인 (139)	1,2차 관리 (75)	1차 모성 (65)	2차 모성 (72)	1차 기본 (86)	2차 기본 (87)	1차 정신 (96)	2차 정신 (93)	1차 지역 (113)	2차 지역 (127)	2차 아동 (205)
V. 생리적 통합유지	78	고위험신생아 보육기 적용간호	고위험 신생아의 통증조절, 광선요법, 교환수혈												아동
	79	호흡재활관리	단계별 호흡재활 간호중재	성인	성인										
	80	심전도관리	심장리듬, 심전도 전극 위치	성인	성인										
	81	순환보조장치관리	심실보조장치, 인공심박동기를 가지고 있는 대상자의 간호	성인	성인										
	82	동맥관관리	동맥관 모니터, 혈역학, 침습적 라인 관리(동맥관 합병증), 동맥혈 가스	성인	성인										
	83	조직관류장애 대상자 사정 및 간호	고혈압, 동맥경화, 대동맥류, 말초동맥질환, 심부정맥혈전증, 정맥류, 림프부종	성인	성인										
	84	체액전해질 불균형 사정 및 간호	부종, 탈수, 화상 간호	성인	성인										아동
			전해질 균형의 생리기전과 증상 및 징후, 관련질환 (고/저칼륨혈증, 고/저나트륨혈증, 고/저칼슘혈증, 부갑상샘기능항진/저하증, 항이뇨호르몬부적절분비증후군)	성인	성인										아동
	85	활동지속성 장애 대상자 사정 및 간호	순환운동과 감각평가, 조기이상, 순환기구, 신체역학, 체위변경 및 가동화 기법	성인	성인										아동
			관련질환(심부전, 관상동맥병, 판막, 빈혈, 호흡기계)	성인	성인										아동
	86	정맥순환증진장치 간호	순환증진 전략, 조직관류, 혈전색전증 예방	성인	성인										

대분류	번호	신규간호사 직무항목	지식항목	1차 성인 (141)	2차 성인 (139)	1,2차 관리 (75)	1차 모성 (65)	2차 모성 (72)	1차 기본 (86)	2차 기본 (87)	1차 정신 (96)	2차 정신 (93)	1차 지역 (113)	2차 지역 (127)	2차 아동 (205)
V. 생리적 통합유지	87	혈액기능장애 대상자 간호	핵심질환 : 빈혈,백혈병,파종성혈관내응고,혈우병,림프종,다발골수종, 응고장애의 병태생리, 증상과 징후	성인	성인										아동
			혈액의 기능 및 구성성분	성인	성인										아동
			혈액계 건강사정, 각 진단검사의 목적 및 결과해석	성인	성인										아동
			혈액제제 투여와 효과 평가, 혈액기능장애 간호문제에 따른 간호중재,	성인	성인										아동
			항응고요법, 혈전용해요법관련 간호중재	성인	성인										
			아동기 혈액 특성, 혈액계 건강사정, 진단검사의 목적 및 결과해석												아동
			철결핍성빈혈, 재생불량성빈혈 환자, 혈우병, 특발성 저혈소판 잭반병 간호	성인	성인										아동
	88	순환기능장애 대상자간호	심박출량 감소, 심장의 구조 및 기능, 심장주기, 심박동 조절 및 심박출량 영향요인	성인	성인										아동
			심혈관계 건강사정(정상과 비정상 구별,흉통, 심계항진, 심잡음)	성인	성인										아동
			핵심질환 : 심부전, 심장판막질환, 심근근육병, 심장눌림증, 심내막염, 심근염, 심장염	성인	성인										아동
			핵심질환 : 심부전, 관상동맥병, 부정맥, 심장눌림증	성인	성인										아동
			아동 심장의 구조 및 기능 및 심박출량 영향요인, 선천심장병의 병태생리												아동
			아동 심혈관계 건강사정(정상과 비정상 구분)												아동
			선천 심장병 (심방중격결손, 심실중격결손, 대동맥축착, 대동맥판협착, 폐동맥판협착, 팔로네증후, 대혈관전위, 심내막염) 간호												아동

대분류	번호	신규간호사 직무항목	지식항목	1차 성인 (141)	2차 성인 (139)	1,2차 관리 (75)	1차 모성 (65)	2차 모성 (72)	1차 기본 (86)	2차 기본 (87)	1차 정신 (96)	2차 정신 (93)	1차 지역 (113)	2차 지역 (127)	2차 아동 (205)
V. 생리적 통합유지	89	심장수술 후 간호	심장수술 후 합병증(예방)관리	성인	성인										
			심장이식의 적응증과 간호관리	성인	성인										
	90	소화기능장애 대상자 간호	소화기계의 구조 및 기능, 소화와 흡수기전	성인	성인										아동
			소화기계 건강사정(정상과 비정상 구분)	성인	성인										아동
			위장질환(위염, 위암, 소화성궤양) 간호	성인	성인										아동
			간질환(간염, 간경화, 간암, 담석증, 담낭염, 담낭암, 췌장염, 췌장암) 간호	성인	성인										아동
			장질환(과민대장증후군, 크론병, 궤양성대장염, 소장흡수불량증후군, 장폐색증) 간호	성인	성인										아동
			복막염 대상자 간호	성인	성인										
			소화기계 질환별 치료식이	성인	성인										아동
			아동기 소화기능의 특성과 소화기계 건강사정												아동
			아동의 급성위장관염, 변비와 유분증, 장중첩증 간호												아동
			고위험신생아 소화기능의 특성과 건강사정												아동
			신생아 소화기계장애 (구순/구개열,기관식도루,항문직장기형,선천성유문협착증,선천거대결장,담도폐쇄, 복벽손상, 탈장, 괴사소장대장염) 간호												아동

대분류	번호	신규간호사 직무항목	지식항목	1차 성인 (141)	2차 성인 (139)	1,2차 관리 (75)	1차 모성 (65)	2차 모성 (72)	1차 기본 (86)	2차 기본 (87)	1차 정신 (96)	2차 정신 (93)	1차 지역 (113)	2차 지역 (127)	2차 아동 (205)
V. 생리적 통합유지	91	배뇨장애를 가진 대상자 간호	비뇨기계 구조 및 기능, 배뇨기전 및 영향요인, 비뇨기계 건강사정 관련질환 (방광염, 사구체신염, 급성.만성신부전, 신장암, 방광암, 신대체요법, 결석, 대사성산증, 전립샘장애)	성인	성인										아동
			아동의 비뇨생식기 특성, 건강사정 비뇨기계 기형 아동간호												아동
			아동의 비뇨생식기 건강문제 (비뇨기계기형, 요로감염, 방광요관역류, 사구체신염, 신증후군) 간호												아동
	92	당질대사장애 대상자 간호	당질대사기전, 저혈당의 원인, 위험요인, 증상, 간호, 고위험신생아의 혈당조절	성인	성인										아동
			소아당뇨병의 증상 및 징후, 진단검사, 혈당감시 및 조절(식이, 약물), 당뇨합병증 예방 및 관리												아동
	93	당뇨병 합병증 예방을 위한 간호	당뇨병의 합병증 증상 및 징후	성인	성인										아동
	94	내분비계장애 대상자 간호	내분비계 해부생리 및 기능(호르몬), 내분비계 건강사정(정상과 비정상 구별)	성인	성인										아동
			뇌하수체질환(뇌하수체 종양, 요붕증, 항이뇨호르몬부적절분비증후군) 간호	성인	성인										아동
			부신질환(쿠싱증후군, 알도스테론증, 에디슨병, 갈색세포종)간호	성인	성인										아동
			갑상샘기능항진/저하증, 부갑상샘기능 항진/저하증 간호	성인	성인										아동
			성장장애, 성조숙 아동 간호												아동
			선천성 갑상선저하증 아동 간호												아동

대분류	번호	신규간호사 직무항목	지식항목	1차 성인(141)	2차 성인(139)	1,2차 관리(75)	1차 모성(65)	2차 모성(72)	1차 기본(86)	2차 기본(87)	1차 정신(96)	2차 정신(93)	1차 지역(113)	2차 지역(127)	2차 아동(205)
V. 생리적 통합유지	95	생식기 질환/생식기 건강문제를 가진 대상자 간호	생식작용과 호르몬, 월경주기, 월경장애의 종류(무월경, 기능성 자궁출혈, 월경전증후군, 월경곤란증), 정의, 원인, 증상 및 징후, 치료방법,				모성	모성							
			무월경, 무월경 치료 및 간호				모성	모성							
			기능성 자궁출혈의 치료 및 간호				모성	모성							
			자궁의 구조 및 기능, 여성 생식기 건강사정				모성	모성							
			자궁내막증, 자궁선근증, 자궁내막증식증, 자궁내막폴립 간호				모성	모성							
			자궁탈수, 자궁전방전위, 생식기누공 간호				모성	모성							
			복압성 요실금 간호				모성	모성							
			난(불)임의 정의 및 진단, 원인, 치료과정				모성	모성							
	96	면역손상 대상자 사정 및 간호	면역반응의 기전 및 건강사정	성인	성인										
			면역손상관련질환(자가면역, 후천면역결핍증후군, 과민반응-알러지, 장기이식, 전신홍반성루프스)의 종류와 간호	성인	성인										
			아동의 면역기전 및 건강사정(정상과 비정상 구분)												아동
			아동의 면역손상관련질환(아토피피부염, 알레르기비염, 천식, 류마티스열, 가와사키병, 헤노흐-쇤라인자색반)												아동
	97	감각기능장애를 가진 대상자 사정 및 간호	감각기계 구조 및 기능, 건강사정(정상과 비정상 구분)	성인	성인										아동
			시각장애 관련질환(염증성눈질환, 망막박리, 녹내장/백내장, 각막이식)간호	성인	성인										아동
			청각장애 관련질환(염증성귀질환, 메니에르병, 난청)	성인	성인										아동

대분류	번호	신규간호사 직무항목	지식항목	1차 성인 (141)	2차 성인 (139)	1,2차 관리 (75)	1차 모성 (65)	2차 모성 (72)	1차 기본 (86)	2차 기본 (87)	1차 정신 (96)	2차 정신 (93)	1차 지역 (113)	2차 지역 (127)	2차 아동 (205)
V. 생리적 통합유지	98	두개내압 상승 환자의 간호	뇌의 구조 및 기능, 뇌 혈액공급, 두개내압 조절기전, 의식수준 사정	성인	성인										아동
			두개내압상승 환자간호	성인	성인										아동
	99	신경계 질환별 간호중재	신경계 신체사정의 정상과 비정상 구별	성인	성인										아동
			신경운동장애 관련질환(뇌졸중, 간질, 파킨슨병, 근무력증, 다발성경화증, 길랑-바레 증후군, 안면신경마비, 삼차신경통, 추간판탈출, 척추외상, 척추관협착)	성인	성인										아동
			뇌동맥류,외상성뇌손상,두통,뇌종양,뇌막염 간호	성인	성인										아동
			뇌졸중 간호	성인	성인										
			인지기능 장애의 원인, 위험요인, 인지기능장애 관련질환(무의식, 뇌졸중, 외상성뇌손상)	성인	성인						정신	정신			
			치매 관련요인, 행동특성, 간호								정신	정신			
			아동기 뇌의 특성, 신경계 건강사정												아동
			아동관련 신경계 장애 관련질환(수막염, 열발적, 뇌전증) 간호												아동
			고위험 신생아 신경계 관련질환(뇌실내 출혈, 경련, 이분척추, 수두증),												아동
			섬망 관련요인, 행동특성, 간호	성인	성인						정신	정신			

대분류	번호	신규간호사 직무항목	지식항목	1차 성인 (141)	2차 성인 (139)	1,2차 관리 (75)	1차 모성 (65)	2차 모성 (72)	1차 기본 (86)	2차 기본 (87)	1차 정신 (96)	2차 정신 (93)	1차 지역 (113)	2차 지역 (127)	2차 아동 (205)
V. 생리적 통합유지	100	운동기능장애 간호중재	근골격계 구조 및 기능, 근골격계 건강사정,	성인	성인										아동
			부동, 운동유형, 관절범위운동, 체위 유지 및 변경, 이동간호, 자가간호결핍 간호	성인	성인										아동
			운동기능장애 관련 근골격계질환(골절, 골관절염, 골다공증, 류마티스관절염, 요통, 통풍, 골수염, 염좌, 강직척추염, 관절대치술, 절단)간호	성인	성인										아동
			운동기능장애 관련 신경계질환(뇌졸중, 길랑바레증후군, 근위축측삭경화증, 파킨슨병, 척수손상, 추간판탈출, 척추관협착, 의식장애)간호	성인	성인										아동
			아동의 근골격계 특성 및 관련질환(척추만곡, 근육디스트로피, 뇌성마비, 소아류마티스관절염)간호												아동
	101	상처간호 수행 및 드레싱 교환	상처유형, 영향요인, 상처의 상태	성인	성인				기본	기본					
			욕창단계, 욕창위험요인, 욕창위험요인 측정도구, 욕창간호	성인					기본	기본					
			드레싱 종류 별 적응증, 상처세척, 배액관관리, 붕대 및 바인더 적용	성인					기본	기본					
	102	화상환자 간호중재	화상의 정의, 원인, 병태생리, 임상증상, 치료과정, 간호중재	성인	성인										아동
	103	피부질환 대상자의 간호중재	피부계 구조 및 기능, 피부계 건강사정(정상과 비정상 구별)	성인	성인										
			아동피부질환(접촉피부염, 기저귀피부염, 농가진, 옴, 머릿니, 칸디다증, 혈관종, 여드름) 간호												아동
			피부통합성장애 관련질환(면역성피부질환, 감염성 피부질환, 화상, 피부종양)간호	성인	성인										아동
	104	배액장치관리	흉관, 상처 음압 장치 유지 및 관리, 밀봉흉곽배액을 가진 대상자 간호	성인	성인										

대분류	번호	신규간호사 직무항목	지식항목	1차 성인 (141)	2차 성인 (139)	1,2차 관리 (75)	1차 모성 (65)	2차 모성 (72)	1차 기본 (86)	2차 기본 (87)	1차 정신 (96)	2차 정신 (93)	1차 지역 (113)	2차 지역 (127)	2차 아동 (205)
V. 생리적 통합유지	105	계통별 신생물 질환 대상자의 간호	관련 기관의 구조 및 기능, 각 종양의 원인, 증상 및 징후, 검사	성인	성인		모성	모성							아동
			신생물 질환 치료방법, 방사선요법, 항암화학요법, 수술 전 간호, 수술 후 간호	성인	성인		모성	모성							아동
			생식기종양(외음양성질환, 외음암, 자궁경관 상피내종양, 자궁경부암, 자궁근종, 자궁내막암, 융모상피암, 난소낭종, 난소양성종양, 난소암) 간호				모성	모성							
			소아종양(조혈모세포이식/골수이식, 골수생검, 백혈병, 뇌종양, 신경모세포종, 빌름스종양, 골육종)간호												아동
	106	통증간호	통증의 원인 및 영향요인, 통증사정, 통증 완화를 위한 약물요법 및 비약물요법	성인	성인		모성	모성							아동
	107	재활간호서비스	재활간호	성인	성인								지역	지역	아동
			정신사회재활								정신	정신	지역	지역	

대분류	번호	신규간호사 직무항목	지식항목	1차	2차	1,2차	1차	2차	1차	2차	1차	2차	1차	2차	2차
				성인 (141)	성인 (139)	관리 (75)	모성 (65)	모성 (72)	기본 (86)	기본 (87)	정신 (96)	정신 (93)	지역 (113)	지역 (127)	아동 (205)
V. 생리적 통합유지	108	(법정) 감염질환자의 전파예방 및 간호	대상 기관의 구조 및 기능, 건강사정, 전파경로별 격리주의, 의료폐기물관리												
			여성생식기의 구조 및 기능, 건강사정, 외음의 감염성 질환				모성	모성							
			외음의 감염성 질환, 세균성 질염, 트리코모니스질염, , 캔디다성 질염, 노인성 질염, 자궁경부염, 난관염, 골반감염 간호				모성	모성							
			성전파성 질환(후천성면역결핍증, 단순포진 바이러스성 질환, 인유두종 바이러스성 질환, 매독 세균성 질환, 임질 세균성 질환, 클라미디아 세균성 질환) 간호				모성	모성							
			아동기 면역의 특성												아동
			아동기 호발 전파성질환(수두, 풍진, 홍역, 볼거리, 일본뇌염, 돌발피진(장미진), 성홍열, 손발입병, 파상풍, 디프테리아, 백일해)												아동
			질병의 자연사와 예방수준										지역	지역	
			집단면역										지역	지역	
			감염성 질환의 잠복기										지역	지역	
			질병발현의 역학적 요인										지역	지역	
			역학적 측정지표(유병률, 사망률)										지역	지역	
			진단검사 및 역학적 연구										지역	지역	
			법정전염병의 정의, 종류, 지정 목적 및 관리방법										지역	지역	

대분류	번호	신규간호사 직무항목	지식항목	1차<br>성인<br>(141)	2차<br>성인<br>(139)	1,2차<br>관리<br>(75)	1차<br>모성<br>(65)	2차<br>모성<br>(72)	1차<br>기본<br>(86)	2차<br>기본<br>(87)	1차<br>정신<br>(96)	2차<br>정신<br>(93)	1차<br>지역<br>(113)	2차<br>지역<br>(127)	2차<br>아동<br>(205)
VI. 약물 및 비경구요법	109	투약의 적절성과 정확성	투약의 5원칙						기본	기본			지역	지역	
			약물작용기전과 유형						기본	기본	정신	정신	지역	지역	
			약물투여경로별 목적 및 특성						기본	기본			지역	지역	
			경구투여절차 및 평가						기본	기본			지역	지역	아동
			처방된 약품의 적절성과 처방의 정확성 평가						기본	기본			지역	지역	아동
			근육/피하/피내/국소투여/정맥주사 절차 및 평가						기본	기본			지역	지역	아동
			검진이나 검사 결과에 기반하여 약물의 투여량 확인 (혈당수치에 따른 인슐린 투여, 특정 혈압을 유지하기 위해 약물의 투여)										지역	지역	아동
	110	약물 투여에 필요한 계산 시행	약용량 계산(성인/아동)						기본	기본					아동
	111	정맥주입장치 관리	중심정맥관, 이식형 포트 관리						기본	기본					
	112	약품관리	지침에 따라 규제약물을 관리(증인, 잔량폐기), 안전하고 통제된 환경에서 의약품 취급 및 관리												
			투약오류 보고 및 예방												
	113	말초정맥관 삽입, 유지, 제거	말초정맥관 삽입/유지/제거						기본	기본					
	114	대상자에게 약물에 대해 교육	약물치료에 대한 대상자의 반응(치료효과, 부작용, 이상반응) 평가	성인	성인		모성	모성	기본	기본	정신	정신	지역	지역	
	115	고위험 약품관리 (마약관리)	마약관리방법								정신	정신			

대분류	번호	신규간호사 직무항목	지식항목	1차	2차	1,2차	1차	2차	1차	2차	1차	2차	1차	2차	2차
				성인 (141)	성인 (139)	관리 (75)	모성 (65)	모성 (72)	기본 (86)	기본 (87)	정신 (96)	정신 (93)	지역 (113)	지역 (127)	아동 (205)
Ⅶ. 심리 사회적 통합유지	116	이상행동 사정 및 간호	정신간호의 원리(정신간호의 개념, 정신간호사의 역할과 기능, 치료적 자기이용, 자기인식, 대상자-간호사의 상호작용)								정신	정신			
			이상행동 사정과 간호								정신	정신			
			정신간호의 발달								정신	정신			
			정신간호의 이론적모형(정신분석모형, 대인관계모형, 사회적 모형, 실존적 모형, 행동모형, 의사소통모형, 간호모형 등)								정신	정신			
			스트레스, 대처 및 관리(스트레스 개념, 반응 및 이론, 스트레스 대처기전 및 관리기법)								정신	정신			
			정신건강사정(정신건강 개념, 신체와 정신과의 관계, 정신건강과 정신질환의 연속성, 정신건강 영향 요인, 정신건강 상태 사정)								정신	정신			
	117	폭력의 잠재성 평가 및 예방	위기사정 및 관리(위기의 정의, 특성, 종류 및 중재, 자살의 단서, 정신역동, 위험요인 및 예방적 간호중재, 자살 시도자 및 유가족 간호중재, 공격행동 사정 및 대처방안)								정신	정신			
	118	학대 또는 방임 대상자를 확인하고 적절하게 중재	성폭력의 정의, 원인, 영향 및 성폭력 피해 대상자 간호				모성	모성			정신	정신			
			가정폭력의 개념, 유형(아동학대, 배우자학대, 노인학대), 가해자와 피해자의 특성, 예방 및 대처방안				모성				정신	정신	지역	지역	아동
			물질관련 및 중독장애의 정의, 유형(물질관련장애, 도박장애), 관련요인, 정신역동, 행동특성과 물질관련 및 중독장애 대상자 간호								정신	정신			
	119	중독 사정 및 간호	중독의 유형, 중독의 유형에 따른 간호								정신	정신			

대분류	번호	신규간호사 직무항목	지식항목	1차 성인 (141)	2차 성인 (139)	1,2차 관리 (75)	1차 모성 (65)	2차 모성 (72)	1차 기본 (86)	2차 기본 (87)	1차 정신 (96)	2차 정신 (93)	1차 지역 (113)	2차 지역 (127)	2차 아동 (205)
Ⅶ. 심리 사회적 통합유지	120	정신사회건강문제 간호 및 교육	인간의 정신생물학적 이해(뇌신경계의 구조와 기능, 신경전달물질의 기능, 정신질환과 신경전달물질, 신경계 및 기타 생물학적 관련성, 뇌영상기법)								정신	정신			
			인간의 정신심리적 이해(정신역동, Freud의 의식구조, 성격 구조, 방어기전 및 정신성적 발달이론, Erikson의 정신사회적 발달이론, Sullivan의 대인관계 이론, Mahler의 분리개별화 이론)								정신	정신			
			정신치료기법의 이해(약물치료, 기타 생물학적 치료, 환경치료, 활동치료, 개인 정신치료, 집단 정신치료, 가족치료, 인지행동치료 등)								정신	정신			
			간호사정, 진단시 지지제공, 가족의 대처방법에 대한 지지, 만성질환과 일반적인 건강관리에 대한 교육, 정상발달 증진, 현실적인 미래의 목표 설정								정신	정신	지역		
			아동의 만성질환이 미치는 영향(부모, 형제자매), 지속되는 스트레스와 주기적인 위기의 대처, 가족구성원의 감정을 조절하도록 돕기, 지지체계확립								정신	정신			
			치료적 환경의 개념, 요소 및 치료적 환경 조성								정신	정신			
			섭식장애의 정의, 유형(신경성 식욕부진증, 신경성 폭식증, 폭식장애 등), 관련요인, 정신역동, 행동특성 및 섭식장애 대상자 간호								정신	정신			
			정상 성반응과 성정체감 발달				모성	모성			정신	정신			
			성 관련장애의 정의, 유형(성기능부전, 성별불편감, 변태성욕장애), 관련요인, 행동특성 및 성 관련장애 대상자 간호								정신	정신			

대분류	번호	신규간호사 직무항목	지식항목	1차 성인 (141)	2차 성인 (139)	1,2차 관리 (75)	1차 모성 (65)	2차 모성 (72)	1차 기본 (86)	2차 기본 (87)	1차 정신 (96)	2차 정신 (93)	1차 지역 (113)	2차 지역 (127)	2차 아동 (205)
Ⅶ. 심리 사회적 통합유지	121	정신질환자 간호	조현병장애의 정의, 관련요인, 행동특성 및 조현병장애 환자 간호								정신	정신			
			기타 정신병적 장애의 정의, 유형(조현양상장애, 단기 정신병적 장애, 조현정동장애, 망상장애 등), 행동특성 및 기타 정신병적 장애 환자 간호								정신	정신			
			우울장애의 정의, 유형(주요우울장애, 기분저하장애), 관련요인, 행동특성 및 우울장애 환자 간호								정신	정신			
			양극성 및 관련 장애의 정의, 유형(제1형 양극성장애, 제2형 양극성장애, 순환성장애), 관련요인, 행동특성 및 양극성장애 환자 간호								정신	정신			
			불안 정도 사정 및 불안 수준별 간호								정신	정신			
			불안장애의 정의, 유형(공황장애, 광장공포증, 특정공포증, 범불안장애, 사회불안장애), 관련요인, 행동특성 및 불안장애 환자 간호								정신	정신			
			강박 및 강박 관련 장애의 개념, 유형(강박장애, 신체변형장애 등), 관련요인, 행동특성 및 강박 및 강박 관련 장애 환자 간호								정신	정신			
			외상 및 스트레스 관련 장애의 개념, 유형(외상후 스트레스장애, 적응장애 등), 관련요인, 행동특성 및 외상 및 스트레스 관련 장애 환자 간호								정신	정신	지역	지역	
			신체증상 및 관련 장애의 정의, 유형(신체증상장애, 전환장애, 질병불안장애, 인위성장애), 관련요인, 행동특성 및 신체증상 및 관련 장애 환자 간호								정신	정신			
			해리장애의 정의, 유형(해리성 정체성장애, 해리성 기억상실, 이인성/비현실감 장애), 관련요인, 행동특성 및 해리장애 환자 간호								정신	정신			
			인격장애의 정의, 유형(편집성, 조현성, 조현형, 반사회성, 경계성, 연극성, 자기애성, 의존성, 강박성, 화피성 인격장애), 관련요인, 정신역동, 행동특성 및 인격장애 환자 간호								정신	정신			

대분류	번호	신규간호사 직무항목	지식항목	1차	2차	1,2차	1차	2차	1차	2차	1차	2차	1차	2차	2차
				성인 (141)	성인 (139)	관리 (75)	모성 (65)	모성 (72)	기본 (86)	기본 (87)	정신 (96)	정신 (93)	지역 (113)	지역 (127)	아동 (205)
Ⅶ. 심리 사회적 통합유지	122	아동정신질환자 간호	신경발달장애의 정의, 유형(지적장애,자폐스펙트럼장애, 주의력결핍 과인행동장애, 특정학습장애, 틱/뚜렛장애 등), 행동특성 및 신경발달장애 환자 간호								정신	정신			아동
			반응성 애착장애 대상자의 행동특성과 간호								정신	정신			아동
			분리불안장애 대상자의 행동특성과 간호								정신	정신			아동
			파괴적, 충동조절 및 품행장애의 정의, 유형(적대적 반항장애, 품행장애 등), 행동특성 및 파괴적, 충동조절 및 품행장애 대상자 간호								정신	정신			
			배설장애 대상자의 행동특성과 간호								정신	정신			아동
	123	임종간호와 교육	사후관리(post-mortem care), 사전연명의료의향서(advance directives)	성인	성인										
			임종징후 사정	성인	성인										
			품위 있는 죽음, 임종 시 신체적 간호, 정신적, 영적간호, 가족 지지	성인	성인						정신	정신			
			사후 신체적 변화, 사체 관리, 사별 가족 지지 / 발달단계에 따른 아동의 죽음에 대한 이해, 아동의 사망과 관련된 윤리적 쟁점, 임종을 앞둔 아동의 간호, 애동(부모, 형제자매), 간호사의 반응	성인	성인						정신				아동
	124	치료적 의사소통 기법	치료적 인간관계와 간호사의 역할								정신	정신			아동
			의사소통의 개념과 치료적, 비치료적 의사소통 기법								정신	정신			아동
			상담기술								정신	정신			아동
Ⅷ.	125	(지역사회)건강교육 계획	역학의 정의와 목적, 질병의 역학모형										—	지역	
			보건교육 개념과 보건교육										지역	지역	
			학습이론, 건강행위이론, 보건교육관련이론										지역	지역	
			보건교육, 학교보건교육										지역	지역	

대분류	번호	신규간호사 직무항목	지식항목	1차 성인 (141)	2차 성인 (139)	1,2차 관리 (75)	1차 모성 (65)	2차 모성 (72)	1차 기본 (86)	2차 기본 (87)	1차 정신 (96)	2차 정신 (93)	1차 지역 (113)	2차 지역 (127)	2차 아동 (205)
Ⅷ. 건강증진 및 유지	126	건강증진 및 유지관리에 대한 정보(예방접종 등) 제공	정신건강증진의 개념, 취약집단 및 정건강증진사업										지역	지역	
			개인/가족/지역사회/인구집단의 건강위험										지역	지역	
			건강증진요인 및 위험요인										지역	지역	아동
			인구구조의 유형별 특징										지역	지역	아동
			한국의 인구정책										지역	지역	
			생활습관개선을 위한 건강증진사업										지역	지역	아동
	127	고위험 건강 행위의 예방 및 치료에 대한 정보(금연, 안전한 성 행위, 바늘교환) 제공	만성질환관리								정신	정신	지역	지역	
			영유아보건사업										지역	지역	아동
			여성보건사업										지역	지역	
			노인보건사업								정신	정신	지역	지역	
			맞춤형 방문간호사업								정신	정신	지역	지역	아동
			가족계획				모성	모성					지역	지역	
			성의 개념과 성건강				모성	모성					지역	지역	아동
	128	성건강증진간호	피임의 원리와 유형				모성	모성							
	129	문화간호	우리나라 가족										지역	지역	
			다문화사회의 건강행태와 신념										지역	지역	아동
			문화적 특성에따른 건강과 질병, 다문화가족의 건강특성										지역	지역	아동
	130	산업과 환경 간호	산업재해 관리, 작업환경관리										지역	지역	
			환경보건, 환경건강정보										지역	지역	
			지역사회/산업장/가정 환경사정										지역	지역	
			risk communication의 원칙										지역	지역	
			근로자 건강진단										지역	지역	
			작업관련성 질환의 예방 및 관리										지역	지역	
			산업보건과 산업간호										지역	지역	

대분류	번호	신규간호사 직무항목	지식항목	1차 성인 (141)	2차 성인 (139)	1,2차 관리 (75)	1차 모성 (65)	2차 모성 (72)	1차 기본 (86)	2차 기본 (87)	1차 정신 (96)	2차 정신 (93)	1차 지역 (113)	2차 지역 (127)	2차 아동 (205)
Ⅷ. 건강증진 및 유지	131	재난간호	공중보건상의 재난 시 지켜야할 원칙의 적용과 활용										지역	지역	
			공중보건법										지역	지역	
			위기/재난시 의사소통체계										지역	지역	
			국제재난구조단체										지역	지역	
	132	치료계획을 결정하기 위해가족 역동성 (family dynamics) (가족 구조, 결속, 의사소통, 경계, 대처기전) 사정	가족역동성 사정										지역	지역	
	133	가정환경에서 대상자를 관리할 수 있는 역량(장비, 지역사회 자원) 평가	지역사회 자원평가										지역	지역	
	134	건강위험요인 사정과 교육	학교건강검사										지역	지역	아동
			학교보건교육										지역	지역	아동
			지역사회 정신보건								정신	정신	지역	지역	

【부록4】 간호사 국가시험 출제모형에 대한 간호행정학회 의견

1영역. 간호관리와 전문성 향상

대분류	No	신규간호사 직무항목 (Activity Statement 개정안)	지식항목 (Knowledge statement)	1차 의견	2차 의견
I. 간호 관리와 전문성 향상	1	인수인계시행	업무 인수인계		
	2	법적 실무범위 내에서 간호 수행	간호표준실무, 간호관리표준실무		관리-간호사의 법적 의무와 책임<br>지역-보건의료관련법과 지역사회간호실무 관련성
	3	기록 시 표준화된 약어 사용	간호기록 시 표준약어	간호기록지침 안에 포함<br>관리-기록과 관련된 간호사의 책임	간호기록지침 안에 포함<br>관리-기록과 관련된 간호사의 책임
	4	지침에 따라 간호기록	간호기록지침	관리-기록과 관련된 간호사의 책임	관리-기록과 관련된 간호사의 책임
			환자 개인정보 보호		관리-간호정보와 기록관리 측면에서 환자 개인정보보호전략과 구분
			간호정보시스템		
	5	입원, 전동, 퇴원	입원관리, 퇴원관리 및 교육, 전과전동업무		
			입퇴원, 전과전동시 인수인계		
			고위험 환자관리체계		
	6	장비를 적절하고 안전하게 사용	물품관리		관리-병원 내 물품관리의 중요성, 간호단위 물품교환체계와 구분
	7	간호단위 물품교환체계에 따른 물품관리	약품관리		관리-병원 내 약품처방체계, 투약오류, 투약오류 감소전략과 구분
			마약 및 고위험 약품관리		관리-병원 내 마약관리, 고위험약품관리와 구분
	8	질 향상(QI) 활동에 참여	의료의 질관리, 간호의 질관리 및 질향상활동		
			질관리 전략 및 절차		
			의료기관인증제도 및 인증기준		

대분류	No	신규간호사 직무항목 (Activity Statement 개정안)	지식항목 (Knowledge statement)	1차 의견	2차 의견
			질관리 평가기준 및 도구		
	9	간호사업 평가 관련 업무수행 (도구개발, 자료조사, 분석, 비교 및 사업 개선)	간호사업평가	지역	
			간호관리 통제기능	지역-보건간호사업 질관리<br>간호관리의 통제 부분과 구별 필요	
	10	간호전문직 윤리 준수와 역할	간호윤리, 생명의료윤리 원칙, 윤리이론 및 윤리적 사고		
			한국간호사 윤리강령		
			연구 윤리		
			전문직 협력관련 간호윤리		관리-간호사와 의사, 간호사와 타직종, 간호사와 동료 관련 간호윤리문제와 구분 필요
			자원분배 관련 간호윤리		관리- 정의의 원칙, 간호업무의 공평한 분배와 관련된 윤리문제와 구분 필요
			간호전문직관		
			간호사의 임무와 역할		관리- 간호사의 법적의무와 법적책임 부분과 구분 필요
			소수집단 인권보호 및 윤리적 간호	정신-정신건강문제 인권 부분과 구별 필요	관리-간호윤리 실무적용<br>정신-정신건강문제 인권 부분과 구별 필요
			정신건강복지법		
			공중보건관리 법률		
	11	대상자의 개인정보 및 사생활 보호	대상자의 개인정보 및 사생활 보호권리(사생활, 인격권, 정보보호권, 평등권)		관리-대상자의 법적권리 정의와 구분 필요
			대상자의 법적 권리 보호방안		관리-대상자의 법적권리 보호방안과 구분 필요
			공중보건 상 재난 시 자료의 비밀보장		
	12	대상자에게 치료 및 절차에 따	간호사의 법적 의무		관리-간호사의 법적의무와 구분 필요

대분류	No	신규간호사 직무항목 (Activity Statement 개정안)	지식항목 (Knowledge statement)	1차 의견	2차 의견
		라 적절한 설명을 하고 동의를 획득하였는지 확인			
	13	환자의 권리와 책임에 관하여 대상자에게 교육 제공	간호와 치료에 관한 환자권리 및 책임		관리- 간호사의 법적의무와 법적책임 부분과 구분 필요
			간호사의 법적 의무와 책임		관리- 간호사의 법적의무와 법적책임 부분과 구분 필요
			환자권리존중 및 보호		관리-대상자의 법적권리 보호방안과 구분 필요
	14	억제대 사용시 법적 및 윤리적 간호	신체억제대 사용지침, 억제대 사용관련 윤리적 사고 및 결정		관리-신체억제대 사용지침, 억제대 사용관련 윤리적 사고 및 결정과 구분 필요
	15	일차의료기반의 보건간호	일차보건의료		
			지역사회간호의 정의		
			지역사회와 건강개념		
			건강의 결정요인		
			국제보건기구의 간호정책 및 활동		
			보건의료체계의 이해		
			진료비 지불보상제도		관리-건강보험수가체계, 상대가치개념, 행위별수가제, 포괄수가제, 간호관리료차등제와 구분 필요
	16	지역사회 간호사업의 법적 기준 및 지침에 따른 활동 참여	보건관리자의 역할 및 업무		
			산업전문간호사의 역할		
			보건/간호사업 기획 및 수행		
			보건교육자, 보건진료원의 역할		
			보건정보 및 기술계획		
			건강증진 종합계획		
			간호사업의 법적 기준 및 지침		
	17	사례관리활동 참여	사례관리 정의 및 원칙	지역 학습목표와 동일	지역 학습목표와 동일

대분류	No	신규간호사 직무항목 (Activity Statement 개정안)	지식항목 (Knowledge statement)	1차 의견	2차 의견
				입원환자 사례관리는 간호관리이므로 구별 필요	입원환자 사례관리는 간호관리이므로 구별 필요
			조정과 협력	전문직간 협업에 중복되는 내용, 구별 필요	전문직간 협업에 중복되는 내용, 구별 필요
			간호관리 체계 및 과정	지역-체계이론의 주요개념과 구별 필요	지역-체계이론의 주요개념과 구별 필요
			사례관리모델, 사례관리과정	지역 학습목표와 동일<br>입원환자 사례관리는 간호관리이므로 구별 필요	지역 학습목표와 동일<br>입원환자 사례관리는 간호관리이므로 구별 필요
	18	업무를 조직화하여 일을 효율적으로 관리	간호관리업무, 역량		간호관리업무와 간호관리역량 부분은 간호관리 이므로 구별 필요
			관리이론		
			기획 원칙 및 구성요소, 기획단계		
			계획안과 목표관리		
			의사결정		
			예산관리		
			조직화 원리, 조직구조 및 권한		
			직무설계, 직무분석, 직무평가		
			간호전달체계		
			간호조직문화와 조직변화		
			간호인적자원 관리		
	19	환자분류체계 관련 정보수집 및 활용	환자분류체계		관리-인적자원관리 중 확보관리의 환자분류체계 개념, 목적, 접근방법, 인력산정방법과 구분
			간호업무량 및 인력산정, 간호업무배정		관리-인적자원관리 중 확보관리의 환자분류체계 개념, 목적, 접근방법, 인력산정방법과 구분
	20	간호대상에 적합한 다양한 자	간호서비스마케팅		관리- 간호서비스마케팅에서 개념, 믹스,

대분류	No	신규간호사 직무항목 (Activity Statement 개정안)	지식항목 (Knowledge statement)	1차 의견	2차 의견
		원 및 매체선택			4가지 전략과 구분 필요
	21	취약가족에 필요한 지역사회 자원 활용	취약가족과 간호, 가족의 개념		
			가족생활주기의 특성		
			가족관련이론		
			가족간호과정		
	22	전문직 간 협업	조정과 협력		
			리더쉽		
			동기부여이론 및 동기부여 적용		
			임파워먼트		
			효과적인 의사소통		관리-조직 내 의사소통 유형, 네트워크, 효과적 의사소통 방안과 구분 필요
			주장행동		
			갈등관리, 직무스트레스 관리		

2영역. 안전과 감염관리

대분류	No	신규간호사 직무항목 (Activity Statement 개정안)	지식항목 (Knowledge statement)	1차 의견	2차 의견
II. 안전과 감염 관리	23	안전한 환경 제공	사생활유지관리		관리-간호단위 내 사생활 유지를 위한 방법과 구분 필요
			환경관리 및 환자안전관리		관리-간호단위 내 환경관리의 중요성, 안전관리의 개념, 안전사고 예방법, 고위험 의료기기 예방점검 및 유지관리 활동과 구분 필요
			환자안전사고 예방(관련법 포함)		관리-간호단위 내 안전사고 예방법과 구분 필요

대분류	No	신규간호사 직무항목 (Activity Statement 개정안)	지식항목 (Knowledge statement)	1차 의견	2차 의견
			환자안전사고 보고체계 및 사고기록		관리-통제 중 환자안전, 간호단위 내 사건보고체계와 구분 필요
			환자안전문제 발생 시 대응방안 및 운영체계		관리-통제 중 환자안전 운영체계, 환자안전문제 발생 시 사건보고체계, RCA 분석과 구분 필요
			조직의 환자안전문화		관리-조직의 환자안전문화, 환자안전전략과 구분 필요
			보안계획(신생아실 안전, 폭력, 통제된 접근) 및 절차 준수		
			안전사고 유형, 위험요인과 예방		관리-환자안전 관련 용어, 환자안전 원칙과 구분 필요
			낙상예방 및 관리		관리-간호단위 내 고위험 환자관리 수행과 구분 필요
			안전사고 시 기록		관리-환자안전문제 발생 시 사건 보고체계, 기록과 관련된 간호사의 책임과 구분 필요
			억제대 적응증, 억제대 종류, 억제대 적용 시 주의점, 적용방법	신체억제대 사용지침, 억제대 사용 관련 윤리적 사고 및 결정 - 간호관리, 기본간호학와 구별 필요	
	24	감염관리	병원감염관리		관리-병원감염관리와 감염관리지침은 간호관리이므로 구별 필요
			감염관리지침		관리-병원감염관리와 감염관리지침은 간호관리이므로 구별 필요
			무균술의 기본원리		
			소독방법 / 소독수준, 소독방법, 소독제의 종류와 용도		
			멸균방법 / 멸균준비, 멸균방법과		

대분류	No	신규간호사 직무항목<br>(Activity Statement 개정안)	지식항목<br>(Knowledge statement)	1차 의견	2차 의견
			적용		
			무균술의 원리, 상처감염예방법		
			외과적 무균술 정의,<br>외과적 무균술의 기본원리와 적용		
			표준주의지침	기본학습목표와 동일 - 병원감염관리와 감염관리지침은 간호관리이므로 구별 필요	관리-병원감염관리와 감염관리지침은 간호관리이므로 구별 필요
			감염위험요인 사정 / 감염유형, 감염회로, 감염에 대한 신체방어, 감염 감수성에 영향을 미치는 요인		
			의료관련감염 정의, 종류,<br>위험요인, 교차감염		
			병원감염(요로감염, 폐렴, 수술창상감염, 혈액감염 등) 관리 및 결과평가	기본학습목표와 동일 - 병원감염관리와 감염관리지침은 간호관리이므로 구별 필요	관리-병원감염관리와 감염관리지침은 간호관리이므로 구별 필요
			전파경로별 주의지침<br>(공기주의, 접촉주의, 비말주의),<br>다제내성균 감염관리,<br>면역저하 대상자 관리(역격리),<br>보호장구 착용과 벗기	기본학습목표와 동일 - 병원감염관리와 감염관리지침은 간호관리이므로 구별 필요	관리-병원감염관리와 감염관리지침은 간호관리이므로 구별 필요
			의료폐기물 종류별 관리 및<br>주의점	기본학습목표와 동일 - 병원감염관리와 감염관리지침은 간호관리이므로 구별 필요	
	25	위험물질과 유해물질 관리	고위험약품 및 마약관리	간호관리와 전문성 향상 실무 항목 7번과 중복	간호관리와 전문성 향상 실무 항목 7번과 중복

【부록5】 간호사 국가시험 출제모형에 대한 여성건강간호학회 의견

1영역. 간호관리와 전문성 향상

대분류	No	신규간호사 직무항목 (Activity Statement 개정안)	지식항목 (Knowledge statement)	1차 의견	2차 의견
I. 간호 관리와 전문성 향상	12	대상자에게 치료 및 절차에 따라 적절한 설명을 하고 동의를 획득하였는지 확인	간호사의 법적 의무		난임여성 관리에서 중요한 부분이라고 사료되어 추가함
	21	취약가족에 필요한 지역사회 자원활용	취약가족과 간호, 가족의 개념		가족중심간호를 반영하는 것 같아 추가함
			가족생활주기의 특성		
			가족관련이론		
			가족간호과정		

3영역. 위험요인 사정

대분류	No	신규간호사 직무항목 (Activity Statement 개정안)	지식항목 (Knowledge statement)	1차 의견	2차 의견
III. 위험 요인 사정	29	신생아 간호	청결한 기도유지, 고무흡인기의 적절한 사용, 호흡측정 및 호흡양상 관찰		분만직후 신생아간호라면 포함
			체온유지간호		
			신생아목욕간호, 제대간호, 기저귀발진 예방		
			신생아의 영양, 모유수유/인공수유 방법, 신생아 수유행동, 체중측정		분만직후 신생아 간호는 모성에 포함되고, 모유수유는 중요한 부분이므로 추가함
			감염과 상해로부터 보호, 예방접종		
			애착행동사정, 부모-신생아 애착증진 간호		
			신생아 퇴원기준목록, 신생아 돌봄지도,		

대분류	No	신규간호사 직무항목 (Activity Statement 개정안)	지식항목 (Knowledge statement)	1차 의견	2차 의견
			수면과 활동, 신생아 선별검사와 예방접종		
	37	성 건강 간호	월경전 증후군 간호	분류오류로 사료됨. 95번에도 해당 질환이 포함이 되어 있어서 이중 배속됨. 95번으로 가는 것이 좋을 것으로 봄	월경전 증후군은 95. 생식기 질환 건강문제 대상자 간호에 포함되어 있으므로, 여기서는 삭제하고, '성교육과 성상담, 원치 않는 임신' 추가
	40	생식기 건강사정	여성생식기 사정		'여성생식기 구조와 기능, 생식작용과 호르몬, 월경주기' 추가
			생식기 검진 전후 간호(질경검진, 자궁경부세포검사, 세포도말검사)	'46. 진단검사간호'와 중복됨	'생식기 건강사정(추가) 및 검진 전후 간호(질경검진, 자궁경부세포검사, 세포도말검사)'
			유방건강사정		
	41	태아 건강사정 및 간호	태아건강사정(태아초음파, 태동측정, 신체계측, 생물리학적 계수, 모체혈청검사, 태아전자감시, 양수천차, 융모막생검)		'태아발달(추가), 태아건강사정(태아초음파, 태동측정, 신체계측, 생물리학적 계수, 모체혈청검사, 태아전자감시, 양수천차, 융모막생검)'
	46	진단검사 간호	진단검사 수행, 진단검사결과 확인, 진단검사 전후 간호		
			침습적 시술(중심정맥관, 흉강천자, 기관지내시경)		
			침습적 검사방법(양수천자, 경피제대혈채취, 유모막융모생검) 검사 전후 간호		
			생식기 검진 전후 간호(질경검진, 자궁경부세포검사, 세포도말검사)	'40. 생식기 건강사정'과 중복된 내용	40번과 중복되므로, 삭제
	48	산전간호와 교육	임부의 생리적 변화		"위험요인 사정" 영역이 아니라, "건강증진 및 유지영역"에 포함되는 것이 적절하다는
			임부의 건강사정과 산전간호		

대분류	No	신규간호사 직무항목 (Activity Statement 개정안)	지식항목 (Knowledge statement)	1차 의견	2차 의견
			임부간호(임신주기별 간호)		이사진의 의견이 있었음. 영역이동이 필요함 (직무항목 48,49,50)
			임부와 가족의 사회심리적 적응과 간호		
	49	분만중 간호와 교육	분만관련 주요개념(분만요소, 산도, 골반경선, 아두경선, 태세, 태향, 태위, 선진부)		
			분만생리(분만과정, 분만기전, 자궁수축, 분만전구증상)		
			통증완화간호(통증사정, 비약물적방법, 약물적 방법)		
			산부 간호(1기, 2기, 3기, 4기, 간호, 상호작용증진)		
	50	산후관리와 교육	산후생리적 변화		
			산모와 가족의 사회심리적 적응과 간호(사회심리적 변화, 모성역할, 애착증진간호)		
			산모 간호(산후건강사정과 건강관리)		
			모유수유 간호		
	51	고위험임부 간호	고위험 임신요인 사정		
			출혈성 임신 건강문제가 있는 임부 간호(유산, 자궁경관무력증, 자궁외임신, 포상기태, 전치태반, 태반조기박리)		
			임신성고혈압 임부 간호		‘임신오조(추가), 임신성고혈압 임부 간호’
			내과적 임신 건강문제가 있는 임부 간호(임신성 당뇨, 갑상선기능장애, 심장질환, 빈혈, 성전파성질환, 비뇨기질환, TORCH 감염)		
			분만과정 관련 건강문제가 있는 산부 간호 (난산, 급속분만, 지연분만, 조기진통 자궁파열, 자궁내번증, 태반유착, 다태분만, 과숙아분만, 조기분만)	분류가 51번이 아니라, 52번에 속해야 됨. 고위험 산부간호임	'51 고위험 임부간호'가 아니라, '52 고위험 산부간호'에 포함되어야 함

대분류	No	신규간호사 직무항목 (Activity Statement 개정안)	지식항목 (Knowledge statement)	1차 의견	2차 의견
	52	고위험 산부간호	태아부속물 관련 건강문제가 있는 산부간호(양수과다증, 양수과소증, 조기파막, 제대탈출)		
			대안적분만 간호(유도분만, 흡입분만, 제왕절개분만)		

5영역. 생리적 통합유지

대분류	No	신규간호사 직무항목 (Activity Statement 개정안)	지식항목 (Knowledge statement)	1차 의견	2차 의견
V. 생리적 통합 유지	77	태아질식 증상과 징후 사정 및 간호	신생아 호흡 특성, 호흡기계 건강사정(정상, 비정상 구분), 고위험신생아 호흡기계 관련장애(호흡곤란증후군, 무호흡, 태변흡인, 기관지폐형성이상, 미숙아망막병)	77 태아질식 증상과 징후 사정과 간호의 내용이라는 이름은 모성의 contents이나, 포함된 지식항목은 고위험 신생아에 대한 내용이라서 맞지 간호사직무항목 명칭과 지식항목 내용이 맞지 않는다고 생각됨	77 태아질식 증상과 징후 사정과 간호의 내용이라는 이름은 모성의 contents이나, 포함된 지식항목은 고위험 신생아에 대한 내용이라서 맞지 간호사직무항목 명칭과 지식항목 내용이 맞지 않으므로, 내용을 수정하거나, 범주명칭을 수정해야 함
	95	생식기 질환/생식기 건강문제를 가진 대상자 간호	생식작용과 호르몬, 월경주기, 월경장애의 종류(무월경, 기능성 자궁출혈, 월경전증후군, 월경곤란증), 정의, 원인, 증상 및 징후, 치료방법	월경장애에 해당하는 무월경과, 기능성 자궁출혈이 이중으로 제시되어 있어서 정리가 필요함	초록색은 40번에 있는 것이 위치상 더 적절하여 '40. 생식기 건강사정' 으로 이동하고 여기서는 삭제 '생식작용과 호르몬(삭제), 월경주기(삭제), 월경장애의 종류(무월경, 기능성 자궁출혈, 월경전증후군, 월경곤란증), 정의, 원인, 증상 및 징후, 치료'
			무월경, 무월경 치료 및 간호	중복	중복이어서 삭제
			기능성 자궁출혈의 치료 및 간호	중복	중복이어서 삭제

			자궁의 구조 및 기능, 여성 생식기 건강사정	‘40. 생식기 건강사정'과 내용이 중복됨	'40. 생식기 건강사정'과 내용이 중복되므로 삭제
			자궁내막증, 자궁선근증, 자궁내막증식증, 자궁내막폴립 간호		
			자궁탈수, 자궁전방전위, 생식기누공 간호		
			복압성 요실금 간호		
			난(불)임의 정의 및 진단, 원인, 치료 과정		
			대상 기관의 구조 및 기능, 건강사정, 전파경로별 격리주의, 의료폐기물관리		
			여성생식기의 구조 및 기능, 건강사정, 외음의 감염성 질환		앞부분에 있으므로, 여기서는 삭제
			외음의 감염성 질환, 세균성 질염, 트리코모나스 질염, 캔디다성 질염, 노인성 질염, 자궁경부염, 난관염, 골반감염 간호		
	108	(법정) 감염질환자의 전파예방 및 간호	성전파성 질환(후천성면역결핍증, 단순포진 바이러스성 질환, 인유두종 바이러스성 질환, 매독 세균성 질환, 임질 세균성 질환, 클라미디아 세균성 질환) 간호		
			아동기 면역의 특성		
			아동기 호발 전파성질환(수두, 풍진, 홍역, 볼거리, 일본뇌염, 돌발피진(장미진), 성홍열, 손발입병, 파상풍, 디프테리아, 백일해)		
			질병의 자연사와 예방수준		
			집단면역		
			감염성 질환의 잠복기		
			질병발현의 역학적 요인		

			역학적 측정지표(유병률, 사망률)		
			진단검사 및 역학적 연구		
			법정전염병의 정의, 종류, 지정 목적 및 관리방법		

8영역. 건강증진 및 유지

대분류	No	신규간호사 직무항목 (Activity Statement 개정안)	지식항목 (Knowledge statement)	1차 의견	2차 의견
VIII. 건강 증진 및 유지	127	고위험 건강 행위의 예방 및 치료에 대한 정보 제공(금연, 안전한 성행위, 바늘교환)	만성질환관리		
			영유아보건사업		
			여성보건사업		
			노인보건사업		
			맞춤형 방문간호사업		
			가족계획	가족계획의 분류 역시 127번이 맞을지 의문이 듬	
			성의 개념과 성건강	성건강의 분류가 고위험 건강행위 예방으로 들어간 것이 맞는지 의문이 듬	
	128	성건강 증진간호	피임의 원리와 유형		
	129	문화간호	우리나라 가족	다문화 출산기 여성 간호 포함?	
			다문화사회의 건강행태와 신념		
			문화적 특성에 따른 건강과 질병, 다문화가족의 건강특성		

【부록6】 간호사 국가시험 출제모형에 대한 아동간호학회 의견

1영역. 간호관리와 전문성 향상

대분류	No	신규간호사 직무항목 (Activity Statement 개정안)	지식항목 (Knowledge statement)	2차 의견
I. 간호 관리와 전문성 향상	10	간호전문직 윤리 준수와 역할	간호윤리, 생명의료윤리 원칙, 윤리이론 및 윤리적 사고	
			한국간호사 윤리강령	
			연구 윤리	
			전문직 협력관련 간호윤리	
			자원분배 관련 간호윤리	
			간호전문직관	
			간호사의 임무와 역할	
			소수집단 인권보호 및 윤리적 간호	아동, 요보호아동, 다문화가족 아동
			정신건강복지법	
			공중보건관리 법률	
	13	환자의 권리와 책임에 관하여 대상자에게 교육 제공	간호와 치료에 관한 환자권리 및 책임	
			간호사의 법적 의무와 책임	
			환자권리존중 및 보호	아동, 요보호아동, 학대아동
	21	취약가족에 필요한 지역사회 자원활용	취약가족과 간호, 가족의 개념	한부모가정 아동, 조손가정 등 요보호아동
			가족생활주기의 특성	영아-학령기 가족
			가족관련이론	
			가족간호과정	

2영역. 안전과 감염관리

대분류	No	신규간호사 직무항목<br>(Activity Statement 개정안)	지식항목<br>(Knowledge statement)	2차 의견
II.<br>안전과<br>감염<br>관리	23	안전한 환경 제공	사생활유지관리	
			환경관리 및 환자안전관리	
			환자안전사고 예방(관련법 포함)	
			환자안전사고 보고체계 및 사고기록	
			환자안전문제 발생 시 대응방안 및 운영체계	
			조직의 환자안전문화	
			보안계획(신생아실 안전, 폭력, 통제된 접근) 및 절차 준수	
			안전사고 유형, 위험요인과 예방	아동 안전
			낙상예방 및 관리	아동 안전사고
			안전사고 시 기록	
			억제대 적응증, 억제대 종류, 억제대 적용 시 주의점, 적용방법	

【부록7】 간호사 국가시험 출제모형에 대한 지역사회간호학회 의견

1영역. 간호관리와 전문성 향상

<table>
<tr><th>대분류</th><th>No</th><th>신규간호사 직무항목
(Activity Statement 개정안)</th><th>지식항목
(Knowledge statement)</th><th>1차 의견</th><th>2차 의견</th></tr>
<tr><td rowspan="18">I.
간호
관리와
전문성
향상</td><td>1</td><td>인수인계시행</td><td>업무 인수인계</td><td></td><td></td></tr>
<tr><td>2</td><td>법적 실무범위 내에서 간호 수행</td><td>간호표준실무, 간호관리표준실무</td><td>간호표준실무</td><td></td></tr>
<tr><td>3</td><td>기록 시 표준화된 약어 사용</td><td>간호기록 시 표준약어</td><td></td><td></td></tr>
<tr><td rowspan="3">4</td><td rowspan="3">지침에 따라 간호기록</td><td>간호기록지침</td><td></td><td></td></tr>
<tr><td>환자 개인정보 보호</td><td></td><td></td></tr>
<tr><td>간호정보시스템</td><td></td><td></td></tr>
<tr><td rowspan="3">5</td><td rowspan="3">입원, 전동, 퇴원</td><td>입원관리, 퇴원관리 및 교육, 전과전동업무</td><td></td><td></td></tr>
<tr><td>입퇴원, 전과전동시 인수인계</td><td></td><td></td></tr>
<tr><td>고위험 환자관리체계</td><td></td><td></td></tr>
<tr><td>6</td><td>장비를 적절하고 안전하게 사용</td><td>물품관리</td><td>학교보건실, 산업장 건강관리실 관리</td><td>좌동</td></tr>
<tr><td rowspan="2">7</td><td rowspan="2">간호단위 물품교환체계에 따른 물품관리</td><td>약품관리</td><td>학교보건실, 산업장 건강관리실 관리</td><td>좌동</td></tr>
<tr><td>마약 및 고위험 약품관리</td><td></td><td></td></tr>
<tr><td rowspan="4">8</td><td rowspan="4">질 향상(QI) 활동에 참여</td><td>의료의 질관리,
간호의 질관리 및 질향상활동</td><td></td><td>보건사업 질 관리 및 질 향상 활동</td></tr>
<tr><td>질관리 전략 및 절차</td><td></td><td>보건사업 질 관리 전략 및 절차</td></tr>
<tr><td>의료기관인증제도 및 인증기준</td><td></td><td></td></tr>
<tr><td>질관리 평가기준 및 도구</td><td></td><td></td></tr>
</table>

<table>
<tr><th>대분류</th><th>No</th><th>신규간호사 직무항목
(Activity Statement 개정안)</th><th>지식항목
(Knowledge statement)</th><th>1차 의견</th><th>2차 의견</th></tr>
<tr><td rowspan="25"></td><td></td><td></td><td></td><td></td><td>보건사업 질 관리 평가기준 및 도구</td></tr>
<tr><td rowspan="2">9</td><td rowspan="2">간호사업 평가 관련 업무수행(도구개발, 자료조사, 분석, 비교 및 사업 개선)</td><td>간호사업평가</td><td>보건사업 평가</td><td>좌동</td></tr>
<tr><td>간호관리 통제기능</td><td></td><td></td></tr>
<tr><td rowspan="11">10</td><td rowspan="11">간호전문직 윤리 준수와 역할</td><td>간호윤리, 생명의료윤리 원칙, 윤리이론 및 윤리적 사고</td><td></td><td></td></tr>
<tr><td>한국간호사 윤리강령</td><td></td><td></td></tr>
<tr><td>연구 윤리</td><td></td><td></td></tr>
<tr><td>전문직 협력관련 간호윤리</td><td></td><td></td></tr>
<tr><td>자원분배 관련 간호윤리</td><td>자원분배 관련 간호윤리(형평성 등)</td><td>좌동</td></tr>
<tr><td>간호전문직관</td><td></td><td></td></tr>
<tr><td>간호사의 임무와 역할</td><td></td><td></td></tr>
<tr><td>소수집단 인권보호 및 윤리적 간호</td><td>취약계층(한부모, 장애인, 다문화 가족 등)</td><td>좌동</td></tr>
<tr><td>정신건강복지법</td><td></td><td></td></tr>
<tr><td>공중보건관리 법률</td><td>공중보건관리 법률(지역보건법, 건강증진법, 감염병 예방 및 관리에 관한 법률 등)</td><td>좌동</td></tr>
<tr><td colspan="0" style="display:none"></td></tr>
<tr><td rowspan="7">15</td><td rowspan="7">일차의료기반의 보건간호</td><td>일차보건의료</td><td>일차보건의료(철학, 전략, 필수서비스, 발전과정)</td><td>좌동</td></tr>
<tr><td>지역사회간호의 정의</td><td></td><td></td></tr>
<tr><td>지역사회와 건강개념</td><td></td><td></td></tr>
<tr><td>건강의 결정요인</td><td></td><td></td></tr>
<tr><td>국제보건기구의 간호정책 및 활동</td><td></td><td></td></tr>
<tr><td>보건의료체계의 이해</td><td>보건의료체계의 이해(보건의료체계 구성요소, 보건의료자원, 보건의료조직, 보건의료 재원조달, 주요 국가별 보건의료체계)</td><td>좌동</td></tr>
<tr><td>진료비 지불보상제도</td><td></td><td></td></tr>
<tr><td rowspan="3">16</td><td rowspan="3">지역사회 간호사업의 법적 기준 및 지침에 따른 활동 참여</td><td>보건관리자의 역할 및 업무</td><td>보건관리자의 역할 및 업무, 보건소 업무</td><td>좌동</td></tr>
<tr><td>산업전문간호사의 역할</td><td></td><td></td></tr>
<tr><td>보건/간호사업 기획 및 수행</td><td>지역사회간호이론, 지역사회간호과정, 보건/간호</td><td>좌동</td></tr>
</table>

대분류	No	신규간호사 직무항목 (Activity Statement 개정안)	지식항목 (Knowledge statement)	1차 의견	2차 의견
				사업기획 및 수행, 보건사업 기획, 보건정보 및 기술의 활용(GIS시스템, 보건간호의 EDI)	
			보건교육자, 보건진료원의 역할	보건교육자, 보건전문간호사, 보건진료원의 역할	좌동
			보건정보 및 기술계획		
			건강증진 종합계획	건강증진 종합계획, 지역보건의료계획	좌동
			간호사업의 법적 기준 및 지침	간호사업의 법적 기준 및 지침, 보건소, 보건지소, 보건진료소 기능 비교	좌동
	17	사례관리활동 참여	사례관리 정의 및 원칙		
			조정과 협력		
			간호관리 체계 및 과정		
			사례관리모델, 사례관리과정		
	18	업무를 조직화하여 일을 효율적으로 관리	간호관리업무, 역량	기획 원칙 및 구성요소, 기획단계	
			관리이론		
			기획 원칙 및 구성요소, 기획단계		
			계획안과 목표관리		
			의사결정		
			예산관리		
			조직화 원리, 조직구조 및 권한		
			직무설계, 직무분석, 직무평가		
			간호전달체계		
			간호조직문화와 조직변화		
			간호인적자원 관리		
	19	환자분류체계 관련 정보수집 및 활용	환자분류체계	사업대상분류체계(방문우선순위,사업우선순위 등)	좌동
			간호업무량 및 인력산정, 간호업무 배정		

3영역. 위험요인 사정

대분류	No	신규간호사 직무항목<br>(Activity Statement 개정안)	지식항목<br>(Knowledge statement)	1차 의견	2차 의견
III.<br>위험<br>요인<br>사정	26	간호계획, 진료지침 수행	간호과정	간호과정(계획, 수행, 평가)	좌동
	27	대상자의 건강문제에 대해 우선순위 결정	건강문제의 우선순위 결정		

5영역. 생리적 통합유지

대분류	No	신규간호사 직무항목<br>(Activity Statement 개정안)	지식항목<br>(Knowledge statement)	1차 의견	2차 의견
V.<br>생리적<br>통합<br>유지	107	재활간호서비스	재활간호	지역사회 재활(정의, 필요성, 목적, 역할, 수행)	좌동
			정신사회재활		
	108	(법정) 감염질환자의 전파예방 및 간호	대상 기관의 구조 및 기능, 건강사정, 전파경로별 격리주의, 의료폐기물관리		
			여성생식기의 구조 및 기능, 건강사정, 외음의 감염성 질환		
			외음의 감염성 질환, 세균성 질염, 트리코모나스 질염, 캔디다성 질염, 노인성 질염, 자궁경부염, 난관염, 골반감염 간호		
			성전파성 질환(후천성면역결핍증, 단순포진 바이러스성 질환, 인유두종 바이러스성 질환, 매독 세균성 질환, 임질 세균성 질환, 클라미디아 세균성 질환) 간호		
			아동기 면역의 특성		

대분류	No	신규간호사 직무항목 (Activity Statement 개정안)	지식항목 (Knowledge statement)	1차 의견	2차 의견
			아동기 호발 전파성질환(수두, 풍진, 홍역, 볼거리, 일본뇌염, 돌발피진(장미진), 성홍열, 손발입병, 파상풍, 디프테리아, 백일해)		
			질병의 자연사와 예방수준		
			집단면역	집단면역, 감염병 예방	
			감염성 질환의 잠복기		
			질병발현의 역학적 요인	질병발현의 역학적 요인, 법정감염병(정의, 종류, 관리법), 역학의 정의와 목적, 질병의 역학 모형	질병발현의 역학적 요인, 법정감염병 (정의, 종류, 관리법)
			역학적 측정지표(유병률, 사망률)		
			진단검사 및 역학적 연구,	진단검사 및 역학적 연구, 보건통계	좌동
			법정전염병의 정의, 종류, 지정 목적 및 관리방법		

7영역. 심리사회적 통합유지

대분류	No	신규간호사 직무항목 (Activity Statement 개정안)	지식항목 (Knowledge statement)	1차 의견	2차 의견
VII. 심리 사회적 통합 유지	121	정신질환자 간호	조현병 장애의 정의, 관련요인, 행동특성 및 조현병 장애 환자 간호		
			기타 정신병적 장애의 정의, 유형(조현양상장애, 단기 정신병적 장애, 조현정동장애, 망상장애 등), 행동특성 및 기타 정신병적 장애 환자 간호		

대분류	No	신규간호사 직무항목<br>(Activity Statement 개정안)	지식항목<br>(Knowledge statement)	1차 의견	2차 의견
			우울장애의 정의, 유형(주요우울장애, 기분저하장애), 관련요인, 행동특성 및 우울장애 환자 간호		
			양극성 및 관련장애의 정의, 유형(제1형 양극성장애, 제2형 양극성장애, 순환성장애), 관련요인, 행동특성 및 양극성장애 환자 간호		
			불안 정도 사정 및 불안 수준별 간호		
			불안장애의 정의, 유형(공황장애, 광장공포증, 특정공포증, 범불안장애, 사회불안장애), 관련요인, 행동특성 및 불안장애환자 간호		
			강박 및 강박 관련 장애의 개념, 유형(강박장애, 신체변형장애 등), 관련요인, 행동특성 및 강박 및 강박 관련 장애 환자 간호		
			외상 및 스트레스 관련 장애의 개념, 유형(외상후 스트레스장애, 적응장애 등), 관련요인, 행동특성 및 외상 및 스트레스 관련 장애 환자 간호	PTSD, 이재민 관리	좌동
			신체증상 및 관련 장애의 정의, 유형(신체증상장애, 전환장애, 질병불안장애, 인위성장애), 관련요인, 행동특성 및 신체증상 및 관련 장애 환자 간호		
			해리장애의 정의, 유형(해리성 정체성장애, 해리성 기억상실, 이인성/비현실감 장애), 관련요인, 행동특성 및 해리장애 환자 간호		
			인격장애의 정의, 유형(편집성, 조현성, 조현형, 반사회성, 경계성, 연극성, 자기애성, 의존성, 강박성, 회피성 인격장애), 관련요인, 정신역동, 행동특성 및 인격장애 환자 간호		

8영역. 건강증진 및 유지

대분류	No	신규간호사 직무항목 (Activity Statement 개정안)	지식항목 (Knowledge statement)	1차 의견	2차 의견
VIII. 건강 증진 및 유지	125	(지역사회)건강교육계획	역학의 정의와 목적, 질병의 역학모형		
			보건교육 개념과 보건교육	보건교육 개념과 보건교육, 보건교육자의 역할, 보건교육 대상별 특성, 보건교육 현장	좌동
			학습이론, 건강행위이론, 보건교육 관련이론		
			보건교육, 학교보건교육	보건교육, 학교보건교육, 보건교육 프로그램 개발(보건교육 요구, 학습목표, 내용선정 및 조직, 보건교육방법, 보건교육매체), 보건교육수행(보건교육계획안 작성, 학습지도 원칙, 의사소통 기술, 상담기술), 보건교육평가(평가도구 개발, 재계획에 반영)	좌동
	126	건강증진 및 유지관리에 대한 정보(예방접종 등) 제공	정신건강증진의 개념, 취약집단 및 정신건강증진사업		
			개인/가족/지역사회/인구집단의 건강위험		
			건강증진요인 및 위험요인	건강증진요인 및 위험요인(생애주기별), 건강증진개념, 건강증진모형	좌동
			인구구조의 유형별 특징		
			한국의 인구정책		
			생활습관개선을 위한 건강증진사업	생활습관개선을 위한 건강증진사업, 지역사회와의 파트너쉽 시도, 대상자 역량강화, 다차원적 접근, 건강증진 적용 현장	좌동
	127	고위험 건강 행위의 예방 및 치료에 대한 정보 제공 (금연, 안전한 성행위, 바늘교환)	만성질환관리	만성질환관리(특성, 관리방법)	좌동
			영유아보건사업	영유아보건사업(중요성,영유아집단 특성, 보건지표, 지역사회사업 종류, 간호사업 기획)	좌동
			여성보건사업	여성보건사업(중요성,여성인구집단 특성, 보건지표, 지역사회사업 종류, 고위험임산부 지원사업, 간호사업 기획)	좌동

대분류	No	신규간호사 직무항목 (Activity Statement 개정안)	지식항목 (Knowledge statement)	1차 의견	2차 의견
			노인보건사업	노인보건사업(노인보건복지기관, 서비스 및 인력, 노인장기요양보험제도)	좌동
			맞춤형 방문간호사업	맞춤형 방문간호사업 (정의, 목적, 대상 및 역할)	좌동
			가족계획		
			성의 개념과 성건강		
	128	성건강 증진간호	피임의 원리와 유형		
	129	문화간호	우리나라 가족		
			다문화사회의 건강행태와 신념		
			문화적 특성에 따른 건강과 질병, 다문화가족의 건강특성		
	130	산업과 환경 간호	산업재해관리, 작업환경관리	산업재해 관리, 작업환경관리(원인과 종류, 예방대책, 관련 통계, 유관기관)	좌동
			환경보건, 환경건강정보	환경보건(공기오염, 기후변화, 수질오염, 식품, 주거환경, 폐기물, 방사능), 환경건강정보(개념, 정책, 유해인자 및 관리), 간호사의 역할	좌동
			지역사회/산업장/가정 환경사정		
			risk communication의 원칙		
			근로자 건강진단	근로자 건강진단과 질환 예방 및 관리	좌동
			작업관련성 질환의 예방 및 관리		
			산업보건과 산업간호	산업보건과 산업간호의 개념, 인력 및 역할	좌동
	131	재난간호	공중보건상의 재난 시 지켜야할 원칙의 적용과 활용	공중보건상의 재난 시 지켜야할 원칙의 적용과 활용(이재민관리, 수용시설 관리, PTSD. 감염병 관리)	공중보건상의 재난 시 지켜야할 원칙의 적용과 활용(이재민관리, 수용시설 관리, PTSD)

대분류	No	신규간호사 직무항목 (Activity Statement 개정안)	지식항목 (Knowledge statement)	1차 의견	2차 의견
			공중보건법	재난예방(관련 법, 재난유형별, 단계별 대응.대비체계, 윤리적 원칙)	재난예방(관련 법, 재난유형별 대응체계, 단계별 대응, 윤리적 원칙)
			위기/재난 시 의사소통체계		
			국제재난구조단체	국제재난구조단체(필요성, 활동, 간호사의 역할)	좌동
	132	치료계획을 결정하기 위해 가족 역동성(가족구조, 결속, 의사소통, 경계, 대처기전) 사정	가족역동성(family dynamics) 사정		
	133	가정환경에서 대상자를 관리할 수 있는 역량 (장비, 지역사회 자원) 평가	지역사회 자원 평가	지역사회 자원평가, 지역사회 간호활동(건강관리실 운영방법, 가정방문 활동원리, 자원활용계획, 상담원리, 매체의 종류별 장단점, 지역사회조직 활용, 상황에 따른 지역사회 간호수단 선택)	좌동
	134	건강위험요인 사정과 교육	학교건강검사	학교건강검사, 학교건강증진프로그램 운영	좌동
			학교보건교육	학교보건교육(계획,수행,평가),학교보건법, 학교보건인력, 보건실 운영(인력, 예산, 약물, 기록 등), 성건강과 학교폭력 관리	좌동
			지역사회 정신보건		

【부록8】 간호사 국가시험 출제모형에 대한 7개 회원학회 의견

(단위: 개)

대분류	신규간호사 직무항목 (134개)									지식항목(481개 범주)								
	직무 항목	관리	기본	모성	성인	아동	정신	지역	계	지식 항목	관리	기본	모성	성인	아동	정신	지역	계
Ⅰ. 간호관리와 전문성 향상	**22**	20	-	3	-	3	8	17	51	**79**	58	-	3	-	4	9	53	127
Ⅱ. 안전과 감염관리	**3**	3	2	-	1	1	3	2	12	**25**	17	16	-	2	2	12	13	62
Ⅲ. 위험요인 사정	**28**	-	2	17	10	13	8	5	55	**93**	-	3	48	20	48	13	6	138
Ⅳ. 기본간호	**19**	-	11	-	14	12	3	-	40	**60**	-	42	-	32	42	8	-	124
Ⅴ. 생리적 통합 유지	**36**	-	4	4	32	25	2	2	69	**130**	-	16	15	79	85	4	9	208
Ⅵ. 약물 및 비경구 요법	**7**	-	5	1	1	2	3	2	14	**14**	-	10	1	1	5	3	8	28
Ⅶ. 심리적 통합 유지	**9**	-	-	2	1	4	9	2	18	**43**	-	-	2	4	9	40	2	57
Ⅷ. 건강증진 및 유지	**10**	-	-	2	-	4	2	9	17	**37**	-	-	3	-	10	4	36	53
합계	134	23	24	29	59	64	38	39	276	481	75	87	72	138	205	93	127	797

【부록9】 공청회에서 제기된 전문가 의견

출제모형의 방향성

- 간호사 면허 취득을 위한 국가시험의 방향이 '전공교과목의 지식수준 평가'에서 '실무에서 요구되는 신규간호사의 역량 평가'로 방향이 전환되어야 한다.
- 간호사 국가시험 방향 변화는 대학교육의 방향 변화에 영향을 미치므로 간호표준, 간호교육인증 평가 기준 등과 일관성을 유지하면서 이를 위한 로드맵을 제시한다.
- 국가시험은 보건의료환경변화에 따른 21세기 간호사에게 기대되는 역량을 제대로 평가할 수 있어야 한다. 최근 우리나라 보건의료환경변화의 가장 큰 화두는 인구고령화(특히 80세 이상 노인의 빠른 증가), 다양성, 감염병 관리 등 인구집단 건강관리 및 간호사 활동영역의 다양성이다.

신규간호사 핵심역량		
**대한간호협회(2019)**	**한국간호교육평가원(2020)**	**AACN(2021)***
환자간호	지식과 간호술의 통합	Knowledge for Nursing Practice
의소통과 협력	비판적 사고	Person-Centered Care
환자안전과 질 향상	의사소통	Population Health
의료정보 활용	리더십	Scholarship for Nursing Practice
전문성과 리더십	안전관리	Quality and Safety
	국제화	Interprofessional Partnerships
		Systems-Based Practice
		Information and Healthcare Technologies
		Professionalism
		Personal, Professional, and Leadership Development

*AACN: American Association of Colleges of Nursing

- 우리나라는 2019년 노인인구가 전체 인구의 14.9%로 고령사회가 되었으며 전 세계에서 가장 빠른 고령화가 진행되고 있는 현실적인 상황을 고려해 볼 때, 국가시험 출제모형에 따른 출제기준안이 병원뿐만 아니라, 노인요양시설, 보건소, 노인복지센터, 주간보호센터, 방문간호서비스, 지역사회 노인 통합돌봄 등 다양한 노인간호 서비스 분야에 종사할 신규간호사의 역량을 포괄적으로 포함하고 있는가에 대한 검토가 요구된다.
- 노인간호 분야의 중요성이 부각 되고 있는 현실적인 상황이 출제기준에 고려되어야 하므로, 출제기준의 시의성, 객관성, 신뢰성을 위해 현행 출제기준에서 노인간호 역량을 평가할 수 있는 방법을 모색하여야 한다.

- 미국 AACN에서는 2010년에 고령사회에 대비하기 위한 간호사의 노인간호역량 강화를 위해 19개의 노인간호핵심역량을 2008년 발표한 간호표준에 더하여 추가로 제시하였고 이를 간호학 교육과정에 반영하도록 하였으며, 2021년 4월 발표한 간호표준에서도 간호사의 다양한 활동 분야를 고려하는 것이 중요함을 강조하면서 급성기 병원뿐만 아니라 장기요양기관을 포함한 지역사회의 다양한 간호실무 현장에서 역할을 수행하기 위한 간호사역량을 제시하고 있다.
- 지필시험으로 평가하기에는 한계가 있는 핵심 간호술기를 평가할 수 있는 실기시험 평가방법을 구체적으로 모색하여 직무중심 평가를 통해 우수 간호인력 배출에 기여한다.
- 향후에는 1년에 한번 시행하는 지문식(paper-pencil)시험제도에서 컴퓨터를 활용한 시스템(CAT: Computer Adaptive Testing)으로 전환하는 것도 고려한다.
- 21세기 한국 간호사의 역량을 제대로 평가하기 위한 국가시험 출제기준에 대한 논의를 위한 것이라면, 현행 국가시험 교과목의 틀에서 벗어나 다양한 분야의 의견을 수렴하여 출제기준에 반영한다.
- 국가시험 문제공개 후 출제문제 오류를 최소화하고 정답 논란의 여지를 피하기 위해 문제해결형 문제의 난이도가 점점 쉬워지거나 변별력이 떨어지는 현상이 나타난다. 이에 대한 해결방안은 문제은행에 좋은 문항을 확보하는 것이므로 문항개발, 정리 및 검토 작업에 국시원의 적극적인 지원이 필요하다.
- 직무기반 통합형 간호사 국가시험 출제모형에 대한 합의된 안을 도출하기 위해서는 이해당사자의 충분한 논의와 의견이 반영되고 공유될 수 있어야 한다. 따라서 한국간호과학회 회원학회와 학과(장)협의와 병원간호사회 등 유관 간호단체로부터 추천되어 구성된 전문가 TF를 구성하여 공동 주관의 연구가 필요하다.
- 직무에 기반한 통합형 표준 학습목표에 따른 국가시험 문제 출제에는 동의하지만, 간호학의 교육과정 통합은 우선적으로 선행되어야 할 과제들을 해결하면서 단계적인 절차를 거쳐서 진행되어야 한다.

출제모형 개발 절차의 정당성
• 절차의 정당성을 확보하기 위해서는 8개 직무영역 134개 신규간호사 직무항목 및 481개 범주의 지식항목에 대한 충분한 의견수렴 과정과 신뢰성과 타당성 검증절차가 확인되어야 한다.

출제관리 시스템
• 간호사 국가시험 질 향상을 위한 지속적 시스템 향상의 필요성을 국시원에 적극 개진한다.<br>• 통합형의 문제해결형 문항을 개발하기 위해서는 임상전문가, 교육전문가 대상의 지원체계가 구비되어야 하고 문항의 질 관리를 위한 검증장치가 구축되어야 한다.<br>• 문항개발을 위한 인프라를 구축하여, 우선적으로 난이도 안정화를 위한 방안을 마련한다.<br>• 현재는 7개 국가시험 교과목별로 문제 출제위원 인력 풀을 구성하여 진행하는데, 통합형 간호사 국가시험 문제 출제위원 인력 풀 구성 등의 방법론에 대한 로드맵을 구체적으로 제시한다.<br>• 국가시험 문제가 공개됨에 따라 기존의 공개되지 않은 문제가 별로 남아 있지 않은 상태에서 문제은행에 확보되어야 할 문제해결형 문제, 암기형 문제, 해석형 문제의 비중에 대한 구체적인 전략을 수립한다.<br>• 간호실무의 실제성과 최신성을 반영하는 문항을 개발하고 개정하기 위해서는 실무현장의 전문가들과 교육현장 전문가들의 합의 과정과 지속적인 검토 시스템 구축이 필요하다.<br>• 문항개발 과정에 출판된 국가시험 전공교과목 교재뿐만 아니라 임상 매뉴얼, 연구논문, 의료기관 인증기준집 등 직무에 기반한 참고문헌을 활용한다.<br>• 간호 상황에 적합한 임상판단을 할 수 있는 통합적 사고 능력을 평가하기 위해서는 임상 및 지역사회에서 실무경험이 많은 퇴직 간호사들을 적극 활용하여 국시원의 재정적 지원 하에 사례 중심의 문제해결형 문항을 개발한다.<br>• 간호사 국가시험 문항개발, 문제출제 및 연구를 수행할 때 대학 교수 뿐만 아니라 임상 및 지역사회 실무전문가가 공동책임자로 참여한다.

출제모형의 8개 직무영역
• 8개 직무영역의 영역별 정의 및 영역별 목표를 제시한다.<br>• Ⅰ.간호관리와 전문성 향상, Ⅱ.안전과 감염관리, Ⅶ.심리사회적 통합유지, Ⅷ.건강증진 및 유지는 영역이 명확하게 구분되나, Ⅲ.위험요인 사정, Ⅳ.기본간호, Ⅴ.생리적 통합유지, Ⅵ.약물 및 비경구요법은 영역의 구분이 명확하지 않다.<br>• 8개의 직무영역이 어떤 배열순서(sequence)를 가지는지 체계 정리가 필요하고, 특히 중복이 많은 Ⅲ. 위험요인 사정과 Ⅳ. 기본간호 및 Ⅴ. 생리적 통합유지는 명확한 개념 정의가 필요하다.

출제모형의 134개 직무항목

- 직무항목이 상호 배타적이어야 하는데 중복되는 항목이 있고, 구체성 수준이 일관되지 않으므로 교육, 임상 및 지역사회 전문가 집단의 의견수렴과 합의 과정을 거쳐서 재정리한다.

- Ⅲ. 위험요인 사정, Ⅳ. 기본간호 및 Ⅴ. 생리적 통합유지 영역과 연계된 직무항목에 대해 성인간호학, 모성간호학, 아동간호학 교과목간 교육, 임상 및 지역사회 전문가 집단의 의견수렴 과정과 합의가 필요하다.

- 8개 직무영역과 연계된 직무항목에서 중복된 직무항목에 대한 교육, 임상 및 지역사회 전문가 집단의 의견수렴과 합의 과정을 거쳐서 재정리 한다.

직무영역	직무항목 중복		
Ⅲ	29 신생아간호	42 신생아 건강사정 및 간호	43 고위험신생아 건강사정 및 간호
Ⅲ	48 산전간호와 교육	51 고위험 임부 간호	
Ⅲ	49 분만중 간호와 교육	52 고위험 산부간호	
Ⅲ	50 산후관리와 교육	53 고위험 산모간호	
Ⅲ	40 생식기 건강사정	46 진단검사 간호	
Ⅴ	73 호흡기능장애에 대상자 간호		74 호흡증진 중재
Ⅲ,Ⅴ	47. 수술간호		89 심장수술 후 간호
Ⅲ,Ⅴ	37. 성건강간호	40, 생식기 건강사정	95.생식기질환을 가진 대상자 간호
Ⅳ,Ⅴ	61. 배뇨장애 대상자사정 및 간호		91. 배뇨장애를 가진 대상자 간호

- 134개 직무항목에 연계된 관련 교과목에 대한 재검토가 요구된다.

직무영역	직무항목	비고
Ⅵ	109, 110, 111, 112, 113, 115	성인간호학 연계성 검토
Ⅵ	115	관련된 연계 교과목 검토
Ⅷ	129	아동간호학 연계성 재검토

481개 범주 지식항목

- 134개 신규간호사 직무항목과 연계된 481개 범주의 지식항목이 범주별로 구체성 수준에 차이가 있으므로 지식항목 구체성 수준을 교육, 임상 및 지역사회 전문가 집단의 의견수렴과 합의 과정을 거쳐서 재정리한다.
- 지식항목이 간호실무 현장의 실무를 구체적으로 시의적절하게 반영하기 위해서는 교육전문가 뿐만 아니라 임상 및 지역사회 실무전문가의 참여하에 134개 직무항목과 연계된 지식항목을 도출한다.
- 전공 교과목과 연계되지 않은 지식항목 및 2개 이상의 전공 교과목과 연계된 지식항목에 대한 교육, 임상 및 지역사회 전문가 집단의 의견수렴 과정과 합의가 필요하다.
- 범주별 지식항목에서 수정, 삭제 및 추가할 항목에 대한 교육, 임상 및 지역사회 전문가 집단의 의견수렴과 합의 과정을 거쳐서 재정리 한다.

직무영역	직무항목	지식항목		
		삭제	수정	비고
Ⅰ	7	약품관리	멸균물품 관리	7 직무항목과 연계된 지식항목이 112, 115 직무항목과 중복
		마약 및 고위험 약품관리	진료재료 재고 관리	
Ⅱ	25	고위험 약품 및 마약관리	유해 화학물질 목록 및 안전관리절차	25 직무항목과 연계된 지식항목과 115 직무항목 중복

직무영역	직무항목	지식항목	
		통합	비고
Ⅰ	14	신체억제대 사용지침, 억제대 사용관련 윤리적 사고 및 결정	14 직무항목에 연계된 지식항목 또는 23 직무항목에 연계된 지식항목으로 통합하여 정리
Ⅱ	23	억제대 적응증, 억제대 종류, 억제대 적용 시 주의점, 적용방법	
Ⅰ	4	간호기록지침	4 직무항목에 연계된 지식항목 또는 26. 직무항목에 통합하여 정리
Ⅲ	26		

직무영역	직무항목	지식항목	
		추가	비고
Ⅵ	115	항암화학제, 고농축약물	

직무영역	직무항목	지식항목	
		전공 교과목에서 선택되지 않은 지식항목	비고
Ⅳ	55	세척(irrigation)(방광, 귀, 눈) 수행	
Ⅴ	108	대상 기관의 구조 및 기능, 건강사정, 전파경로별 격리주의, 의료폐기물관리	
Ⅵ	112	지침에 따라 규제약물을 관리(증인, 잔량폐기), 안전하고 통제된 환경에서 의약품 취급 및 관리	
		투약오류 보고 및 예방	

직무영역	직무항목	지식항목	비고
Ⅰ	9	간호사업평가	간호관리학 연계성 검토
Ⅱ	24	의료관련감염 정의, 종류, 위험요인, 교차감염	성인간호학 연계성 검토

- 현행 국가시험 출제기준에 적용해 보면, 481개 범주의 지식항목에서 275개 문항(보건의약관계법규 20개 문항 포함하지 않음)이 국가시험 문제로 출제되는데, 481개 범주에서 추출한 275개 국가시험 문제 분포의 객관적 정당성을 확보할 수 있는 방안을 모색한다.

- 국가시험 교과목별 문제출제 수와 교과목과 연계된 지식항목의 적절성을 검토한다.

구분	국가시험 교과목						
	기본	관리	모성	성인	아동	정신	지역
지식항목 수	87	75	72	138	205	93	127
출제문제 수	30	35	35	70	35	35	35

* 보건의약관계법규 : 출제문제 20문항 포함 안 됨

- 회원학회 제안을 교육, 임상 및 지역사회 전문가 집단의 의견수렴과 합의 과정을 거쳐서 반영한다.

- 아동간호학 연계 지식항목에 대한 교육, 임상 및 지역사회 전문가 집단의 의견수렴과 합의 과정이 필요하다.

신규간호사의 간호실무현장 적응 현황
• 간호사 국가시험을 치루고 면허를 취득한 신규간호사들이 병원에 입사하면, 충분히 준비되지 못한 상태에서 임상실무 현장에서의 업무수행 과정에 여러 가지 어려움을 겪고 있다.<br>• 특히 요즘 날로 발전하는 의료 기술 및 치료의 복잡성 증가와 간호사의 역할 확대로 학교에서 학습한 이론과 임상에서 요구되는 실무의 격차가 더욱 커지고 있다.<br>• 병원간호사회의 병원간호인력 배치현황 실태조사에서 신규간호사의 1년 이내 사직률은 2017년 42.7%, 2018년 45.5%, 2019년에는 44.5%로 지속적으로 증가하고 있었으며, 이직 사유로는 타 병원으로의 이직, 업무 부적응 순이었다.<br>• 응급실, 중환자실에 근무하는 대부분 신규간호사들은 발령 후 학교에서 배운 내용과 실무의 연결이 어렵다. 실무에서 모르는 것이 많아 답답하다. 배워야 할 일과 과제가 너무 많아 숨이 막힌다. 몸과 마음이 지치고 의지할 곳이 없어 사직하고 싶은 충동을 느낀다. 독립 후 혼자서 업무하는 것이 불안하다고 보고하였다.<br>• 국내 대부분의 병원에서는 신규간호사가 임상 현장에 필요한 역량을 갖추고, 임상 실무에 안정적으로 역할 전이 할 수 있도록 실무적응 지원을 위한 프리셉터십을 운영하고 있다.<br>• 일부 상급종합병원과 종합병원에서는 프리셉터십 외에 신규간호사의 실무적응 지원을 위한 다양한 교육프로그램을 운영하고 있다.<br>• 신규간호사가 수행하기 어려운 간호술기는 응급상황 대처, 인공호흡기 관리, 임종간호, 우선순위 정하기/시관 관리, 심전도 모니터링 순으로 높게 나타났다(권인각 외, 2018).<br>• 학생에서 간호사로 역할을 이행하는 과정에서 경험한 어려움으로는 업무 부담으로 인한 어려움(76.2%), 역할기대로 인한 어려움(53.6%)이었다(권인각 외, 2018).<br>• 상급종합병원 중심의 신규간호사 실무지원 교육프로그램 개발 용역연구(병원간호사회, 2019)에서 교육프로그램을 간호술기, 공통직무교육, 적응지원 및 역량강화교육으로 분류하였다.<br>• 간호술기는 공통핵심술기, 중환자실핵심술기, 부서별 술기로 분류하여 공통핵심술기는 기본간호, 환자사정, 호흡간호, 배뇨/배번간호, 투약간호, 수혈간호, 위관영양, 심폐소생술, 의료기기 및 장비관리로 구성하였고 중환자실 핵심술기는 호흡기간호, 순환기간호, 응급간호, 신경계간호로 구성하였으며, 부서별 술기는 수술 및 시술 간호, 배액관 관리, 피부간호로 구성하였다.<br>• 공통직무교육은 간호전달체계 및 환자분류, 환자분담 및 교대근무, 간호기록 및 간호과정, 감염간호, 환자안전 및 질 향상, 투약 및 약품관리, 수혈간호, 통증간호, 검사간호, 수술/시술간호, 입/퇴원 간호, 응급간호, 항암간호, 말기 및 임종간호, 간호교육, 물품 및 비품관리, 의사소통 및 협력, 처방확인 및 수가관리로 구성하였다.<br>• 적응지원 및 역량강화 교육은 응급상황 대처, 윤리적 의사결정 및 환자경험관리, 갈등관리 및 스트레스 대처, 전문직과 리더십, 근거기반 간호로 구성하였다.<br>• 신규간호사의 역량은 간호의 질, 환자 안전과 관련되어 있어 매우 중요하므로, 간호학생이 신규간호사로서 역할전이를 잘 할 수 있도록 간호사의 직무역량을 평가하는 국가시험으로 방향이 전환되어야 할 필요성이 있다.

【부록10】 한국간호과학회 7개 회원학회 1차 의견조사

국민이 신뢰하고 감동하는 시험평가기관

한국보건의료인국가시험원

수 신 한국간호과학회장

(경유) 한국간호과학회 회원학회장

제 목 2020년도 국시원 자유주제 위탁연구「간호사 국가시험의 통합모형에 근거한 출제기준개발 기초연구」출제기준(안) 개발을 위한 의견 요청

1. 귀 기관의 무궁한 발전을 기원합니다.
2. 한국보건의료인국가시험원은 대한간호협회와 계약을 체결하고 2020년도 자유주제 위탁연구「간호사 국가시험의 통합모형에 근거한 출제기준개발 기초연구」를 진행하고 있습니다.
3. 위 연구와 관련하여, 통합모형에 근거한 간호사 출제기준(안) 개발을 위해 선행연구를 통해 도출된 8개 통합 실무영역 및 134개 신규간호사 실무항목과 연계된 총 481개 범주의 지식항목에 대한 간호학계의 의견을 수렴하고자 합니다.
4. 이에, 동 지식항목과 출제기준(안)에 대한 한국간호과학회 회원학회의 의견을 요청하오니 통합모형에 근거한 출제기준이 원활히 개발될 수 있도록 적극적인 협조를 부탁드립니다.

가. 연구 개요

1) 연구과제명 : 간호사 국가시험의 통합모형에 근거한 출제기준개발 기초연구

2) 주관연구기관 : 사단법인 대한간호협회

3) 책임연구자 : 김경희 교수(중앙대학교)

나. 검토 대상

1) 8개 통합 실무영역 및 134개 신규간호사 실무항목과 연계된 481개 지식항목([붙임 2.] 참조)

2) 직무기반 통합형 간호사 국가시험 출제모형에 근거한 출제기준(안)([붙임 3.] 참조)

다. 회신 방법 : [붙임 1.]의 검토대상 작성법을 참고하여 [붙임 2.] 및 [붙임 3.] 작성 후 아래 회신처로 회신기한 내 회신

라. 회신 기한 : **2021. 3. 19.(금)까지**

마. 회신처 : 한국간호과학회 최금숙 사무국장(kan@kan.or.kr)

바. 문의처 : 책임연구자 김경희 교수(kyung@cau.ac.kr).

붙임 1. 의견조회 안내문 1부.

2. 8개 통합 실무영역 및 134개 신규간호사 실무항목과 연계된 481개 지식항목 1부.

3. 직무기반 통합형 간호사 국가시험 출제모형에 근거한 출제기준(안) 1부. 끝.

한국보건의료인국가시험원장

대리 **정보경** 연구개발부장 **임미경** 연구개발본부장 전결 03/05 **이경신**

협조자

시행 연구개발부-555 (2021.03.05.) 접수 ( )

우 05103 서울특별시 광진구 자양로 45 (자양동) / www.kuksiwon.or.kr

전화 02-2087-8957 /전송 02-2087-8885 / luckys1004@kuksiwon.or.kr / 공개

- 소중한 개인정보 우리가 지켜야 합니다. -

【부록11】한국간호과학회 회원학회 2차 의견조사

국민이 신뢰하고 감동하는 시험평가기관

한국보건의료인국가시험원

수 신 한국간호과학회장

(경유) 한국간호과학회 회원학회장

제 목 2020년도 국시원 자유주제 위탁연구「간호사 국가시험의 통합모형에 근거한 출제기준개발 기초연구」출제기준(안) 개발을 위한 의견 요청 (2차)

1. 귀 기관의 무궁한 발전을 기원합니다.
2. 한국보건의료인국가시험원은 대한간호협회와 계약을 체결하고 2020년도 자유주제 위탁연구「간호사 국가시험의 통합모형에 근거한 출제기준개발 기초연구」를 진행하고 있습니다.
3. 신규간호사 직무기반 통합형 간호사국가시험 출제기준(안) 개발을 위해 8개 통합 실무영역과 연계된 134개 신규간호사 실무항목의 481개 범주 지식항목에 대한 1차 의견수렴 과정에 적극적으로 협조해 주셔서 진심으로 감사드립니다.
4. 앞선 의견조회에서 신규간호사의 직무역량을 평가하기 위해 도출한 481개 범주의 지식항목에 대한 한국간호과학회 회원학회의 1차 의견수렴 결과를 공유해 드리며, 1차 의견수렴 과정에서 선정한 지식항목 중에서 타 회원학회의 의견을 참조하면서 조정과 수정이 필요하다고 생각되는 항목에 대한 재검토를 부탁드립니다.
   가. 연구 개요
      1) 연구과제명 : 간호사 국가시험의 통합모형에 근거한 출제기준개발 기초연구
      2) 주관연구기관 : 사단법인 대한간호협회
      3) 책임연구자 : 김경희 교수(중앙대학교)
   나. 검토 대상
      1) 8개 통합 실무영역 및 134개 신규간호사 실무항목과 연계된 지식항목(붙임1)
      2) 직무기반 통합형 간호사 국가시험 출제모형에 근거한 출제기준(붙임2)
   다. 회신 방법 : [붙임1]과 [붙임2] 작성 후 아래 회신처로 회신기한 내 회신
   라. 회신 기한 : 2021. 5. 14.(금)까지
   마. 회신처 : 한국간호과학회 최금숙 사무국장(kan@kan.or.kr)
   바. 문의처 : 책임연구자 김경희 교수(kyung@cau.ac.kr).

붙임 1. 8개 통합 실무영역 및 134개 신규간호사 실무항목과 연계된 지식항목(481개)_회원학회(2차의견수렴)
2. 직무기반 통합형 간호사 국가시험 출제모형에 근거한 출제기준(안)_회원학회(2차 의견수렴) 끝.

한국보건의료인국가시험원장

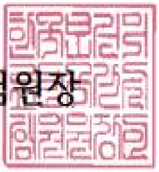

대리 **김준기** 연구개발부장 **임미경** 연구개발본부장 전결 04/29 **이경신**

협조자

시행 연구개발부-1062 (2021.04.29.) 접수 ( )

우 05103 서울특별시 광진구 자양로 45 (자양동) / www.kuksiwon.or.kr

전화 02-2087-8956 /전송 02-2087-8885 / tontates@kuksiwon.or.kr / 공개

- 소중한 개인정보 우리가 지켜야 합니다. -

【부록12】한국간호과학회, 한국간호대학(과)장협의회, 한국전문간호대학(부)장 협의회, 병원간호사회 의견조사

국민이 신뢰하고 감동하는 시험평가기관

한국보건의료인국가시험원

수 신 수신자참조

(경유)

제 목 2020년도 국시원 자유주제 위탁연구「간호사 국가시험의 통합모형에 근거한 출제기준개발 기초연구」출제기준(안) 개발을 위한 의견 요청 (3차)

---

1. 귀 기관의 무궁한 발전을 기원합니다.
2. 한국보건의료인국가시험원은 대한간호협회와 계약을 체결하여 2020년도 자유주제 위탁연구「간호사 국가시험의 통합모형에 근거한 출제기준개발 기초연구」를 진행하고 있습니다.
3. 신규 간호사 직무에 기반한 통합형 간호사 국가시험 출제기준(안) 개발을 위해 의견을 조회하오니 붙임의 파일을 검토하시어 의견 작성을 요청드립니다.

가. 연구 개요

1) 연구과제명 : 간호사 국가시험의 통합모형에 근거한 출제기준개발 기초연구

2) 주관연구기관 : 사단법인 대한간호협회

3) 책임연구자 : 김경희 교수(중앙대학교)

나. 의견요청 개요

1) 8개 통합 실무영역과 연계된 134개 신규간호사 실무항목 및 481개 범주의 지식항목에 대한 회원학회의 1차, 2차 의견을 검토 후 의견 작성

2) 간호사 국가시험 출제기준(안)에 대한 회원학회의 의견을 검토 후 의견 작성

3) 8개 통합 실무영역과 연계된 134개 신규간호사 실무항목 및 481개 범주의 지식항목에 대한 회원학회의 제안사항을 검토 후 의견 작성

4) 간호사 국가시험의 앞으로의 방향에 대한 의견 작성

다. 회신기한 : 2021. 6. 4.(금)까지

라. 회신처 : 책임연구자 김경희 교수(kyung@cau.ac.kr)

붙임 1. 의견조회 안내문

2. 회원학회 1,2차의견수렴(8개 통합 실무영역 및 134개 신규간호사 실무항목과 연계된 지식항목481개) 1부.

3. 회원학회 1,2차 출제기준(안) 1부.

4. 회원학회 제안사항 1부. 끝.

한국보건의료인국가시험원장

수신자 한국간호과학회, 한국간호대학(과)장협의회, 한국전문대간호학(부)장협의회, 병원간호사회

대리 **김준기** 연구개발부장 **임미경** 연구개발본부장 전결 05/21 **이경신**

협조자

시행 연구개발부-1289 (2021.05.21.) 접수 ( )

우 05103 서울특별시 광진구 자양로 45 (자양동) / www.kuksiwon.or.kr
전화 02-2087-8956 /전송 02-2087-8885 / tontates@kuksiwon.or.kr / 비공개(5)

- 소중한 개인정보 우리가 지켜야 합니다. -
