# Supplementary material for: Suggestion for item allocation to 8 nursing activity categories of the Korean Nursing Licensing Examination: a survey-based descriptive study
Source: J Educ Eval Health Prof. 2023 Jun 12;20:18. doi: 10.3352/jeehp.2023.20.18 (PMC10352010; doi:10.3352/jeehp.2023.20.18)
Supplement: Supplementary file 4 — Supplement 3. Survey questionnaires sent to 7 academic societies on the number of items in each nursing activity category of the Korean Nursing Licensing Examination. [file jeehp-20-18-suppl3.pdf]

전문가 타당도 평가 설문지

안녕하십니까?
바쁘신 중에 귀중한 시간을 내어 주셔서 고맙습니다.
대한간호협회 간호사국가시험위원회에서는 한국보건의료인국가시험원 2020년도 자유주제 위탁연구 과제 '간호사 국가시험의 통합 모형에 근거한 출제기준개발 기초연구'를 진행하고 있습니다. 출제기준을 개발하기 위해서는 최신의 간호사 직무분석이 선행되어야 하므로, 그동안 문헌고찰과 선행연구를 통해서 **8개 영역별로 개발된 134개의 신규간호사 실무항목**에 대해 전문가분들께 **타당도(적 절성)**를 여쭙고자 합니다. **134개의 신규간호사 실무항목**에 대해서 **빠짐없이** 응답해 주시기를 당부드립니다. **2021년 3월 2일(화)까지 응답**해 주셔서 기간 내에 전문가 타당도 평가 설문이 회수될 수 있도록 협조해 주시기 바랍니다. 바쁘신 중에도 설문에 성실히 응 해 주심에 감사드립니다.

설문의 목적을 이해하고 참여에 동의합니다. □

2021. 2.22. 책임연구자 김경희
공동연구자 강소영 강윤희 권영란 김현정 송영신 조주연 최미영

1. 귀하가 근무하는 기관은 다음 중 어디에 해당합니까?
① 상급종합병원 ② 종합병원 ③ 학교(보건교사) ④ 보건소 ⑤ 기타

2. 귀하의 간호사로서의 근무기간은 다음 중 어디에 해당합니까?
① 1년 미만 ② 1 ~3년 미만 ③ 3 ~ 5년 미만 ④ 5 ~ 7년 미만 ⑤ 7년 이상

3. 귀하가 현재 근무하고 있는 부서는 다음 중 어디에 해당합니까?
① 내 · 외과 ② 분만실, 산부인과 ③ 소아청소년과 ④ 정신과 ⑤ 학교 또는 보건소

▪ 다음은 8개 영역별로 개발 된 134개 신규간호사 실무항목 입니다. 각 실무항목의 적절성을 아래에 ✔표 하여 주시고, 수정 의견이 있으시면 기술해주시기 바랍니다.

영역	번호	신규간호사 실무항목	실무항목 적절성<br>1. 매우 타당하지 않음<br>2. 타당하지 않음<br>3. 타당함<br>4. 매우 타당함				수정의견
I. 간호관리와 전문성 향상	1	인수인계 시행	1	2	3	4	
	2	법적 실무범위 내에서 간호 수행	1	2	3	4	
	3	기록시 표준화된 약어 사용	1	2	3	4	
	4	지침에 따라 간호기록	1	2	3	4	
	5	입원, 전동, 퇴원	1	2	3	4	
	6	장비를 적절하고 안전하게 사용	1	2	3	4	
	7	간호단위 물품교환체계에 따른 물품관리	1	2	3	4	
	8	질 향상(QI) 활동에 참여	1	2	3	4	
	9	간호사업 평가 관련 업무수행 (도구개발, 자료조사, 분석, 비교 및 사업개선)	1	2	3	4	
	10	간호전문직 윤리 준수와 역할	1	2	3	4	
	11	대상자의 개인정보 및 사생활 보호	1	2	3	4	
	12	대상자에게 치료 및 절차에 따라 적절한 설명을 하고 동의를 획득 하였는지 확인	1	2	3	4	
	13	환자의 권리와 책임에 관하여 대상자에게 교육 제공	1	2	3	4	

	14	억제대 사용시 법적 및 윤리적 간호	1	2	3	4	
	15	일차의료기반의 보건간호	1	2	3	4	
	16	지역사회 간호사업의 법적 기준 및 지침에 따른 활동 참여	1	2	3	4	
	17	사례관리활동 참여	1	2	3	4	
	18	업무를 조직화하여 일을 효율적으로 관리	1	2	3	4	
	19	환자분류체계 관련 정보수집 및 활용	1	2	3	4	
	20	간호대상에 적합한 다양한 자원 및 매체선택	1	2	3	4	
	21	취약가족에 필요한 지역사회 자원활용	1	2	3	4	
	22	전문직간 협업	1	2	3	4	

영역	번호	신규간호사 실무항목	실무항목 적절성<br>1. 매우 타당하지 않음<br>2. 타당하지 않음<br>3. 타당함<br>4. 매우 타당함				수정의견
II. 안전과 감염관리	23	안전한 환경 제공	1	2	3	4	
	24	감염관리	1	2	3	4	
	25	위험물질과 유해물질관리	1	2	3	4	

영역	번호	신규간호사 실무항목	실무항목 적절성<br>1. 매우 타당하지 않음<br>2. 타당하지 않음<br>3. 타당함<br>4. 매우 타당함				수정의견
Ⅲ. 위험요인 사정	26	간호계획, 진료지침 수행	1	2	3	4	
	27	대상자의 건강문제에 대해 우선순위 결정	1	2	3	4	
	28	활력징후 사정	1	2	3	4	

Ⅲ. 위험요인 사정	29	신생아간호	1	2	3	4	
	30	영아간호	1	2	3	4	
	31	유아간호	1	2	3	4	
	32	학령전기간호	1	2	3	4	
	33	학령기간호	1	2	3	4	
	34	청소년간호	1	2	3	4	
	35	폐경기 여성간호	1	2	3	4	
	36	노인간호	1	2	3	4	
	37	성건강간호	1	2	3	4	
	38	건강력 사정	1	2	3	4	
	39	신체검진 수행 및 결과해석	1	2	3	4	
	40	생식기 건강사정	1	2	3	4	
	41	태아 건강사정 및 간호	1	2	3	4	
	42	신생아 건강사정 및 간호	1	2	3	4	
	43	고위험신생아 건강사정 및 간호	1	2	3	4	
	44	심폐소생술 간호	1	2	3	4	
	45	응급간호	1	2	3	4	
	46	진단검사간호	1	2	3	4	
	47	수술간호	1	2	3	4	
	48	산전간호와 교육	1	2	3	4	
	49	분만 중 간호와 교육	1	2	3	4	
	50	산후관리와 교육	1	2	3	4	
	51	고위험임부 간호	1	2	3	4	
	52	고위험산부간호	1	2	3	4	
	53	고위험산모간호	1	2	3	4	

영역	번호	신규간호사 실무항목	실무항목 적절성<br>1. 매우 타당하지 않음<br>2. 타당하지 않음<br>3. 타당함<br>4. 매우 타당함				수정의견
Ⅳ. 기본간호	54	체온유지간호	1	2	3	4	
	55	세척(irrigation)(방광, 귀, 눈) 수행	1	2	3	4	
	56	개인위생간호	1	2	3	4	
	57	섭취 및 배설량 사정과 간호	1	2	3	4	
	58	영양사정 및 관리	1	2	3	4	
	59	질환별 영양문제의 사정 및 관리	1	2	3	4	
	60	섭취장애 대상자 사정 및 간호	1	2	3	4	
	61	배뇨장애 대상자 사정 및 간호	1	2	3	4	
	62	투석대상자 관리	1	2	3	4	
	63	요루 및 장루 관리	1	2	3	4	
	64	기관절개부 관리	1	2	3	4	
	65	배변관리	1	2	3	4	
	66	수면과 휴식 간호	1	2	3	4	
	67	피부통합성 사정 및 간호	1	2	3	4	
	68	이동간호	1	2	3	4	
	69	활동과 자기돌봄장애 사정 및 간호	1	2	3	4	
	70	외과적 장치 관리	1	2	3	4	
	71	척추손상 및 질환대상자의 간호	1	2	3	4	

	72	관절대치술 환자간호	1	2	3	4	
영역	번호	신규간호사 실무항목	실무항목 적절성<br>1. 매우 타당하지 않음<br>2. 타당하지 않음<br>3. 타당함<br>4. 매우 타당함				수정의견
	73	호흡기능장애 대상자 간호	1	2	3	4	
	74	호흡증진중재	1	2	3	4	
	75	호흡보조장치 관리	1	2	3	4	
	76	중심정맥관 관리	1	2	3	4	
	77	태아질식 증상과 징후 사정 및 간호	1	2	3	4	
	78	고위험신생아 보육기 적용간호	1	2	3	4	
	79	호흡재활관리	1	2	3	4	
	80	심전도관리	1	2	3	4	
	81	순환보조장치관리	1	2	3	4	
	82	동맥관관리	1	2	3	4	
	83	조직관류장애 대상자 사정 및 간호	1	2	3	4	

V. 생리적 통합유지	84	체액전해질 불균형 사정 및 간호	1	2	3	4	
	85	활동지속성 장애 대상자 사정 및 간호	1	2	3	4	
	86	정맥순환증진장치 간호	1	2	3	4	
	87	혈액기능장애 대상자 간호	1	2	3	4	
	88	순환기능장애 대상자간호	1	2	3	4	
	89	심장수술후 간호	1	2	3	4	
	90	소화기능장애 대상자 간호	1	2	3	4	
	91	배뇨장애를 가진 대상자 간호	1	2	3	4	
	92	당질대사장애 대상자 간호	1	2	3	4	
	93	당뇨병 합병증 예방을 위한 간호	1	2	3	4	
	94	내분비계장애 대상자 간호	1	2	3	4	
	95	생식기 질환/생식기 건강문제를 가진 대상자 간호	1	2	3	4	
	96	면역손상 대상자 사정 및 간호	1	2	3	4	
	97	감각기능장애를 가진 대상자 사정 및 간호	1	2	3	4	
	98	두개내압 상승 환자의 간호	1	2	3	4	

	99	신경계 질환별 간호중재	1	2	3	4	
	100	운동기능장애 간호중재	1	2	3	4	
	101	상처간호 수행 및 드레싱 교환	1	2	3	4	
	102	화상환자 간호중재	1	2	3	4	
	103	피부질환 대상자의 간호중재	1	2	3	4	
	104	배액장치관리	1	2	3	4	
	105	계통별 신생물 질환 대상자의 간호	1	2	3	4	
	106	통증간호	1	2	3	4	
	107	재활간호서비스	1	2	3	4	
	108	(법정) 감염질환자의 전파예방 및 간호	1	2	3	4	

영역	번호	신규간호사 실무항목	실무항목 적절성<br>1. 매우 타당하지 않음<br>2. 타당하지 않음<br>3. 타당함<br>4. 매우 타당함				수정의견
VI. 약물 및 비경구요법	109	투약의 적절성과 정확성	1	2	3	4	
	110	약물 투여에 필요한 계산 시행	1	2	3	4	
	111	정맥주입장치 관리	1	2	3	4	
	112	약품관리	1	2	3	4	
	113	말초정맥관 삽입, 유지, 제거	1	2	3	4	
	114	대상자에게 약물에 대해 교육	1	2	3	4	
	115	고위험 약품관리(마약관리)	1	2	3	4	

영역	번호	신규간호사 실무항목	실무항목 적절성<br>1. 매우 타당하지 않음<br>2. 타당하지 않음<br>3. 타당함<br>4. 매우 타당함				수정의견
Ⅶ. 심리사회적 통합유지	116	이상행동 사정 및 간호	1	2	3	4	
	117	폭력의 잠재성 평가 및 예방	1	2	3	4	
	118	학대 또는 방임 대상자를 확인하고 적절하게 중재	1	2	3	4	
	119	중독 사정 및 간호	1	2	3	4	
	120	정신사회건강문제 간호 및 교육	1	2	3	4	
	121	정신질환자 간호	1	2	3	4	
	122	아동정신질환자 간호	1	2	3	4	
	123	임종간호와 교육	1	2	3	4	
	124	치료적 의사소통 기법	1	2	3	4	

영역	번호	신규간호사 실무항목	실무항목 적절성<br>1. 매우 타당하지 않음<br>2. 타당하지 않음<br>3. 타당함<br>4. 매우 타당함				수정의견
Ⅷ.	125	(지역사회)건강교육계획	1	2	3	4	

건강증진 및 유지	126	건강증진 및 유지관리에 대한 정보(예방접종 등) 제공	1	2	3	4	
	127	고위험 건강 행위의 예방 및 치료에 대한 정보(금연, 안전한 성 행위, 바늘교환) 제공	1	2	3	4	
	128	성 건강증진 간호	1	2	3	4	
	129	문화간호	1	2	3	4	
	130	산업과 환경 간호	1	2	3	4	
	131	재난간호	1	2	3	4	
	132	치료계획을 결정하기 위해 가족 역동성(family dynamics)(가족 구조, 결속, 의사소통, 경계, 대처기전) 사정	1	2	3	4	
	133	가정환경에서 대상자를 관리할 수 있는 역량(장비, 지역사회 자원) 평가	1	2	3	4	
	134	건강위험요인 사정과 교육	1	2	3	4	

귀하께서 응답해 주신 자료는 간호사국가시험의 발전에 귀중한 자료가 될 것입니다. 설문에 응답해 주셔서 진심으로 감사드립니다.
